# Naphthalimide‐Fused Dipyrrins: Tunable Halochromic Switches and Photothermal NIR‐II Dyes

**DOI:** 10.1002/advs.202105886

**Published:** 2022-02-17

**Authors:** Yogesh Kumar Maurya, Piotr J. Chmielewski, Joanna Cybińska, Bibek Prajapati, Tadeusz Lis, Seongsoo Kang, Seokwon Lee, Dongho Kim, Marcin Stępień

**Affiliations:** ^1^ Wydział Chemii Uniwersytet Wrocławski ul. F. Joliot‐Curie 14 Wrocław 50‐383 Poland; ^2^ PORT – Polski Ośrodek Rozwoju Technologii ul. Stabłowicka 147 Wrocław 54‐066 Poland; ^3^ Department of Chemistry and Spectroscopy Laboratory for Functional *π*‐Electronic Systems Yonsei University Seoul 03722 Korea

**Keywords:** dipyrrins, donor–acceptor systems, halochromism, near‐infrared dyes, photothermal agents

## Abstract

A family of tunable halochromic switches is developed using a naphthalimide‐fused dipyrrin as the core *π*‐conjugated motif. Electronic properties of these dipyrrins are tuned by substitution of their alpha and meso positions with aryl groups of variable donor–acceptor strength. The first protonation results in a conformational change that enhances electronic coupling between the dipyrrin chromophore and the meso substituent, leading to halochromic effects that occasionally exceed 200 nm and switch the absorption between the near‐infrared (NIR)‐I and NIR‐II ranges. A NIR‐II photothermal effect, switchable by acid–base chemistry is demonstrated for selected dipyrrins. Further protonation is possible for derivatives bearing additional amino groups, leading to up to four halochromic switching step. The most electron‐rich dipyrrins are also susceptible to chemical oxidation, yielding NIR‐absorbing radical cations and closed‐shell dications.

## Graphical abstract

Naphthalimide‐fused dipyrrins decorated with aryl groups of variable electron‐donating strength are a new class of tunable near‐infrared dyes that can be switched between near‐infrared (NIR)‐I and NIR‐II absorptions via one or more protonation steps. The NIR‐II absorbing dipyrrinium cations can be employed as halochromic photothermal agents, which can be reversibly activated by addition of an acid or base.

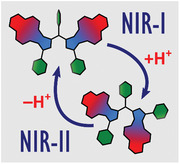

## 1 Introduction

*π*‐Conjugated organic chromophores with strong absorptions in the near‐infrared (NIR) region have drawn considerable interest because of their diverse applications in biomedical research, materials science, and related fields.^[^ ^1^, ^2^, ^3^, ^4^ ^]^ NIR‐active organic dyes are based on various structural motifs, for example, rylenes, cyanines, squaraines, and porphyrinoids,^[^ ^5^, ^6^, ^7^, ^8^, ^9^ ^]^ which often show additional intense absorption in the visible region. In a given class of organic dyes, bathochromic shifts of absorptions into the NIR region are typically achieved by extending the *π*‐system of the chromophore or by incorporation of donor and acceptor groups into the parent structure.^[^ ^10^, ^11^ ^]^ These two strategies have been explored in diverse families of dyes, such as cyanines, rylenes, BODIPYs, expanded porphyrins, and porphyrin tapes, all of which can be engineered to yield near‐infrared activity.^[^ ^4^, ^12^, ^13^, ^14^, ^15^, ^16^, ^17^, ^18^, ^19^, ^20^, ^21^, ^22^, ^23^, ^24^, ^25^ ^]^ In spite of this progress, switchable NIR dyes that will change their absorption and emission signatures in response to external stimuli remain relatively rare in spite of their potential uses in bioimaging, sensing, and actuation. Base‐induced halochromism was reported by the Würthner group in bay‐substituted PDI dyes,^[^ ^26^, ^27^ ^]^ some of which were also found to produce distinct hydrochromism.^[^ ^27^ ^]^ Solvatochromic switching between visible and NIR absorption and emission is also possible for systems that undergo significant solvent‐dependent conformational changes.^[^ ^28^ ^]^

**Figure 1:**
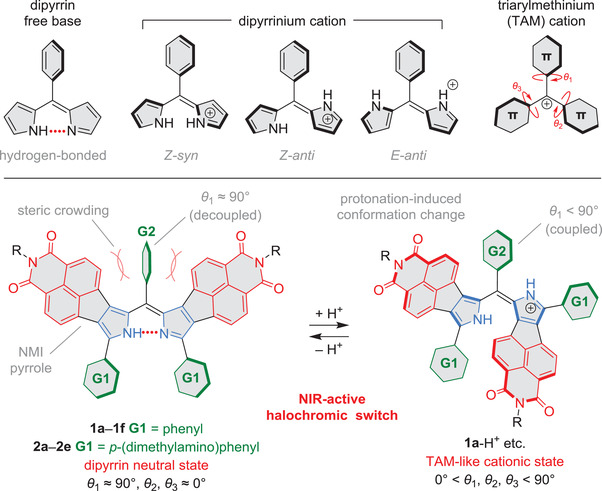
*π*‐extended dipyrrins as NIR‐active halochromic switches.

Inspired by these examples, we reasoned that judicious use of conformational switching may also enhance halochromic responses of NIR dyes. Examples of profound protonation‐induced conformational changes have been particularly well documented in porphyrinoid chemistry,^[^ ^29^ ^]^ occasionally enabling multi‐state switching of aromaticity.^[^ ^30^ ^]^ However, the complexity of the conformational dynamics and prototropic equilibria in these systems makes it difficult to design and control protonation‐induced switching equilibria. We accordingly considered exploring a smaller oligopyrrole motif, the dipyrrin, which has a single protonation site and well‐defined acid–base chemistry.^[^ ^31^ ^]^ Free‐base dipyrrins adopt a *Z*‐*syn* configuration stabilized by an intramolecular hydrogen bond, but become conformationally flexible upon protonation (**Figure** **1**).^[^ ^32^, ^33^, ^34^ ^]^ The resulting dipyrrinium cation may be seen as a special case of triarylmethinium (TAM) cation^[^ ^35^, ^36^, ^37^ ^]^ bearing two strongly conjugated 2‐pyrrolyl groups. TAM cations have propeller‐like structures, and can be transformed into NIR‐absorbing dyes by *π*‐extension of individual aryl groups.^[^ ^38^, ^39^, ^40^, ^41^, ^42^ ^]^

Here we report on the halochromism and electronic structure properties of a family of dipyrrins bearing fused naphthalimide (NMI) moieties (Figure 1). NMI pyrroles^[^ ^43^ ^]^ have been previously shown to be versatile building blocks for the synthesis of electron‐deficient porphyrins,^[^ ^43^, ^44^, ^45^ ^]^ azacoronenes,^[^ ^46^, ^47^, ^48^ ^]^ bipyrroles,^[^ ^49^, ^50^, ^51^ ^]^ and polymers.^[^ ^52^ ^]^ The family comprises two series of dipyrrins **1a**–**1f** and **2a–2e**, bearing respectively phenyl, and *p*‐(dimethylamino)phenyl groups at the pyrrolic *α* (or 1,9) positions (**G1**), and aryl substituents with variable donor/acceptor strength at the meso bridge (**G2**). Aminophenyl substituents have been previously shown to affect electronic gaps of NIR dyes, and can also serve as additional protonation and oxidation sites.^[^ ^4^, ^53^, ^54^, ^55^, ^56^, ^57^ ^]^ The placement of NMe_2_ groups at the para positions of **G2** (rather than ortho or meta) ensures the strongest possible coupling with the dipyrrin without introducing steric hindrance. Thus, geometrical factors affecting the conformations of free‐base and protonated dipyrrins should be similar in the **1a**–**e** and **2a**–**e** series. In the present design, the NMI units do not only impart a distinct donor–acceptor character to the dipyrrin but also create steric hindrance around the *meso*‐aryl group **G1**. As a result of this crowding, the **G1** group is rotated perpendicular to the plane of the dipyrrin and is largely decoupled from the oligopyrrole *π* system. Upon protonation, the conformation of the dipyrrinium cation changes enabling a more TAM‐like conjugation that includes the meso substituent. Depending on the combination of **G1** and **G2** groups, the optical gaps of the neutral and cationic forms of the dipyrrins are differently affected, yielding highly variable halochromic responses extending into the NIR‐II optical window. Substitution with dimethylaminophenyl groups enables additional protonation events of the corresponding dipyrrins and makes them highly susceptible to electrochemical and chemical oxidation.

## 2 Results and Discussion

### 2.1 Synthesis and Characterization

**Scheme 1:**
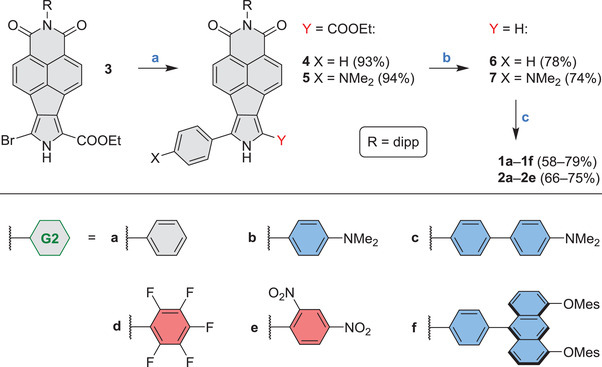
Synthesis of *π*‐extended donor–acceptor dipyrrins. Reagents and conditions: a) phenylboronic acid (for 4), 4‐(*N*,*N*‐dimethylamino)phenylboronic acid pinacol ester (for 5), K_2_CO_3_, Pd(dba)_2_, XPhos, THF/water (1/0.1, v/v), 100 °C; b) KOH, (CH_2_OH)_2_, MW (100 W, 190 °C, PowerMax); c) i) arylaldehyde, TFA, CH_2_Cl_2_, or CHCl_3_, RT; ii) DDQ, RT.

Initial design of the dipyrrin targets was guided by density functional theory (DFT) calculations, which provided semi‐quantitative information on electronic gaps, halochromism, and charge‐transfer (CT) character of these chromophores. Dipyrrins **1a**–**1f** and **2a**–**2e**, selected for further investigations on the basis of their predicted properties, were prepared from the common monopyrrole building block **3**, following a three‐step procedure outlined in **Scheme** **1** (for details, see the Supporting Information). Bromopyrrole **3** was subjected to Suzuki coupling followed by decarboxylation and saponification, to produce *α*‐arylpyrroles **6** and **7**, bearing respectively phenyl and *p*‐(dimethylamino)phenyl substituents. Trifluoroacetic acid (TFA)‐catalyzed condensation^[^ ^58^ ^]^ of **6** and **7** with appropriate arylaldehydes, followed by oxidation with DDQ, furnished the desired dipyrrins, **1a**–**1f** and **2a**–**2e** in satisfactory yields. The dipyrrins yielded ^1^H NMR and ^13^C NMR, and mass spectrometric analyses that were consistent with the proposed structures. In particular, the NH resonances of all dipyrrins appeared at ≈14–15 ppm, indicating the presence of an intramolecular hydrogen bond. The upfield relocation of one of the naphthalene resonances (≈4.5–5.0 ppm) is consistent with the shielding effect of the *meso*‐aryl substituent, which is held in perpendicular alignment in between the edges of the two NMI units.

**Figure 2:**
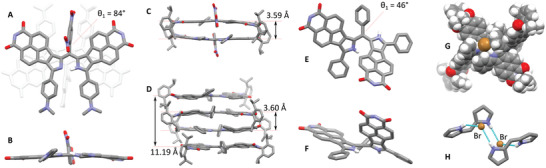
Molecular structures and packing diagrams of selected dipyrrins revealed by XRD analyses. A,B) Molecular geometry of **2e** with H atoms, dipp groups, and solvent molecules removed for clarity. The other molecule in the *π*‐stacked dimer (light gray) is shown in panel A. C) Side view of the solid‐state *π*‐stacked dimer of **2e**. D) Side view of the solid‐state *π*‐stacked tetramer of **1a**. Distances have been measured between mean plane of dipyrrins (excluding substituents). E,F) Molecular geometry of [**1a**‐H]Br with the bromide ion, H atoms, dipp groups, and solvent molecules removed for clarity. G,H) Structure of the cyclic hydrogen‐bonded assembly found in the crystal of [**1a**‐H]Br.

Single crystals of free‐base dipyrrins **1a**, **1b**, **1e**, and **2e**, suitable for X‐ray diffraction analysis, were obtained using slow vapor diffusion of *n*‐hexane or methanol into concentrated solutions of the dipyrrins in chloroform or other solvents. In the solid state, all dipyrrins showed near planar conformations characterized by small *θ*_2_ and *θ*_3_ angles (**Figure** **2** and **Table** **1**, see Supporting Information for additional data). In all cases, the large torsional angles *θ*_1_ (73–84°), and relatively long *C*_meso_–*C*_ipso_ distances (1.49–1.51 Å) are indicative of disrupted conjugation between the dipyrrin and the meso substituent. Free bases **1b**, **1e**, and **2e** form centrosymmetric *π*‐stacked dimers in the solid state, characterized by an antiparallel alignment of the dipyrrins, and relatively small interplanar distances of ≈3.6 Å (Figure 2A,C). In the crystals of **1a**, centrosymmetric *π*‐stacked tetramers were observed consisting of two types of symmetry‐independent molecules (Figure 2D). The close stacking distances in all these assembles are achieved in spite of relatively bulky substitution of the dipyrrin cores and are likely promoted by the dipolar character of the NMI‐pyrrole subunits.

**Table 1:** Selected torsion angles (°), bond lengths (Å), and mean‐plane deviations (Å) for dipyrrins in the neutral and protonated forms obtained from the X‐ray crystal structures

Entry	*θ*_1_ ^a)^	*θ*_2_	*θ*_3_	C—N ^b)^	m.p.d ^c)^	*θ*_1_ ^d)^
**1a**	74.2	11.7	1.2	—	0.104	80.61
**1b**	75.8	3.0	0.4	1.381	0.060	60.81
**1e**	73.4	4.8	0.6	—	0.088	79.86
**2e**	84.2	9.8	2.1	1.371	0.057	82.86
[**1a**‐H]Br	45.5	25.6	30.8	—	—	39.21
[**1b**‐H]Cl	28.8	34.7	26.6	1.350	—	28.23

^a)^ average values

^b)^ bond length of C—NMe_2_ substituents

^c)^ mean plane deviation considering 31 atoms (see Figure S8, Supporting Information, for definition)

^d)^ *meso*‐aryl angle from DFT calculations.

Diffraction‐quality crystals of dipyrrinium salts [**1a**‐H]Br and [**1b**‐H]Cl were obtained from chloroform solutions of respective free bases acidified respectively with HBr and HCl. In the salts, the dipyrrinium cations [**1a**‐H]^+^ and [**1b**‐H]^+^ were found to adopt the Z‐anti conformation, with one of the pyrrole units turned away from its original orientation in the free base (Figure 2E–F). This change results in a significant reduction of the *θ*_1_ torsion (to 46° and 29° respectively), permitting a more efficient communication with the meso substituent. The resulting propeller conformations of the dipyrrinium cations resemble those adopted by TAM cations. The latter analogy is especially valid for [**1b**‐H]^+^, which shows a more flattened structure and near equalization of C—C bond lengths around the meso carbon (1.419, 1.427, and 1.431 Å). In the solid state, the salts form cyclic hydrogen‐bonded assemblies consisting of two dipyrrinium cations bridged by two halide anions (Figure 2G–H).

The conformational behavior of protonated **1b** was investigated in solution using ^1^H NMR spectroscopy (Figure S23, Supporting Information). When treated with 1 equiv of camphorsulfonic acid, **1b** produced a spectrum consistent with the formation of a monocation, [**1b**‐H]^+^, which apparently adopts the Z‐anti conformation analogous to that found in the solid state. In particular, two NH resonances of equal intensities were found at 12.60 and 12.39 ppm, respectively. These signals were shifted to higher field relative to the NH signal of the free‐base **1b** (13.98 ppm), consistent with the absence of an intramolecular hydrogen bond in the monocation. Some of the signals in the 6–9 ppm range were dynamically broadened, but the overall number of observed resonances was consistent with rapid flipping of pyrrole units in the Z‐anti conformer. A different spectrum was obtained upon addition of dilute sulfuric acid, indicating the formation of a higher protonated form, [**1b**‐H_2_]^2+^. Two NH resonances were also observed for the latter species, while the number of aromatic resonances appeared to correspond to slow conformational self‐exchange.

**Figure 3:**
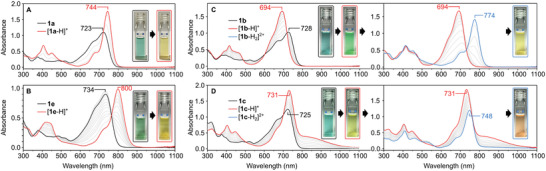
Absorption spectral changes observed for A) **1a**, B) **1e**, C) **1b,** and D) **1c** in CH_2_Cl_2_ seen upon addition of TFA. The second protonation of **1b** is induced by addition of a dilute solution of sulfuric acid in dichloromethane.

**Figure 4:**
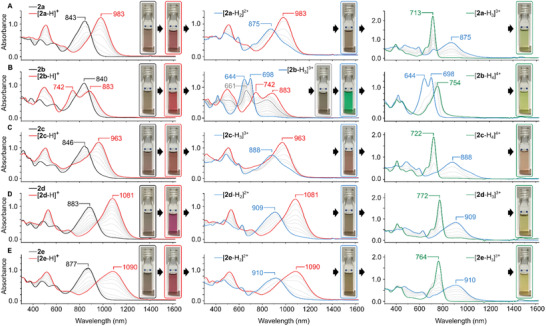
Absorption spectral changes observed for A) **2a**, B) **2b**, C) **2c 2d**, and D) **2e** in CH_2_Cl_2_ seen upon addition of TFA. The second protonation of **2b** is induced by addition of a dilute solution of sulfuric acid in dichloromethane.

*π*‐Extended dipyrrins **1a**–**1e** form emerald‐green solutions in dichloromethane (**Figure** **3** and Figure S20, Supporting Information and **Table** **2**). Their absorption spectra feature one weakly structured band, with a maximum in the 723–734 nm range and an extinction coefficient in the order of 10^4^ dm^3^ mol^−1^ cm^−1^. Such weak dependence of the electronic gap on *meso*‐substitution agrees well with the weak coupling expected for the free base dipyrrins. In the **2a**–**2e** series, the presence of electron donating *α* substituents results in a significant red shift of the principal band further into the NIR‐I region (*λ*_max_ = 840 to 883 nm) and somewhat greater sensitivity to *meso*‐substitution in comparison with **1a**–**1e** (**Figure** **4**). An analogous red shift is observed in the parent monopyrroles of both series (*λ*_max_ = 483 and 536 nm for **6** and **7**, respectively, see the Supporting Information for additional data). In both series, the largest red shift is caused by electron‐withdrawing *meso*‐aryl groups, that is, pentafluorophenyl and 2,4‐dinitrophenyl. Unlike the parent monopyrroles **6**–**7**, which show noticeable positive solvatochromism in the visible range, the dipyrrins are only weaky solvatochromic (Figures S15–S19 and S34, Supporting Information). Dipyrrins **1a**–**1e** are weakly fluorescent in the NIR range (QY < 0.1%, *τ* = 2 to 3 ns in toluene, Figures S34–S35 and Table S8, Supporting Information). Emission maxima for **1a**–**1c** are at ≈760 nm in toluene, while those of **1d** and **1e** are bathochromically shifted to ≈780 nm. All species are moderately solvatofluorochromic (*λ*_max_^em^ ≈ 780 nm for **1a**–**1c** and 810 nm for **1d** and **1e**). Nonradiative rate constants determined for the entire series are by 1 to 2 orders of magnitude larger than the corresponding radiative constants (Figure S35 and Table S9, Supporting Information), implying efficient nonradiative decay channels, possibly involving vibrational relaxation or the CT character of the excited states.

**Table 2:** Photophysical Properties

Dipyrrins	Neutral ^a)^ *λ*_max_, [nm]	Protonated stages [nm] ^b)^		Halochromic Δ*E*_g_ [eV] ^c)^	KS H‐L_g_ ^d)^ [eV] [N P^−1^]	Optical H‐L_g_ [eV] [N P^−1^] ^e)^	Halochromic Δ*E* [eV] ^f)^	Osc. Str. (*f*) [N P^−1^]
		first	last					
1a	723	744	–	−0.05	2.02/2.06	1.76/1.73	−0.03	1.22/0.68
1b	728	694	774^h)^	0.08	2.03/2.18	1.68/1.86	0.18	0.30/0.50
1c	725	840^g)^	748	−0.23	1.68/1.48	1.76^i)^/1.35	−0.41	1.20/0.82
1d	733	797	–	−0.13	1.96/1.90	1.72/1.63	−0.09	1.21/0.78
1e	734	800	–	−0.14	1.98/1.84	1.72/1.56	−0.16	1.16/0.69
2a	843	983	713	−0.21	1.52/1.42	1.30/1.19	−0.11	0.75/0.53
2b	840	742, 883	754^h)^	−0.07	1.57/1.56	1.34/1.26	−0.08	0.74/0.40
2c	846	963	722	−0.18	1.52/1.46	1.31/1.21	−0.10	0.74/0.46
2d	883	1081	772	−0.25	1.44/1.31	1.25/1.13	−0.12	0.73/0.62
2e	877	1090	764	−0.28	1.47/1.22	1.25/1.05	−0.20	0.69/0.56

^a)^ Data recorded in CH_2_Cl_2_,

^b)^ Absorption maxima from the titration experiments using TFA as acid

^c)^ Experimental band‐gap change upon protonation

^d)^ KS energy gap (H→L) calculated at the B3LYP‐GD3BJ/6‐31G(d,p)/PCM(DCM) level of theory

^e)^ TD‐DFT based energy gap (H→L) calculated at the B3LYP‐GD3BJ/6‐31G(d,p)/PCM(DCM) level of theory

^f)^ TD‐DFT based band‐gap change upon protonation

^g)^ approximate position of CT band

^h)^ last protonation state was achieved using H_2_SO_4_ as acid source

^i)^ TD‐DFT‐based H‐1→L energy gap. N = neutral form, P = first protonated form.

To acquire insights into excited‐state dynamics of **1a** and **1b**, we performed the femtosecond transient absorption (fs‐TA) measurements. The excitation pump at 670 nm was employed to probe the TA spectra in the visible region. The fs‐TA spectra of **1a** and **1b** in toluene displayed ground‐state bleaching (GSB) signals corresponding to their steady‐state absorption spectra in the region of 630–760 nm overlaid with very weak stimulated emission (SE) bands in the NIR region, as well as the excited‐state absorption, features below 630 nm (Figure S35, Supporting Information). Both species exhibited similar TA decay profiles with time constants of ≈4 to 6 ps and ≈2.0 to 2.3 ns, respectively. Regarding the monotonous spectral evolution in the observed time window, we assigned the faster decay to the structural relaxation from two dipyrrin moieties and the latter to the excited singlet state lifetime. The latter decay component matches well with the fluorescence lifetime obtained by the TCSPC measurement, and is followed by the residual triplet state population. The TA spectra of **1a** in benzonitrile showed comparable spectral evolution and dynamics to those in toluene. Some disparities were, however, observed for **1b**: the TA spectral evolution in the polar medium revealed an initial rise and broadening of the GSB band in the region of 550–725 nm with a decreased GSB signal below 740 nm with a time constant of 14 ps. After that, the ensuing TA spectra monotonically decreased with a time constant of 30 ps. The accelerated excited‐state dynamics and spectral evolution indicate the formation of a CT state from the local singlet excited‐state, and then charge recombination to the ground state.^[^ ^59^ ^]^ These results show that, in the more polar benzonitrile, the formation of the CT state of **1b** may be attributed to the effect of the electron‐donating meso substituent.

Acid titrations of the NMI dipyrrins revealed a highly variable behavior dependent on the identity of both meso and *α* substituents (Figures 3 and 4 and Figures S20 and S21, Supporting Information, Table 2). When treated with trifluoroacetic acid (TFA) in CH_2_Cl_2_, the all‐phenyl derivative **1a** produced the corresponding dipyrrinium cation [**1a**‐H]^+^. The latter species showed a relatively small relocation of the low‐energy absorption band to 744 nm, corresponding to a small change of the energy gap of Δ*E*_g_ = −0.05 eV, which was accompanied by a notable increase of maximum absorbance. Analogs containing electron‐withdrawing meso groups, **1d** and **1e**, showed more significant positive halochromism, with the principal band shifted up to ≈800 nm into the NIR range. The [**1f**‐H]^+^ cation, for which a CT band had been predicted by TD‐DFT, showed an absorption spectrum very similar to that of [**1a**‐H]^+^, with no observable CT features. Two‐step protonation was observed for **1b**, bearing the *p*‐dimethylaminophenyl *meso*‐substituent. The first protonation induced a negative halochromic effect (from 728 to 694 nm), whereas a marked shift to longer wavelengths (from 694 to 774 nm) was induced by the second protonation (achieved using sulfuric acid). Apparently, the second protonation takes place at the NMe_2_ group, which thus becomes electron‐deficient, yielding a significant red shift. A two‐stage protonation was also observed for **1c**, which however showed a different behavior. The first protonation produced a spectrum containing a slightly red shifted maximum at 731 nm, which was accompanied by a broader, red‐shifted band with a maximum at ≈820–830 nm. The latter band was found to correspond to a CT transition, as discussed below. The second protonation resulted in a spectrum closely resembling that of [**1a**‐H]^+^, and was consequently assumed to occur at the terminal meso NMe_2_ group. For all members of the **1a**–**1e** series, the spectral changes in the NIR region caused by protonation are accompanied by variation of the visible absorption profiles, which lead to noticeable color changes of the dipyrrin solutions.

Acid titrations of the *α*‐NMe_2_‐substituted dipyrrins **2a**–**2e** produced complex absorption responses in the vis‐NIR range, with a marked bathochromic shift relative to their **1a**–**1e** congeners (Figure 4). The simplest derivative, *meso*‐phenyl‐substituted **2a**, revealed a three‐stage protonation, exhibiting clean isosbestic points, which was ascribed to the availability of two *α*‐NMe_2_ groups. The first protonation step apparently produced the dipyrrinium cation [**2a**‐H]^+^ characterized by an absorption at 983 nm, corresponding to a halochromic change of −0.21 eV. The increase of absorbance induced by first protonation is smaller for **2a** than for **1a**. This trend is preserved for other members of the series and could be semi‐quantitatively reproduced by TD‐DFT calculations. Subsequent protonations resulted in a negative halochromic effect, which reflected the progressive shortening of the conjugation length in the *π* system. The ultimate absorption spectrum of [**2a**‐H_3_]^3+^ (*λ*_max_ = 713 nm) resembles that of [**1a**‐H]^+^ indicating a similar electronic structure. Interestingly, in contrast to electron‐withdrawing groups placed at the meso position, which yield a bathochromic shift of the absorption maximum, alpha substitution with acceptor groups (herein, —NHMe_2_^+^) results in a hypsochromic shift (relative to [**1a**‐H]^+^). **2d** and **2e** showed a qualitatively similar three‐step response to acids, but the positions of absorptions displayed by the protonated forms were red‐shifted relative to those observed for **2a**. The bathochromic shifts for the dipyrrinium cations [**2d**‐H]^+^ and [**2e**‐H]^+^ were particularly large (*λ*_max_ = 1081 and 1090 nm, respectively). The latter value corresponds to the largest positive halochromic effect observed in all dipyrrins (+213 nm or −0.28 eV).

The most complicated behavior was observed upon protonation of **2b** and **2c**, each of which contains four potential protonation sites: the dipyrrin, two *α*‐NMe_2_ groups, and one *meso*‐NMe_2_ group (Figure 4B,C). **2c** was first protonated at the dipyrrin core, yielding the expected dipyrrinium monocation [**2c**‐H]^+^, which however did not display a CT similar to the one observed for [**2a**‐H]^+^. Subsequent addition of acid caused a composite process consisting of initial slight relocation of the maximum to higher wavelengths followed by the appearance of a more blue‐shifted maximum at 888 nm. Comparison with **1c** and **2a** suggests that this stage consists of initial *meso*‐NMe_2_ protonation, which is closely followed by protonation of one of the *α*‐NMe_2_ groups, to yield the trication [**2c**‐H_3_]^3+^. The latter species is ultimately protonated to form the tetracation [**2c**‐H_4_]^4+^, which produces the expected blue‐shifted, narrow band (*λ*_max_ = 722 nm). Interestingly, **2b**, which differs from **2c** by the length of the meso linker between the dipyrrin and the NMe_2_ group, showed a significantly different sequence of acid‐induced spectral changes. The difference is reflected in the particularly rich palette of colors observed during titration (Figure 4). The spectrum of the initial dipyrrinium cation [**2b**‐H]^+^ is already unusual, with two low‐energy absorptions at 742 and 883 nm. Subsequent acidification led to an apparent two‐step process that ended in the formation of an unprecedented green species characterized by a markedly blue‐shifted spectrum with two maxima at 644 and 698 nm. The latter species, which may be assigned to a trication [**2b**‐H_3_]^3+^, undergoes one final protonation, which leads to a spectrum that resembles those observed for doubly *α*‐protonated forms discussed above. The final species could thus be tentatively identified as the fully protonated [**2b**‐H_4_]^4+^. Since its spectrum is red‐shifted relative to the spectrum of [**2b**‐H_3_]^3+^, it may be presumed that the final protonation leading to [**2b**‐H_4_]^4+^ may actually occur at the *meso*‐NMe_2_. Such a sequence would indicate that the latter group is less basic than the *α*‐NMe_2_ groups in **2b**. Lack of isosbestic points in the final protonation stage further suggests that the overall process may involve additional species, differentiated not only by their protonation level, but also by conformations and interactions with anions.

**Figure 5:**
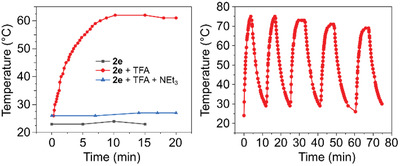
A) Photothermal response of **2e** and [**2e**‐H]^+^ to irradiation with a 1064 nm laser source (16 µM, toluene in air, 24 W cm^−2^). B) Temperature variation observed for [**2e**‐H]^+^ during 5 on–off irradiation cycles.

Importantly, the first protonation of **2d** and **2e** switches the principal absorption between the therapeutically relevant NIR‐I and NIR‐II ranges, providing an opportunity to use these compounds as pH‐sensitive photothermal agents (PTAs).^[^ ^60^, ^61^ ^]^ In a preliminary photothermal experiment (**Figure** **5**), a 16 µM solution of **2e** in toluene was irradiated with the same laser source (3 W, 24 W cm^−2^) for 15 min. Since the free base **2e** has a negligible absorbance at this wavelength, we observed only a residual photothermal effect of ≈1 °C. However, when the solution was treated with TFA to produce the dipyrrinium cation [**2e**‐H]^+^, the sample temperature increased significantly when the laser source was turned on, reaching 64 °C after ≈10 min of continuous irradiation (Figure 5A). The effect was reproducible in consecutive irradiation cycles (Figure 5B), with a minor decrease of the maximum temperature attributable to gradual evaporation of TFA caused by heating. Indeed, after addition of triethylamine to the solution, the free base **2e** was recovered, as evidenced by the change of the absorption spectrum, and the sample no longer showed any thermal response upon irradiation. The dipyrrinium cation [**2d**‐H]^+^ showed a similar switching behavior, producing comparable temperature changes upon irradiation (Figure S37, Supporting Information). Photothermal conversion efficiencies (PCE, *η*) estimated for [**2e**‐H]^+^ and [**2d**‐H]^+^ were relatively low (4–6%), but can likely be improved in an optimized experimental setup. These results demonstrate that the photothermal effect can be reversibly turned on and off in the NIR‐II range by changing the protonation status of the dipyrrin. encouraging their further exploration as NIR‐II organic PTAs.^[^ ^62^, ^63^, ^64^, ^65^, ^66^ ^]^

DFT calculations, performed for the neutral and protonated dipyrrins at the B3LYP‐GD3BJ/6‐31G(d,p) level of theory, correlate well with the experimental results, providing additional insight into the electronic structures of these dyes and their halochromism. For the dipyrrinium cations, the propeller‐like Z‐anti conformation was found to be the most stable one, in line with experimental findings (Table S12, Supporting Information). S_1_–S_0_ transition energies obtained from TD‐DFT calculations show a generally very good linear correlation with the electronic energy gaps estimated for dipyrrins and the dipyrrinium monocations (Table 2). The correlation is less accurate for halochromic Δ*E*_g_ values, although the experimental trend is reproduced by TD data.

**Figure 6:**
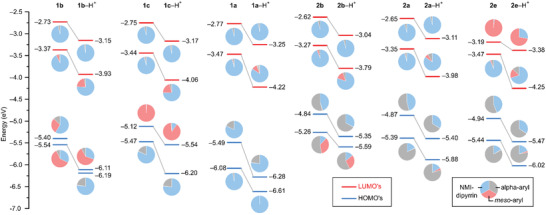
a) Fragmented pie charts representation of Kohn‐Sham MOs of selected dipyrrins calculated at B3LYP‐GD3BJ/6‐31G(d,p) level of theory with PCM(dichloromethane) solvation, b) MO energy level diagram of dipyrrins, neutral form (left side), and their first protonated form (right side).

A quantitative analysis of frontier molecular orbitals (MOs) was used to explore the influence of protonation, as well as *α*‐ and *meso*‐substitution, on the electronic structure of the dipyrrins (**Figure** **6** and Figures S38–S58, Supporting Information). In particular, contributions of key molecular fragments, namely 1) the NMI‐dipyrrin unit, 2) *α*‐substituents, and 3) meso substituents, were evaluated for frontier MOs. For **1a**, the highest occupied molecular orbital (HOMO, −5.49 eV) is localized mainly on the NMI‐dipyrrin unit and on the *α*‐phenyls (81% and 18% respectively), whereas the lowest unoccupied molecular orbital (LUMO, −3.47 eV) is almost exclusively confined to the NMI‐dipyrrin fragment. For both molecular orbitals (MOs), the contribution of the *meso*‐Ph group is negligible (≤1%), in line with the conformation‐induced decoupling of the latter substituent. In the dipyrrinium cation [**1a**‐H]^+^, the composition of the HOMO remains similar to that observed for the free base whereas an increased contribution of the meso substituent (10%) is observed for the LUMO. The latter effect is observed for all dipyrrins: the resultant lowering of LUMO energy caused by electron‐withdrawing *meso*‐substituents, explains the red shift they induce in dipyrrinium cations. While the HOMO levels are insignificantly affected by the C_6_F_5_ and 2,4‐dinitrophenyl *meso*‐substituents, dramatic changes are produced by the NMe_2_‐bearing groups. In **1b**, the meso contribution to the HOMO is 29%, whereas in **1c** the HOMO is localized almost exclusively on the meso substituent (99%). The HOMO–LUMO transition for the free base **1c** is however dipole‐forbidden according to TD calculations, and consequently, the associated CT band is not observed experimentally. For [**1c**‐H]^+^, the HOMO‐LUMO transition is predicted to have a high oscillator strength, thus validating the assignment of the 840 nm CT band. In the **2a**–**2e** series, contributions of *α*‐substituents to highest occupied MOs are much larger (>50%) than in the **1a**–**1e** series, resulting in significant elevation of HOMO energies and consequent reduction of the electronic gaps. Thus, *α*‐ and *meso*‐substitution in the NMI dipyrrins have complementary effects on the electronic structure of the chromophore.

**Figure 7:**
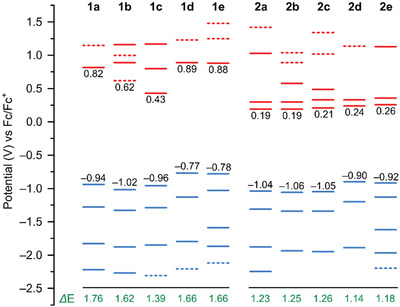
Redox potentials of dipyrrins **1a**–**1e** and **2a**–**2e** (differential pulse voltammetry, CH_2_Cl_2_, 0.1 M TBAPF_6_). red and blue bars indicate respectively oxidation and reduction couples. Dashed lines correspond to non‐reversible processes.

Electrochemical measurements revealed rich redox chemistry of the NMI dipyrrins, which showed up to five one‐electron oxidations and up to five one‐electron reductions in the −2.5 to 1.5 V range of potentials (versus Fc/Fc^+^, **Figure** **7** and Figures S30 and S31 and Table S10, Supporting Information). Remarkably, the majority of these redox events are electrochemically reversible, with each system possessing at least five stable oxidation states. These features result from the combined effect of NMI fusion, which is known to stabilize multiply reduced states,^[^ ^43^, ^46^, ^47^ ^]^ and the presence of electron‐rich *α*‐dimethylaminophenyl groups, which confer significant stability to the first two oxidized forms in the **2a**–**2e** series.

**Figure 8:**
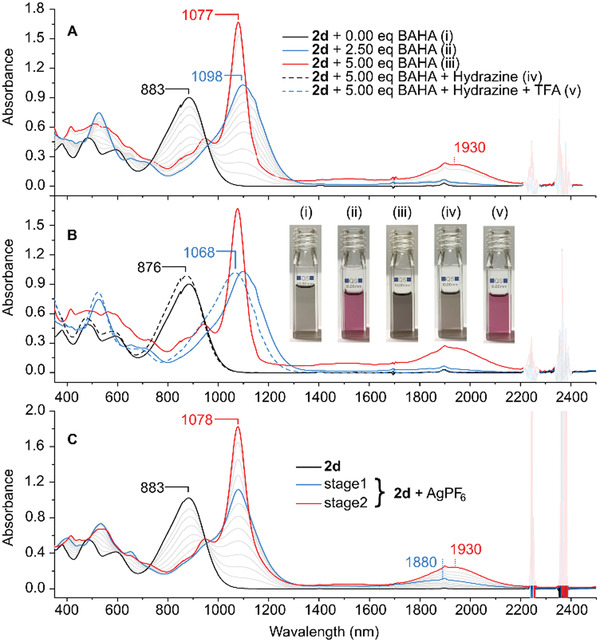
Spectral changes of 2d upon oxidation and reduction (CH_2_Cl_2_, 298 K). A) stepwise oxidation with BAHA, B) reduction with hydrazine followed by addition of acid, C) stepwise oxidation with AgPF_6_.

For instance, dipyrrin **2a** showed three reversible oxidations with (0.19, 0.30, and 1.03 V) suggesting that the radical cation and dication states might be accessible by chemical oxidation (Figure 7). **2a** and **2d** were accordingly treated with tris(4‐bromophenyl)ammoniumyl hexachloroantimonate (BAHA), and silver hexafluorophosphate (AgPF_6_),^[^ ^67^ ^]^ both of which were found to affect two‐electron oxidation of the dipyrrins (**Figure** **8** and Figure S28, Supporting Information). In particular, stepwise addition of 2.5 equiv of BAHA to **2d** resulted in the formation of a new absorption maximum at 1098 nm with a clear isosbestic point (Figure 8A). In comparison, **2a** showed the main maximum at 1000 nm accompanied by a much weaker broad band centered at ≈1850 nm. Upon further addition of BAHA (up to 5 equiv.) both **2d** and **2a** produced a new species, displaying two NIR bands (*λ*_max_ = 1077 and 1930 nm for **2d**). When these final oxidation products were reduced with excess hydrazine hydrate, the resulting spectra were blue‐shifted relative to the initial free base spectra (7–11 nm, Figure 8B and Figure S28, Supporting Information), suggesting a chemical modification of the starting dipyrrins. Indeed, for **2d**, the ultimate oxidation product, denoted **3d**, was subjected to a MALDI‐TOF analysis and found to contain an additional chloro substituent (Figure S152, Supporting Information). An XRD analysis of a disordered co‐crystal of **3d** and **2d** showed that the chlorination occurred at the 3 position of the *α*‐(4‐(dimethylamino)phenyl) substituents (total Cl occupancy of ≈0.4, Figure S13, Supporting Information). This type of reactivity, with the chlorine apparently originating from the SbCl_6_^−^ anion, has been previously documented for other BAHA oxidations.^[^ ^68^ ^]^

To avoid the chlorination, AgPF_6_ was used as an alternative oxidant for **2d**, yielding a somewhat different sequence of spectral changes (Figure 8C). Interestingly, while the final spectrum observed under these conditions was very similar to that obtained using BAHA, intact **2d** was recovered after reduction of the AgPF_6_‐oxidized mixture with either hydrazine hydrate or KO_2_. Oxidation of **2d** with 2.5 equiv of BAHA under ^1^H NMR control (CD_2_Cl_2_, 240 K, Figure S24, Supporting Information), showed that the primary species formed under these conditions is the diamagnetic dication [**2d**]^2+^, with a well‐defined spectrum, which was partly analyzed using correlation spectroscopy. In particular, it was found that the rotation of the *α*‐aryl substituents is hindered in [**2d**]^2+^ in comparison with the neutral **2d**, suggesting a more quinoidal character of the dication. The coexistence of the radical cation [**2d**]^•+^ was inferred from the presence of an additional broadened set of signals corresponding to a dipp substituents (other resonances could not be observed because of paramagnetic broadening) (Figure S25, Supporting Information). In comparison with **2a** and **2d**, the less electron‐rich **1a** appeared to undergo only partial oxidation to the radical cation [**1a**]^•+^ when treated with an excess of BAHA (Figure S27, Supporting Information). This behavior appears to be consistent with the high first oxidation potential of **1a** (0.82 V).

**Scheme 2:**
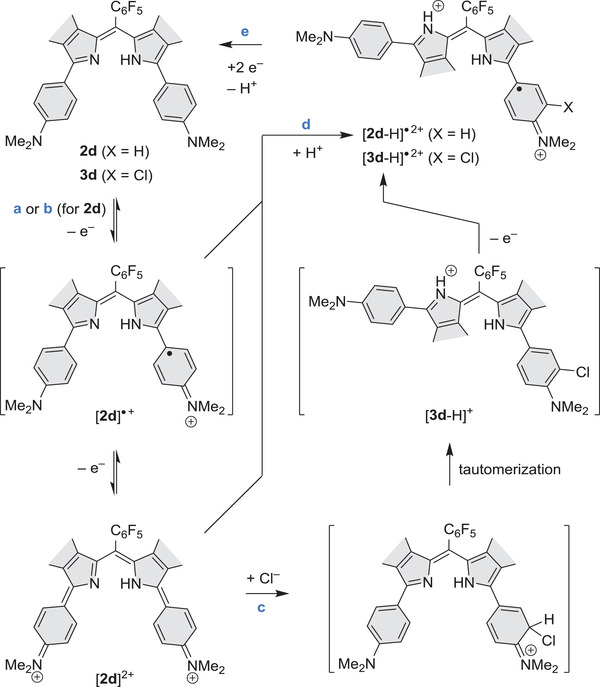
Protonation‐coupled redox reactivity of dipyrrin 2d. Reagents and conditions: a) 0 to 2.5 eq. BAHA, CH_2_Cl_2_; b) AgPF_6_, CH_2_Cl_2_; c) excess BAHA, CH_2_Cl_2_; b) excess AgPF_6_, CH_2_Cl_2_; e) hydrazine hydrate or KO_2_, CH_2_Cl_2_.

Combined experimental and TD‐DFT data can be used to rationalize the different effects of excess BAHA and AgPF_6_ oxidants on the equilibrium mixture of [**2d**]^•+^ and [**2d**]^2+^ (**Scheme** **2**, Figures S95–S102 and Tables S53–S60, Supporting Information). During BAHA oxidations, the dication reacts with chlorides released from the SbCl_6_^−^ anions to produce an arenium intermediate that tautomerizes to the chloro‐substituted dipyrrinium cation [**3d**‐H]^+^, which immediately undergoes one‐electron oxidation with excess BAHA, yielding the radical dication [**3d**‐H]^•2+^, which is stable under these conditions. The behavior observed with AgPF_6_ may be related to the acidity of the salt, which, when used in excess, can apparently lead to protonation of the radical cation [**2d**]^•+^. The resulting species, [**2d**‐H]^•2+^, is analogous to [**3d**‐H]^•2+^, and produces a very similar absorption spectrum, which can be accurately reproduced using TD‐DFT calculations. The possibility to reduce [**2d**‐H]^•2+^ back to the neutral dipyrrin **2d** shows that no substitution takes place when the dipyrrin is oxidized with AgPF_6_ (Figures S26 and S29, Supporting Information).

## 3 Conclusion

In conclusion, we have developed a family of acid‐ and redox‐responsive *π*‐extended dipyrrins containing fused naphthalimide subunits. The electronic properties of these dyes can be finely tuned using the complementary effects of *α* and meso substitution. Specifically, the energy gap is reduced by electron‐donating *α* groups, which increase the HOMO energy, and by electron‐withdrawing meso groups, which stabilize the LUMO level. LUMO stabilization is also responsible for the reduction of energy gaps in dipyrrinium monocations, in which a significant positive halochromic effect is produced via enhanced coupling with the *meso*‐substituent. The latter change is enhanced by a protonation‐induced conformational change, which imparts a TAM‐like character to the *π*‐system of the chromophore. As shown in our preliminary experiments, the unique combination of halochromism, photostability, and good photothermal efficiency of these dipyrrins makes them of interest as pH‐selective NIR‐II‐active agents for photothermal therapy.

## 4 Experimental Section

All experimental details are included in the Supporting Information.

[CCDC 2108054, 2110050, 2110061, 2110067, 2110070, and 2110072 contains the supplementary crystallographic data for this paper. These data can be obtained free of charge from The Cambridge Crystallographic Data Centre via www.ccdc.cam.ac.uk/data_request/cif.]

## Conflict of Interest

The authors declare no conflict of interest.

## Supporting information

Supporting Information

## Data Availability

The data that support the findings of this study are available in the supplementary material of this article.
