# Supplementary material for: Naphthalimide‐Fused Dipyrrins: Tunable Halochromic Switches and Photothermal NIR‐II Dyes
Source: Adv Sci (Weinh). 2022 Feb 17;9(19):2105886. doi: 10.1002/advs.202105886 (PMC9259717; doi:10.1002/advs.202105886)
Supplement: Supplementary file 1 — Supporting Information [file ADVS-9-2105886-s001.pdf]

ADVANCED SCIENCE Open Access

Supporting Information

for *Adv. Sci.*, DOI 10.1002/advs.202105886

Naphthalimide-Fused Dipyrrins: Tunable Halochromic Switches and Photothermal NIR-II Dyes

Yogesh Kumar Maurya, Piotr J. Chmielewski, Joanna Cybińska, Bibek Prajapati, Tadeusz Lis, Seongsoo Kang, Seokwon Lee, Dongho Kim and Marcin Stępień**

ADVANCED SCIENCE
Open Access

Supporting Information

for *Adv. Sci.*, DOI: 10.1002/advs.202105886

Naphthalimide-Fused Dipyrrins: Tunable Halochromic Switches and Photothermal NIR-II Dyes

Yogesh Kumar Maurya, Piotr J. Chmielewski, Joanna Cybińska, Bibek Prajapati, Tadeusz Lis, Seongsoo Kang, Seokwon Lee, Dongho Kim, and Marcin Stępień**

Table of Contents

Experimental 2

Synthesis 7

Additional Figures 21

Additional Tables 83

NMR Spectra 141

Mass Spectra 158

References 168

Experimental

General. Tetrahydrofuran was dried using a commercial solvent purification system. Dichloromethane was distilled from calcium hydride when used as reaction solvents. NBS was purified as described previously.[1]All other solvents and reagents were used as received. Compound **S1** was synthetized as previously described.[2] Microwave reactions were performed in a CEM Discover unit using external infrared temperature control system monitoring. ^{1}H NMR spectra were recorded on high-field spectrometers (^{1}H frequency 500.13 or 600.13 MHz), equipped with broadband inverse or conventional gradient probeheads. Spectra were referenced to the residual solvent signals (chloroform-*d*, 7.24 ppm). ^{13}C NMR spectra were recorded with ^{1}H broadband decoupling and referenced to solvent signals ($^{13}CDCl_3$, 77.0 ppm). Two-dimensional NMR spectra were recorded with 2048 data points in the t_2 domain and up to 2048 points in the t_1 domain, with a 1 s recovery delay. All 2D spectra were recorded with gradient selection. High resolution mass spectra were recorded using electrospray or MALDI ionization in the positive or negative mode on Bruker Daltonics micrOTOF-Q II™ – ESI-Q-TOF, Apex ultra FT-ICR 7T and Ultraflextreme MALDI TOF/TOF instruments. Absorption spectrometry was performed using Agilent Cary 60 UV-Vis and Cary 5000 UV-Vis-NIR spectrophotometers. Electrochemical measurements (DCM, 0.1 M $[NBu_4][PF_6]$, 293 K) were performed on an Autolab (Metrohm) potentiostat/galvanostat system using a glassy-carbon working electrode, platinum wire as the auxiliary electrode, and silver/silver chloride as a reference electrode. The voltammograms were referenced against the half-wave potential of ferrocenium/ferrocene couple.

Fluorescence Spectrometer from Edinburgh Instruments Ltd. A 450 W Xenon arc lamp (PL and PLE) was used as excitation sources. Emission spectra were corrected for the recording system efficiency and excitation spectra were corrected for the incident light intensity. PLE and PL spectra and QY were measured using a cooled extended-red Hamamatsu photomultiplier operating in a 200 – 1050 nm range. Quantum yield measurements were performed by using an Edinburgh Instruments integrating sphere equipped with a small elliptical mirror and a baffle plate for beam steering and shielding against directly detected light. For the measurement, the integrating sphere replaced the standard sample holder inside the sample chamber. Calculations of quantum yields were made using the software provided by Edinburgh Instruments.

A time-correlated single-photon-counting (TCSPC) system was used for measurements of spontaneous fluorescence decay. As an excitation light source, we used a mode-locked Ti:sapphire laser (Spectra Physics, MaiTai BB) which provides ultrashort pulse (center wavelength of 800 nm with 80 fs at FWHM) with high repetition rate (80 MHz). This high repetition rate was reduced to 800 kHz by using homemade pulse-picker. The pulse-picked output was frequency doubled by a 1-mm-thick BBO crystal (typeI, θ = 29.2°, EKSMA). The fluorescence was collected by a microchannel plate photomultiplier (MCPPMT, Hamamatsu, R3809U-51) with a thermoelectric cooler (Hamamatsu, C4878) connected to a TCSPC board (Becker & Hickel SPC-130). The overall instrumental response function was about 25 ps (FWHM). A vertically polarized pump pulse by a Glan-laser polarizer was irradiated to samples, and a sheet polarizer set at an angle complementary to the magic angle (54.7°), was placed in the fluorescence collection path to obtain polarization-independent fluorescence decays.

Femtosecond Transient Absorption Spectroscopy: A femtosecond time-resolved transient absorption (TA) spectrometer used for this study consisted of a femtosecond optical parametric amplifier (OPA, Quantronix, Palitra-FS) pumped by a Ti:sapphire regenerative amplifier system (Quantronix, Integra-C) operating at 1 kHz repetition rate and an accompanying optical detection system. The generated OPA pulses had a pulse width of ~100 fs and an average power of 1 mW in the range 550 to 690 nm, which

were used as pump pulses. White light continuum (WLC) probe pulses were generated using a sapphire window (2 mm thick) by focusing of small portion of the fundamental 800 nm pulses, which were picked off by a quartz plate before entering into the OPA. The time delay between pump and probe beams was carefully controlled by making the pump beam travel along a variable optical delay (Newport, ILS250). Intensities of the spectrally dispersed WLC probe pulses were monitored by high speed spectrometer (Ultrafast Systems). To obtain the timeresolved transient absorption difference signal (ΔA) at a specific time, the pump pulses were chopped at 500 Hz and absorption spectra intensities were saved alternately with or without pump pulse. Typically, 6000 pulses were used to excite samples and to obtain the TA spectra at a particular delay time. The polarization angle between pump and probe beam was set at the magic angle (54.7°) using a Glan-laser polarizer with a half-wave retarder to prevent polarization-dependent signals. The cross-correlation FWHM in the pump-probe experiments was less than 200 fs, and chirp of WLC probe pulses was measured to be 800 fs in the 450-850 nm regions. To minimize chirp, all reflection optics were used in the probe beam path, and a quartz cell of 2 mm path length was employed. After completing each set of fluorescence and TA experiments, the absorption spectra of all compounds were carefully checked to rule out the presence of artifacts or spurious signals arising from, for example, degradation or photooxidation of the samples in question

Chemical Oxidation of 2a and **2d**: The chemical oxidation experiments were performed in dichloromethane solution using various commercially available oxidants such as tris(4-bromophenyl)ammoniumyl hexachloroantimonate, and silver hexafluorophosphate. The stock or saturated solutions of oxidants were prepared in dichloromethane and was titrated in UV cuvette into the solution of desired dipyrrin (keeping the absorbance value around 1.0 ($A \approx 1.0$), $V = 2.5$ mL). The progress of the oxidation events was monitored spectrophotometrically upon gradually increasing the concetration of oxidant. The reduction of the oxidized species of dipyrrin was attempted using hydrazine hydrate or KO_2 solution dissolved in dichloromethane. The bulk oxidation experiment was monitored via NMR spectroscopy and the samples of dipyrrin (**2d**) and BAHA were prepared in deuterated dichloromethane solution. The desired 1D and 2D NMR spectra were recorded at 240 K for the oxidized and subsequent reduced form of dipyrrins. NMR measurments were carried out in deuterated chloroform when the oxidant was $AgPF_6$.

Protonation of Dipyrrins: The protonation forms of several dipyrrins were achieved from the stepwise titration using TFA as acid. To achieve the fully last stage protonation form of **1b** and **2b**, the stronger acid H_2SO_4 was used. The dipyrrins were titrated from the stock solutions of TFA (and H_2SO_4 whereever needed). The increasing concentrations of added TFA was used from the stock solutions of 6.47×10^{-3} M, 5.16×10^{-2} M, 1.03×10^{-1} M, 2.04×10^{-1} M and neat TFA. The increasing concentrations of added H_2SO_4 was used from the stock solution of 4.36×10^{-1} M. The high concentrations of acid solution were made in order to avoid the solvent volume increase in the UV cuvette cells.

Photothermal experiments: Similar as in titration experiments, TFA acid was used to protonate the dipyrrin **2d** and **2e**. Triethylamine was used as a base to deprotonate **2d**–H^+ and **2e**–H^+. After checking the photostability test, a toluene solution (volume 4 mL) of **2d**–H^+ and **2e**–H^+ were irradiated with 1064 nm laser (3 W) at a power density of 25 W/cm^2. In the experiment, a pulsed fiber laser was used (SPI G4). The laser emitted at the wavelength of 1064 nm with the repetition of 33 kHz and a pulse duration of 250 ns. During the experiment, the laser power of 3 W was used. The collimated laser beam of the diameter around 4mm was directed at the cuvette with the solution and passed vertically through the

sample. On the top of the container, a temperature detector (thermocouple) was installed. During the experiment, solvents were stirred. The thermocouple was dipped into the UV cuvette solution to record the change in temperature over time. The photothermal conversion efficiency (PCE) was calculated according to the equation (1) [3]

$$\eta = \frac{hS(T_{max} - T_{surr}) - Q_{dis}}{I(1 - 10^{-A_{1064}})} \qquad \text{equation (1)}$$

where h is the heat transfer coefficient, S is the surface area of the container, T_{max} represents the maximum steady-state temperature, T_{Surr} is the ambient temperature of the environment, Q_{dis} represents the heat dissipation from the light absorbed by the solvent and the quartz sample cell, I is the incident laser power (3.2 W/cm^2), and A_{1064} is the absorbance of the sample at 1064 nm. For **2d**–H^+, T_{max} is 58 °C, T_{surr} is 25 °C, A_{1064} is 1.15 and for **2e**–H^+, T_{max} is 75 °C, T_{surr} is 24 °C, A_{1064} is 1.28. Q_{dis} was assumed nearly zero here as the change in temperature in case of pure toluene was found to be only 1 °C (in 7 minutes).

hS was calculated referring to the following equation (2)

$$\tau_s = \frac{m_D c_D}{hS} \qquad \text{equation (2)}$$

where m_D and C_D index the solution mass and heat capacity (1.7 J g-1) of pure toluene used as the solvent, respectively.

τ_s was calculated from a linear graph from equation (3)

$$t = -\tau_s ln \frac{(T_{rt} - T_{surr})}{T_{max} - T_{surr}} \qquad \text{equation (3)}$$

where T_{rt} denotes as the real-time temperature in the cooling period. For **2d**–H^+, τ_s is calculated to be 207.5 s and for **2e**–H^+, τ_s is calculated to be 206.5 s.

According to the obtained data and substituting the values in equation (1), the photothermal conversion efficiency of **2d**–H^+ and **2e**–H^+ was determined to be 4.1 % and 6.1 %, respectively.

Computational methods. Density functional theory (DFT) calculations were performed using Gaussian 16.[4] DFT geometry optimizations were carried out in unconstrained C1 symmetry, using extended tight binding (xTB)[5,6] or semiempirical models as starting geometries. DFT geometries were refined to meet standard convergence criteria, and the existence of a stationary point was verified by a normal mode frequency calculation. Geometry optimizations, frequency calculations, and thermochemistry calculations were performed using the hybrid functional B3LYP [7–9] combined with the 6-31G(d,p) basis set and the GD3BJ dispersion correction.[10] In the calculations of absorption spectra, up to 25-50 electronic transitions were calculated by means of time-dependent DFT (TD-DFT), using the above level of theory, PCM solvation (standard dichloromethane parameters).[11] Open-shell singlets were optimized using the broken-symmetry formalism, with mixed initial guesses. The dication of dipyrrins were optimized using the 6-31G(d,p) basis set and the PCM solvent model using standard dichloromethane parameterization. The percent (%) contribution of fragments (NMI-dipyrrin, alpha-aryl and *meso*-aryl) to each molecular orbital (PDOS) were obtained using GaussSum version 3.0. In structure **1f'**, the mesityl groups were replaced with methyl.

X-ray crystallography. X-ray quality crystals for dipyrrins, **1a** were grown using chloroform/methanol/*n*-hexane solution, **1b** were grown using chloroform/*n*-hexane solution, **1e** were grown using chloroform/n-hexane solution, **2e** were grown in $CDCl_3$, **3d** were obtained in $CDCl_3$. X-ray crystals of protonated dipyrrins, **1a**–H^+ were obtained in $CDCl_3$ solution using HBr as acid source, **1b**–H^+ were obtained in $CDCl_3$ solution using HCl as acid source. Diffraction measurements were performed on a κ geometry XCALIBUR diffractometer (ω scans), equipped with an ONYX CCD camera, with graphitemonochromatized Cu Kα radiation. The data were collected at 110 K, corrected for Lorenz and polarization effects. Data collection, cell refinement, data reduction and analysis were carried out with the Xcalibur PX software, CRYSALIS CCD and CRYSALIS RED, respectively (Oxford Diffraction Ltd., Abignon, England, 2009). An analytical absorption correction was applied with the use of CRYSALIS RED. All structures were solved by direct methods with the SHELXS-97 program and refined using SHELXL-97 with anisotropic thermal parameters for non-H atoms. In the final refinement cycles, all H atoms were treated as riding atoms in geometrically optimized positions. CCDC **2108054**, **2110050**, **2110061**, **2110067**, **2110070** and **2110072** contains the supplementary crystallographic data for this paper. These data can be obtained free of charge from the Cambridge Crystallographic Data Centre via www.ccdc.cam.ac.uk/data_request/cif.

Statistical analysis. The majority of work presented herein did not require statystical analyses. Statistical treatment of X-ray diffraction data was performed using standard approaches.

Synthesis

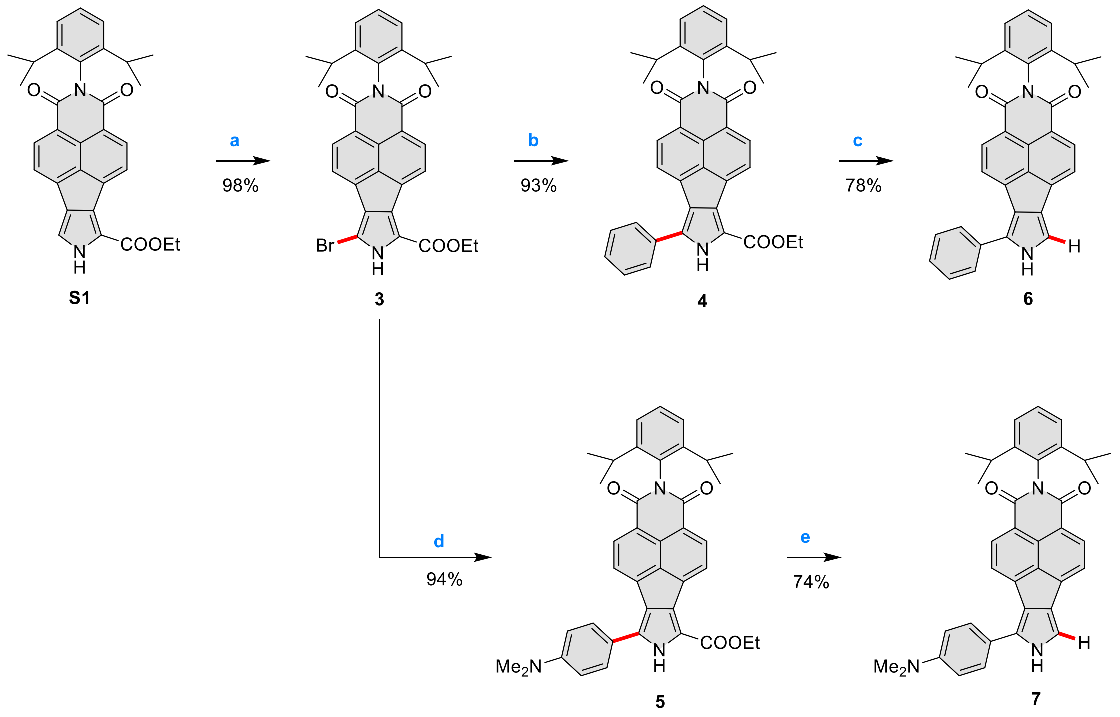

Scheme S1. *Synthesis of monopyrroles.* Reagents and conditions: (a) NBS, CH_2Cl_2, RT; (b) pressure tube, $PhB(OH)_2$, K_2CO_3, $Pd(dba)_2$, XPhos, THF/water (1/0.1, v/v), 100 °C; (c) KOH, $(CH_2OH)_2$, MW (100W, 190 °C, PowerMax); (d) pressure tube, *p*-$C_6H_4N(CH_3)_2$Bpin, K_2CO_3, $Pd(dba)_2$, XPhos, THF/water (1/0.1, v/v), 100 °C, (e) KOH, $(CH_2OH)_2$, MW (100W, 190 °C, PowerMax); (f) pressure tube, *p*-$C_6H_4NO_2$Bpin, K_2CO_3, $Pd(dba)_2$, THF/water (1/0.1, v/v), 100 °C, (g) KOH, $(CH_2OH)_2$, MW (100W, 190 °C, PowerMax).

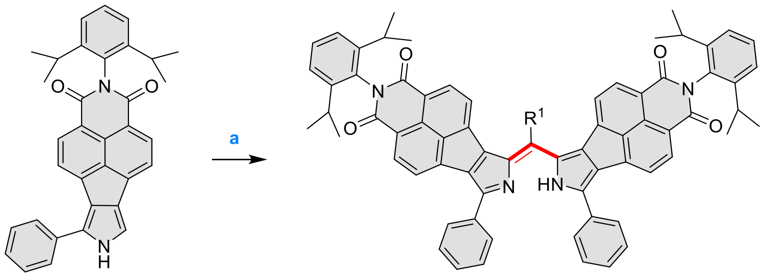

6

1a (79%, R^1 = phenyl)
1b (70%, R^1 = 4-dimethylaminophenyl)
1c (58%, R^1 = 4-dimethylaminobiphenyl)
1d (65%, R^1 = pentafluorophenyl)
1e (63%, R^1 = 2,4-dinitrophenyl)
1f (71%, R^1 = 4-(4,5-bis(mesityloxy)anthracen-9-yl)phenyl)

Scheme S2. Synthesis of dipyrrins **1a–1e**. Reagents and conditions: (a) R^{1}CHO, CF_3COOH, CH_2Cl_2, RT, 2 days; (ii) DDQ, RT, 30 min–4 h.

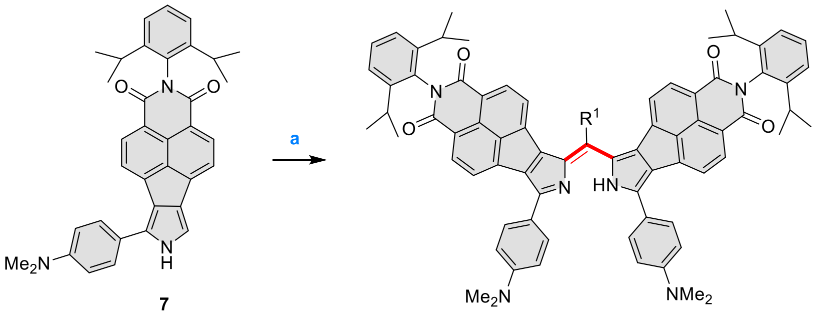

7

2a (70%, R^1 = phenyl)
2b (69%, R^1 = 4-dimethylaminophenyl)
2c (66%, R^1 = 4-dimethylaminobiphenyl)
2d (75%, R^1 = pentafluorophenyl)
2e (68%, R^1 = 2,4-dinitrophenyl)

Scheme S3. Synthesis of dipyrrins **2a–2e**. Reagents and conditions: (a) R^{1}CHO, CF_3COOH, $CHCl_3$, RT, 4 days; (ii) DDQ, RT, 20 min.

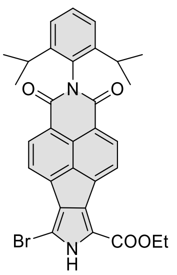

Ethyl 8-bromo-2-(2,6-diisopropylphenyl)-1,3-dioxo-1,2,3,7-etrahydropyrrolo[3',4':2,3]indeno[6,7,1-def] isoquinoline-6-carboxylate (3). Compound **S1** (0.40 g, 0.81 mmol) was dissolved in dichloromethane and freshly crystallized NBS (0.15 g, 0.84 mmol) was added to the reaction flask and the reaction mixture was stirred at room temperature for overnight. The reaction mixture was diluted with water and extracted with dichloromethane. The combined organic layers were dried over anhydrous sodium sulfate, filtered and evaporated under reduced pressure. The crude was purified by column chromatography on alumina (Grade V) using 50-60% dichloromethane in n-hexane. The solvent was evaporated on vaccuo to give yellow color solid powder as the desired product (0.45 g, 98%). **^{1}H NMR** (500 MHz, chloroform-*d*, 300 K): δ 9.22 (s, 1H), 8.53 (d, *J* = 7.4 Hz, 1H), 8.49 (d, *J* = 7.3 Hz, 1H), 8.14 (d, *J* = 7.3 Hz, 1H), 7.84 (d, *J* = 7.3 Hz, 1H), 7.44 (t, *J* = 7.8 Hz, 1H), 7.30 (d, *J* = 7.8 Hz, 2H), 4.51 (q, *J* = 7.1 Hz, 2H), 2.77 (sept, *J* = 6.8 Hz, 2H), 1.53 (t, *J* = 7.1 Hz, 3H), 1.14 (d, *J* = 6.8, 12H). **^{13}C NMR** (125 MHz, chloroform-*d*, 300 K): δ 163.97, 163.95, 159.67, 145.86, 137.15, 136.84, 135.93, 132.88, 132.81, 131.13, 129.65, 129.41, 126.38, 123.95, 123.60, 121.81, 120.94, 119.95, 119.78, 100.93, 77.28, 77.03, 76.77, 61.78, 29.09, 24.03, 14.65. **HRMS** (ESI–TOF): *m/z*: [M – H]$^+$ Calcd for $C_{31}H_{26}BrN_2O_4$: 569.1070; Found 569.1026. **UV-vis** (dichloromethane, 300 K) *λ* [nm] (*ε* in M^{-1} cm^{-1}): 303 (15300), 339 (5000), 356 (8500), 418 (21900), 438 (22800).

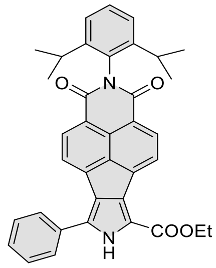

Ethyl 2-(2,6-diisopropylphenyl)-1,3-dioxo-8-phenyl-1,2,3,7-tetrahydropyrrolo[3',4':2,3]indeno[6,7,1-def]isoquinoline-6-carboxylate (4). A solution of compound **3** (100 mg, 0.175 mmol), phenylboronic acid (33.7 mg, 0.262 mmol), and K_2CO_3 (60.5 mg, 0.437 mmol), in aq-THF (water/ THF: 0.5/5 mL) was mixed in pressure tube and then filled with Argon. After $Pd(dba)_2$ (3 mg, 3 mol%) and XPhos (5 mg, 6 mol%) was added, the mixture was heated at 100 °C for 20 h. The reaction mixture was cooled to room temperature and quenched with water. The organic phase was washed with water and brine, dried over Na_2SO_4, and concentrated in vacuo. The crude was purified by column chromatography on alumina (Grade V) using 20-30% dichloromethane in n-hexane as eluent. The solvent was evaporated on vaccuo to give orange-red color powder as the desired product. (Yield: 93 mg, 93 %). **^{1}H NMR** (500 MHz, chloroform-*d*, 300 K): δ 9.06 (s, 1H), 8.53 (d, *J* = 7.3 Hz, 1H), 8.44 (d, *J* = 7.4 Hz, 1H), 8.18 (d, *J* = 7.3 Hz, 1H), 7.90 (d, *J* = 7.4 Hz, 1H), 7.81 (d, *J* = 7.2 Hz, 2H), 7.60 (t, *J* = 7.7 Hz, 2H), 7.50 (t, *J* = 7.5 Hz, 1H), 7.44 (t, *J* = 7.8 Hz, 1H), 7.30 (d, *J* = 7.8 Hz, 2H), 4.55 (q, *J* = 7.1 Hz, 2H), 2.79 (sept, *J* = 6.9 Hz, 2H), 1.56 (t, *J* = 7.1 Hz, 3H), 1.15 (d, *J* = 6.9 Hz, 12H). **^{13}C NMR** (151 MHz, chloroform-*d*, 300 K): δ 164.09, 164.00, 160.61, 145.89, 138.83, 137.70, 136.43, 133.82, 133.00,

132.84, 131.24, 130.61, 129.57, 129.51, 129.33, 126.50, 126.41, 126.34, 123.90, 123.40, 121.55, 120.61, 119.92, 118.49, 77.23, 77.02, 76.81, 61.56, 29.07, 24.02, 14.70. **HRMS** (ESI–TOF): *m/z*: $[M – H]^+$ Calcd for $C_{37}H_{31}N_2O_4$: 567.2278; Found 567.2237. **UV-vis** (dichloromethane, 300 K) λ [nm] (ε in M^{-1} cm^{-1}): 323 (27900), 358 (10400), 434 (20900), 459 (25400).

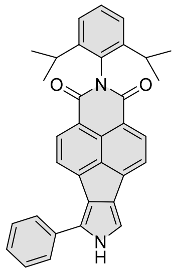

2-(2,6-diisopropylphenyl)-6-phenylpyrrolo[3',4':2,3]indeno[6,7,1-def]isoquinoline-1,3(2H,7H)-dione (6). The mixture of compound **4** (50 mg, 0.088 mmol) and potassium hydroxide, KOH (19.7 g, 0.532 mmol) was dissolved in ethylene glycol in microwave vial. The solution was purged with nitrogen for 10 min, and the reaction mixture was irradiated at 190 °C for 1.5 hour. The mixture was cooled to room temperature, poured into water and extracted with dichloromethane. The organic phase was washed with water and brine, dried over anhydrous sodium sulfate and evaporated under reduced pressure. The desired compound was obtained as orange-red powder (34 mg, 78 %). **^{1}H NMR** (600 MHz, chloroform-*d*, 300 K): δ 8.52 (s, 1H), 8.42 (d, *J* = 7.4 Hz, 2H), 7.84 (d, *J* = 7.4 Hz, 1H), 7.73 (d, *J* = 7.3 Hz, 2H), 7.66 (d, *J* = 7.3 Hz, 1H), 7.55 (t, *J* = 7.8 Hz, 2H), 7.43 (m, 2H), 7.30 (d, *J* = 7.8 Hz, 2H), 7.06 (d, *J* = 2.4 Hz, 1H), 2.79 (sept, *J* = 6.9 Hz, 2H), 1.15 (d, *J* = 6.8 Hz, 12H). **^{13}C NMR** (151 MHz, chloroform-*d*, 300 K): δ 164.36, 164.28, 145.94, 140.15, 139.67, 136.96, 133.11, 132.91, 131.61, 131.48, 130.42, 130.10, 129.41, 129.24, 128.45, 126.93, 125.69, 125.07, 123.87, 119.80, 119.74, 119.28, 118.84, 115.44, 77.23, 77.02, 76.81, 29.06, 24.03. **HRMS** (ESI–TOF): *m/z*: $[M – H]^+$ Calcd for $C_{34}H_{27}N_2O_2$: 495.2067; Found 495.2051. **UV-vis** (dichloromethane, 300 K) λ [nm] (ε in M^{-1} cm^{-1}): 313 (11700), 339 (5700), 356 (7400), 459 (23800), 483 (25400).

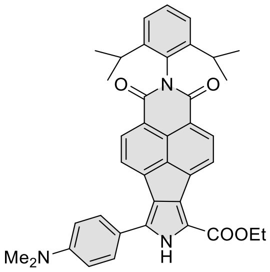

Ethyl 2-(2,6-diisopropylphenyl)-8-(4-(dimethylamino)phenyl)-1,3-dioxo-1,2,3,7-tetrahydropyrrolo[3',4':2,3]indeno[6,7,1-def] isoquinoline-6-carboxylate (5). A solution of compound **3 (**74 mg, 0.13 mmol), 4-(dimethylamino)phenylboronic acid pinacol ester (49.5 mg, 0.19 mmol), and K_2CO_3 (43.8 mg, 0.317 mmol), in aq-THF (water/ THF: 0.4/4 mL) was mixed in pressure tube and then filled with Argon. After $Pd(dba)_2$ (2.2 mg, 3 mol%) and XPhos (3.6 mg, 6 mol%) was added, the mixture was heated at 100 °C for 20 h. The reaction mixture was cooled to room temperature and quenched with water. The organic phase was washed with water and brine, dried over Na_2SO_4, and concentrated in vacuo. The crude was purified by column chromatography on alumina (Grade V) using 30-40% dichloromethane in n-hexane as eluent. The solvent was evaporated on vaccuo to give dark

blue-voilet color powder as the desired product (Yield: 74 mg, 94 %). **^{1}H NMR** (500 MHz, chloroform-*d*, 300 K): δ 8.85 (s, 1H), 8.50 (d, *J* = 7.4 Hz, 1H), 8.42 (d, *J* = 7.4 Hz, 1H), 8.15 (d, *J* = 7.4 Hz, 1H), 7.86 (d, *J* = 7.4 Hz, 1H), 7.68 (d, *J* = 8.9 Hz, 2H), 7.44 (t, *J* = 7.8 Hz, 1H), 7.30 (d, *J* = 7.8 Hz, 2H), 6.87 (d, *J* = 8.9 Hz, 2H), 4.53 (q, *J* = 7.1 Hz, 2H), 2.80 (sept, *J* = 6.9 Hz, 2H), 1.55 (t, *J* = 7.2 Hz, 3H, overlapping with water peak), 1.15 (d, *J* = 6.8 Hz, 12H). **^{13}C NMR** (151 MHz, chloroform-*d*, 300 K): δ 164.20, 164.12, 151.03, 145.91, 139.51, 137.95, 134.48, 132.97, 132.70, 129.27, 127.40, 123.86, 123.16, 121.33, 119.39, 117.79, 117.72, 112.41, 77.22, 77.01, 76.80, 61.34, 40.28, 29.05, 24.02, 14.73. **HRMS** (ESI–TOF): *m/z*: $[M-H]^+$ Calcd for $C_{39}H_{36}N_3O_4$: 610.2700; Found 610.2642. **UV-vis** (dichloromethane, 300 K) λ [nm] (ε in M^{-1} cm^{-1}): 364 (42300), 529 (17600).

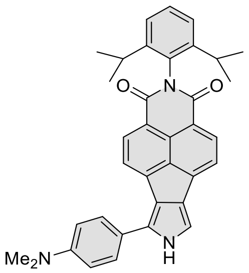

2-(2,6-diisopropylphenyl)-6-(4-(dimethylamino)phenyl)pyrrolo[3',4':2,3]indeno[6,7,1-def] isoquinoline-1,3(2H,7H)-dione (7). The mixture of compound **5** (40 mg, 0.066 mmol) and potassium hydroxide, KOH (14.5 mg, 0.262 mmol) was dissolved in ethylene glycol in microwave vial. The solution was purged with nitrogen for 10 min, and the reaction mixture was irradiated at 190 °C for 1.5 hour. The mixture was cooled to room temperature, poured into water and extracted with dichloromethane. The organic phase was washed with water and brine, dried over anhydrous sodium sulfate and evaporated under reduced pressure. The desired compound was obtained as orange powder (26 mg, 73 %). **^{1}H NMR** (600 MHz, chloroform-*d*, 300 K): δ 8.41 (d, *J* = 7.4 Hz, 1H), 8.38 (d, *J* = 7.5 Hz, 1H), 8.21 (s, 1H), 7.79 (d, *J* = 7.5 Hz, 1H), 7.62 (m, 3H), 7.43 (t, *J* = 7.8 Hz, 1H), 7.29 (d, *J* = 7.8 Hz, 2H), 7.05 (d, *J* = 2.4 Hz, 1H), 6.86 (d, *J* = 8.9 Hz, 2H), 2.80 (sept, *J* = 6.8 Hz, 2H), 1.14 (d, *J* = 6.9 Hz, 12H). **^{13}C NMR** (151 MHz, chloroform-*d*, 300 K): δ 164.40, 164.33, 150.46, 145.94, 140.65, 139.80, 137.06, 133.21, 132.73, 131.74, 131.61, 129.86, 129.14, 127.04, 126.77, 123.81, 123.22, 119.50, 119.17, 119.10, 118.94, 118.20, 114.73, 112.55, 77.22, 77.01, 76.80, 40.35, 24.02. **HRMS** (ESI–TOF): *m/z*: $[M-H]^+$ Calcd for $C_{36}H_{32}N_3O_2$: 538.2489; Found 538.2459. **UV-vis** (dichloromethane, 300 K) λ [nm] (ε in M^{-1} cm^{-1}): 336 (14000), 359 (11500), 536 (18300).

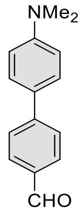

4'-(dimethylamino)-[1,1'-biphenyl]-4-carbaldehyde (S2). Compound **S2** was prepared by using modified procedure to that of reported compound.[11] A solution of 4-bromobenzaldehyde (50 mg, 0.268 mmol), 4-(dimethylamino)phenylboronic acid pinacol ester (67.5 mg, 0.268 mmol), and K_2CO_3 (73.9 mg, 0.54 mmol), in aq-THF (water/ THF: 0.2/2 mL) was mixed in pressure tube and then filled with Argon. After $PdCl_2(dppf)_2$ (2.2 mg, 3 mol%) was added, the mixture was heated for overnight at 100 °C. The reaction mixture was cooled to room temperature and quenched with water. The organic

phase was washed with water and brine, dried over Na_2SO_4, and concentrated in vacuo. The crude was recrystallized using *n*-hexane/dcm and the product was collected as brown-orange solid (Yield: 59 mg, 98 %) Spectrsoscopic data matches well with that of the reported compound.

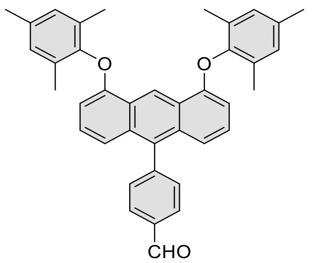

4-(4,5-bis(mesityloxy)anthracen-9-yl)benzaldehyde (S3). 10-bromo-1,8-bis(mesityloxy) anthracene[12] (50 mg, 0.095 mmol) and 4-formylphenylboronic acid (19.12 mg, 0.12 mmol) was dissolved in 4 mL of 5:2:1 of toluene/water/ethanol mixture and the solution was purged with nitrogen for 15 minutes. Potassium carbonate (52.60 mg, 0.38 mmol) was added to the mixture followed by the addition of tetrakis(triphenylphosphine)palladium(0) (5.57 mg, 0.005 mmol) The mixture was purged with nitrogen for few minutes and heated at 110 °C for overnight. After cooling to room temperature, water was added and extracted with dichloromethane. The organic layer was washed with brine, dried over anhydrous sodium sulfate and the solvent was removed on rotary evaporator. The crude mixture was purified via silica column chromatography using 30% dichloromethane in *n*-hexane as an eluent to give compound **S3** (47 mg, 89%) as a yellow solid. **^{1}H NMR** (500 MHz, chloroform-*d* , 300 K): δ 10.17 (1H, s), 9.92 (1H, s), 8.09 (2H, d, ^3J = 8.2 Hz), 7.65 (2H, d, ^3J = 8.2 Hz), 7.12 (4H, d, ^3J = 4.5 Hz), 6.95 (4H, s), 6.32 (2H, t, ^3J = 4.1 Hz), 2.33 (6H, s), 2.17 (12H, s). **^{13}C NMR** (125 MHz, chloroform-$d_{\#}$, 300 K): δ 192.05, 154.07, 149.10, 146.37, 135.58, 134.59, 132.09, 131.35, 130.95, 129.64, 126.12, 123.70, 118.96, 116.62, 104.12, 20.84, 16.12. **MS** (ESI–TOF): *m/z*: $[M + H]^+$ Calcd for $C_{39}H_{34}O_3$: 550.2502; Found 550.2607.

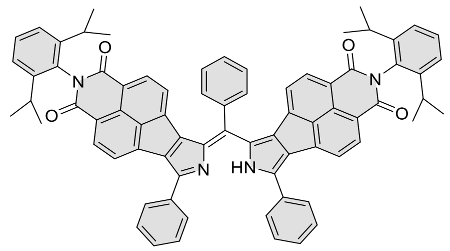

Dipyrrin (1a). A solution of compound **6** (7 mg, 0.14 mmol) and benzaldehyde (1.5 µL, 0.14 mmol, excess) in dichloromethane was purged with nitrogen for 10 min and trifluoroacetic acid (15 µL) was added to the solution and the resulting mixture was stirred for 2 days. Then 2,3- dichloro-5,6-dicyano-1,4-benzoquinone (DDQ, 2 mg) was added and the mixture was stirred for additional 4 h. The solvent was evaporated and the residue was passed through alumina (grade V) column chromatography with CH_2Cl_2/n-hexane (1/2, v/v to 2/1, v/v) as eluent. The green color fraction was collected and solvent was evaporated on rotary evaporator. Subsequently, the product was recrystallized from CH_2Cl_2/n-hexane to give a product as a black solid (6.4 mg, 79%). **^{1}H NMR** (600 MHz, chloroform-*d*, 300 K): δ 14.60 (s, 1H), 8.40 (d, *J* = 7.4 Hz, 2H), 8.12 (d, *J* = 7.8 Hz, 4H), 8.02 (d, *J* = 7.6 Hz, 2H), 7.97 (d, *J* = 7.4 Hz, 2H), 7.93 (d, *J* = 7.5 Hz, 1H), 7.85 (t, *J* = 7.6 Hz, 2H), 7.73 (d, *J* = 6.9 Hz, 2H), 7.69 (t, *J* = 7.6 Hz, 4H), 7.62 (t, *J* = 7.4

Hz, 2H), 7.44 (t, *J* = 7.9 Hz, 2H), 7.28 (d, *J* = 7.9 Hz, 4H), 4.94 (d, *J* = 7.5 Hz, 2H), 2.73 (sept, *J* = 6.8 Hz, 2H), 1.12 (d, *J* = 7.1 Hz, 24H). ^{13}**C NMR** (151 MHz, chloroform-*d*, 300 K): δ 163.77, 150.76, 146.66, 145.83, 139.91, 138.70, 138.19, 137.69, 137.55, 135.87, 134.65, 132.63, 132.36, 132.21, 131.77, 130.99, 130.51, 130.48, 129.43, 129.40, 129.13, 127.58, 125.79, 124.67, 123.91, 121.94, 121.69, 121.31, 77.23, 77.02, 76.81, 29.12, 23.99. **HRMS** (ESI–TOF): *m/z*: $[M + H]^+$ Calcd for $C_{75}H_{59}N_4O_4$: 1079.4536; Found 1079.4531. **UV-vis** (dichloromethane, 300 K) λ [nm] (ε in M^{-1} cm^{-1}): 376 (23 500), 396 (19 500), 484 (14 800), 519 (14 000), 668 (58 000), 723 (80 000).

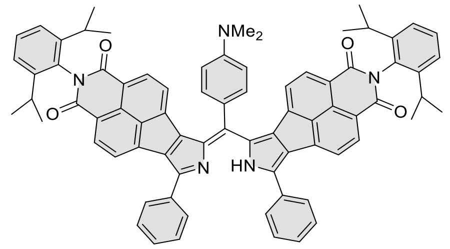

Dipyrrin (1b). A solution of compound **6** (30 mg, 0.061 mmol) and 4-(dimethylamino)benzaldehyde (4.5 mg, 0.031 mmol) in dichloromethane was purged with nitrogen for 10 min and trifluoroacetic acid (2 µL) was added to the solution and the resulting mixture was stirred for 2 days. Then 2,3- dichloro-5,6-dicyano-1,4-benzoquinone (DDQ, 8.3 mg, 0.037 mmol) was added and the mixture was stirred for additional 30 min. The solvent was evaporated and the residue was passed through alumina (grade V) column chromatography with CH_2Cl_2/n-hexane (1/2, v/v to 2/1, v/v) as the eluent. The green color fraction was collected and solvent was evaporated on rotary evaporator. Subsequently, the product was recrystallized from CH_2Cl_2/n-hexane to give a product as a black solid (24 mg, 70%). ^{1}**H NMR** (600 MHz, chloroform-*d*, 300 K): δ 13.70 (s, 1H), 8.40 (d, *J* = 7.4 Hz, 2H), 8.12 (d, *J* = 7.1 Hz, 4H), 8.07 (d, *J* = 7.5 Hz, 2H), 7.96 (d, *J* = 7.4 Hz, 2H), 7.67 (t, *J* = 7.6 Hz, 4H), 7.59 (t, *J* = 7.4 Hz, 2H), 7.49 (d, *J* = 8.7 Hz, 2H), 7.44 (t, *J* = 7.8 Hz, 2H), 7.29 (d, *J* = 7.8 Hz, 4H), 7.01 (d, *J* = 8.8 Hz, 2H), 5.43 (d, *J* = 7.5 Hz, 2H), 3.23 (s, 6H), 2.75 (sept, *J* = 6.8 Hz, 4H), 1.13 (d, *J* = 6.8, 24H). ^{13}**C NMR** (151 MHz, chloroform-*d*, 300 K): δ 164.03, 163.90, 152.81, 150.15, 146.61, 145.88, 142.48, 138.47, 138.09, 135.50, 134.87, 132.95, 132.45, 132.29, 131.10, 130.22, 129.38, 127.48, 125.89, 125.55, 125.01, 123.88, 121.67, 121.35, 121.06, 113.99, 77.22, 77.01, 76.80, 40.70, 29.08, 24.00, 23.97. **HRMS** (ESI–TOF): *m/z*: $[M + H]^+$ Calcd for $C_{77}H_{64}N_5O_4$: 1122.4958; Found 1122.5016. **UV-vis** (dichloromethane, 300 K) λ [nm] (ε in M^{-1} cm^{-1}): 377 (26 800), 395 (26 500), 486 (15 500), 523 (16 500), 668 (58 500), 728 (87 000).

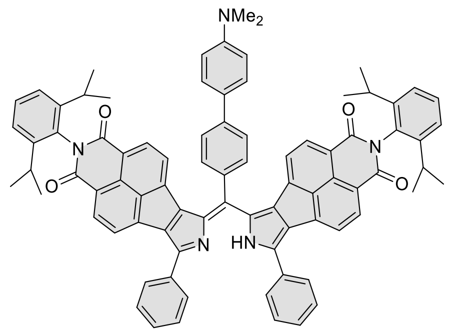

Dipyrrin (1c). A solution of compound **6** (14 mg, 0.027 mmol) and **S2** (3.2 mg, 0.013 mmol) in dichloromethane was purged with nitrogen for 10 min and trifluoroacetic acid (2 µL) was added to the solution and the resulting mixture was stirred for 2 days. Then 2,3- dichloro-5,6-dicyano-1,4-benzoquinone (3.7 mg, 0.016 mmol) was added and the mixture was stirred for additional 30 min. The

solvent was evaporated and the residue was passed through alumina (grade V) column chromatography with CH_2Cl_2/n-hexane (1/2, v/v to 2/1, v/v) as eluent. The green color fraction was collected and solvent was evaporated on rotary evaporator. Subsequently, the product was recrystallized from CH_2Cl_2/n-hexane to give a product as a black solid (9.5 mg, 58%). **^{1}H NMR** (600 MHz, chloroform-*d*, 300 K): δ 14.51 (s, 1H), 8.40 (d, *J* = 7.4 Hz, 2H), 8.14 (d, *J* = 7.1 Hz, 4H), 8.02 (d, *J* = 8.3 Hz, 2H), 7.98 (d, *J* = 7.6 Hz, 2H), 7.96 (d, *J* = 7.6 Hz, 2H), 7.74 (d, *J* = 8.7 Hz, 2H) 7.70 (d, *J* = 7.9 Hz, 6H), 7.62 (t, *J* = 7.4 Hz, 2H), 7.42 (t, *J* = 7.8 Hz, 2H), 7.27 (d, *J* = 7.9 Hz, 4H), 6.90 (d, *J* = 8.8 Hz, 2H), 5.25 (d, *J* = 7.5 Hz, 2H), 2.71 (sept, *J* = 6.9 Hz, 4H), 1.10 (d, *J* = 6.9 Hz, 24H). **^{13}C NMR** (151 MHz, chloroform-*d*, 300 K): δ 163.83, 163.77, 150.96, 150.65, 146.72, 145.85, 144.48, 140.59, 138.33, 137.83, 137.69, 135.81, 135.54, 134.72, 132.77, 132.35, 131.04, 130.44, 129.41, 129.36, 129.08, 127.94, 127.59, 126.93, 125.85, 125.02, 123.85, 121.89, 121.59, 121.26, 113.08, 77.23, 77.02, 76.80, 40.50, 31.93, 29.71, 29.08, 23.99, 23.96, 22.70, 14.12. **HRMS** (ESI–TOF): *m/z*: [M + H]$^+$ Calcd for $C_{83}H_{68}N_5O_4$: 1198.5271; Found 1198.5147. **UV-vis** (dichloromethane, 300 K) λ [nm] (ε in M^{-1} cm^{-1}): 376 (26 700), 396 (21 500), 483 (14 200), 521 (13 800), 665 (53 400), 725 (74 600).

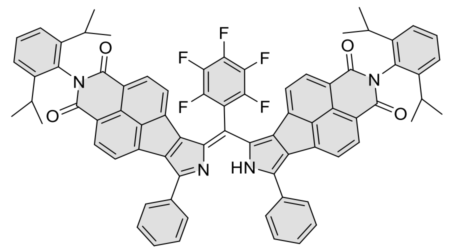

Dipyrrin (1d). A solution of compound **6** (7.5 mg, 0.015 mmol) and 2,3,4,5,6-pentafluorobenzaldehyde (1.5 µL, 0.015 mmol) in dichloromethane was purged with nitrogen for 10 min and trifluoroacetic acid (15 µL) was added to the solution and the resulting mixture was stirred for 2 days. Then 2,3- dichloro-5,6-dicyano-1,4-benzoquinone (DDQ, 2 mg) was added and the mixture was stirred for additional 4 h. The solvent was evaporated and the residue was passed through alumina (grade V) column chromatography with CH_2Cl_2/n-hexane (1/2, v/v to 2/1, v/v) as the eluent. The green color fraction was collected and solvent was evaporated on rotary evaporator. Subsequently, the product was recrystallized from CH_2Cl_2/*n*-hexane to give a product as a black solid (5.6 mg, 65%). **^{1}H NMR** (500 MHz, chloroform-*d*, 300 K): δ 14.75 (s, 1H), 8.46 (d, *J* = 7.4 Hz, 2H), 8.27 (d, *J* = 7.5 Hz, 2H), 8.12 (d, *J* = 7.0 Hz, 4H), 8.04 (d, *J* = 7.4 Hz, 2H), 7.70 (t, *J* = 7.3 Hz, 4H), 7.65 (t, *J* = 7.3 Hz, 2H), 7.46 (t, *J* = 7.8 Hz, 2H), 7.30 (d, *J* = 7.9 Hz, 4H), 5.45 (d, *J* = 7.5 Hz, 2H), 2.74 (sept, *J* = 6.7 Hz, 4H), 1.14 (d, *J* = 6.8, 24H). **^{13}C NMR** (125 MHz, chloroform-*d*, 300 K): δ 163.59, 163.50, 152.11, 145.84, 145.08, 137.82, 137.26, 137.18, 137.01, 134.68, 132.88, 132.23, 131.09, 130.78, 129.56, 127.79, 126.21, 123.98, 122.74, 122.62, 121.87, 121.83, 121.10, 77.27, 77.02, 76.76, 29.71, 29.14, 23.99, 23.97. **HRMS** (ESI–TOF): *m/z*: [M + H]$^+$ Calcd for $C_{75}H_{54}F_5N_4O_4$: 1169.4065; Found 1169.4045. **UV-vis** (dichloromethane, 300 K) λ [nm] (ε in M^{-1} cm^{-1}): 381 (25 100), 485 (17 400), 521 (14 700), 681 (60 200), 733 (76 400).

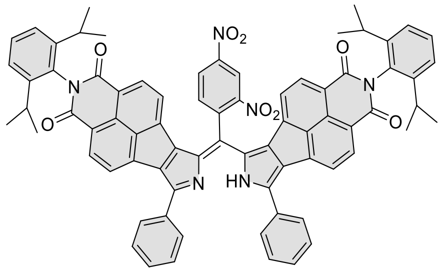

Dipyrrin (1e). A solution of compound **6** (7 mg, 0.014 mmol) and 2,4-dinitrobenzaldehyde (1.5 mg, 0.008 mmol) in dichloromethane was purged with nitrogen for 10 min and trifluoroacetic acid (15 µL) was added to the solution and the resulting mixture was stirred for 2 days. Then 2,3- dichloro-5,6-dicyano-1,4-benzoquinone (DDQ, 2 mg) was added and the mixture was stirred for additional 4 h. The solvent was evaporated and the residue was passed through alumina (grade V) column chromatography with CH_2Cl_2/n-hexane (1/2, v/v to 2/1, v/v) as eluent. The green color fraction was collected and solvent was evaporated on rotary evaporator. Subsequently, the product was recrystallized from CH_2Cl_2/n-hexane to give a product as a black solid (5.0 mg, 63%). **^{1}H NMR** (500 MHz, chloroform-*d*, 300 K): δ 14.79 (s, 1H), 9.38 (d, *J* = 2.3 Hz, 1H), 8.93 (dd, *J* = 8.3, 2.3 Hz, 1H), 8.44 (d, *J* = 7.4 Hz, 2H), 8.24 (d, *J* = 8.3 Hz, 1H), 8.12 (d, *J* = 7.0 Hz, 4H), 8.02 (dd, *J* = 7.5, 2.7 Hz, 4H), 7.71 (t, *J* = 7.3 Hz, 4H), 7.65 (t, *J* = 7.3 Hz, 2H), 7.44 (t, *J* = 7.8 Hz, 2H), 7.28 (d, *J* = 7.9 Hz, 4H), 4.90 (d, *J* = 7.5 Hz, 2H), 2.70 (m, 4H), 1.14 (m, 24H).. **^{13}C NMR** (151 MHz, chloroform-*d*, 300 K): δ 163.52, 163.40, 151.82, 149.82, 149.47,145.83, 145.77, 144.76, 139.03, 137.14, 137.03, 136.96, 136.72, 134.63, 132.86, 132.18, 131.91, 131.44, 131.06, 130.72, 129.74, 129.56, 127.77, 126.09, 124.00, 123.91, 122.58, 122.11, 121.77, 77.22, 77.01, 76.80, 29.70, 29.12, 23.98, 23.95, 23.92. **HRMS** (ESI–TOF): *m/z*: [M + H]$^+$ Calcd for $C_{75}H_{57}N_6O_8$: 1169.4238; Found 1169.4246. **UV-vis** (dichloromethane, 300 K) *λ* [nm] (*ε* in M^{-1} cm^{-1}): 379 (25 800), 483 (17 600), 521 (14 500), 682 (62 000), 734 (78 300).

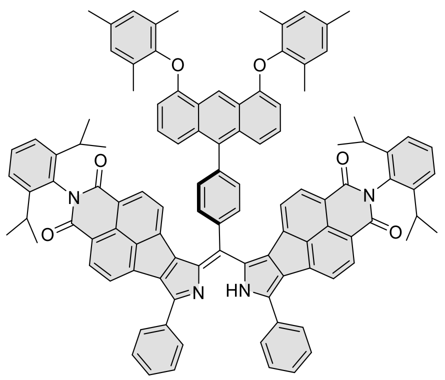

Dipyrrin (1f). A solution of compound **6** (13 mg, 0.026 mmol) and **S3** (7.9 mg, 0.014 mmol) in dichloromethane was purged with nitrogen for 10 min and trifluoroacetic acid (25 µL) was added to the solution and the resulting mixture was stirred for 2 days. Then 2,3- dichloro-5,6-dicyano-1,4-benzoquinone (DDQ, 4 mg) was added and the mixture was stirred for additional 4 h. The solvent was evaporated and the residue was passed through alumina (grade V) column chromatography with CH_2Cl_2/n-hexane (1/2, v/v to 2/1, v/v) as eluent. The green color fraction was collected and solvent was evaporated on rotary evaporator. Subsequently, the product was recrystallized from CH_2Cl_2/n-hexane to give a product as a black solid (14 mg, 71%). **^{1}H NMR** (500 MHz, chloroform-*d*, 300 K): δ δ 14.66 (s, 1H), 10.03 (s, 1H), 8.44 (d, *J* = 7.4 Hz, 2H), 8.38 (d, *J* = 7.4 Hz, 2H), 8.17 (d, *J* = 7.1 Hz, 4H), 8.06 (d, *J* = 8.1 Hz, 2H), 8.02 (m, 4H), 7.91 (d, *J* = 8.8 Hz, 2H), 7.71 (t, *J* = 7.5 Hz, 4H), 7.64 (t, *J* = 7.4 Hz, 2H), 7.42 (m, 4H), 7.28 (d, *J* = 7.9 Hz, 4H), 6.96 (s, 4H), 6.37 (d, *J* = 7.3 Hz, 2H), 5.85 (d, *J* = 7.4 Hz, 2H), 2.74 (m, 4H),

2.33 (s, 6H), 2.17 (s, 10H), 1.11 (m, 24H). **^{13}C NMR** (151 MHz, chloroform-*d*, 300 K): δ 163.77, 163.68, 154.52, 150.92, 149.12, 146.62, 145.79, 142.32, 140.33, 138.56, 138.01, 137.67, 137.42, 136.16, 135.11, 134.83, 134.64, 133.70, 132.68, 132.57, 132.48, 131.47, 130.98, 130.85, 130.57, 129.80, 129.73, 129.46, 127.66, 127.19, 125.96, 124.67, 124.00, 123.94, 122.29, 121.87, 121.44, 118.59, 117.33, 104.79, 29.11, 24.00, 23.97, 20.86, 16.19. **HRMS** (ESI–TOF): *m/z*: $[M + H]^+$ Calcd for $C_{107}H_{87}N_4O_6$: 1523.6620; Found 1523.6212. **UV-vis** (dichloromethane, 300 K) λ [nm] (ε in M^{-1} cm^{-1}): 378 (34 100), 393 (33 500), 484 (14 000), 521 (12 800), 673 (51 400), 726 (70 600).

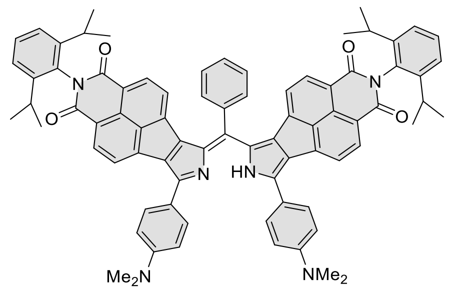

Dipyrrin (2a). A solution of compound **7** (7.5 mg, 0.0138 mmol) and benzaldehyde (1.0 µL, 0.010 mmol) in chloroform was purged with nitrogen for 10 min and trifluoroacetic acid (6 µL) was added to the solution and the resulting mixture was stirred for 4 days. Then 2,3- dichloro-5,6-dicyano-1,4-benzoquinone (DDQ, 1.9 mg, 0.008 mmol) was added and the mixture was stirred for additional 20 min. The solvent was evaporated and the residue was passed through alumina (grade V) column chromatography with CH_2Cl_2/n-hexane (1/2, v/v to pure CH_2Cl_2) as the eluent. The red-black fraction was collected and solvent was evaporated on rotary evaporator. Subsequently, the product was recrystallized from CH_2Cl_2/n-hexane to give a product as a grey-black solid (5.6 mg, 70%). **^{1}H NMR** (500 MHz, chloroform-*d*, 300 K): δ 14.62 (s, 1H), 8.37 (d, *J* = 7.4 Hz, 2H), 8.05 (d, *J* = 8.8 Hz, 4H), 7.97 (d, *J* = 7.5 Hz, 4H), 7.89 (t, *J* = 7.4 Hz, 1H), 7.80 (t, *J* = 7.7 Hz, 2H), 7.70 (d, *J* = 7.0 Hz, 2H), 7.43 (t, *J* = 7.9 Hz, 2H), 7.28 (d, *J* = 7.9 Hz, 4H), 6.96 (d, *J* = 8.8 Hz, 4H), 4.88 (d, *J* = 7.6 Hz, 2H), 3.16 (s, 12H), 2.73 (m, 4H), 1.12 (d, *J* = 7.0 Hz, 24H). **^{13}C NMR** (151 MHz, chloroform-*d*, 300 K): δ 163.97, 163.92, 151.66, 150.90, 146.05, 145.88, 139.26, 138.46, 138.37, 138.12, 136.58, 135.17, 134.73, 132.40, 132.16, 131.56, 131.15, 130.10, 129.52, 129.33, 129.15, 125.83,124.28, 123.85, 121.45, 121.28, 120.68, 120.12, 112.18, 77.22, 77.01, 76.79, 40.31, 29.70, 29.32, 29.06, 27.22, 23.97,22.69, 14.11. **HRMS** (ESI–TOF): *m/z*: $[M + H]^+$ Calcd for $C_{79}H_{68}N_6O_4$: 1165.5375; Found 1165.5447. **UV-vis** (dichloromethane, 300 K) λ [nm] (ε in M^{-1} cm^{-1}): 376 (34 800), 464 (33 500), 580 (26 300), 843 (70 000).

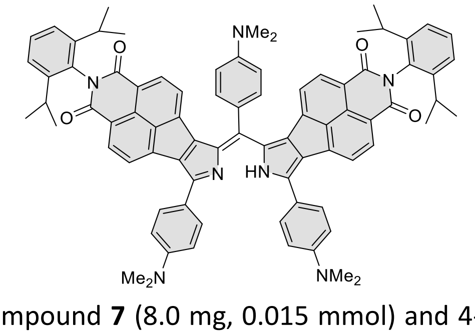

Dipyrrin (2b). A solution of compound **7** (8.0 mg, 0.015 mmol) and 4-(dimethylamino)benzaldehyde (1.1 mg, 0.007 mmol) in chloroform was purged with nitrogen for 10 min and trifluoroacetic acid (6 µL)

was added to the solution and the resulting mixture was stirred for 4 days. Then 2,3- dichloro-5,6-dicyano-1,4-benzoquinone (DDQ, 2.0 mg, 0.009 mmol) was added and the mixture was stirred for additional 20 min. The solvent was evaporated and the residue was passed through alumina (grade V) column chromatography with CH_2Cl_2/n-hexane (1/2, v/v to pure CH_2Cl_2) as the eluent. The red-black fraction was collected and solvent was evaporated on rotary evaporator. Subsequently, the product was recrystallized from CH_2Cl_2/n-hexane to give a product as a grey-black solid (6.2 mg, 70%). **^{1}H NMR** (500 MHz, chloroform-*d*, 300 K): δ 13.98 (s, 1H), 8.36 (d, *J* = 7.4 Hz, 2H), 8.03 (d, *J* = 7.7 Hz, 6H), 7.92 (d, *J* = 7.5 Hz, 2H), 7.44 (dd, *J* = 15.7, 8.2 Hz, 4H), 7.29 (d, *J* = 7.9 Hz, 4H), 6.99 (d, *J* = 8.8 Hz, 2H), 6.94 (d, *J* = 8.9 Hz, 4H), 5.33 (d, *J* = 7.6 Hz, 2H), 3.20 (s, 6H), 3.15 (s, 12H), 2.76 (sept, *J* = 6.8 Hz, 4H), 1.13 (t, *J* = 6.5 Hz, 24H). **^{13}C NMR** (151 MHz, chloroform-*d*, 300 K): δ 164.16, 164.06, 151.51, 150.45, 146.15, 145.92, 138.82, 138.76, 138.40, 134.88,134.76, 132.44, 132.25, 131.25, 130.93, 129.29, 128.99, 125.90, 124.73, 123.83, 120.95, 120.43, 120.37, 114.05, 112.20, 77.22, 77.01, 76.80, 40.73, 40.32, 29.70, 29.06. **HRMS** (ESI–TOF): *m/z*: $[M + H]^+$ Calcd for $C_{81}H_{73}N_7O_4$: 1208.5797; Found 1208.5792. **UV-vis** (dichloromethane, 300 K) λ [nm] (ε in M^{-1} cm^{-1}): 380 (45 200), 392 (44 400), 458 (42 800), 582 (32 000), 839 (81 000).

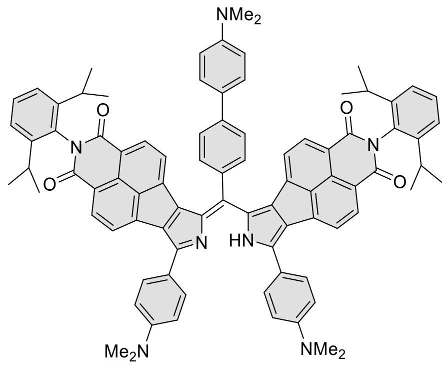

Dipyrrin (2c). A solution of compound **7** (17 mg, 0.03 mmol) and **S2** (3.5 mg, 0.015 mmol) in chloroform was purged with nitrogen for 10 min and trifluoroacetic acid (8 µL) was added to the solution and the resulting mixture was stirred for 4 days. Then 2,3- dichloro-5,6-dicyano-1,4-benzoquinone (DDQ, 4.3 mg, 0.019 mmol) was added and the mixture was stirred for additional 30 min. The solvent was evaporated and the residue was passed through alumina (grade V) column chromatography with CH_2Cl_2/n-hexane (1/2, v/v to pure CH_2Cl_2) as the eluent. The red-black fraction was collected and solvent was evaporated on rotary evaporator. Subsequently, the product was recrystallized from CH_2Cl_2/n-hexane to give a product as a grey-black solid (13.2 mg, 66%). **^{1}H NMR** (500 MHz, chloroform-*d*, 300 K): δ 14.63 (s, 1H), 8.31 (d, *J* = 7.4 Hz, 2H), 7.97 (d, *J* = 8.8 Hz, 4H), 7.92 (d, *J* = 8.2 Hz, 2H), 7.86 (d, *J* = 7.6 Hz, 2H), 7.81 (d, *J* = 7.5 Hz, 2H), 7.69 (d, *J* = 7.3 Hz, 4H), 7.42 (t, *J* = 7.9 Hz, 2H), 7.27 (d, *J* = 7.9 Hz, 4H), 6.92 (d, *J* = 8.9 Hz, 4H), 6.89 (d, *J* = 8.8 Hz, 2H), 5.11 (d, *J* = 7.6 Hz, 2H), 3.15 (s, 12H), 3.05 (s, 6H), 2.74 (sept, *J* = 6.8 Hz, 4H), 1.12 (d, *J* = 6.9 Hz, 24H). The compound produced broadened ^{13}C NMR spectrum, which were not analytically useful. **HRMS** (ESI–TOF): *m/z*: $[M + H]^+$ Calcd for $C_{87}H_{78}N_7O_4$: 1284.6110; Found 1284.6245. **UV-vis** (dichloromethane, 300 K) λ [nm] (ε in M^{-1} cm^{-1}): 376 (43 900), 463 (36 500), 580 (28 600), 846 (68 800).

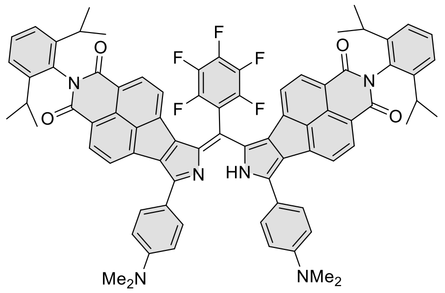

Dipyrrin (2d). A solution of compound **7** (18 mg, 0.033 mmol) and 2,3,4,5,6-pentafluorobenzaldehyde (3.2 µL, 0.017 mmol) in chloroform was purged with nitrogen for 10 min and trifluoroacetic acid (5 µL) was added to the solution and the resulting mixture was stirred for 4 days. Then 2,3- dichloro-5,6-dicyano-1,4-benzoquinone (DDQ, 6.5 mg, 0.029 mmol) was added and the mixture was stirred for additional 20 min. The solvent was evaporated and the residue was passed through alumina (grade V) column chromatography with CH_2Cl_2/n-hexane (1/2, v/v to pure CH_2Cl_2) as the eluent. The red-black fraction was collected and solvent was evaporated on rotary evaporator. Subsequently, the product was recrystallized from CH_2Cl_2/n-hexane to give a product as a grey-black solid (15.5 mg, 75%). **^{1}H NMR** (500 MHz, chloroform-*d*, 300 K): δ 14.84 (s, 1H), 8.43 (d, *J* = 7.4 Hz, 2H), 8.22 (d, *J* = 7.5 Hz, 2H), 8.03 (m, 6H), 7.45 (t, *J* = 7.9 Hz, 2H), 7.30 (d, *J* = 7.9 Hz, 4H), 6.95 (d, *J* = 9.0 Hz, 4H), 5.38 (d, *J* = 7.5 Hz, 2H), 3.18 (s, 11H), 2.75 (sept, *J* = 13.6, 6.8 Hz, 4H), 1.13 (d, *J* = 6.8, 23H). **^{13}C NMR** (151 MHz, chloroform-*d*, 300 K): δ 163.76, 163.66, 151.92, 151.83, 145.88, 144.30, 138.18, 138.00, 137.69, 136.24, 134.75, 132.85, 132.14, 130.95, 129.60, 129.46, 126.21, 123.93, 122.15, 122.12, 121.38, 121.16, 119.54, 112.52, 112.14, 40.24, 29.12, 24.00, 23.97. **HRMS** (ESI–TOF): *m/z*: [M + H]$^+$ Calcd for $C_{79}H_{64}F_5N_6O_4$: 1255.4909; Found 1255.4902. **UV-vis** (dichloromethane, 300 K) *λ* [nm] (*ε* in M^{-1} cm^{-1}): 377 (37 800), 474 (38 400), 582 (30 300), 886 (66 000).

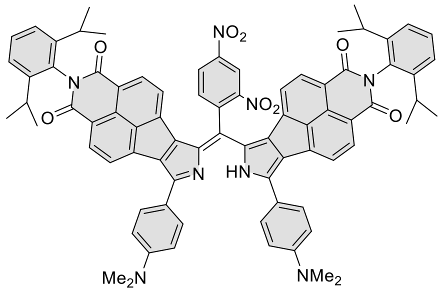

Dipyrrin (2e). A solution of compound **7** (26 mg, 0.048 mmol) and 2,4-dinitrobenzaldehyde (4.7 mg, 0.023 mmol) in chloroform was purged with nitrogen for 10 min and trifluoroacetic acid (10 µL) was added to the solution and the resulting mixture was stirred for 4 days. Then 2,3- dichloro-5,6-dicyano-1,4-benzoquinone (DDQ, 6.4 mg, 0.028 mmol) was added and the mixture was stirred for additional 20 min. The solvent was evaporated and the residue was passed through alumina (grade V) column chromatography with CH_2Cl_2/n-hexane (1/2, v/v to pure CH_2Cl_2) as the eluent. The red-black fraction was collected and solvent was evaporated on rotary evaporator. Subsequently, the product was recrystallized from CH_2Cl_2/n-hexane to give a product as a grey-black solid (20 mg, 68%). **^{1}H NMR** (500 MHz, chloroform-*d*, 300 K): δ 14.96 (s, 1H), 9.22 (s, 1H), 8.61 (broad, 1H), 8.35 (broad, 1H), 8.25 (broad, 2H), 7.71 (broad, 8H), 7.45 (m, 2H), 7.30 (m, 4H), 6.89 (d, *J* = 8.7 Hz, 4H), 4.65 (broad, 2H), 3.18 (s, 12H), 2.82 (broad, 2H), 2.71 (m, 2H), 1.21 (m, 12H), 1.11 (m, 12H). The compound produced broadened ^{13}C NMR spectrum, which were not analytically useful. **HRMS** (ESI–TOF): *m/z*: [M + H]$^+$ Calcd for

$C_{79}H_{67}N_8O_8$: 1255.5076; Found 1255.5578. **UV-vis** (dichloromethane, 300 K) λ [nm] (ε in M^{-1} cm^{-1}): 381 (35 800), 485 (40 200), 595 (30 200), 878 (73 900).

Additional Figures

(a)

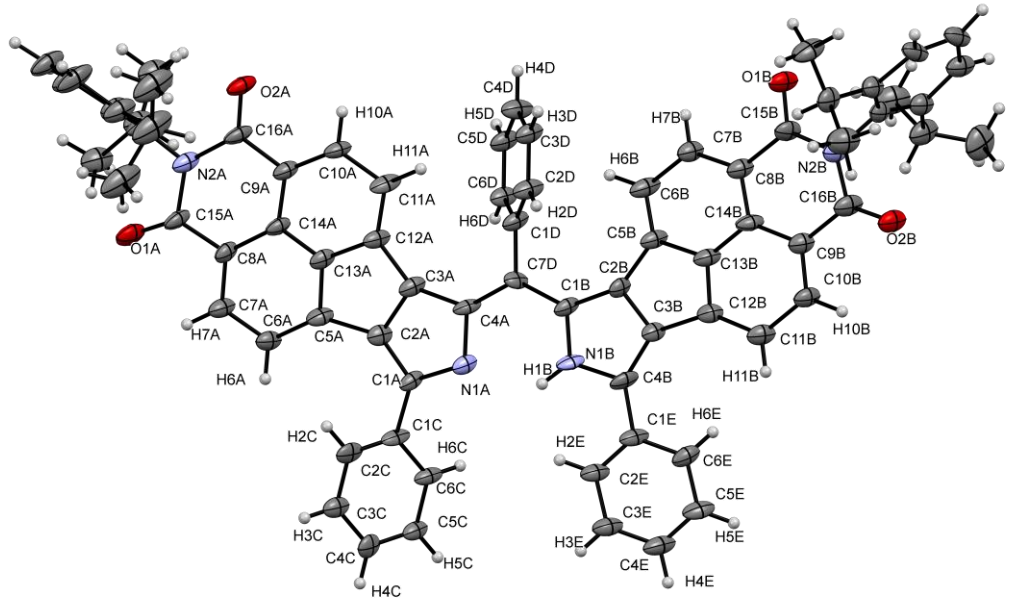

(b)

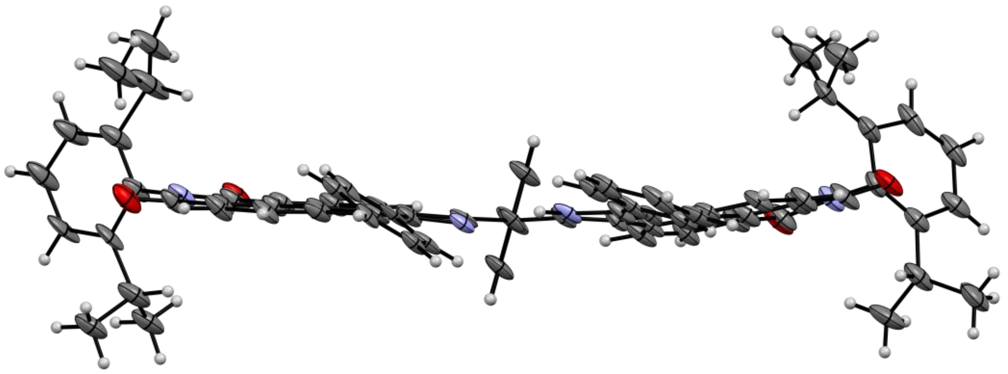

Figure S1. X-ray crystal structure (a) top view and (b) side view of **1a**. For clarity, all solvent molecules are omitted (ellipoids are scaled to 10% probability level).

(a)

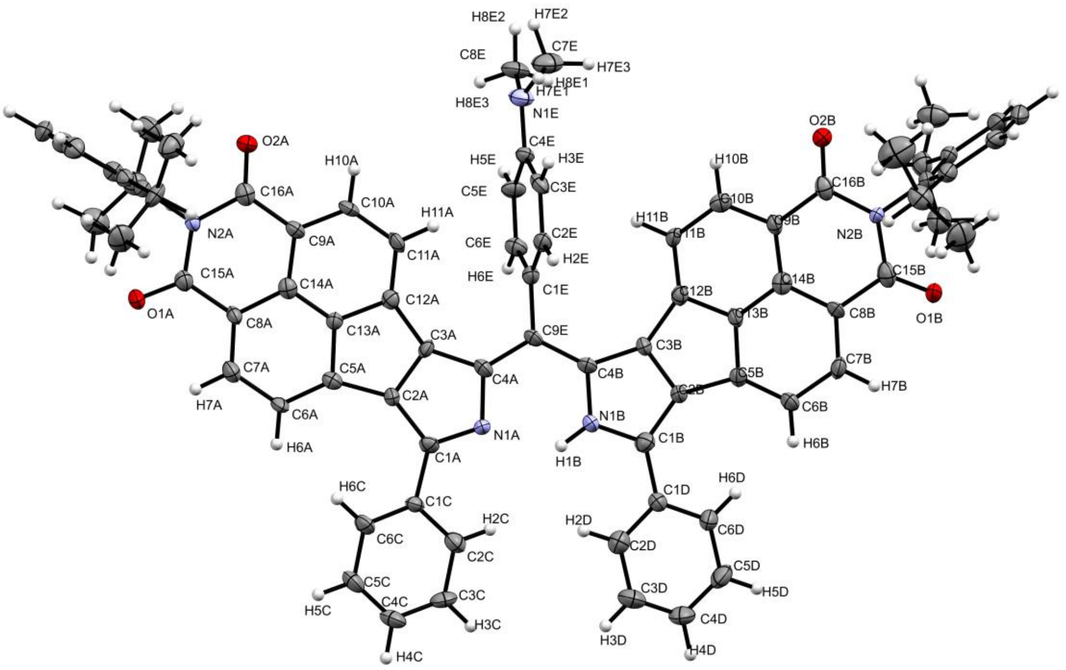

(b)

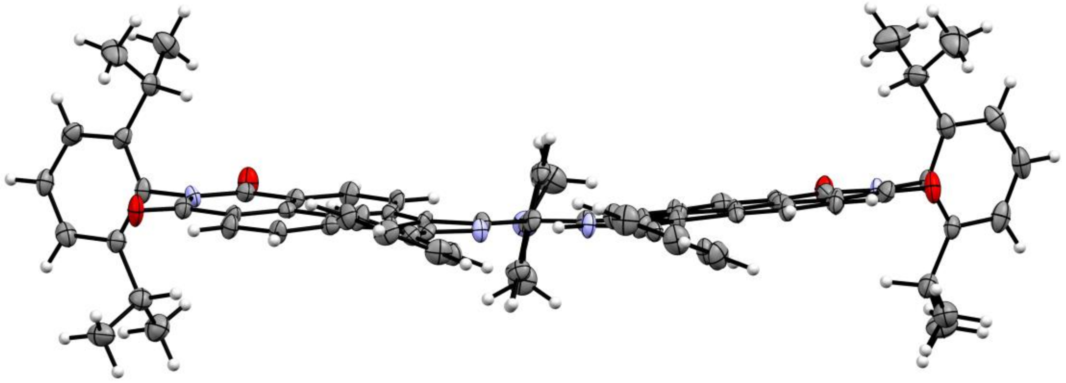

Figure S2. X-ray crystal structure (a) top view and (b) side view of **1b**. For clarity, all solvent molecules are omitted.

(a)

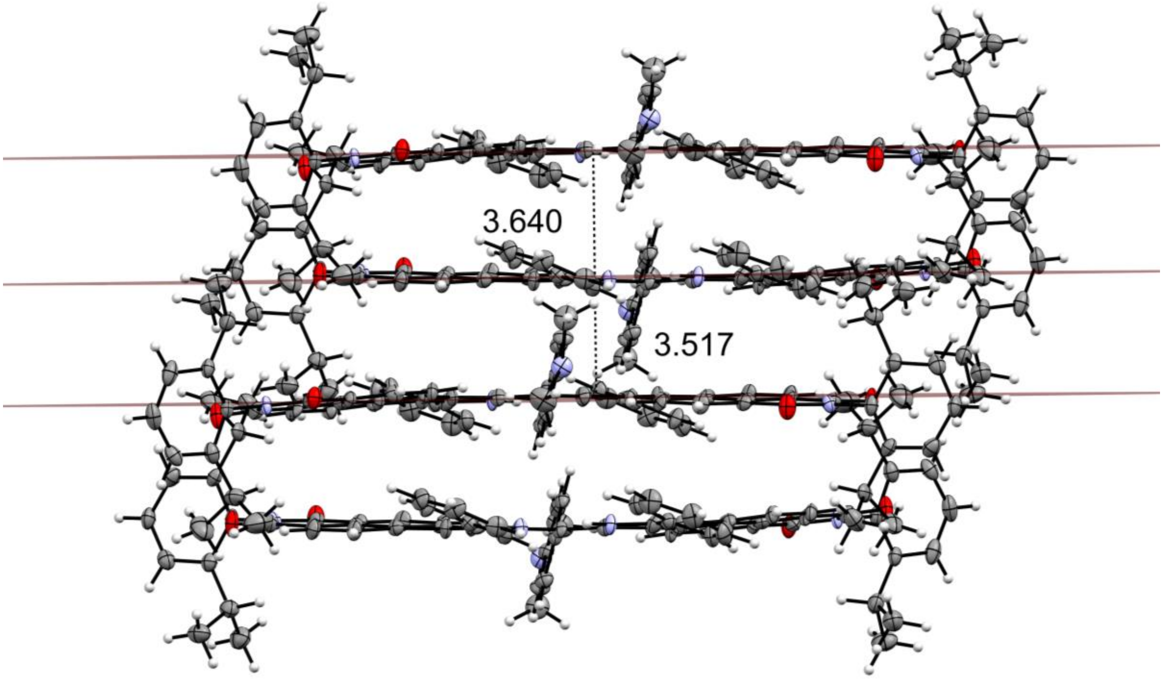

(b)

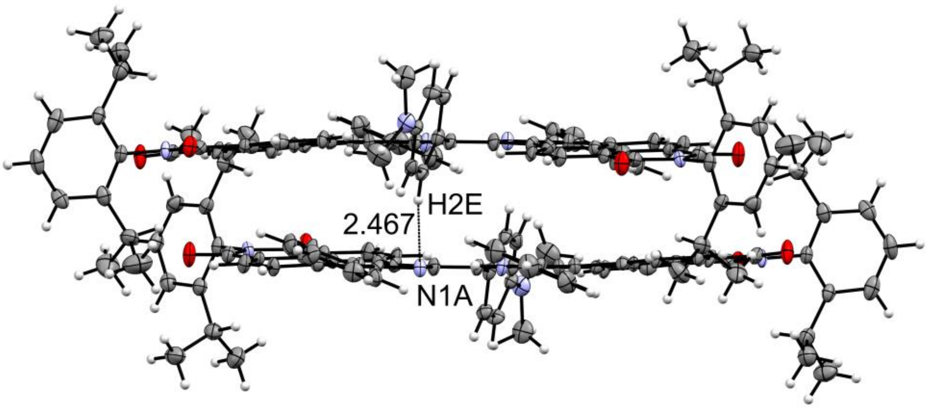

Figure S3. Short intermolecular (a) plane contacts and (b) hydrogen bonding (H--N) meso-aryl (H) - pyrrolic(N) interactions in the crystal structure of **1b**.

(a)

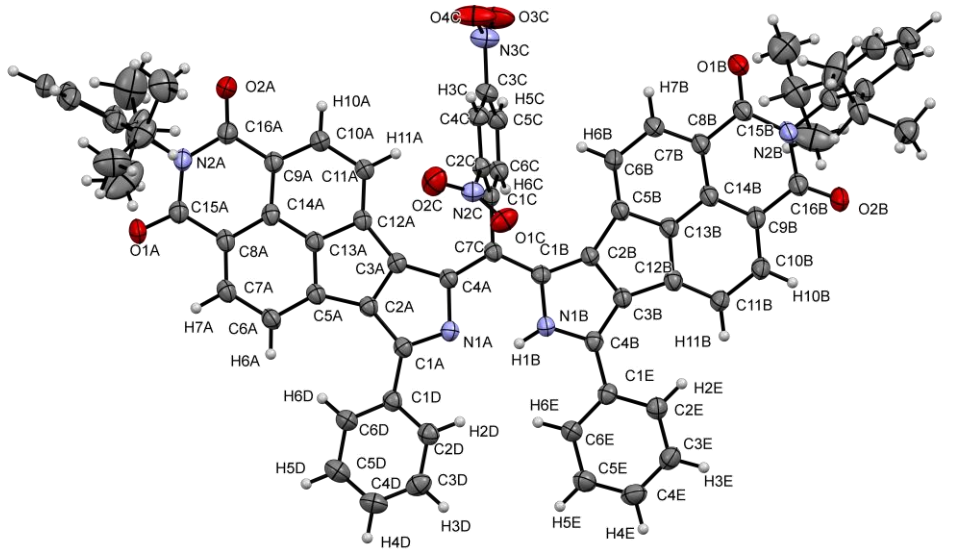

(b)

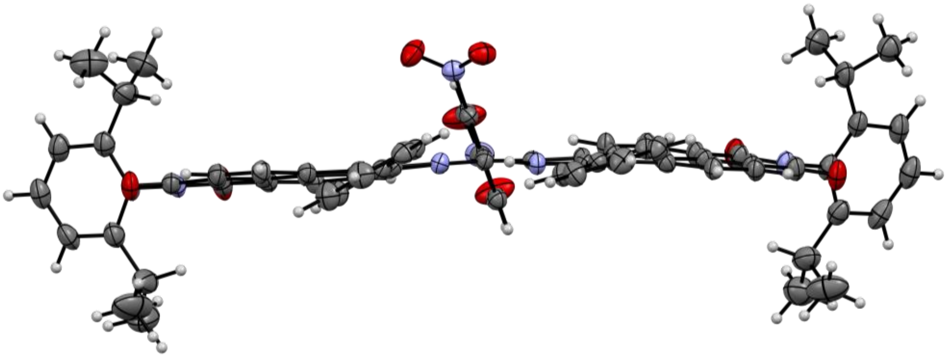

Figure S4. X-ray crystal structure (a) top view and (b) side view of **1e**. For clarity, all solvent molecules are omitted.

(a)

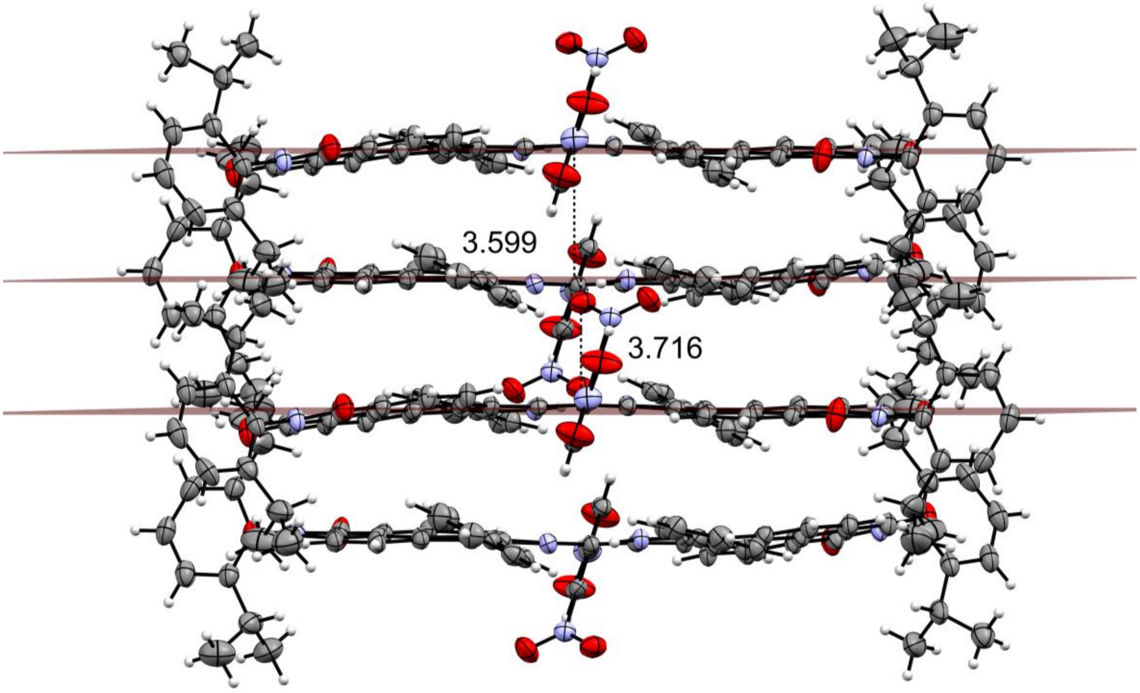

(b)

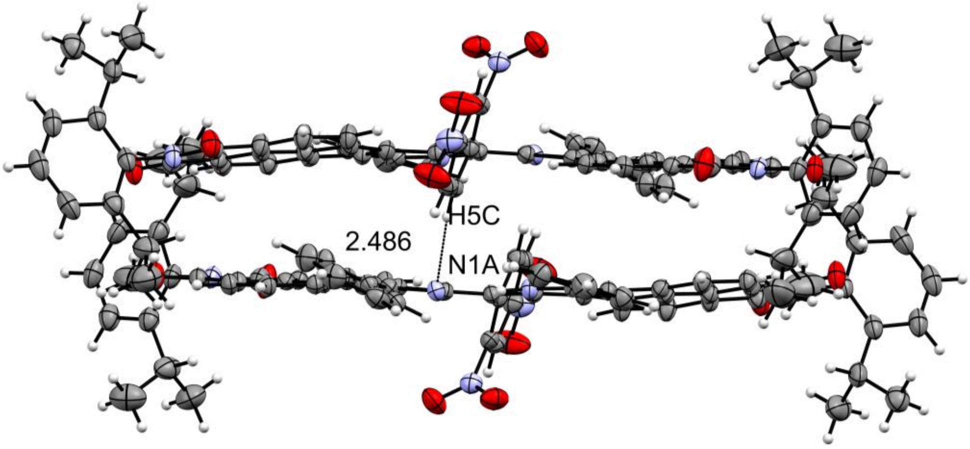

Figure S5. Short intermolecular (a) plane contacts and (b) hydrogen bonding (H--N) meso-aryl (H) - pyrrolic(N) interactions in the crystal structure of **1e**.

(a)

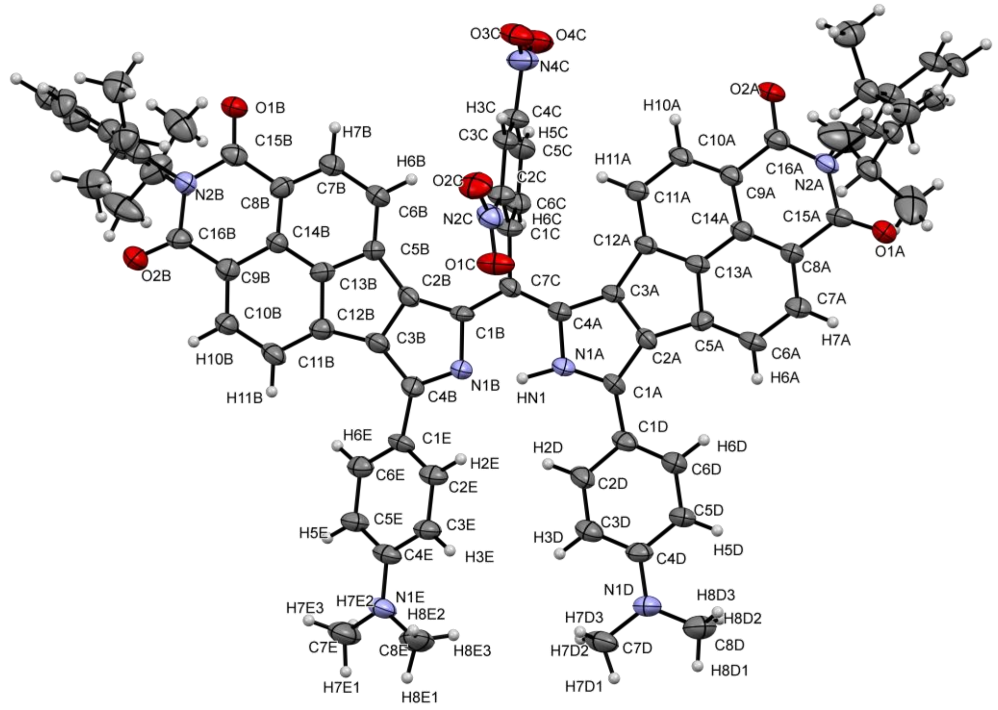

(b)

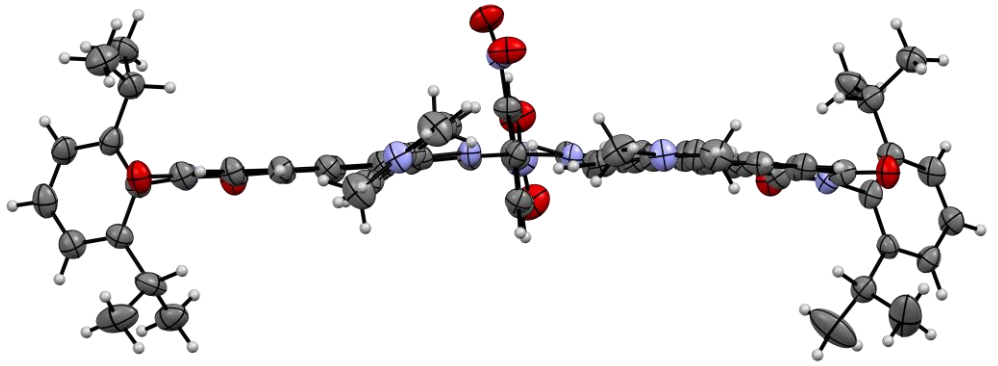

Figure S6. X-ray crystal structure of (a) top view and (b) side view of **2e**. For clarity, all solvent molecules are omitted.

(a)

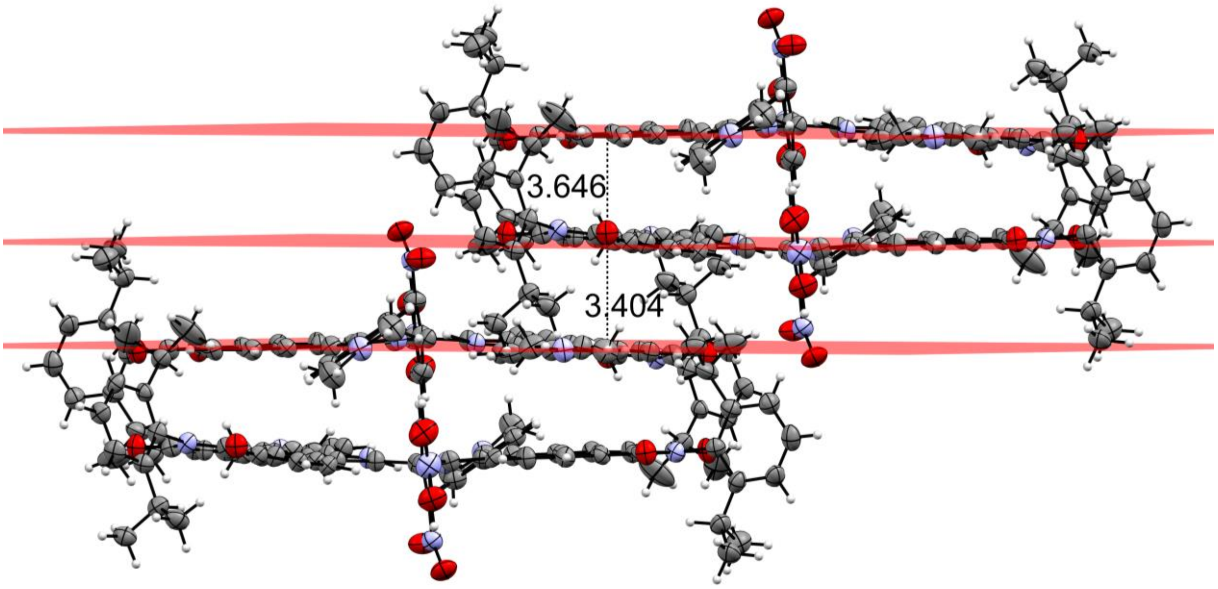

(b)

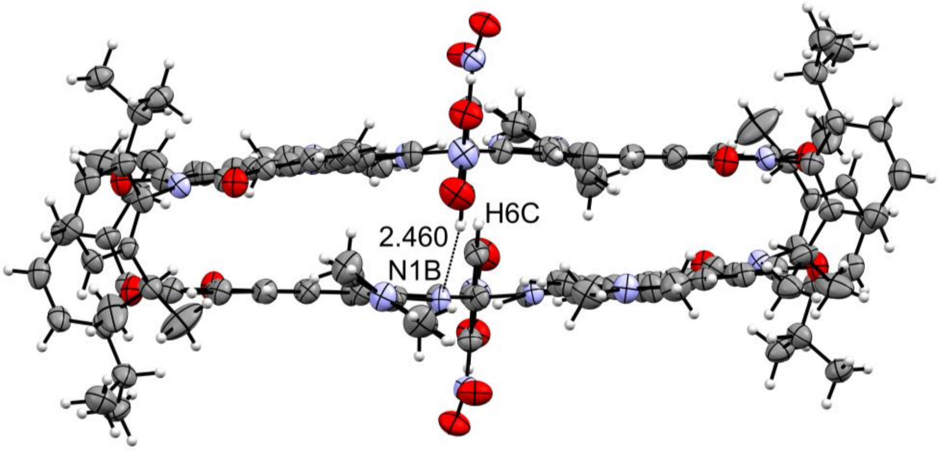

Figure S7. Short intermolecular (a) plane contacts and (b) hydrogen bonding (H--N) meso-aryl (H) - pyrrolic(N) interactions in the crystal structure of **2e**.

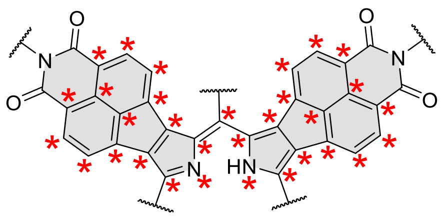

Figure S8. Atoms marked with red asterisk (*) are used to define the mean plane of dipyrrins.

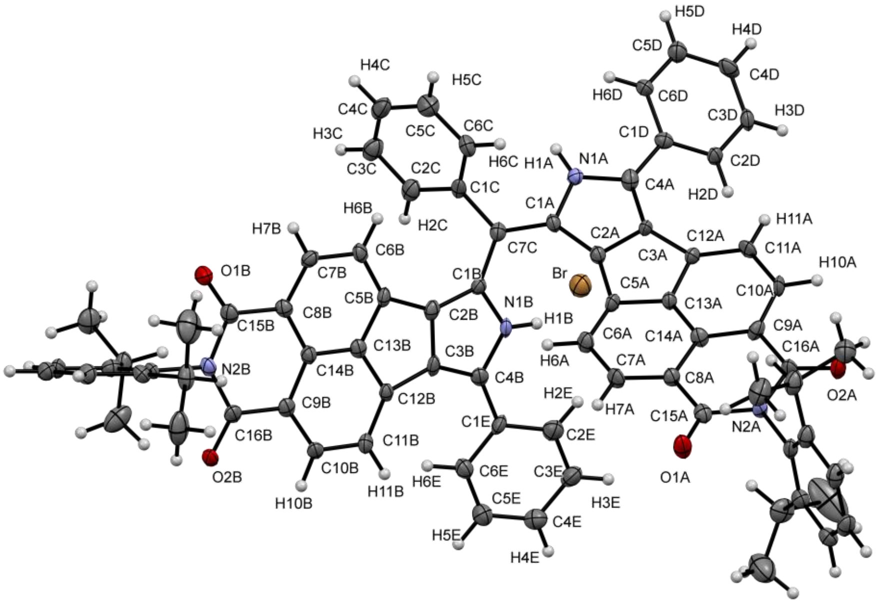

Figure S9. X-ray crystal structure of **1a**–H$^+$. For clarity, all solvent molecules are omitted. Brown sphere show bromine atom as a counter anion.

(a)

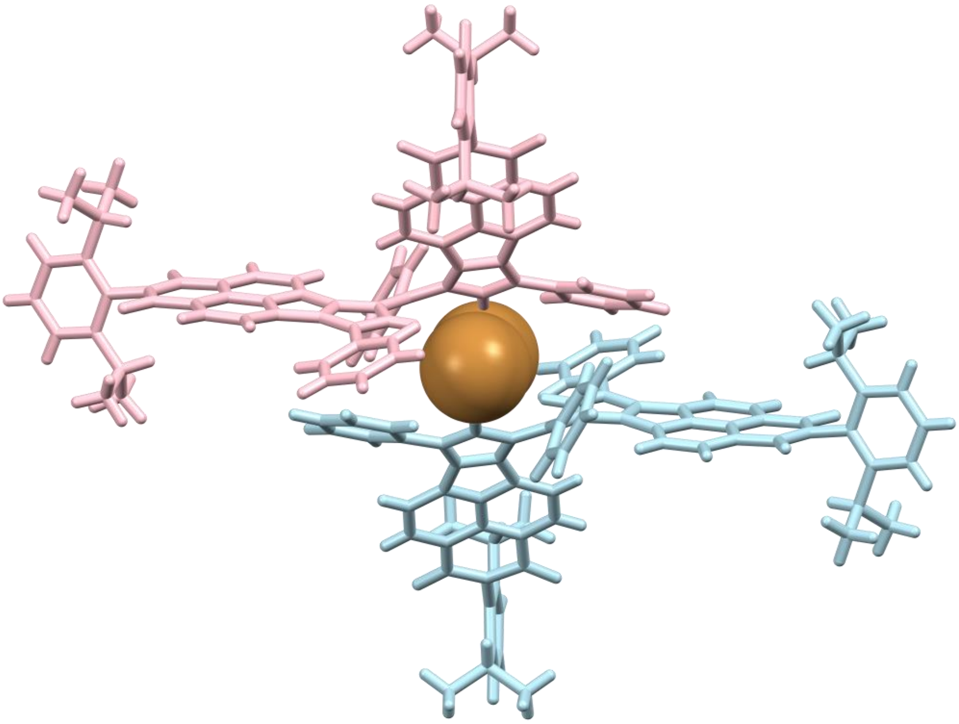

(b)

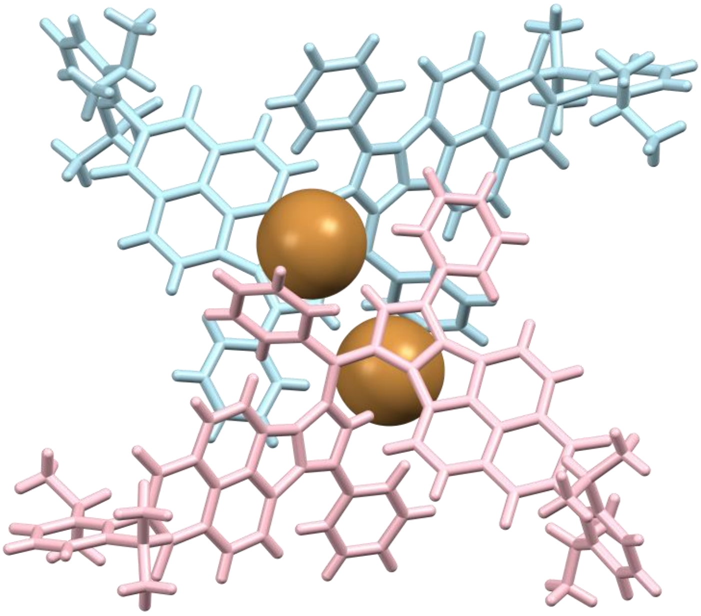

Figure S10. Cyclic dimer structure a) side view and b) top view of **1a**–H^+. For clarity, all solvent molecules are omitted. Brown sphere show bromine atom as a counter anion.

(a)

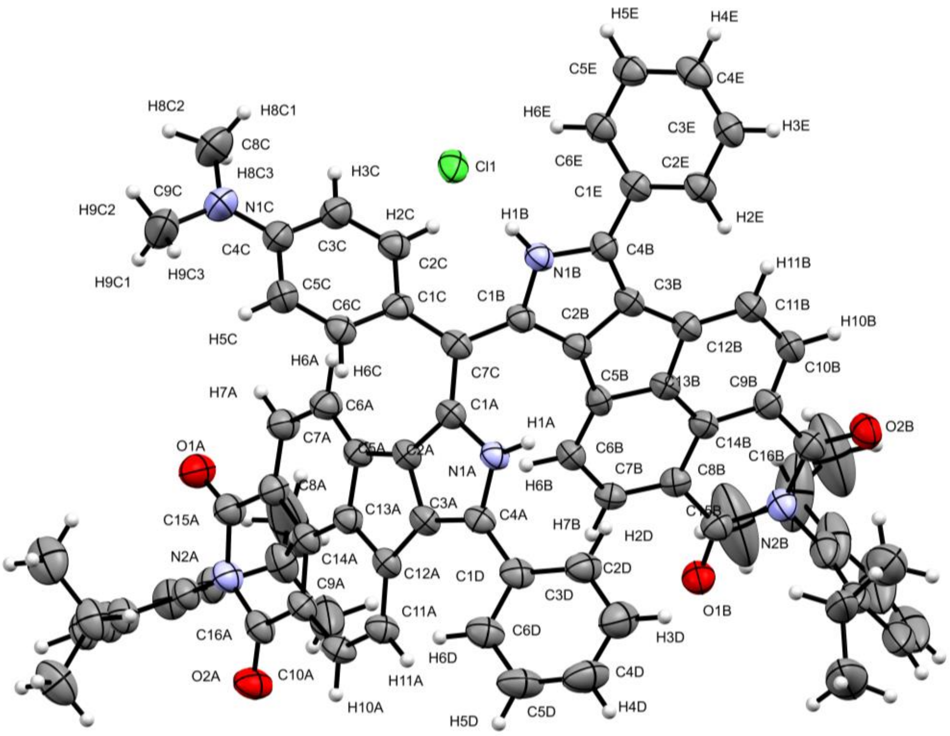

Figure S11. X-ray crystal structure of **1b**–H^{+}. For clarity, all solvent molecules are omitted. Green sphere show chlorine atom as a counter anion.

(a)

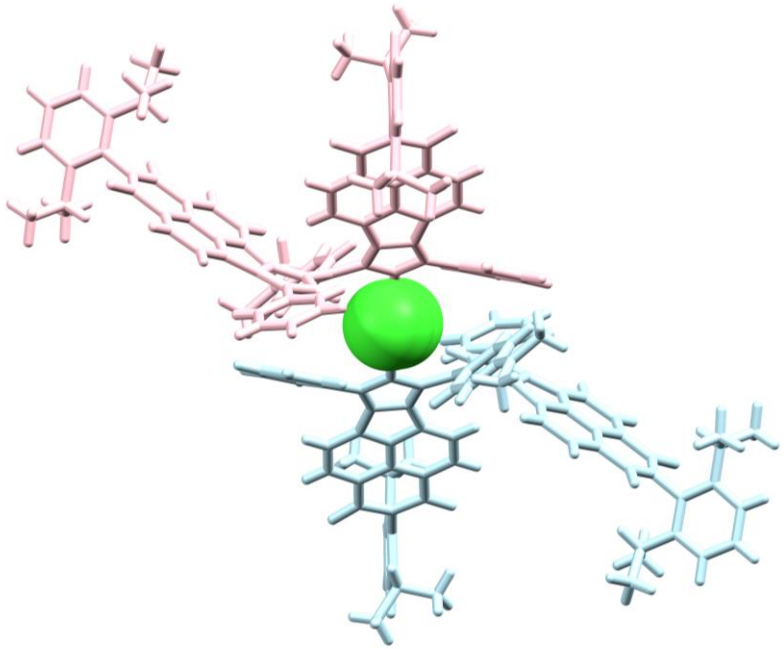

(b)

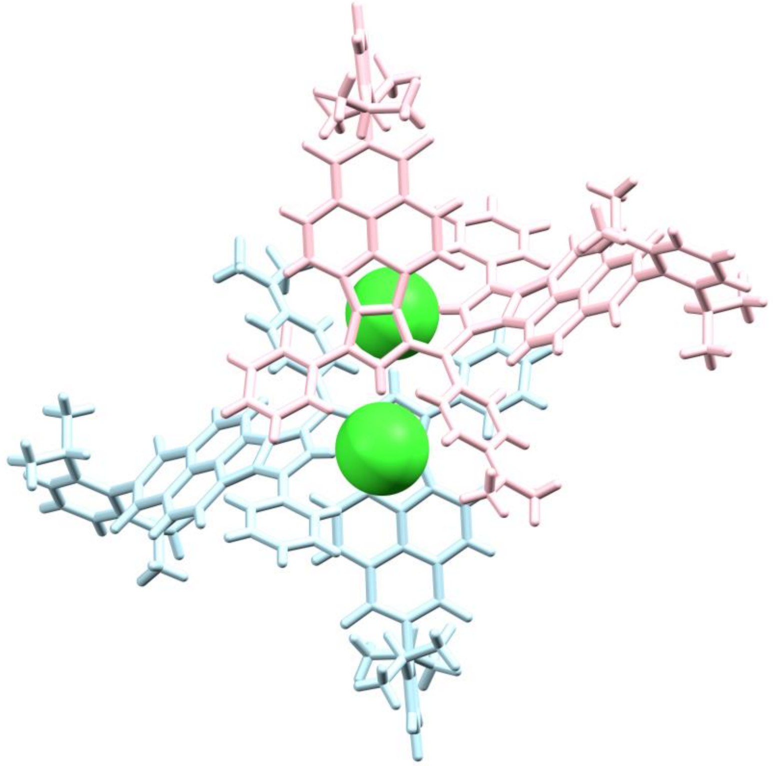

Figure S12. Cyclic dimer structure a) side view and b) top view of **1b**–H$^+$ For clarity, all solvent molecules are omitted. Green sphere show chlorine atom as a counter anion.

(a)

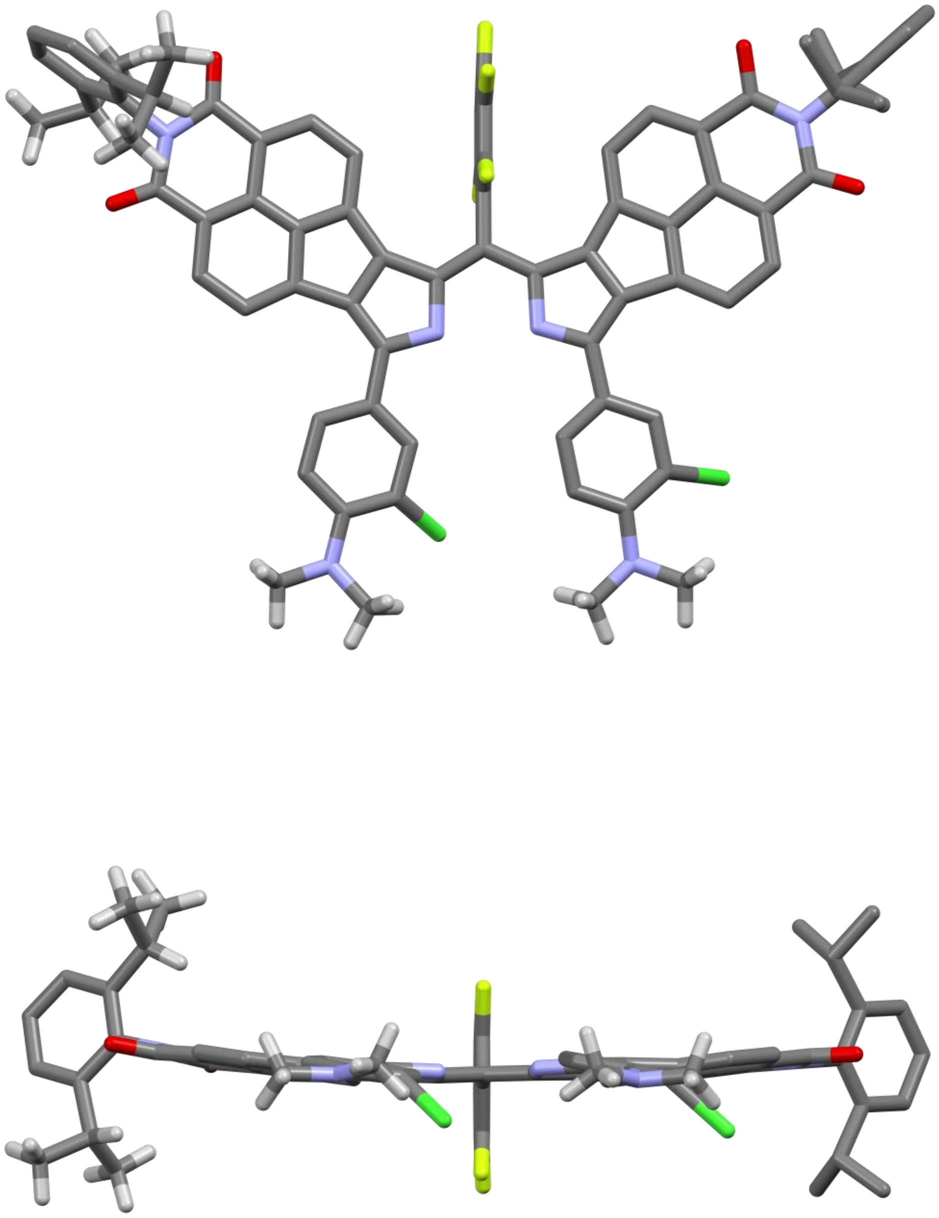

(b)

Figure S13. Preliminary X-ray crystal structure (stick views) of (a) top view and (b) side view of **3d**. For clarity, all solvent molecules are omitted. In this structure, chlorine element occupancy was found to be 10.8% and 31.2%.

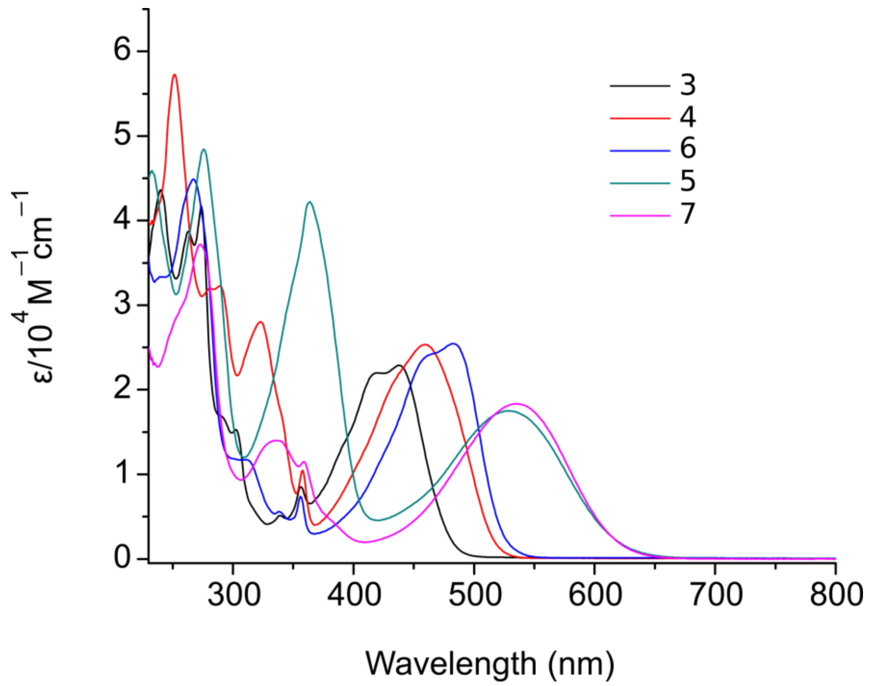

Figure S14. Absorption spectra of **3**–**7** in dichloromethane at 293K.

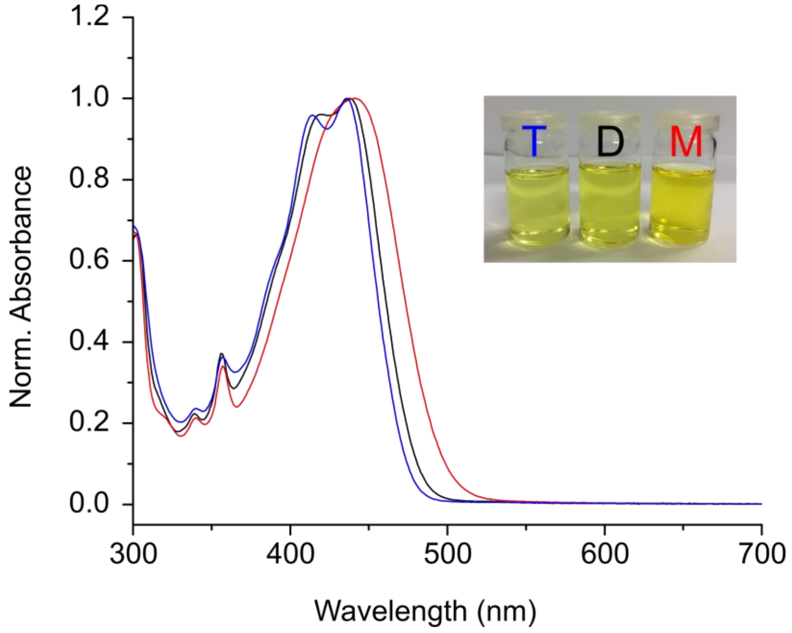

Figure S15. Absorption spectra of **3** in different solvents (blue line - toluene solution, black line - dichloromethane solution, red line - methanol solution). (Insets shows the solution in different solvents at ≈ 60 µM concentrations).

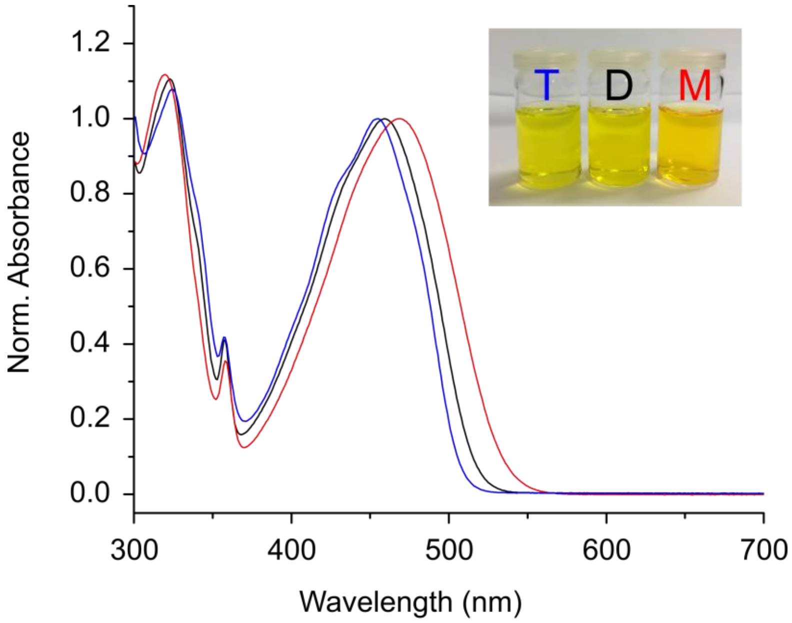

Figure S16. Absorption spectra of **4** in different solvents (blue line - toluene solution, black line - dichloromethane solution, red line - methanol solution). (Insets shows the solution in different solvents at ≈ 60 µM concentrations).

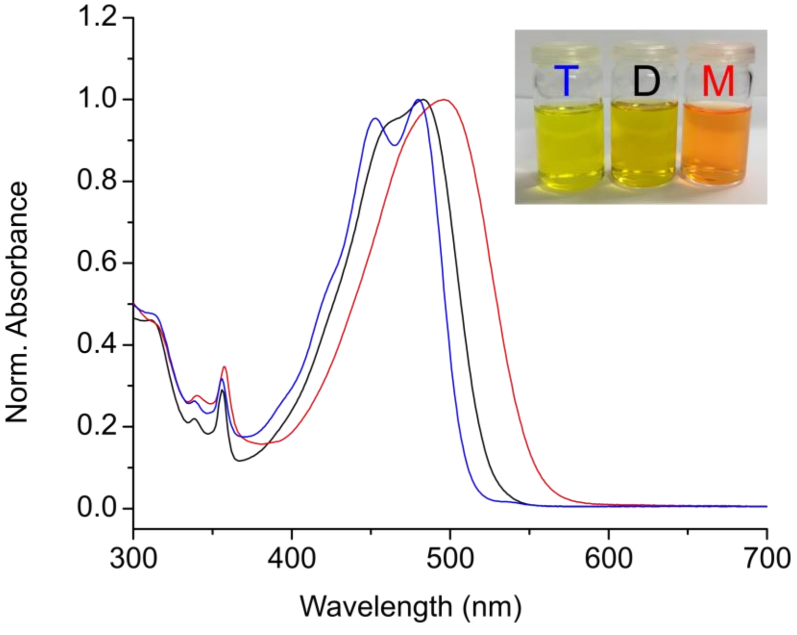

Figure S17. Absorption spectra of **6** in different solvents (blue line - toluene solution, black line - dichloromethane solution, red line - methanol solution). (Insets shows the solution in different solvents at ≈ 60 µM concentrations).

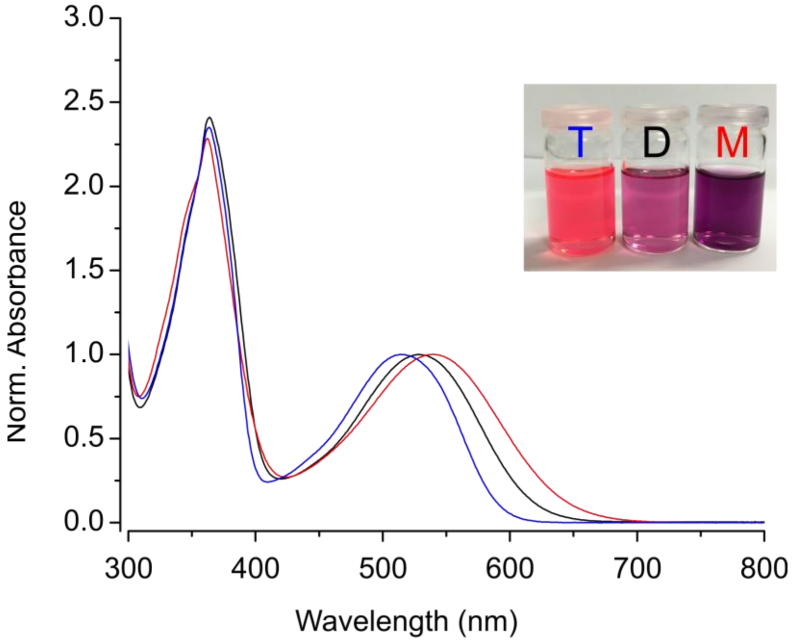

Figure S18. Absorption spectra of **5** in different solvents (blue line - toluene solution, black line - dichloromethane solution, red line - methanol solution). (Insets shows the solution in different solvents at ≈ 60 µM concentrations).

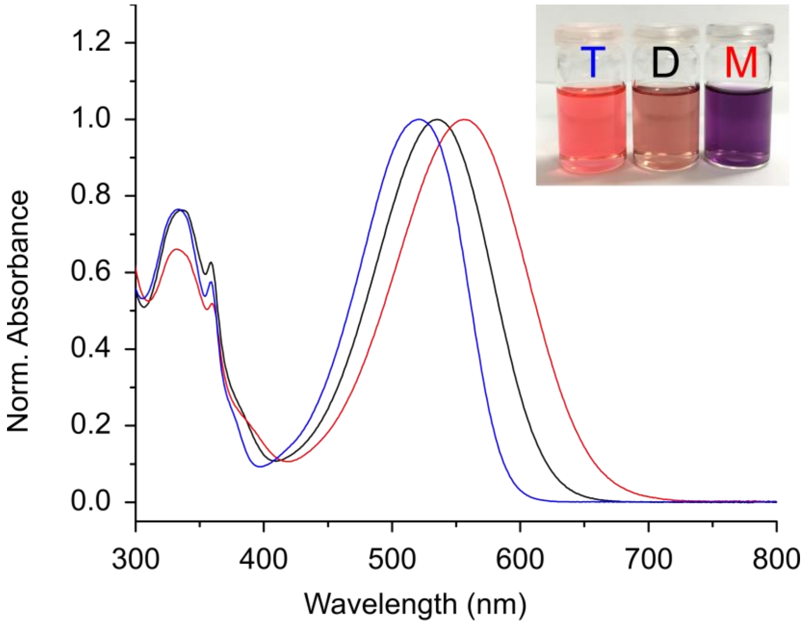

Figure S19. Absorption spectra of **7** in different solvents (blue line - toluene solution, black line - dichloromethane solution, red line - methanol solution). (Insets shows the solution in different solvents at ≈ 60 µM concentrations).

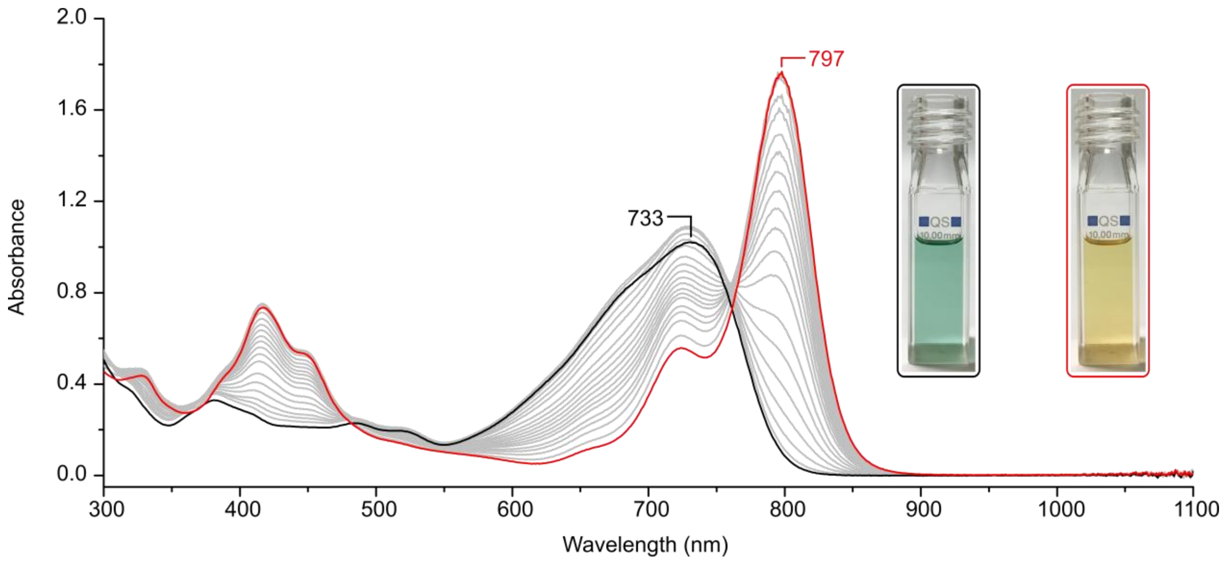

Figure S20. Absorption spectral change of **1d** in CH_2Cl_2 seen upon the addition of TFA with increasing concentrations from stock solution. The black spectral line represents the neutral form and the red spectral line represents the protonated form upon addition of acid.

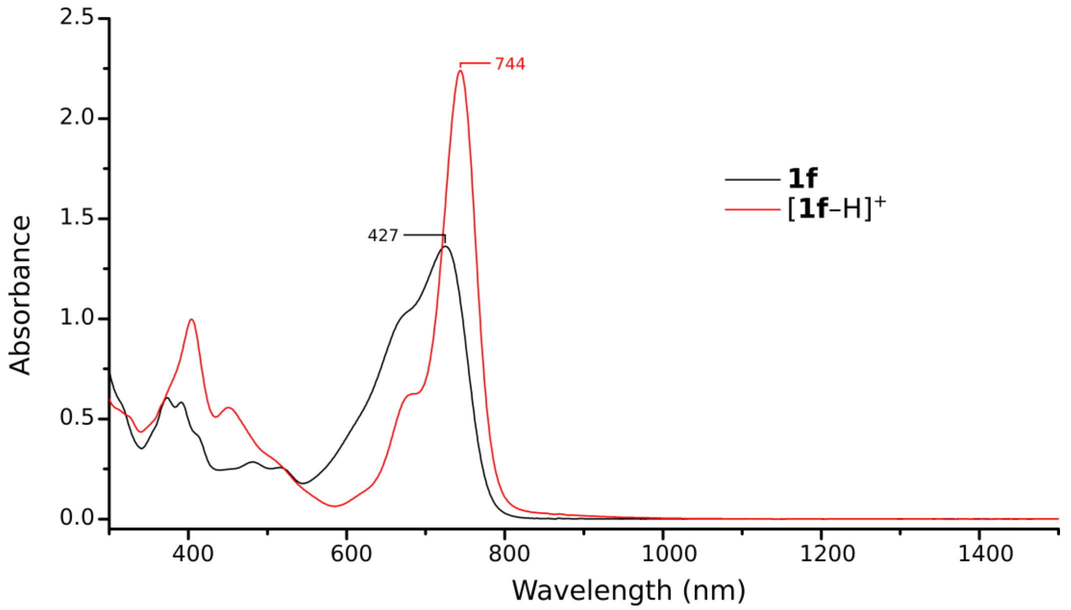

Figure S21. Absorption spectral change of **1f** in CH_2Cl_2 seen upon the addition of TFA. The black spectral line represents the neutral form and the red spectral line represents the protonated form.

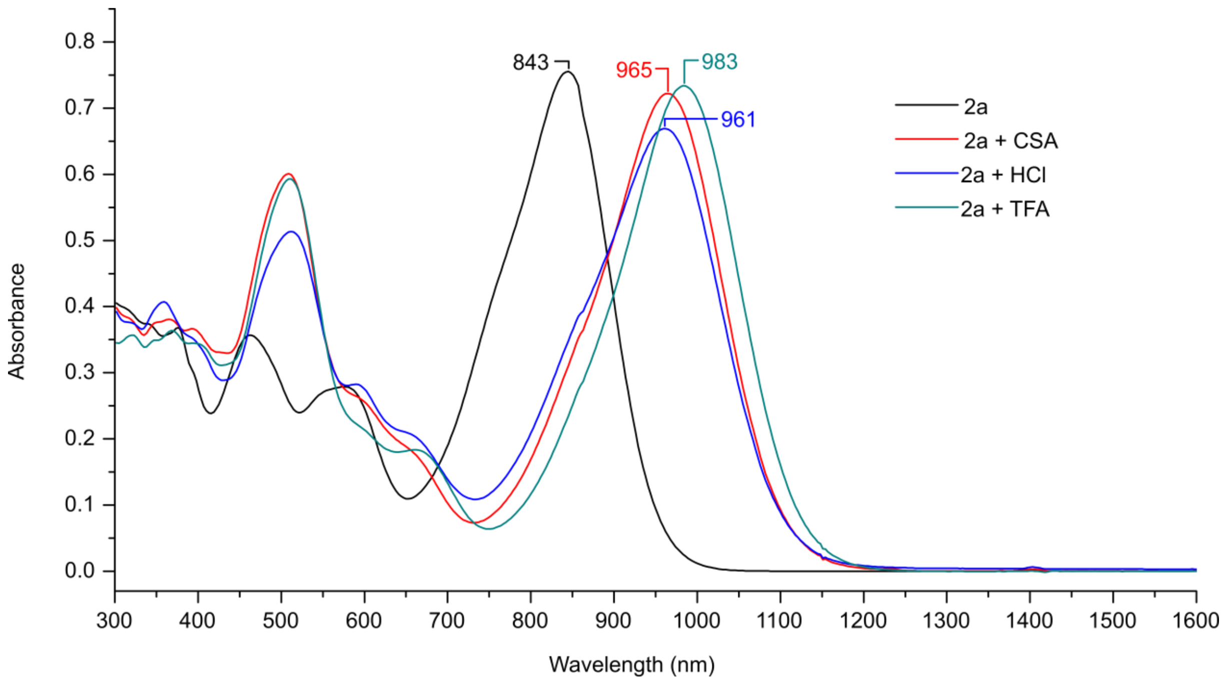

Figure S22. Absorption spectra showing the neutral and the first protonation state of **2a** in CH_2Cl_2 at 293K using various acid source, CSA, HCl, and TFA.

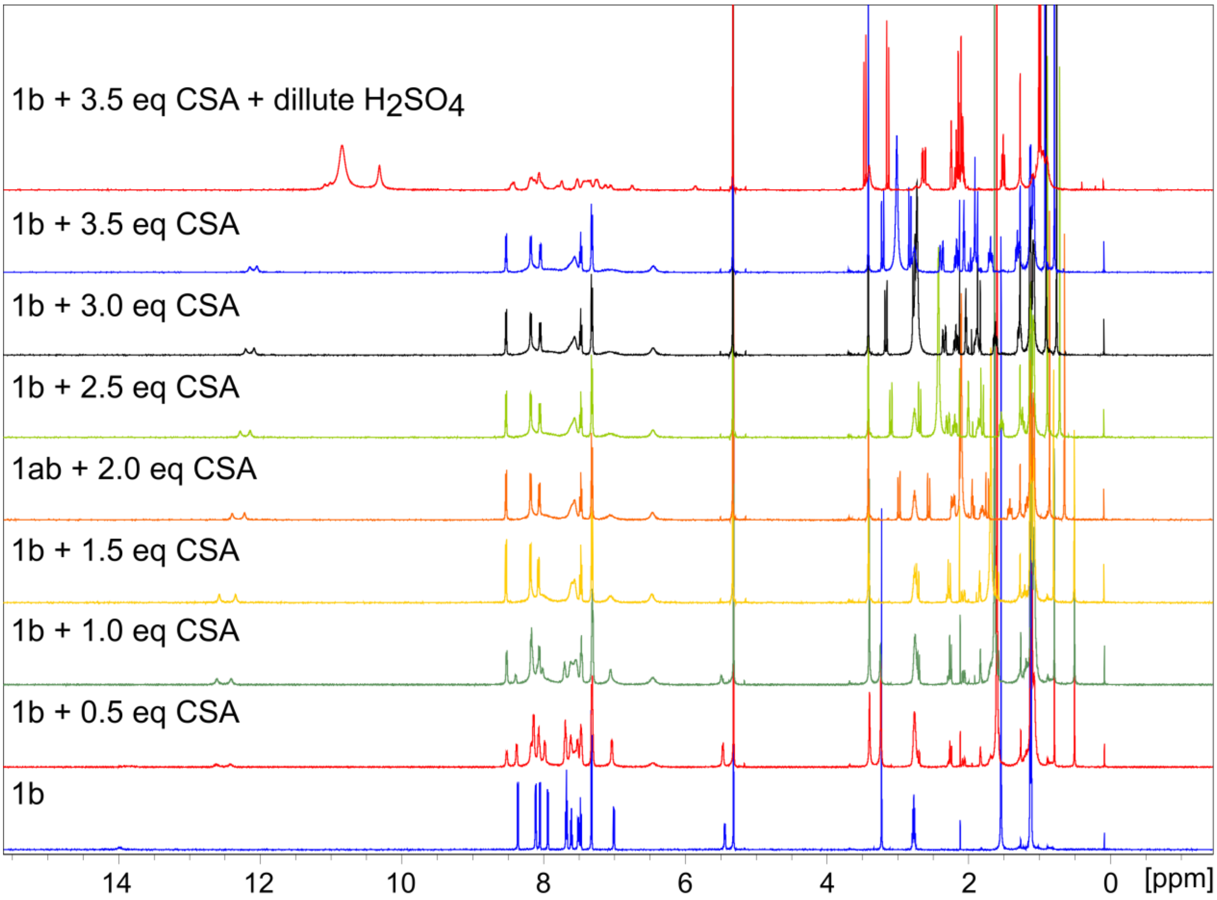

Figure S23. Comparison of ^{1}H NMR spectra upon stepwise addition of camphorsulphonic acid (CSA) to dipyrrin **1b** recorded in $CDCl_3$ at room temperature. The complete protonation was achieved by using a drop of 98% H2SO$_4$ solution dissolved in $CDCl_3$.

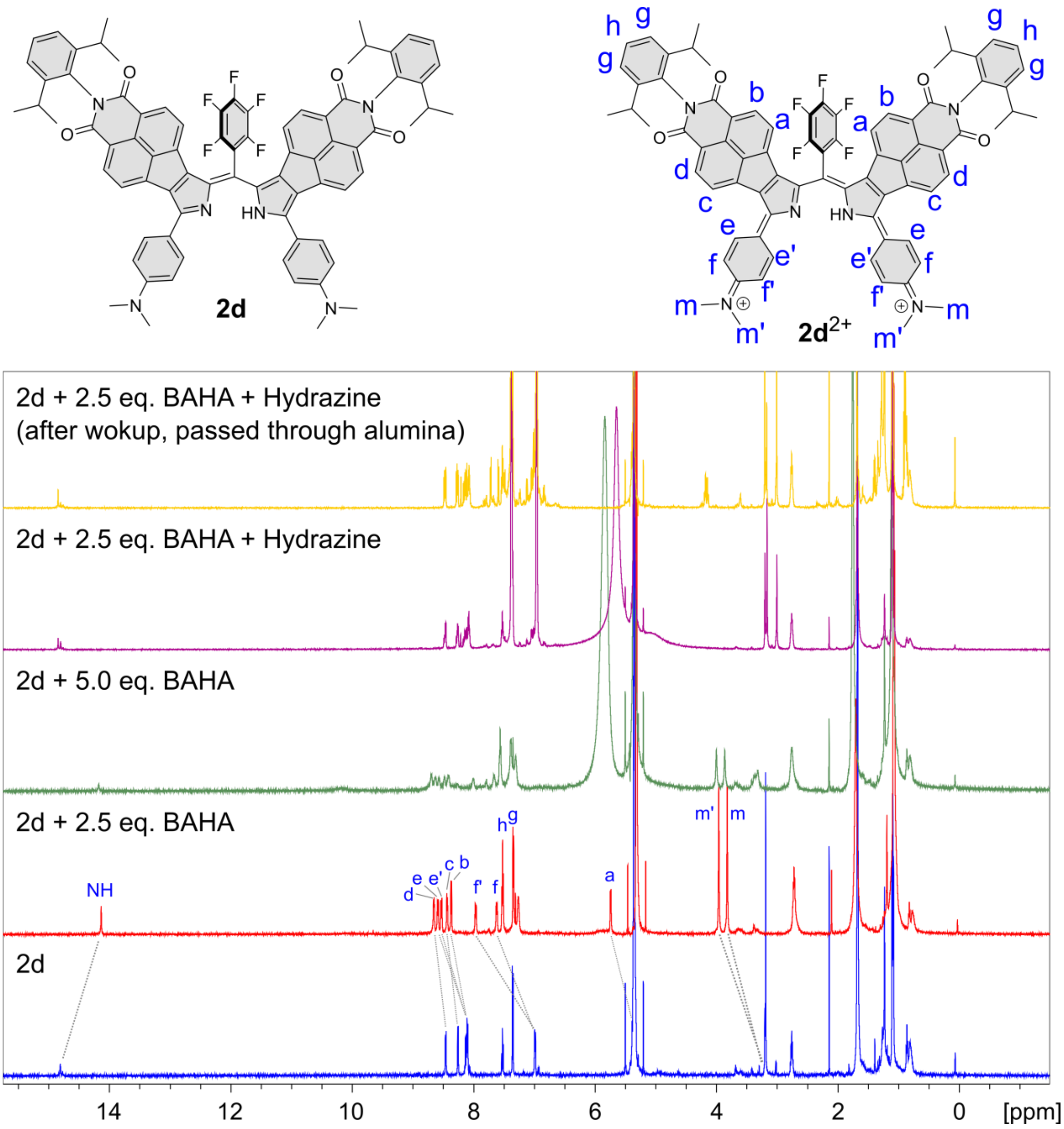

Figure S24. Comparison of ^{1}H NMR spectra upon addition of 0-5.0 equiv. of BAHA (dissolved in CD_2Cl_2) to dipyrrin **2d** recorded in CD_2Cl_2 at 240 K.

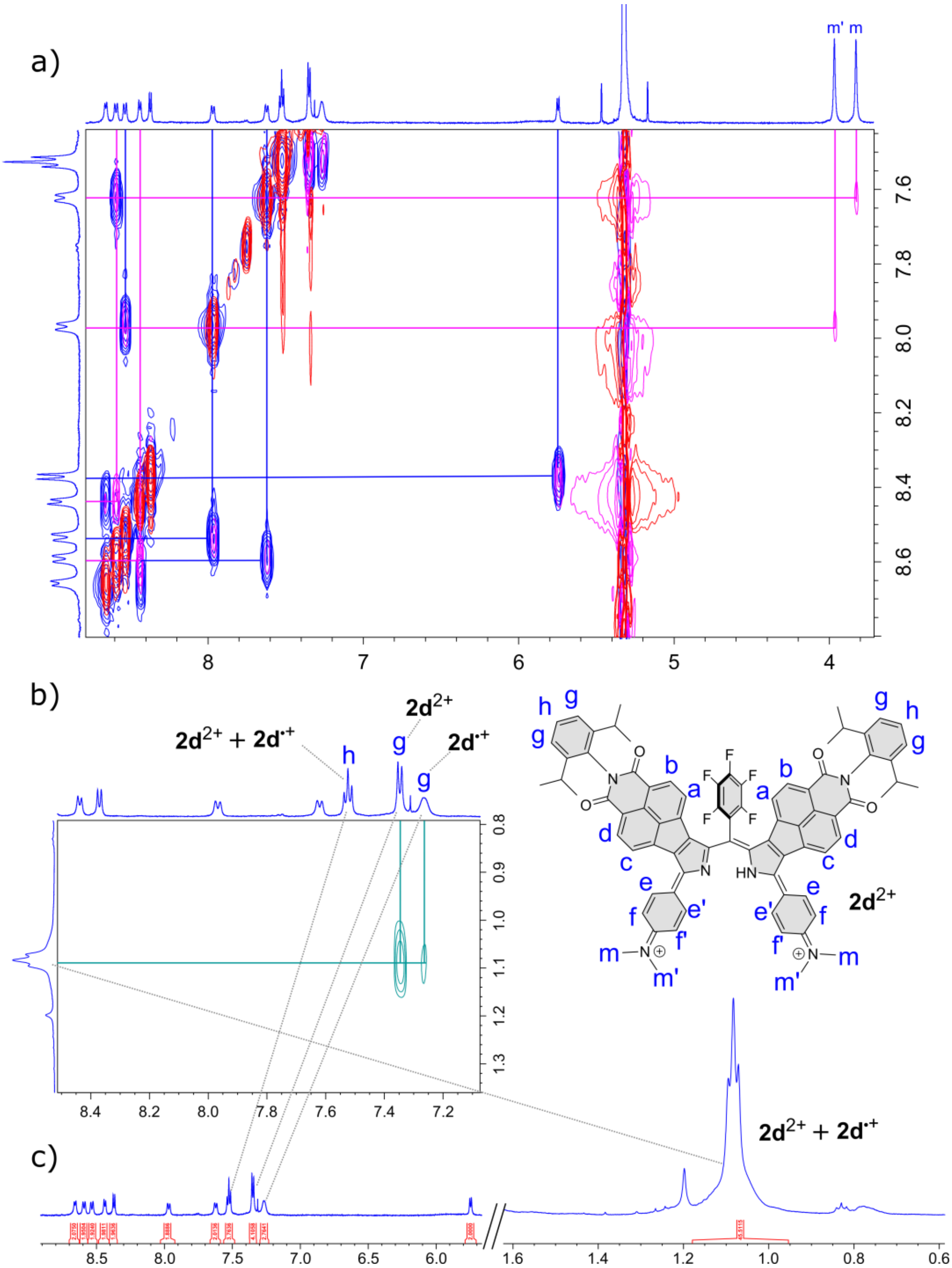

Figure S25. A) Overlay of partial COSY (red) and ROESY (green/blue) of chemically oxidized **2d** using 2.5 equivalent of BAHA recorded in CD_2Cl_2 at 240 K. B) Partial ROESY spectrum showing the iPr group peak signals corresponding to the presence of both radical monocation and dication species. c) Partial ^{1}H NMR showing the integral peaks of selected region which are assigned to the peaks in ROESY spectrum suggesting the radical monocation is in exchange with dication species.

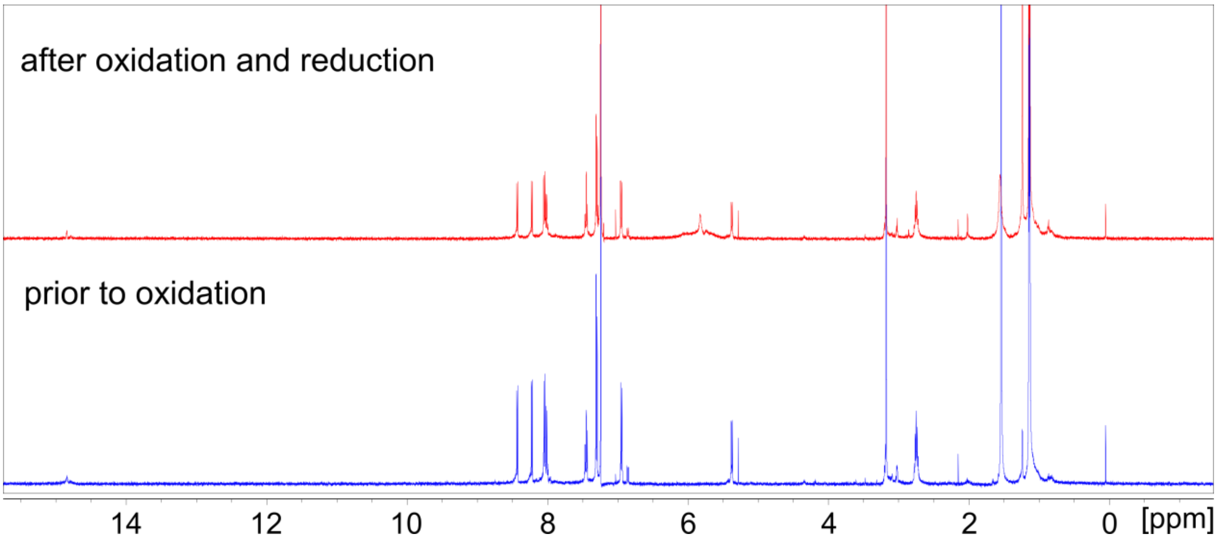

Figure S26. Reversibility of chemical oxidation. Comparison of ^{1}H NMR spectra of **2d** and reduced form of oxidized dipyrrin recorded in $CDCl_3$ at room temperature. In this experiment, $AgPF_6$ was used an oxidant and the solid was added to the NMR tube containing dipyrrin solution; KO_2 was used as reductant and the solid was added to the NMR tube containing oxidized dipyrrin.

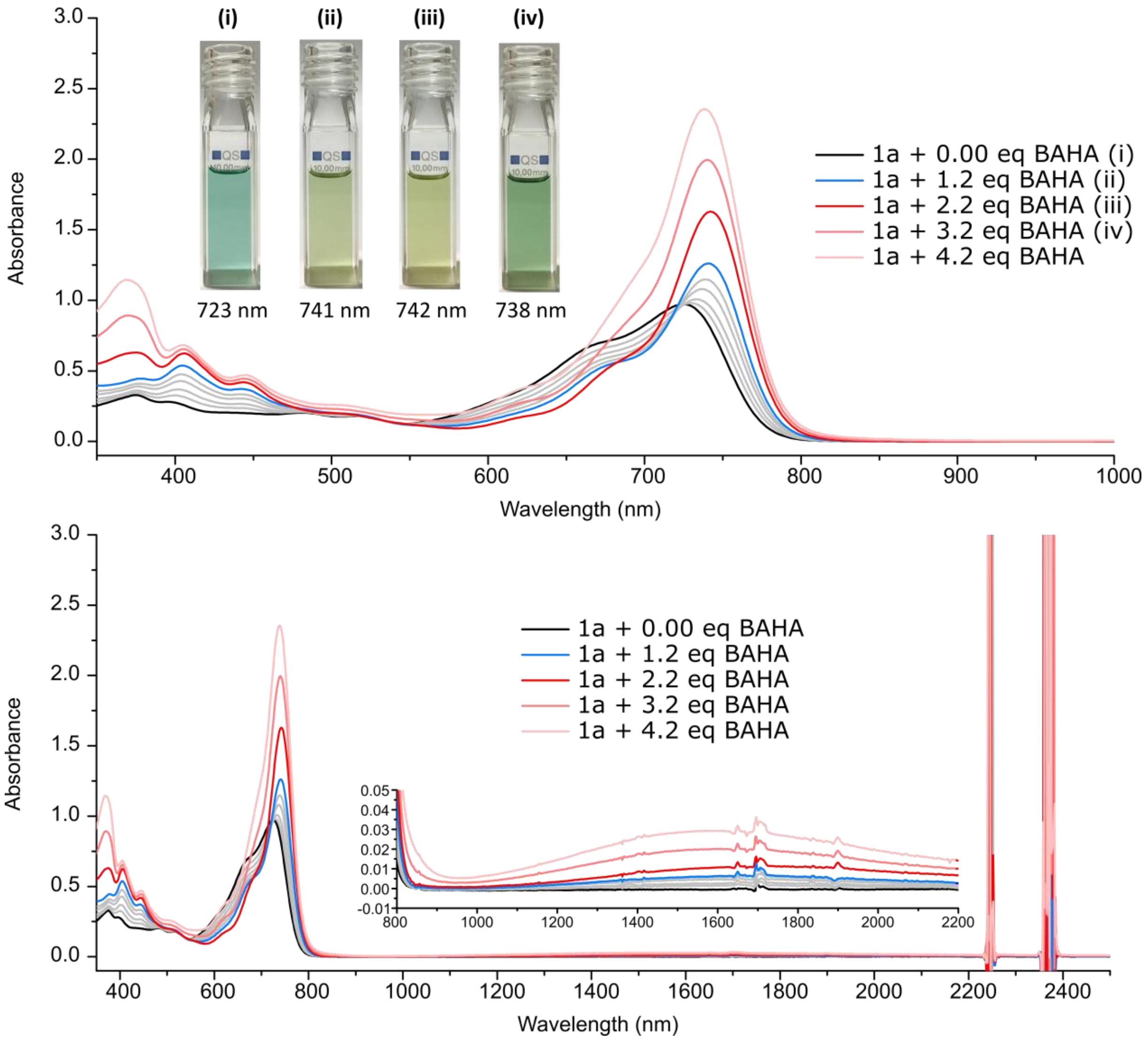

Figure S27. Chemical oxidation investigated for **1a** in CH_2Cl_2 at 293K using BAHA as chemical oxidant; black line represents the neutral dipyrrin; inset in the bottom figure shows the formation of radical dipyrrin.

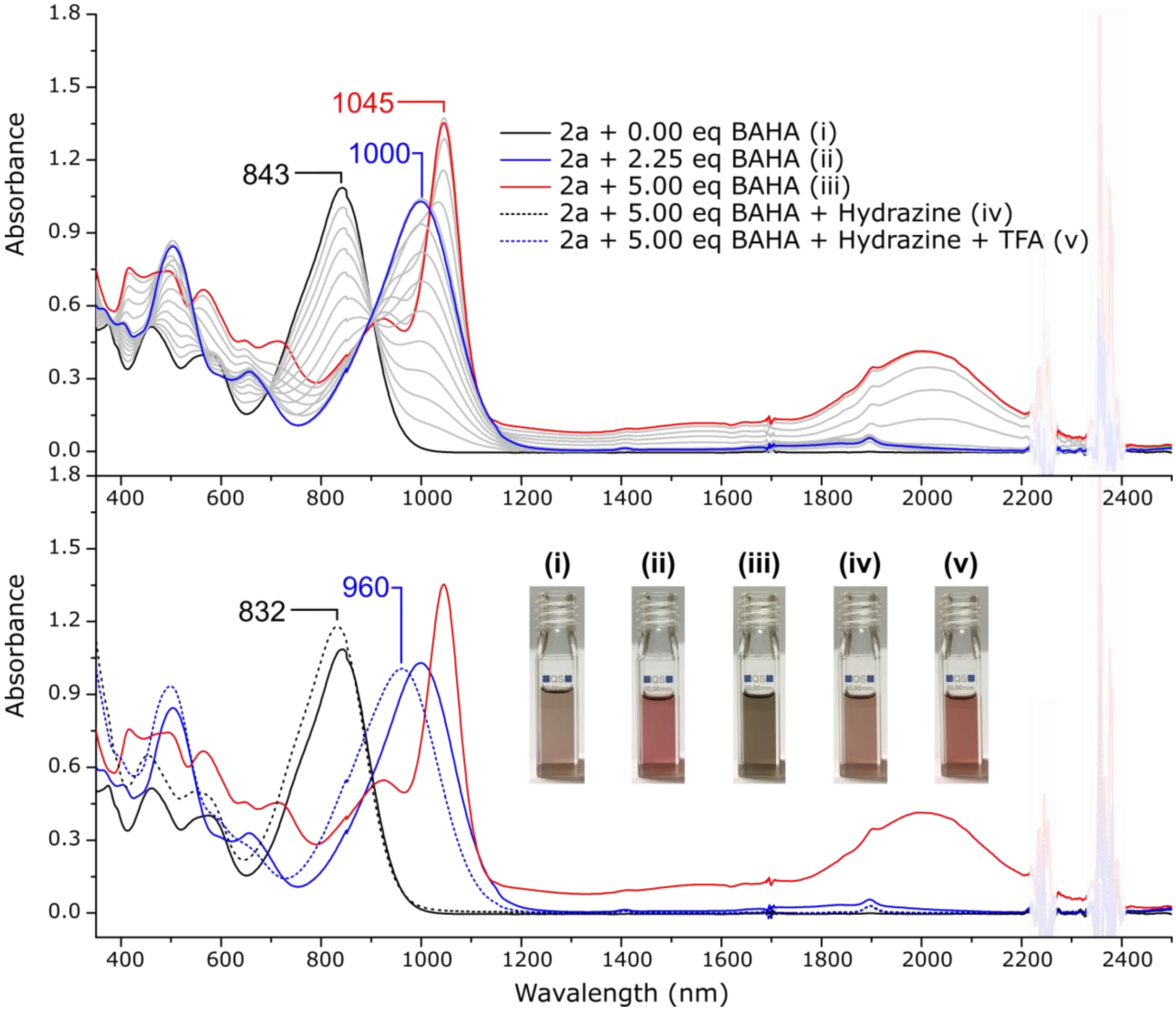

Figure S28. Chemical oxidation investigated for **2a** in CH_2Cl_2 at 293K using BAHA as chemical oxidant. In the top figure, black line corresponds to the neutral dipyrrin; blue line corresponds to the dicationic solution; red line corresponds to the protonated dicationic solution. In the bottom figure, dashed black line corresponds to the reduction with excess hydrazine hydrate solution; dashed blue line sorresponds to the protonated form of the partially chlorinated dipyrrin.

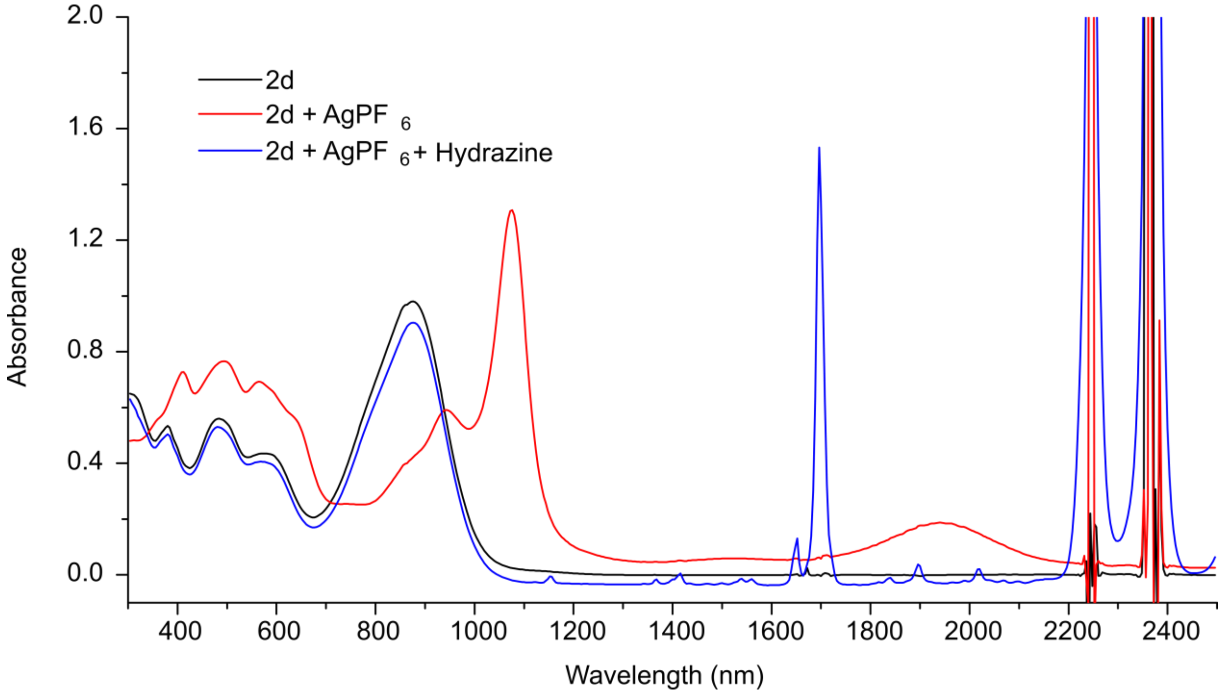

Figure S29. Reversibility of chemical oxidation investigated for **2d** in CH_2Cl_2 at 293K using $AgPF_6$ as chemical oxidant. The blue curve was obtained upon adding hydrazine hydrate dissolved in dichloromethane to the oxidized **2d** dipyrrin.

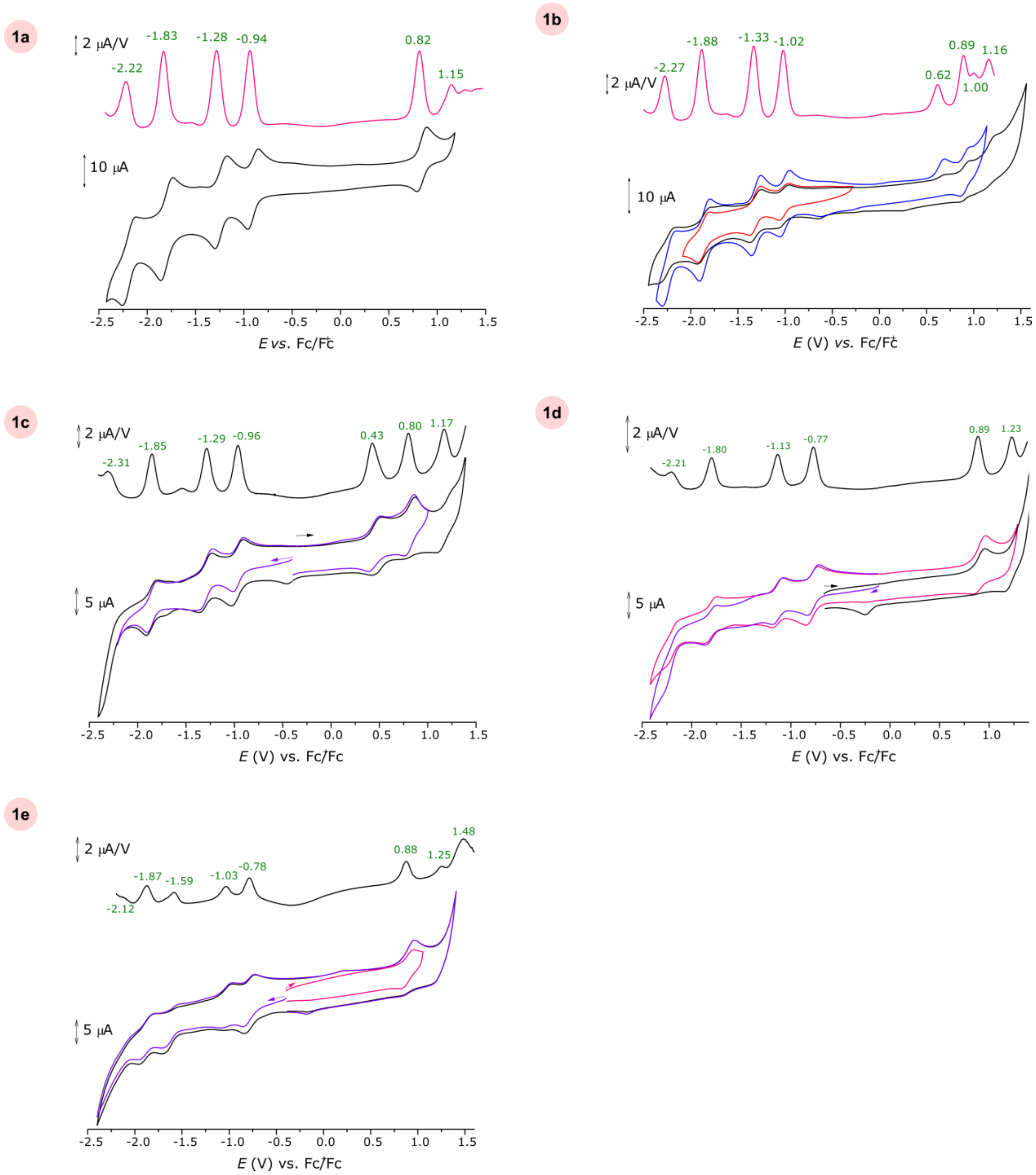

Figure S30. Differential pulse voltammogram (top curve) and cyclic voltammogram (bottom curves) for dipyrrin **1a**–**e** in different potential setup (dichloromethane solvent, $[Bu_4N]PF_6$ as supporting electrolyte; glassy carbon as working electrode, Au wire as pseudoreference electrode, and Pt rod as counter electrode, scan rate: 100 mV/s.

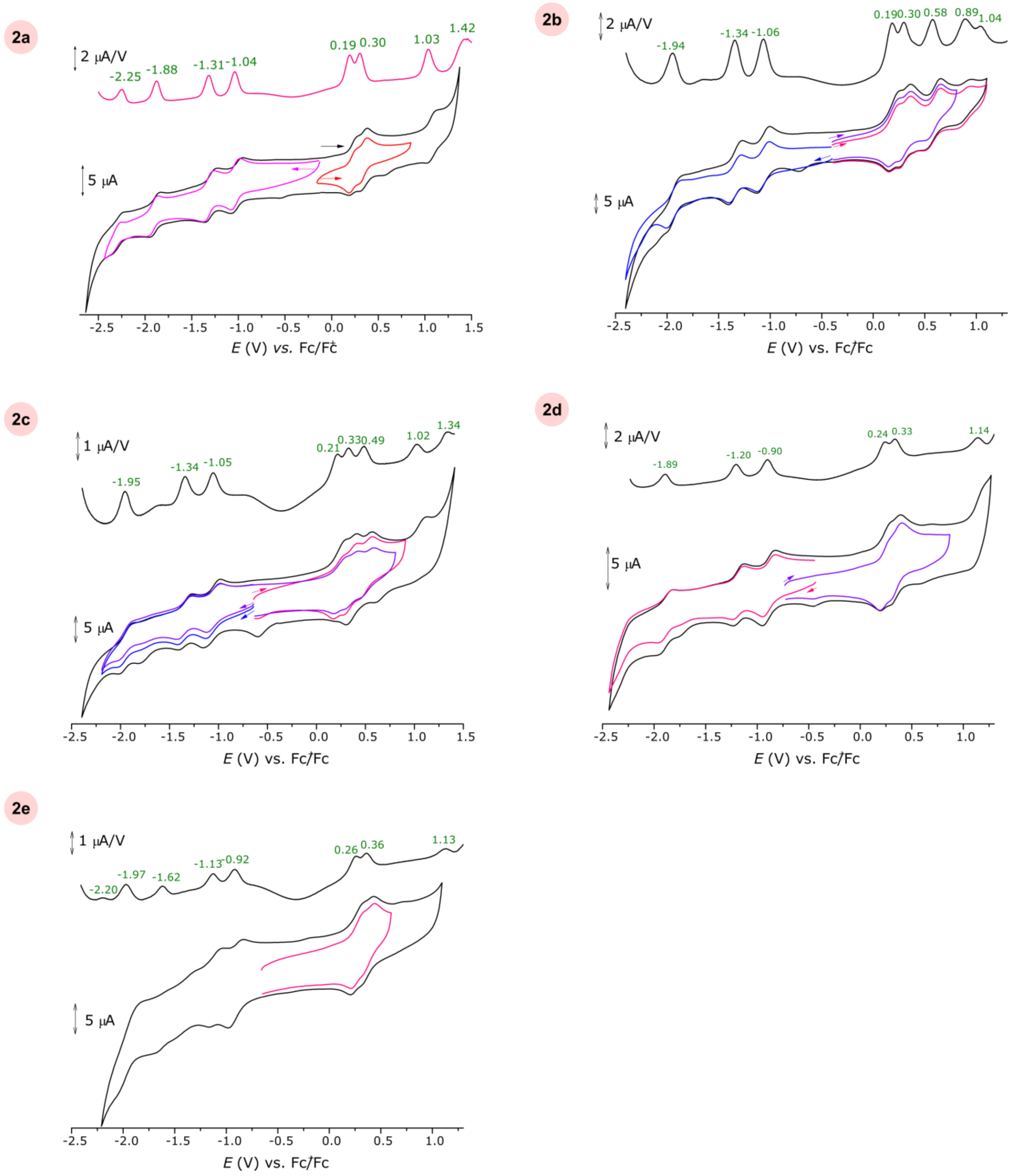

Figure S31. Differential pulse voltammogram (top curve) and cyclic voltammogram (bottom curves) for dipyrrin **2a**–**e** in different potential setup (dichloromethane solvent, $[Bu_4N]PF_6$ as supporting electrolyte; glassy carbon as working electrode, Au wire as pseudoreference electrode, and Pt rod as counter electrode, scan rate: 100 mV/s.

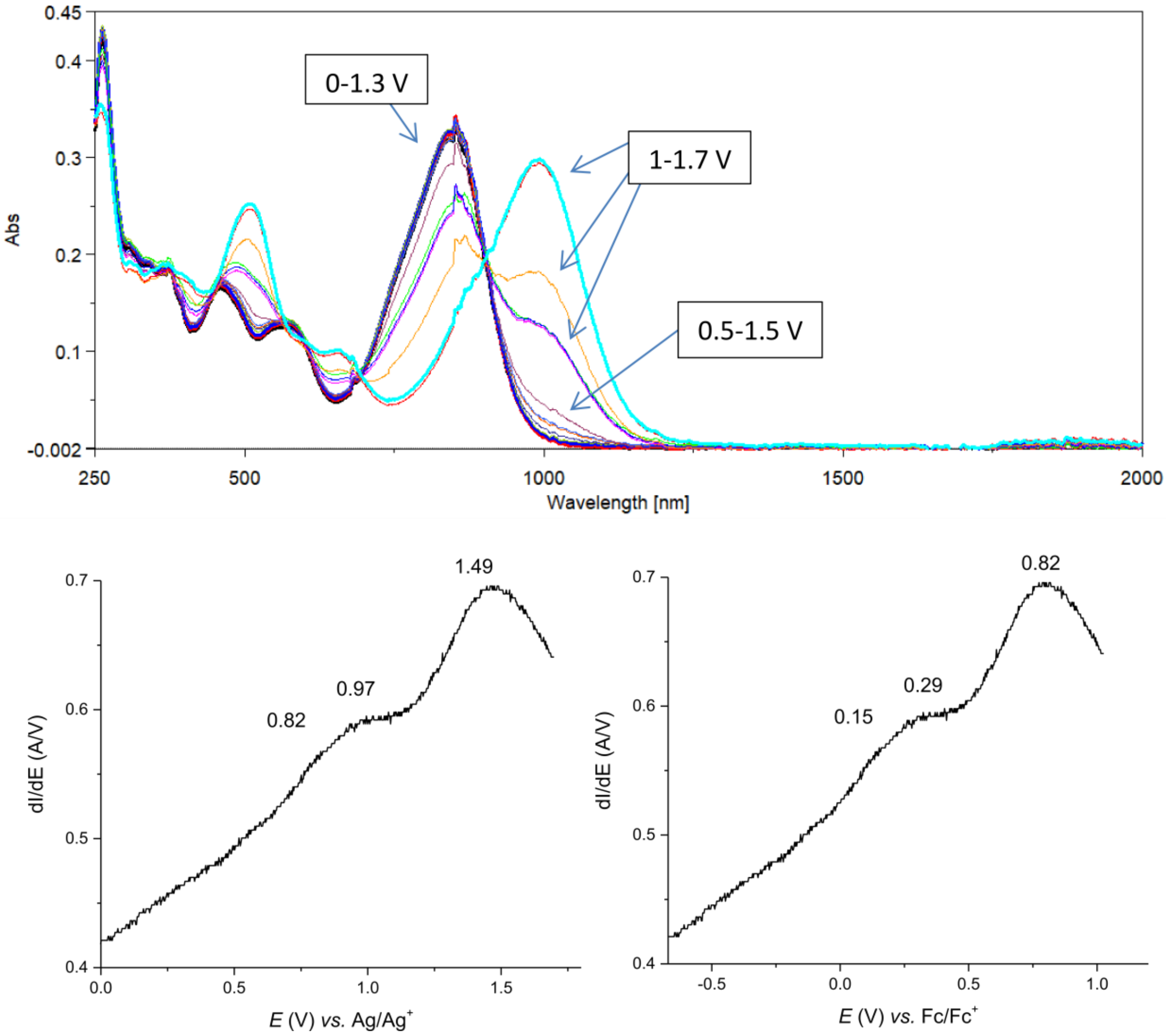

Figure S32. UV-vis-NIR spectroelectrochemistry of **2a** in in different potential setup (dichloromethane solvent, $[Bu_4N]PF_6$ as supporting electrolyte, Pt, Ag, Pt, with an increasing applied oxidation potential upto 1.7 V vs Ag/Ag^+.

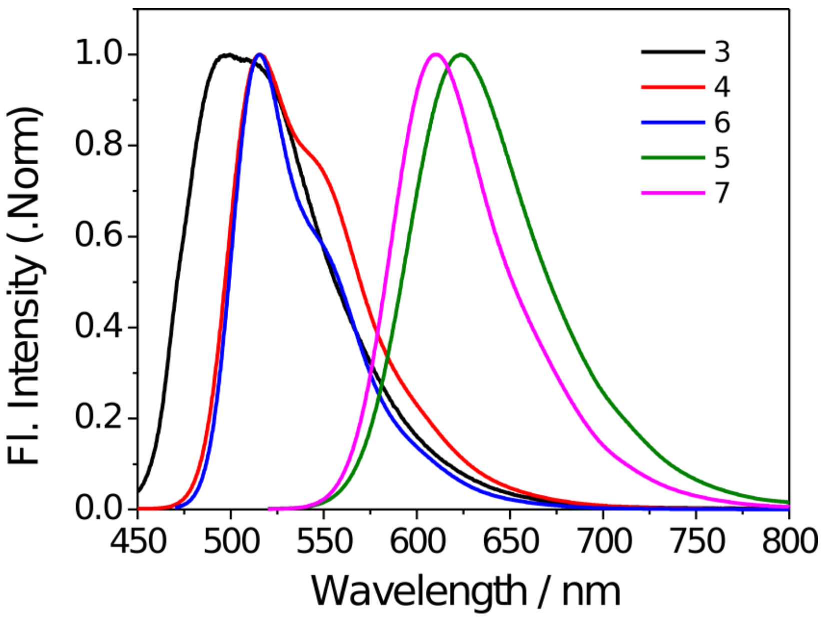

Figure S33. Emission spectra of **3-7** monopyrroles recorded in toluene.

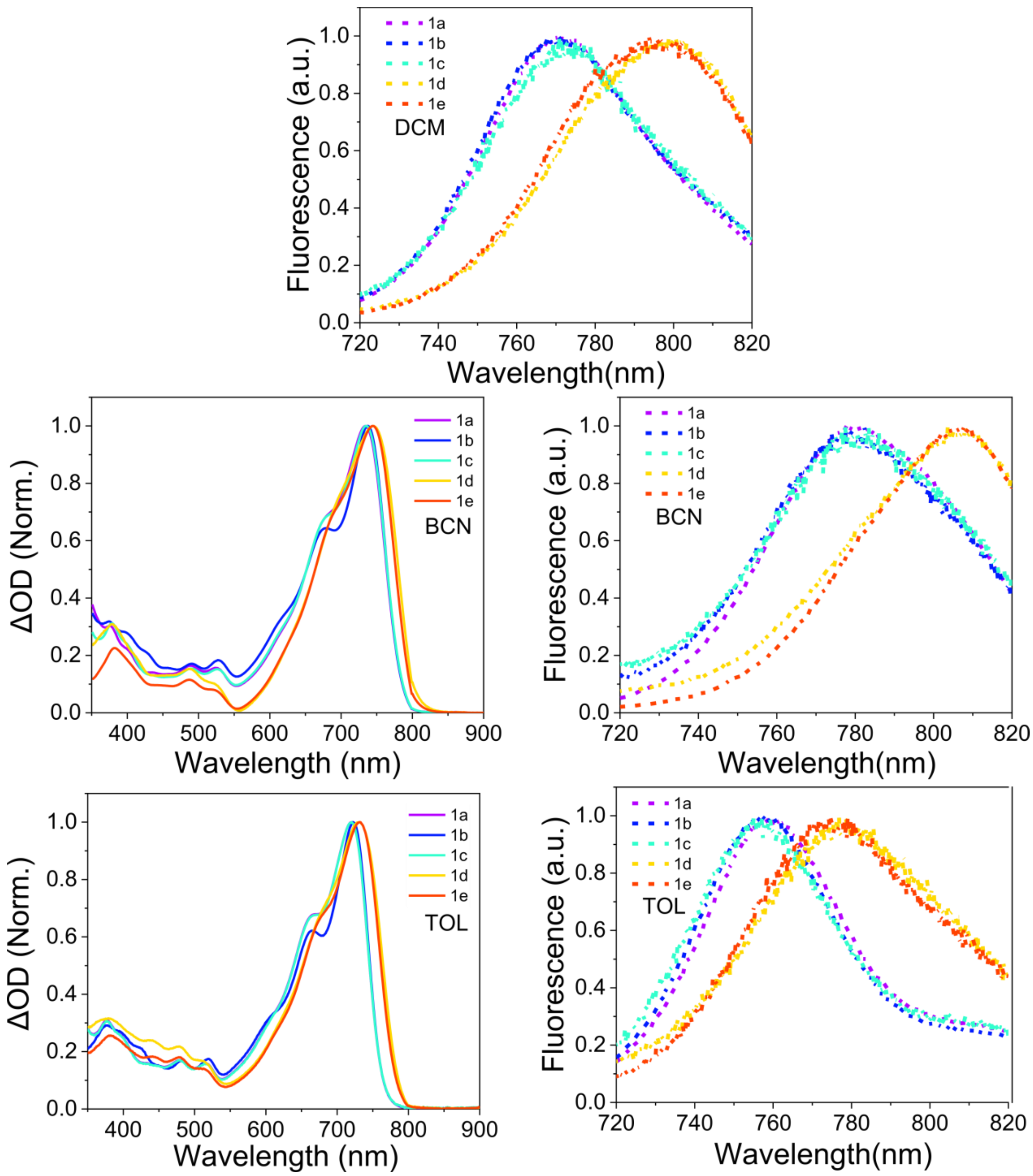

Figure S34. Absorption and emission spectra of **1a**–**1e** in different solvents (toluene, dichloromethane and benzonitrile solvent).

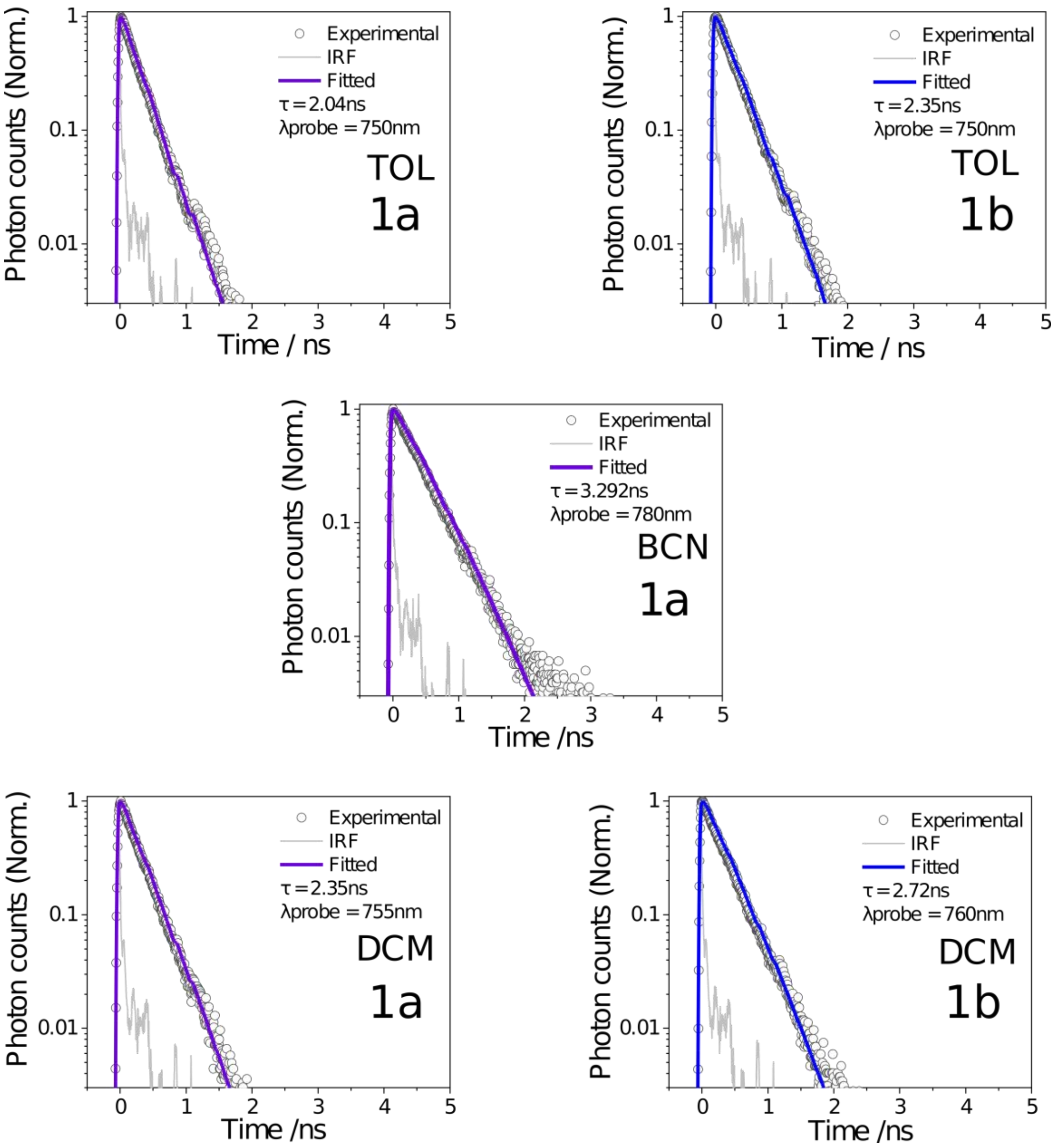

Figure S35. TCSPC data of **1a** and **1b** in toluene, dichloromethane and benzonitrile solvent.

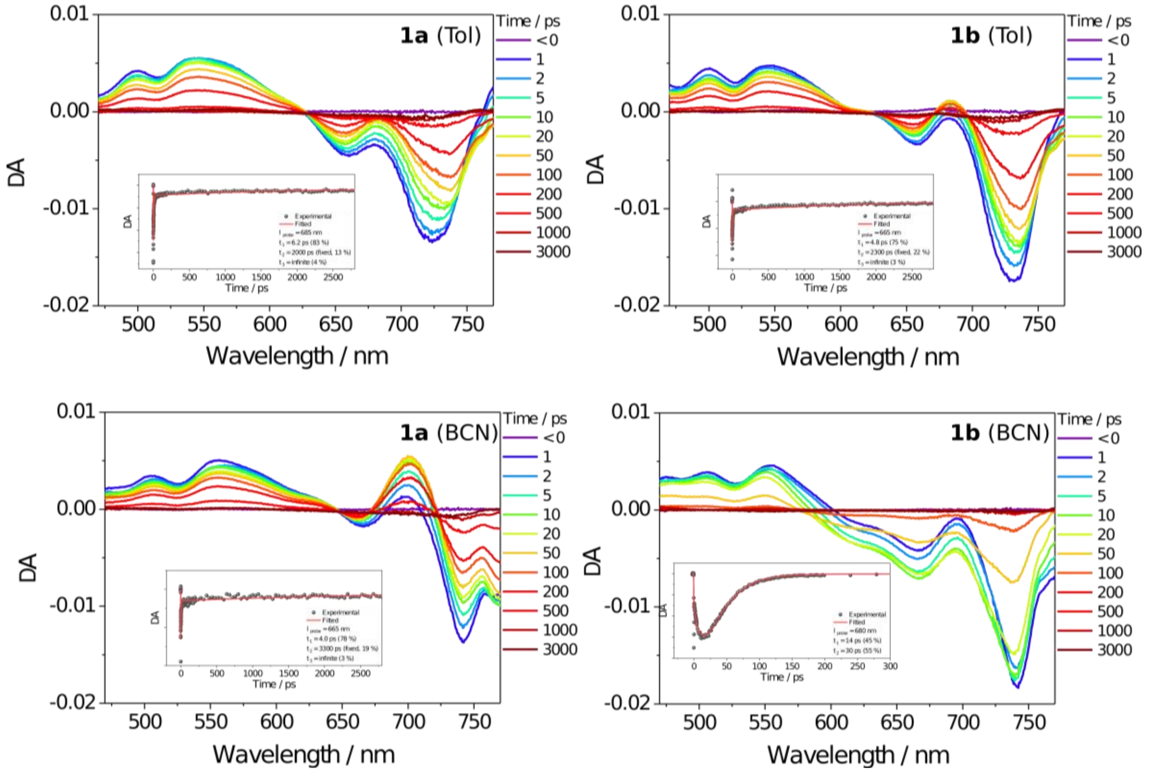

Figure S36. Transient absorption spectra of **1a** and **1b** in toluene and benzonitrile.

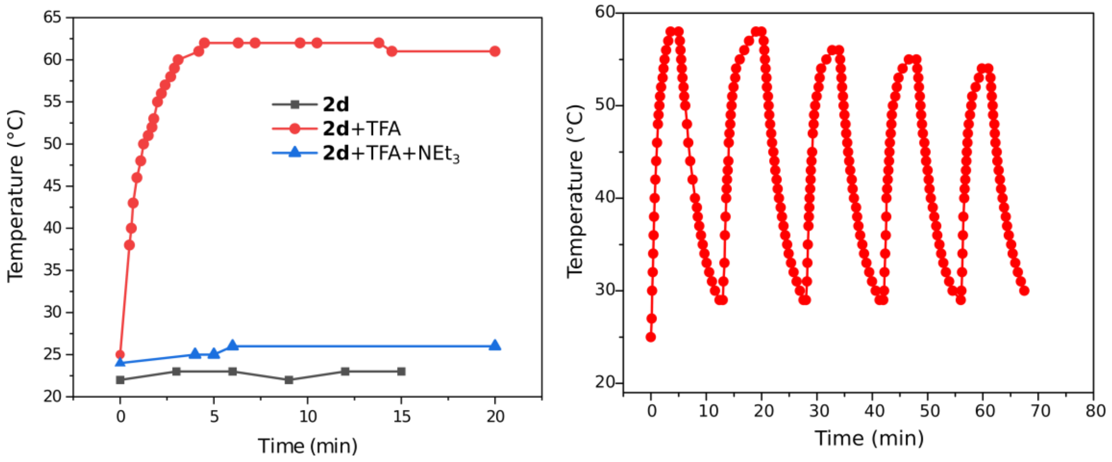

Figure S37. (A) Photothermal response of **2d** and [**2d**-H]$^+$ to irradiation with a 1064 nm laser source (16 μM, toluene in air, 25 W/cm^2). (B) Temperature variation observed for [**2d**-H]$^+$ during 5 on–off irradiation cycles.

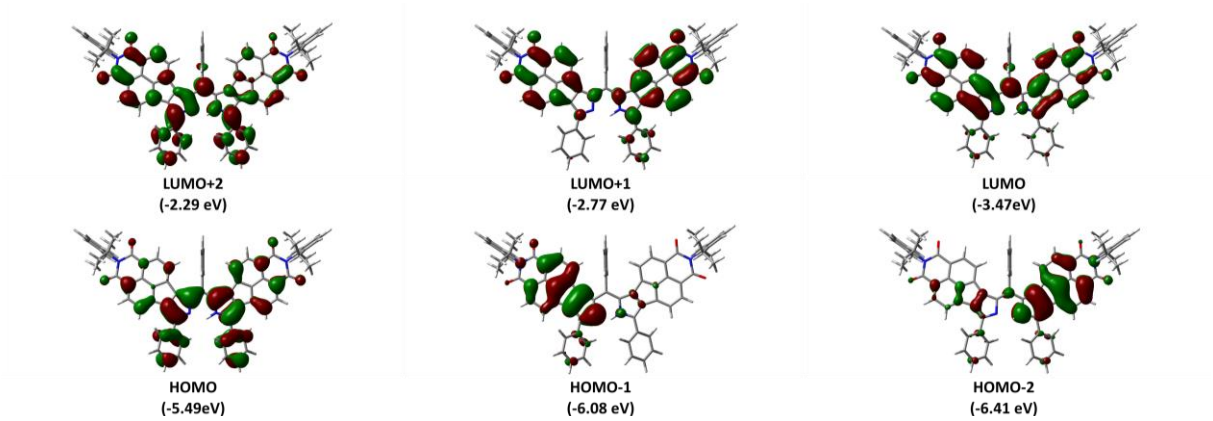

Figure S38. Kohn-Sham frontier molecular orbitals of dipyrrin **1a** calculated at the level of GD3BJ-B3LYP/6-31G(d,p) with PCM solvation (dichloromethane).

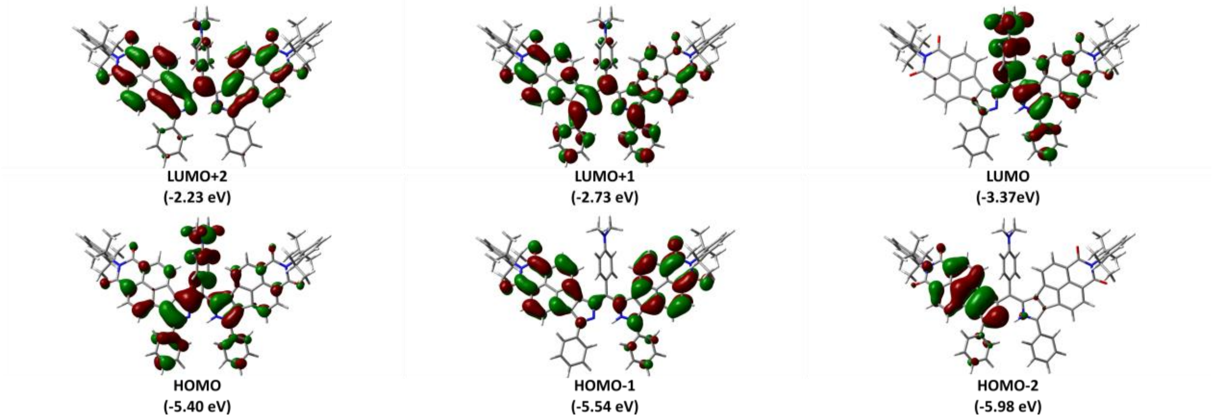

Figure S39. Kohn-Sham frontier molecular orbitals of dipyrrin **1b** calculated at the level of GD3BJ-B3LYP/6-31G(d,p) with PCM solvation (dichloromethane).

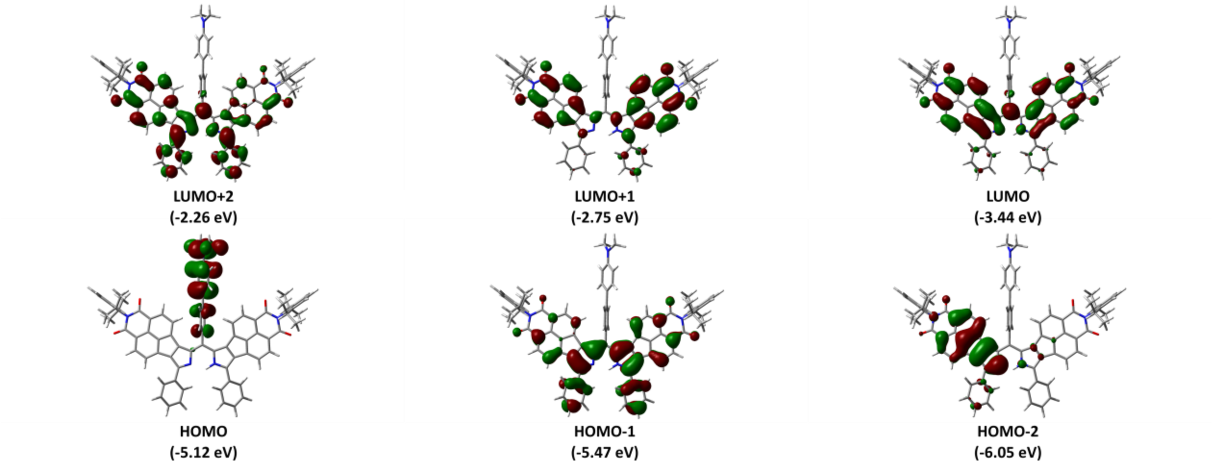

Figure S40. Kohn-Sham frontier molecular orbitals of dipyrrin **1c** calculated at the level of GD3BJ-B3LYP/6-31G(d,p) with PCM solvation (dichloromethane).

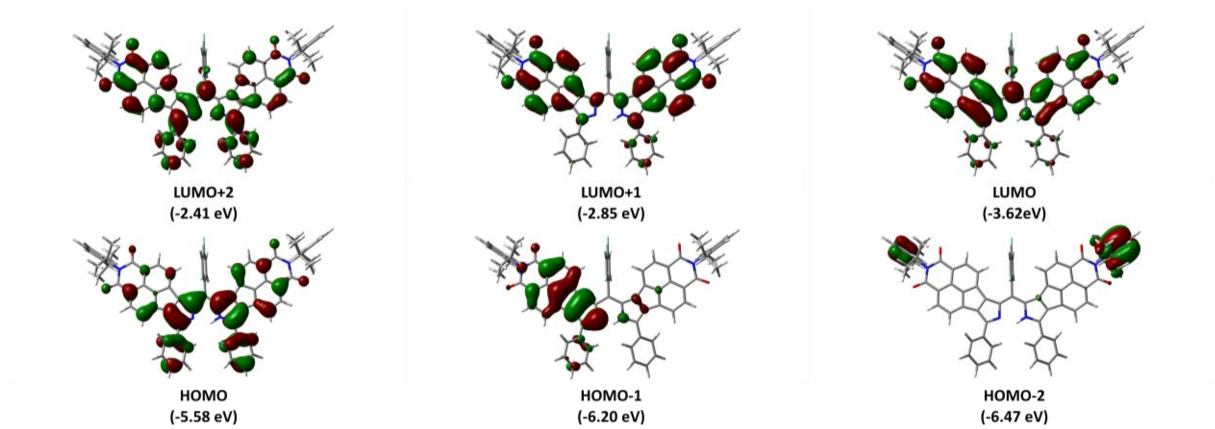

Figure S41. Kohn-Sham frontier molecular orbitals of dipyrrin **1d** calculated at the level of GD3BJ-B3LYP/6-31G(d,p) with PCM solvation (dichloromethane).

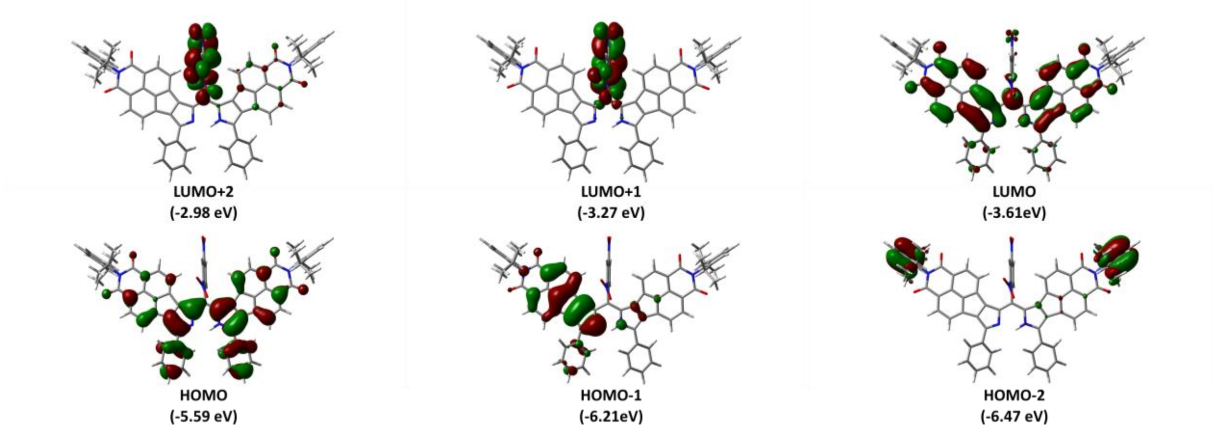

Figure S42. Kohn-Sham frontier molecular orbitals of dipyrrin **1e** calculated at the level of GD3BJ-B3LYP/6-31G(d,p) with PCM solvation (dichloromethane).

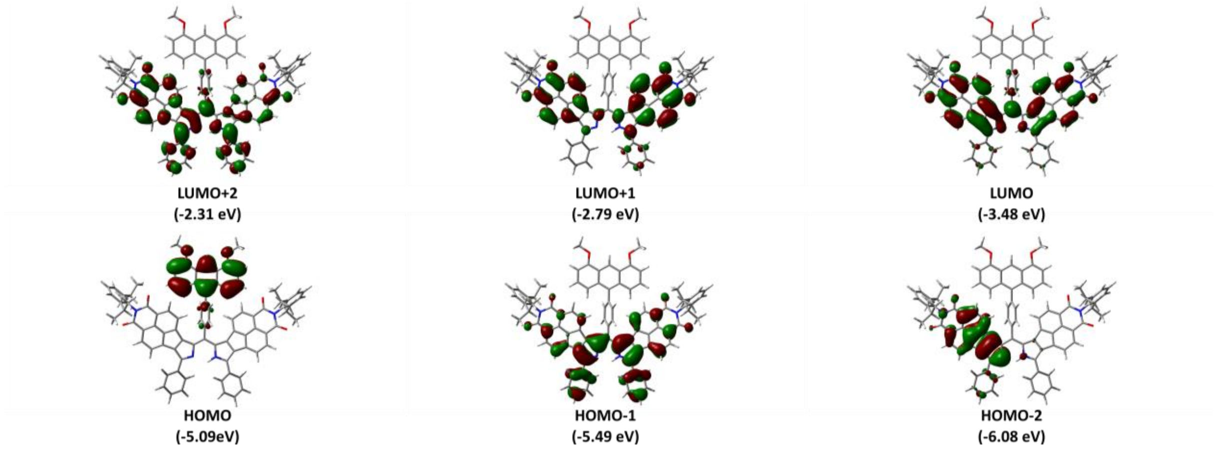

Figure S43. Kohn-Sham frontier molecular orbitals of dipyrrin **1f'** calculated at the level of GD3BJ-B3LYP/6-31G(d,p) with PCM solvation (dichloromethane).

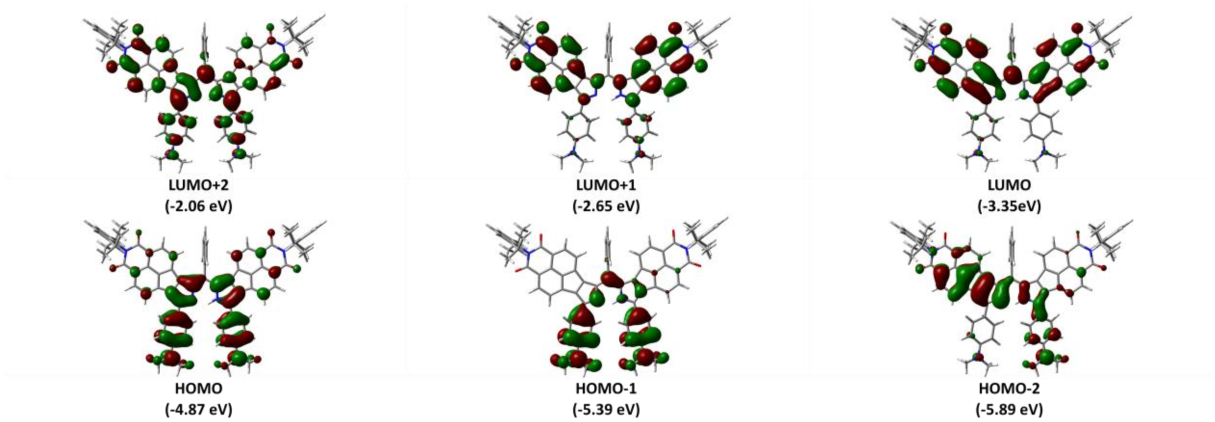

Figure S44. Kohn-Sham frontier molecular orbitals of dipyrrin **2a** calculated at the level of GD3BJ-B3LYP/6-31G(d,p) with PCM solvation (dichloromethane).

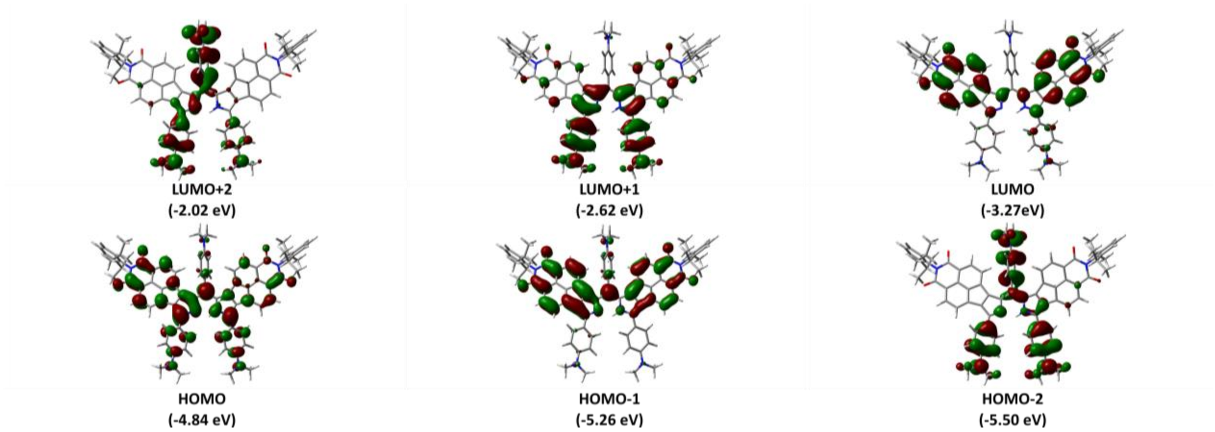

Figure S45. Kohn-Sham frontier molecular orbitals of dipyrrin **2b** calculated at the level of GD3BJ-B3LYP/6-31G(d,p) with PCM solvation (dichloromethane).

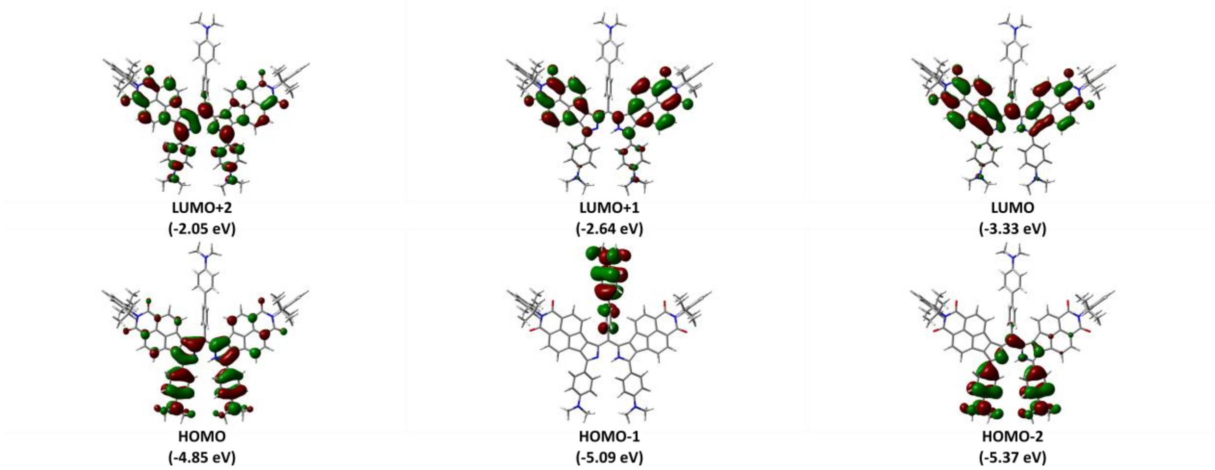

Figure S46. Kohn-Sham frontier molecular orbitals of dipyrrin **2c** calculated at the level of GD3BJ-B3LYP/6-31G(d,p) with PCM solvation (dichloromethane).

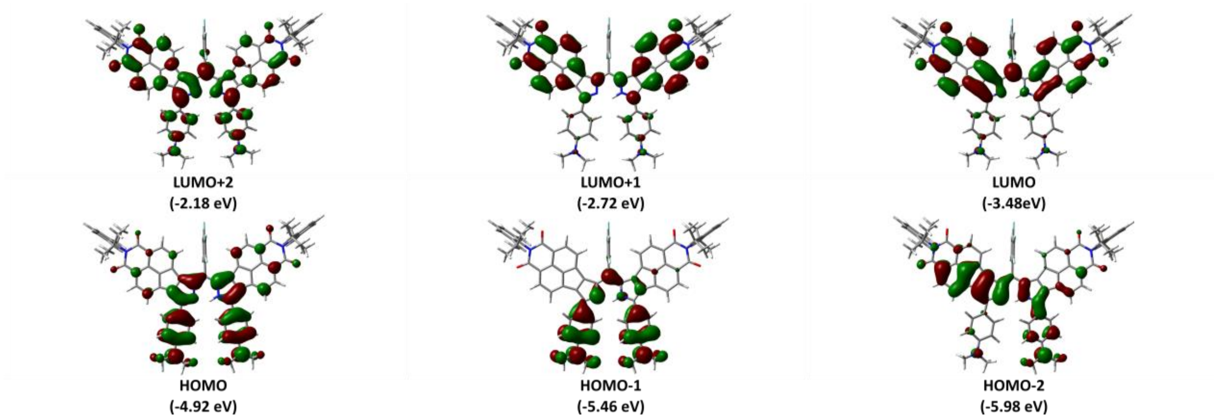

Figure S47. Kohn-Sham frontier molecular orbitals of dipyrrin **2d** calculated at the level of GD3BJ-B3LYP/6-31G(d,p) with PCM solvation (dichloromethane).

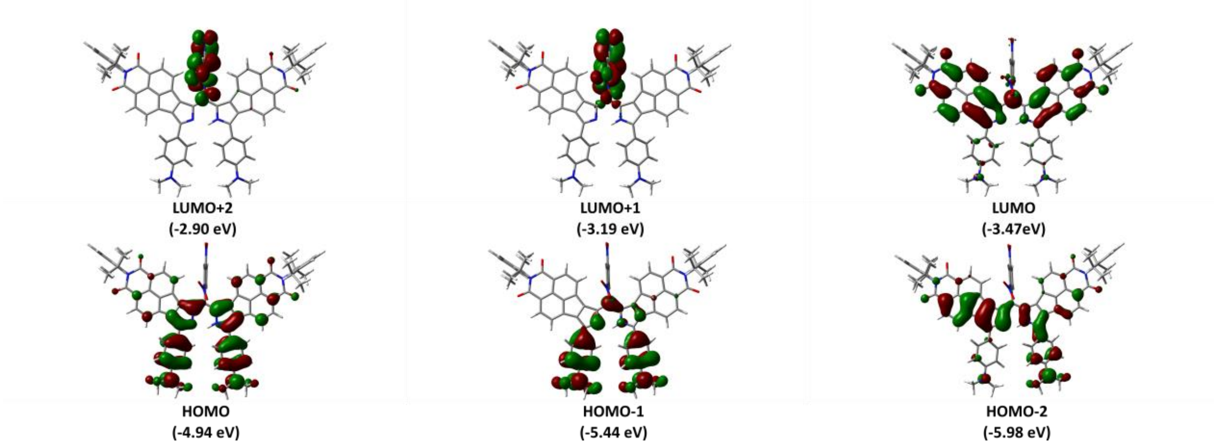

Figure S48. Kohn-Sham frontier molecular orbitals of dipyrrin **2e** calculated at the level of GD3BJ-B3LYP/6-31G(d,p) with PCM solvation (dichloromethane).

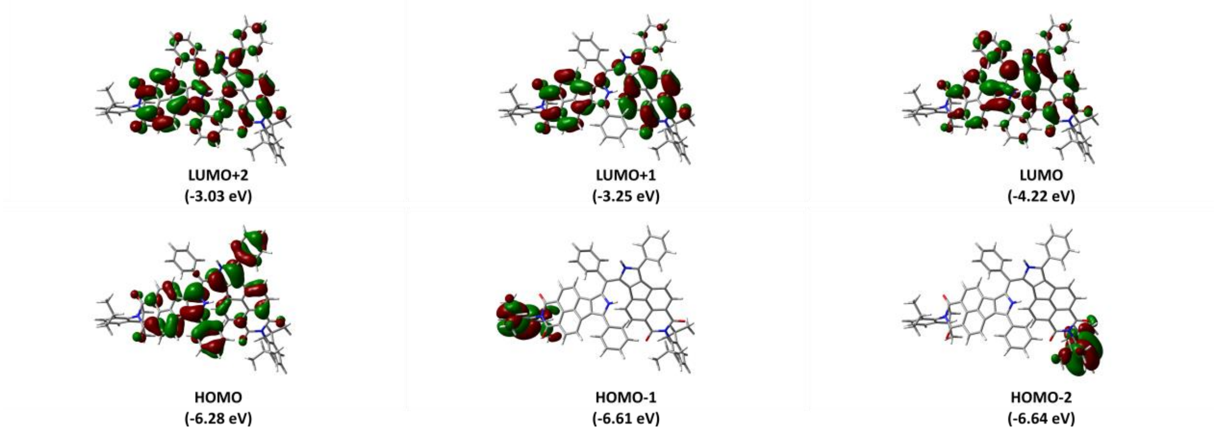

Figure S49. Kohn-Sham frontier molecular orbitals of dipyrrin **1a**–H^+ calculated at the level of GD3BJ-B3LYP/6-31G(d,p) with PCM solvation (dichloromethane).

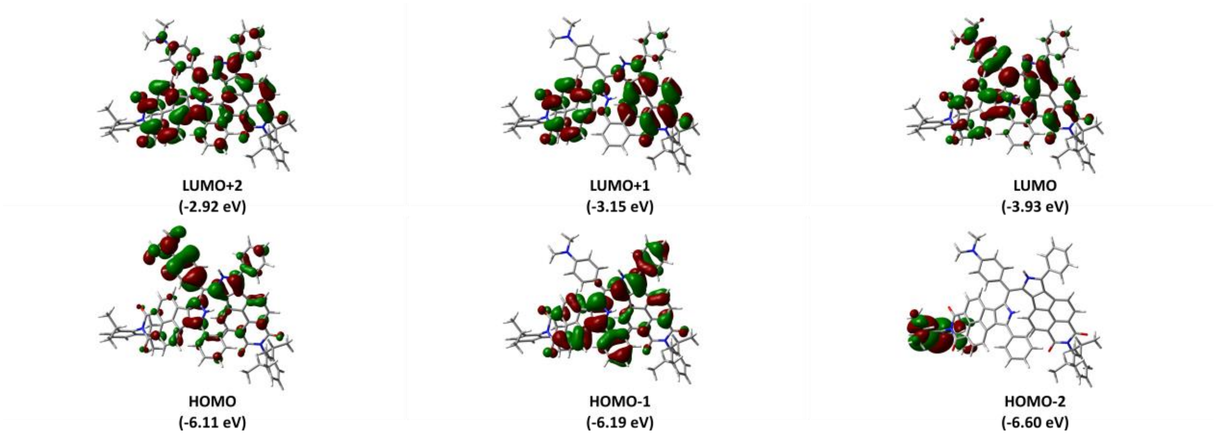

Figure S50. Kohn-Sham frontier molecular orbitals of dipyrrin **1b**–H⁺ calculated at the level of GD3BJ-B3LYP/6-31G(d,p) with PCM solvation (dichloromethane).

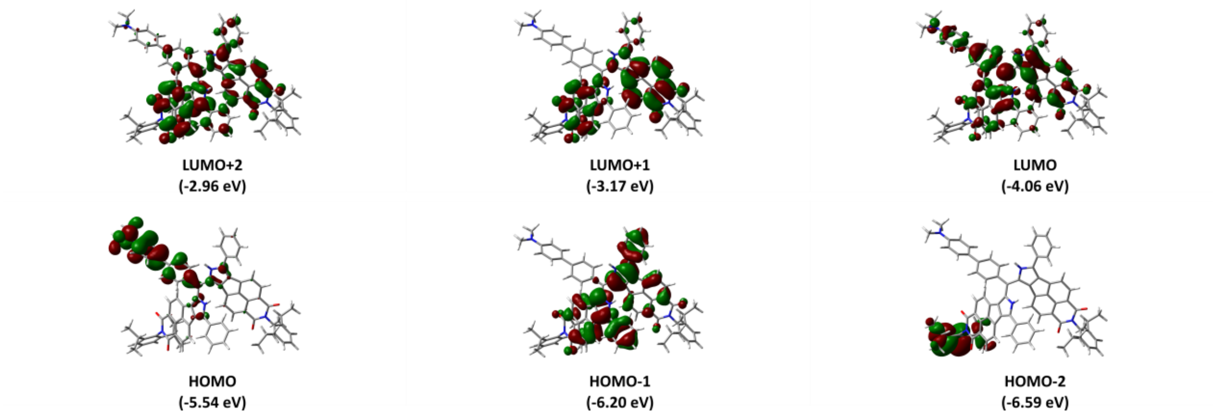

Figure S51. Kohn-Sham frontier molecular orbitals of dipyrrin **1c**–H⁺ calculated at the level of GD3BJ-B3LYP/6-31G(d,p) with PCM solvation (dichloromethane).

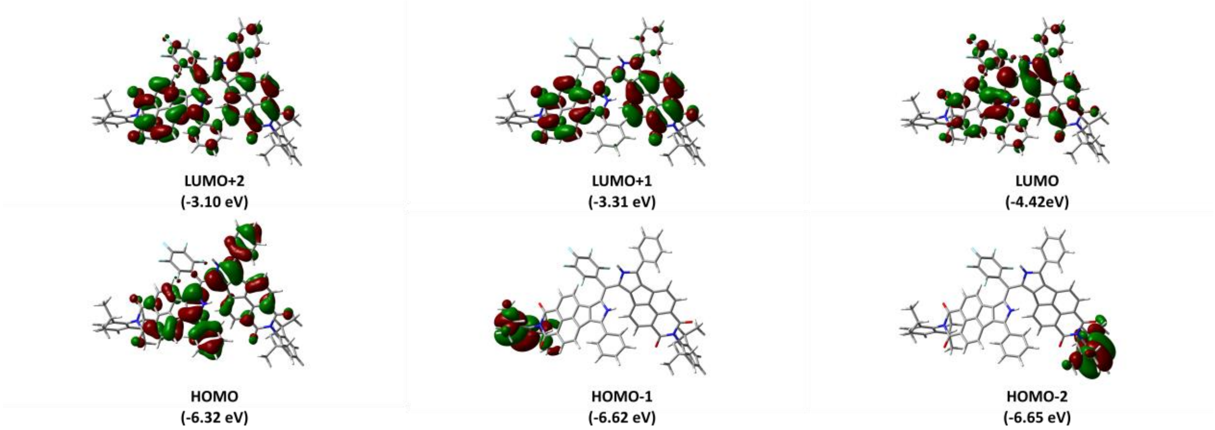

Figure S52. Kohn-Sham frontier molecular orbitals of dipyrrin **1d**–H⁺ calculated at the level of GD3BJ-B3LYP/6-31G(d,p) with PCM solvation (dichloromethane).

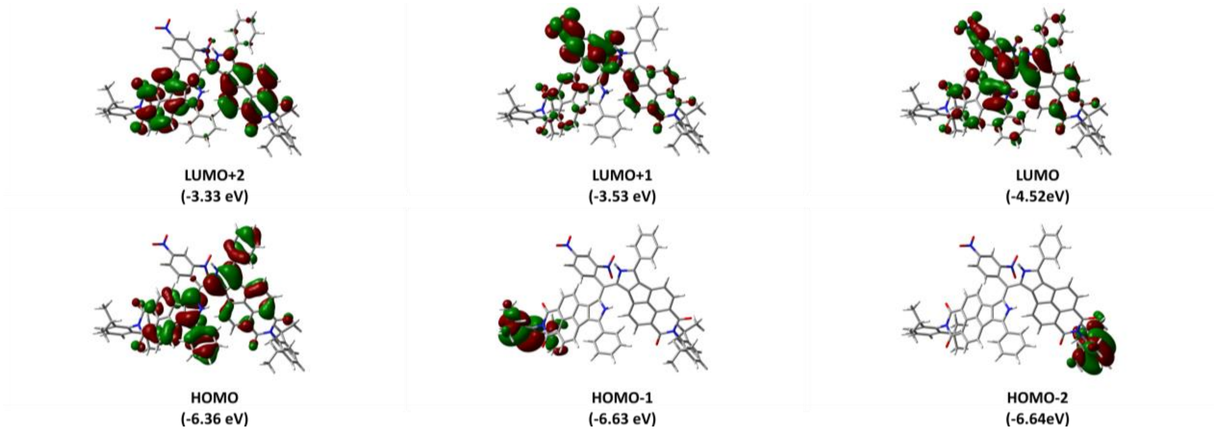

Figure S53. Kohn-Sham frontier molecular orbitals of dipyrrin **1e**–H^+ calculated at the level of GD3BJ-B3LYP/6-31G(d,p) with PCM solvation (dichloromethane).

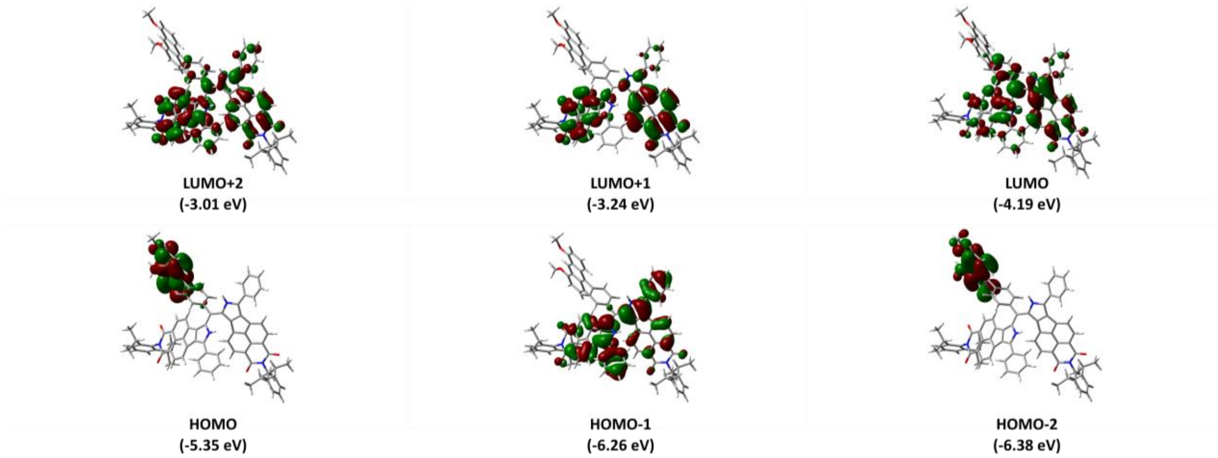

Figure S54. Kohn-Sham frontier molecular orbitals of dipyrrin **1f'**–H^+ calculated at the level of GD3BJ-B3LYP/6-31G(d,p) with PCM solvation (dichloromethane).

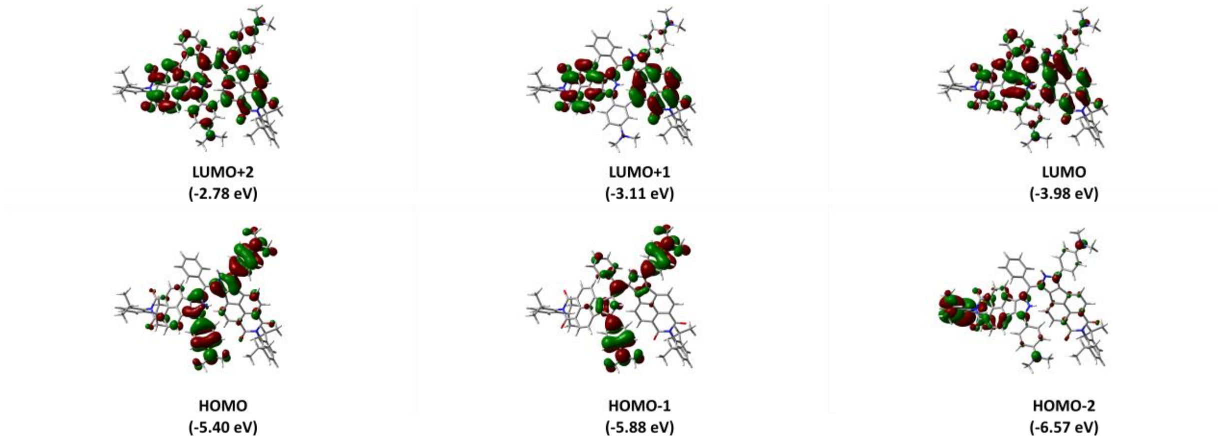

Figure S55. Kohn-Sham frontier molecular orbitals of dipyrrin **2a**–H^+ calculated at the level of GD3BJ-B3LYP/6-31G(d,p) with PCM solvation (dichloromethane).

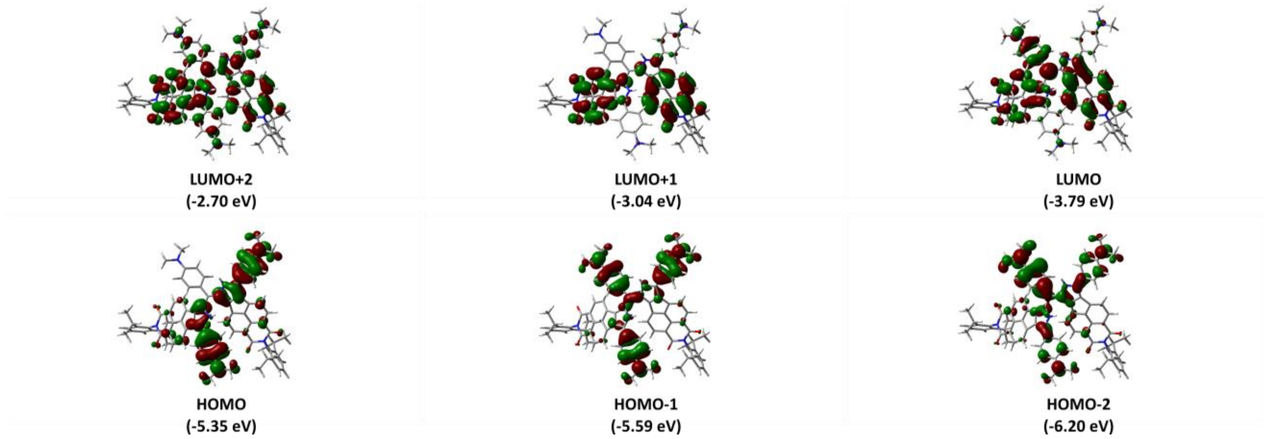

Figure S56. Kohn-Sham frontier molecular orbitals of dipyrrin **2b**–H$^+$ calculated at the level of GD3BJ-B3LYP/6-31G(d,p) with PCM solvation (dichloromethane).

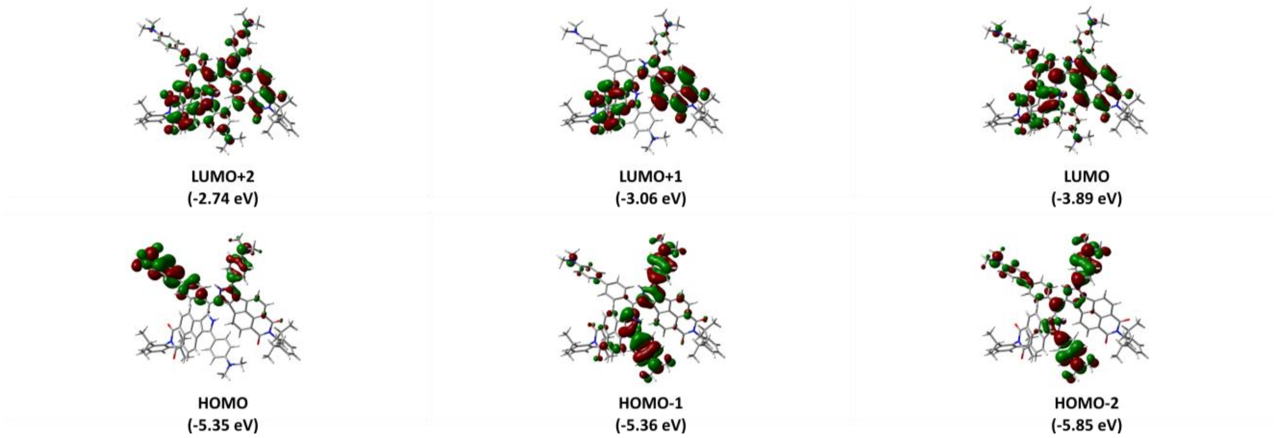

Figure S57. Kohn-Sham frontier molecular orbitals of dipyrrin **2c**–H$^+$ calculated at the level of GD3BJ-B3LYP/6-31G(d,p) with PCM solvation (dichloromethane).

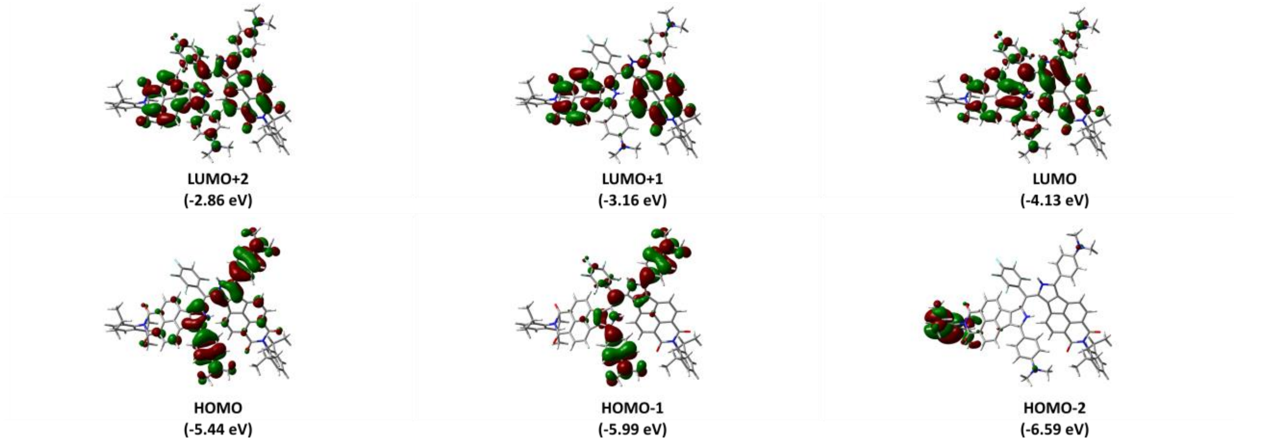

Figure S58. Kohn-Sham frontier molecular orbitals of dipyrrin **2d**–H$^+$ calculated at the level of GD3BJ-B3LYP/6-31G(d,p) with PCM solvation (dichloromethane).

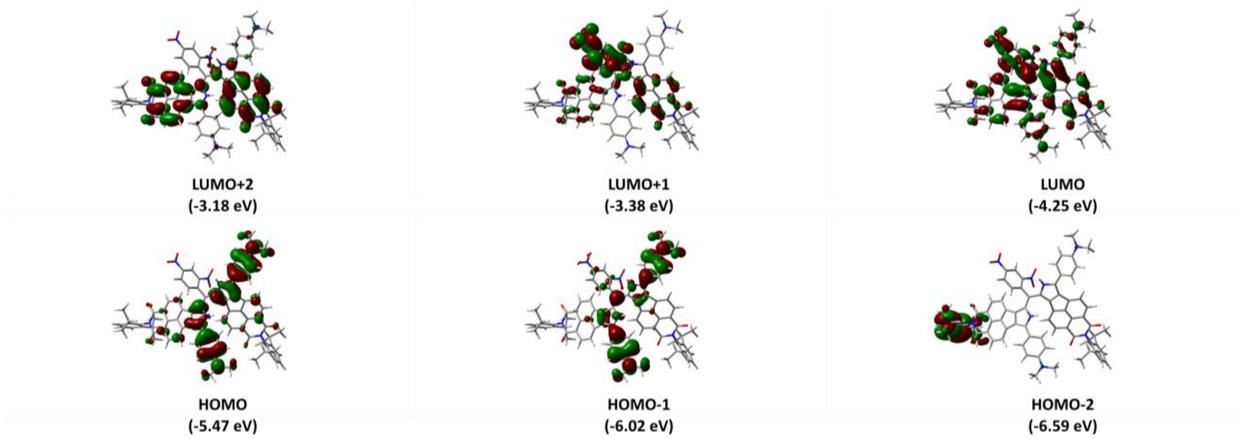

Figure S59. Kohn-Sham frontier molecular orbitals of dipyrrin **2e**–H^+ calculated at the level of GD3BJ-B3LYP/6-31G(d,p) with PCM solvation (dichloromethane).

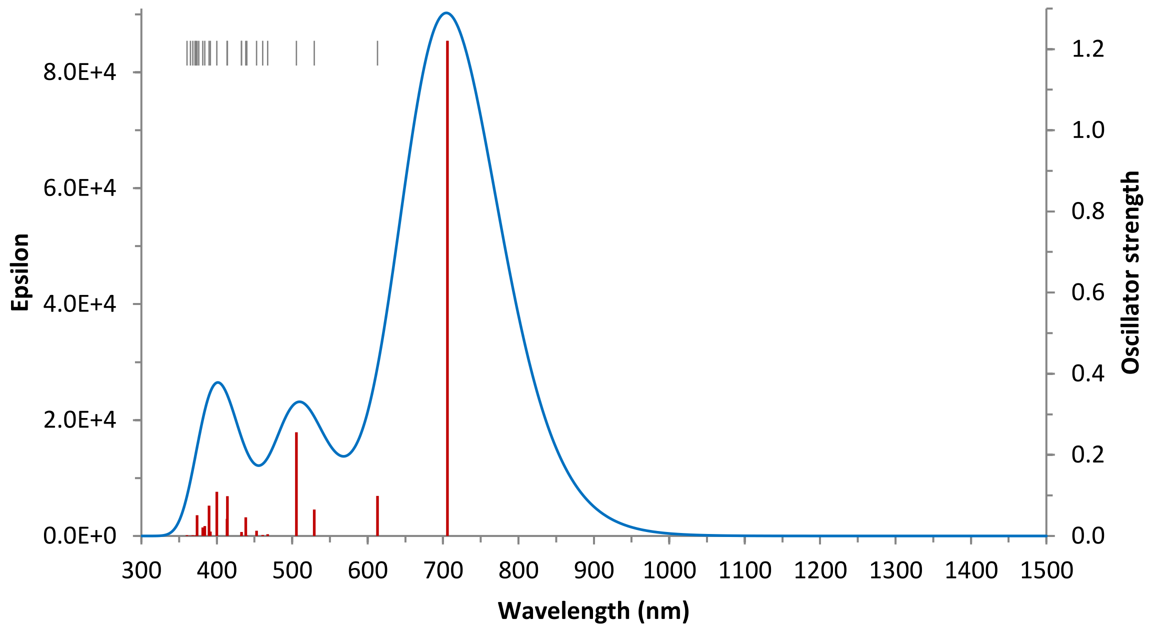

Figure S60. Simulated electronic absorption spectrum of **1a** (TD/PCM(dichloromethane)GD3BJ-B3LYP/6-31G(d,p)).

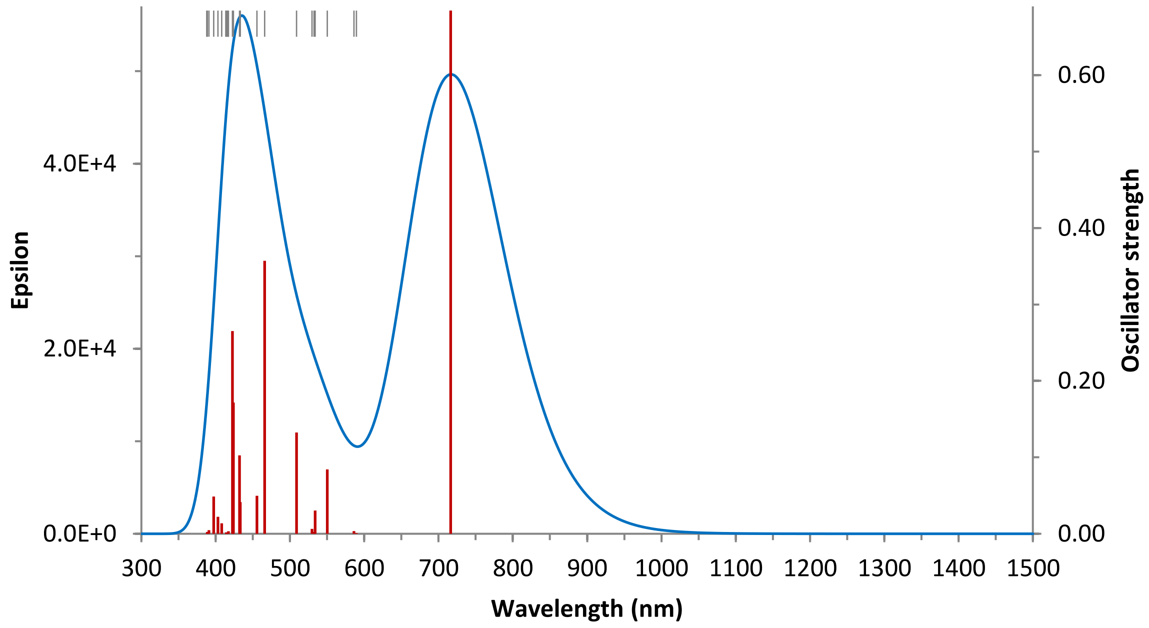

Figure S61. Simulated electronic absorption spectrum of **1a**–H$^+$ (TD/PCM(dichloromethane)GD3BJ-B3LYP/6-31G(d,p)).

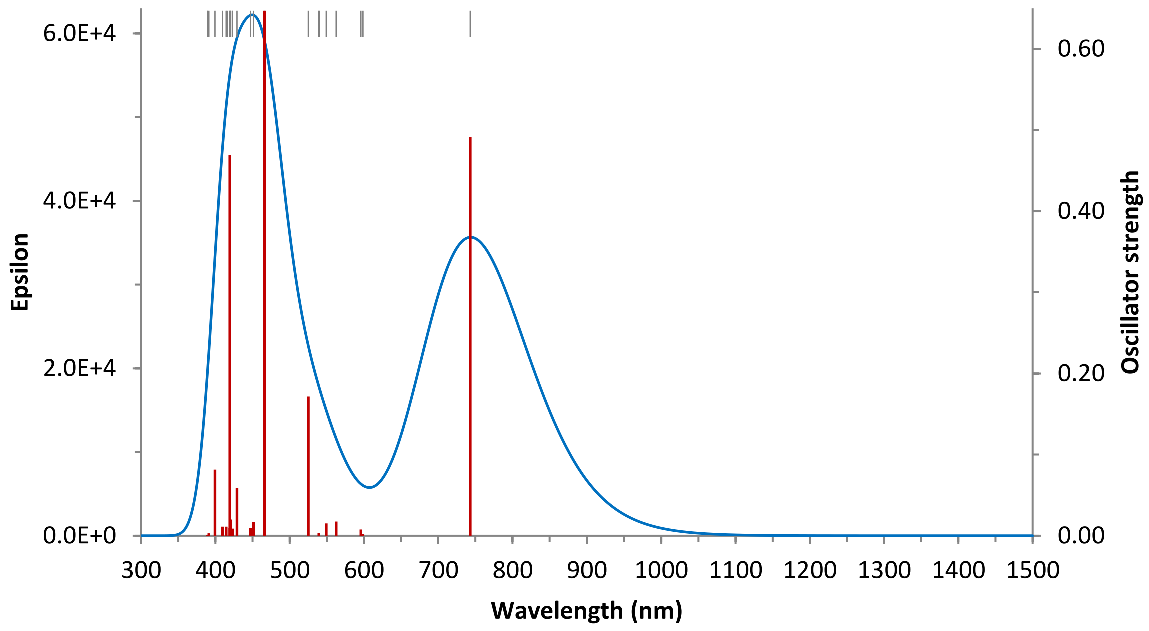

Figure S62. Simulated electronic absorption spectrum of **1a**–H+ (*E-anti*) (TD/PCM(dichloromethane)GD3BJ-B3LYP/6-31G(d,p)).

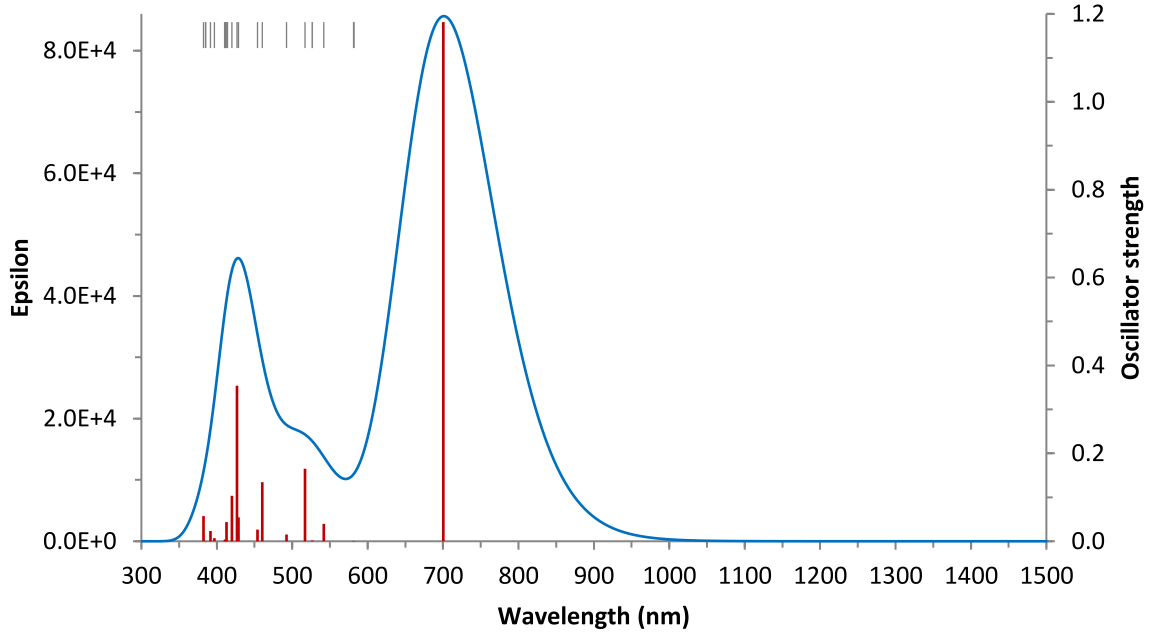

Figure S63. Simulated electronic absorption spectrum of **1a**–H+ (*Z-syn*) (TD/PCM(dichloromethane)GD3BJ-B3LYP/6-31G(d,p)).

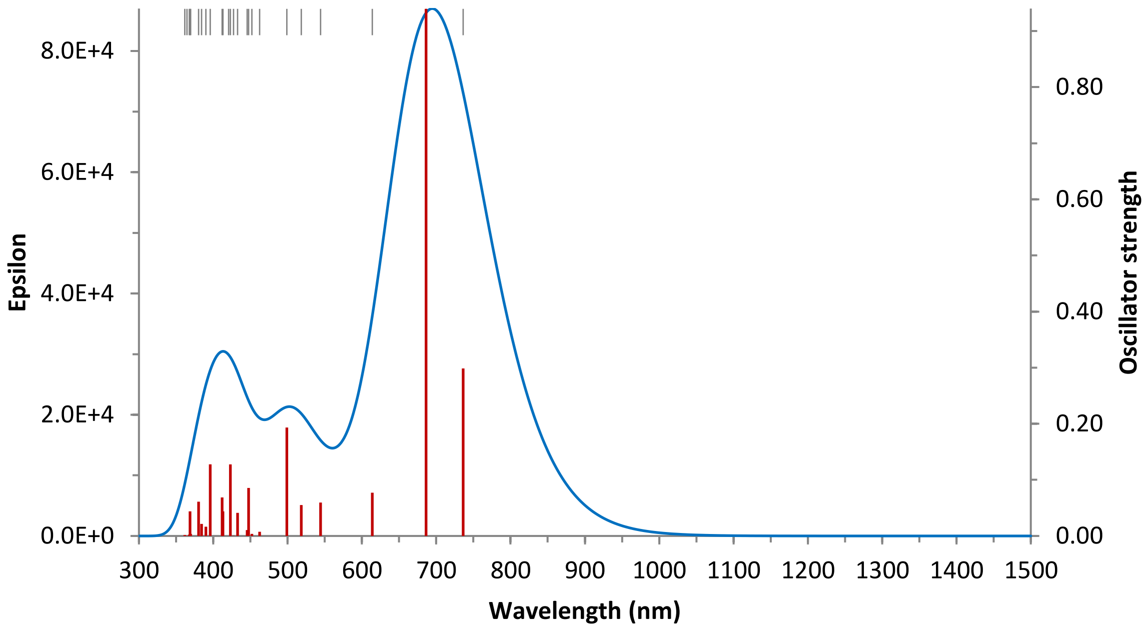

Figure S64. Simulated electronic absorption spectrum of **1b** (TD/PCM(dichloromethane)GD3BJ-B3LYP/6-31G(d,p)).

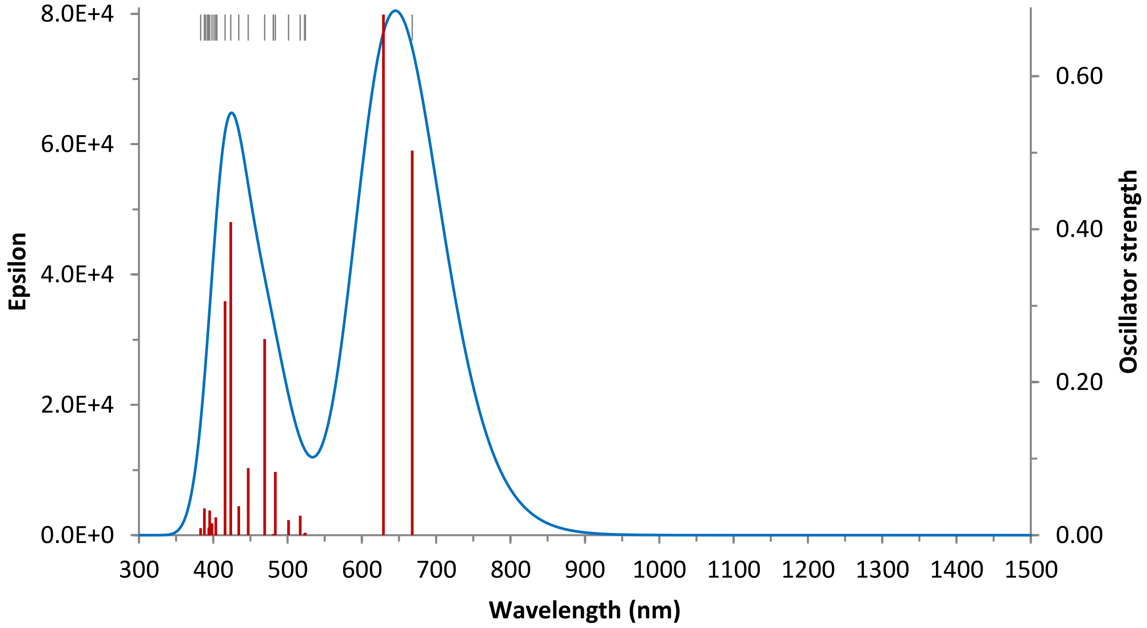

Figure S65. Simulated electronic absorption spectrum of **1b**–H^+ (TD/PCM(dichloromethane)GD3BJ-B3LYP/6-31G(d,p)).

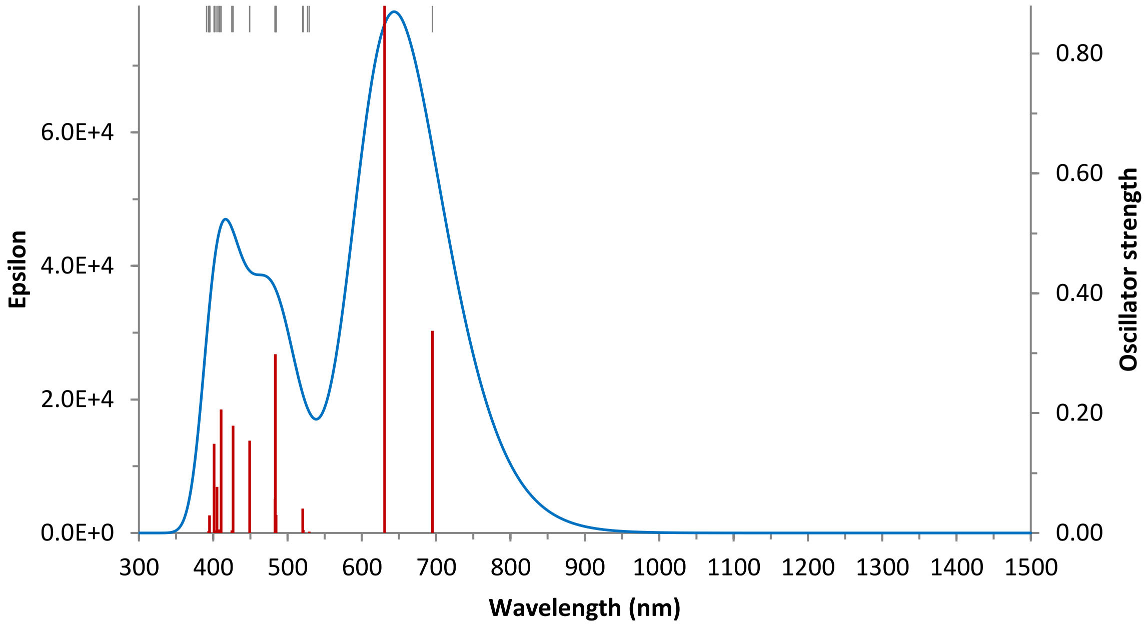

Figure S66. Simulated electronic absorption spectrum of **1b**–H⁺ (*E-anti*) (TD/PCM(dichloromethane)GD3BJ-B3LYP/6-31G(d,p)).

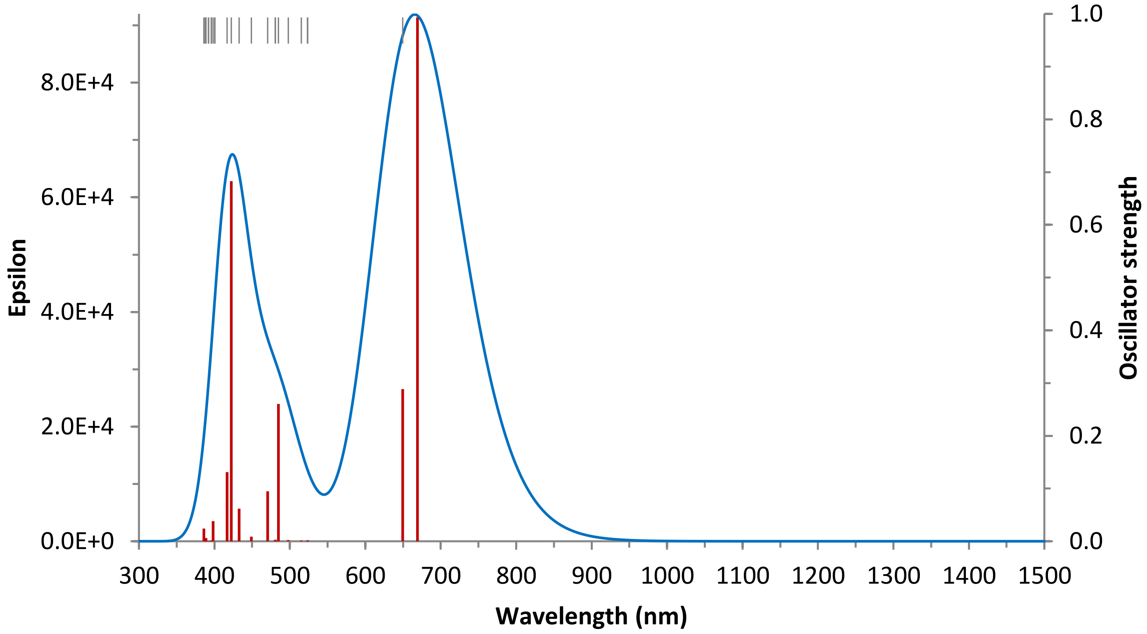

Figure S67. Simulated electronic absorption spectrum of **1b**–H⁺ (*Z-syn*) (TD/PCM(dichloromethane)GD3BJ-B3LYP/6-31G(d,p)).

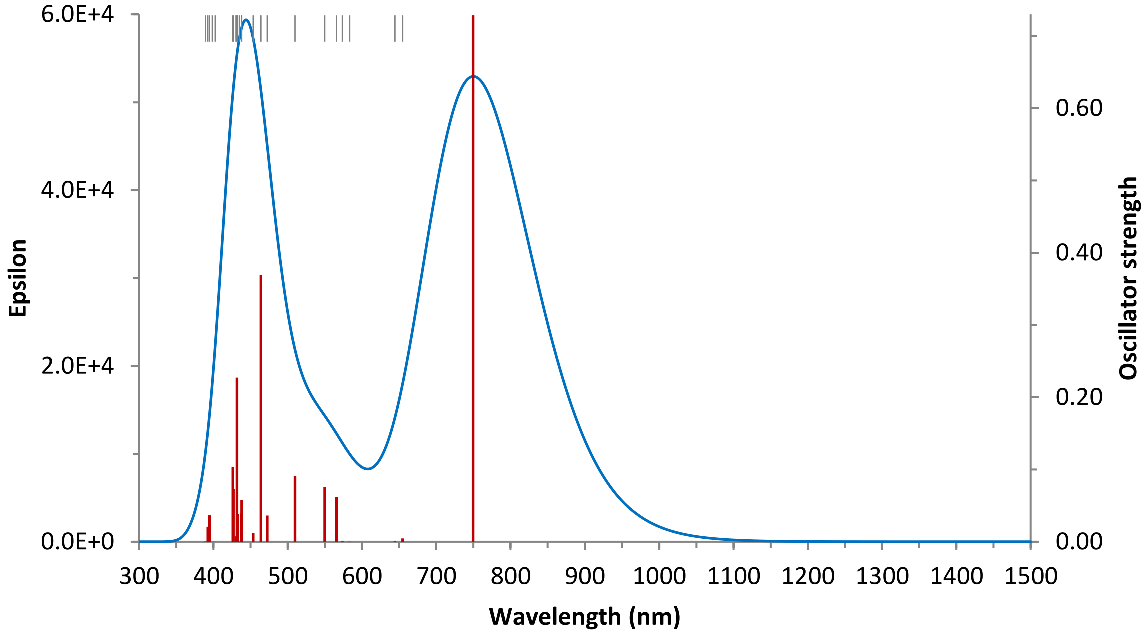

Figure S68. Simulated electronic absorption spectrum of **1b**–$2H^{2+}$ (TD/PCM(dichloromethane)GD3BJ-B3LYP/6-31G(d,p)).

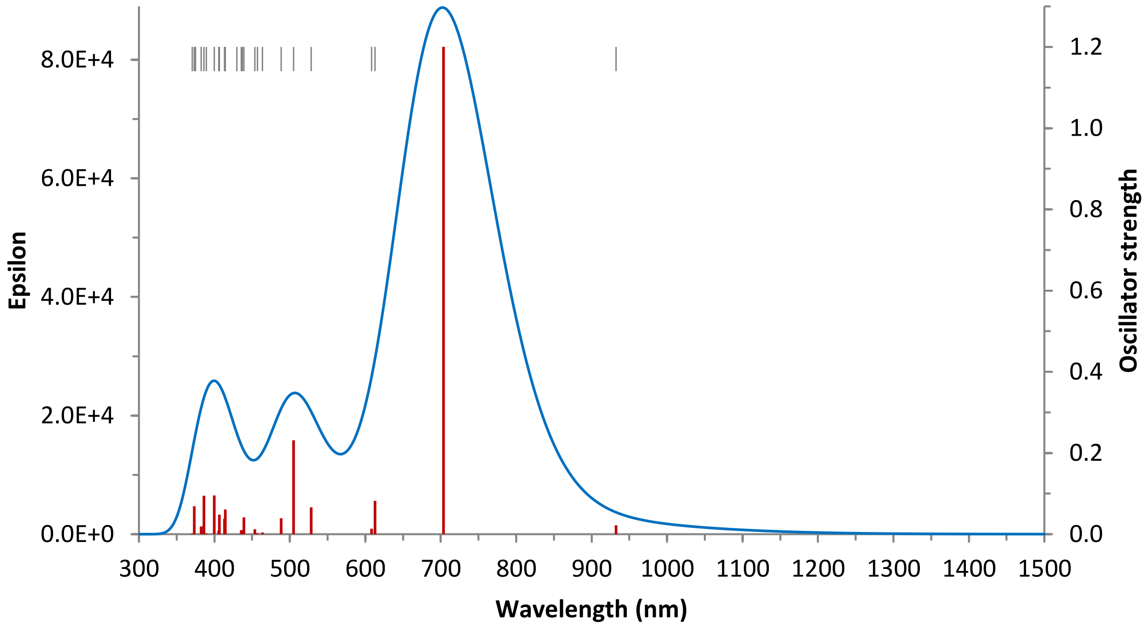

Figure S69. Simulated electronic absorption spectrum of **1c** (TD/PCM(dichloromethane)GD3BJ-B3LYP/6-31G(d,p)).

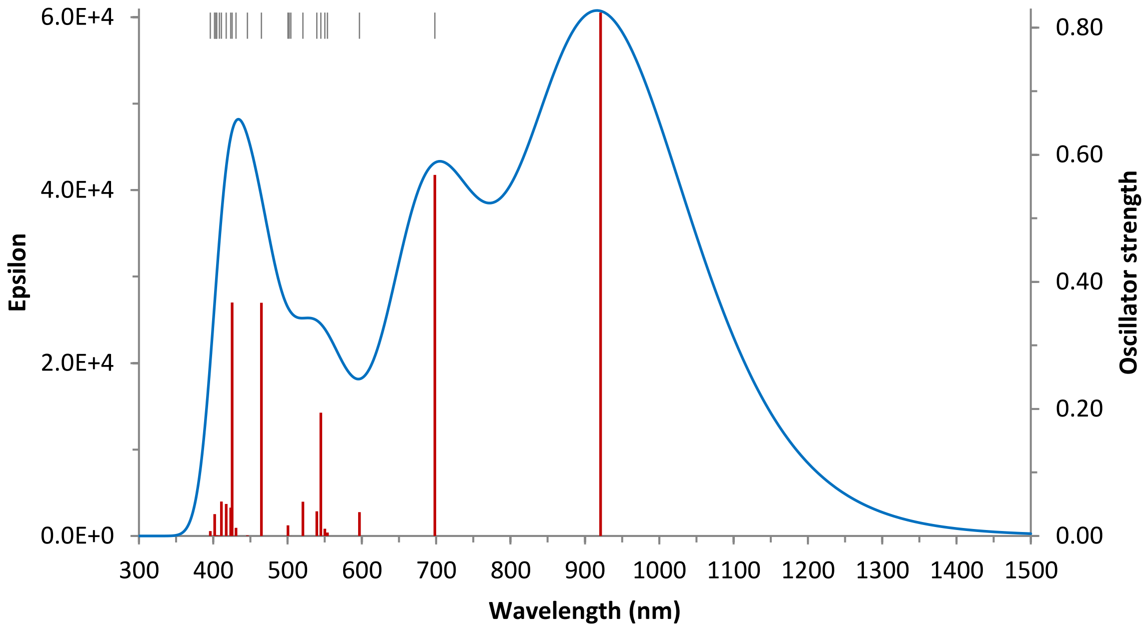

Figure S70. Simulated electronic absorption spectrum of **1c**–H^{+} (TD/PCM(dichloromethane)GD3BJ-B3LYP/6-31G(d,p)).

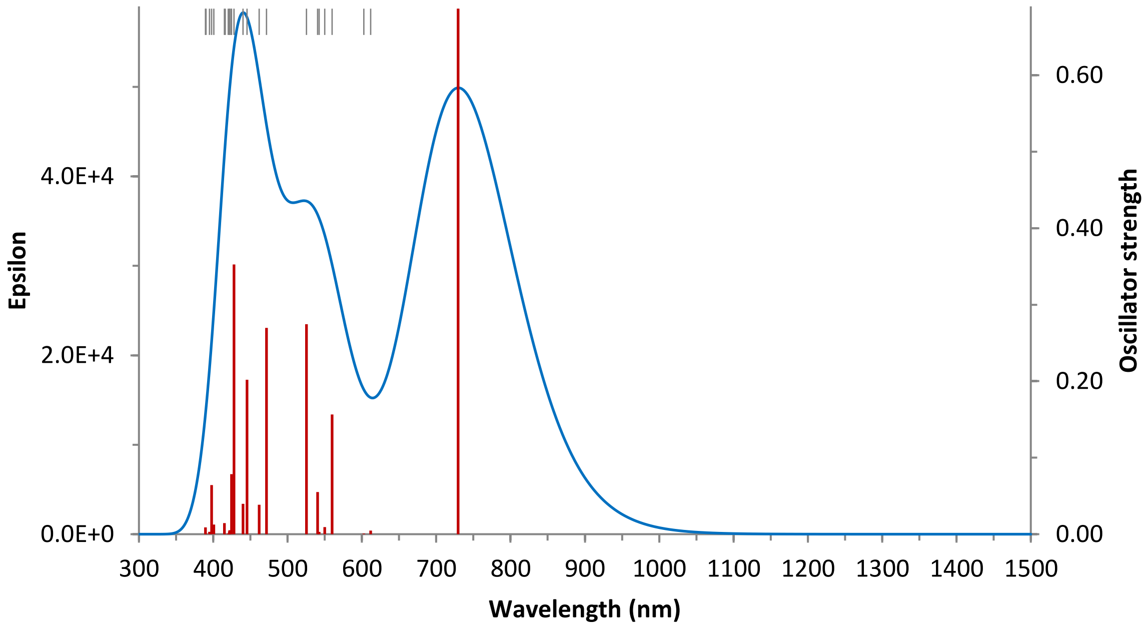

Figure S71. Simulated electronic absorption spectrum of **1c**–$2H^{2+}$ (TD/PCM(dichloromethane)GD3BJ-B3LYP/6-31G(d,p)).

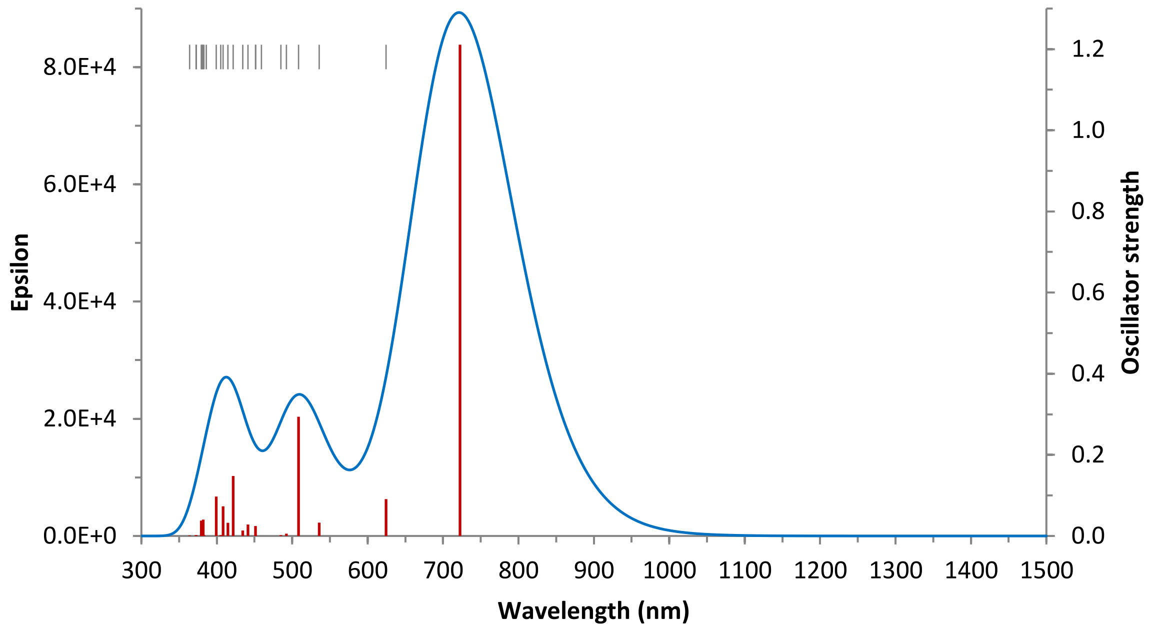

Figure S72. Simulated electronic absorption spectrum of **1d** (TD/PCM(dichloromethane)GD3BJ-B3LYP/6-31G(d,p)).

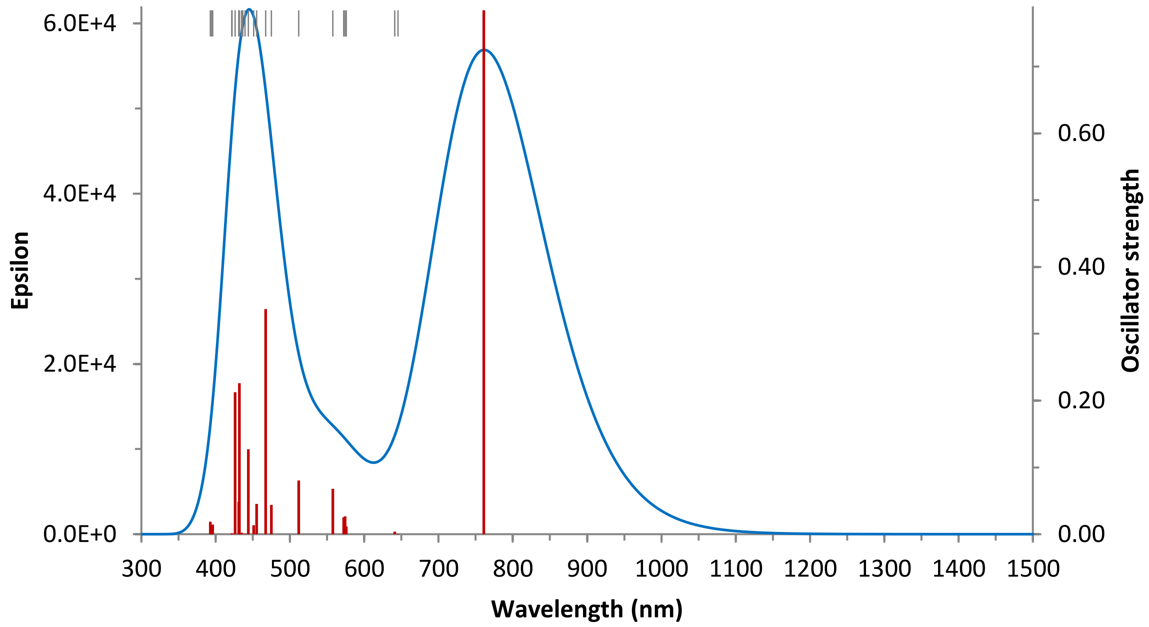

Figure S73. Simulated electronic absorption spectrum of **1d**–H⁺ (TD/PCM(dichloromethane)GD3BJ-B3LYP/6-31G(d,p)).

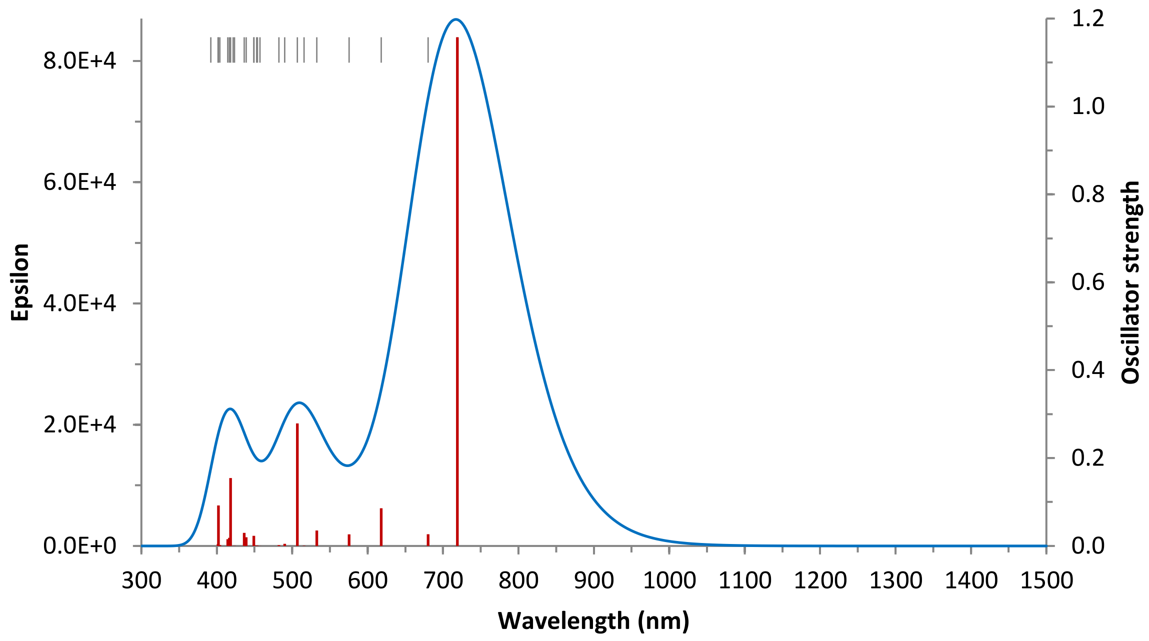

Figure S74. Simulated electronic absorption spectrum of **1e** (TD/PCM(dichloromethane)GD3BJ-B3LYP/6-31G(d,p)).

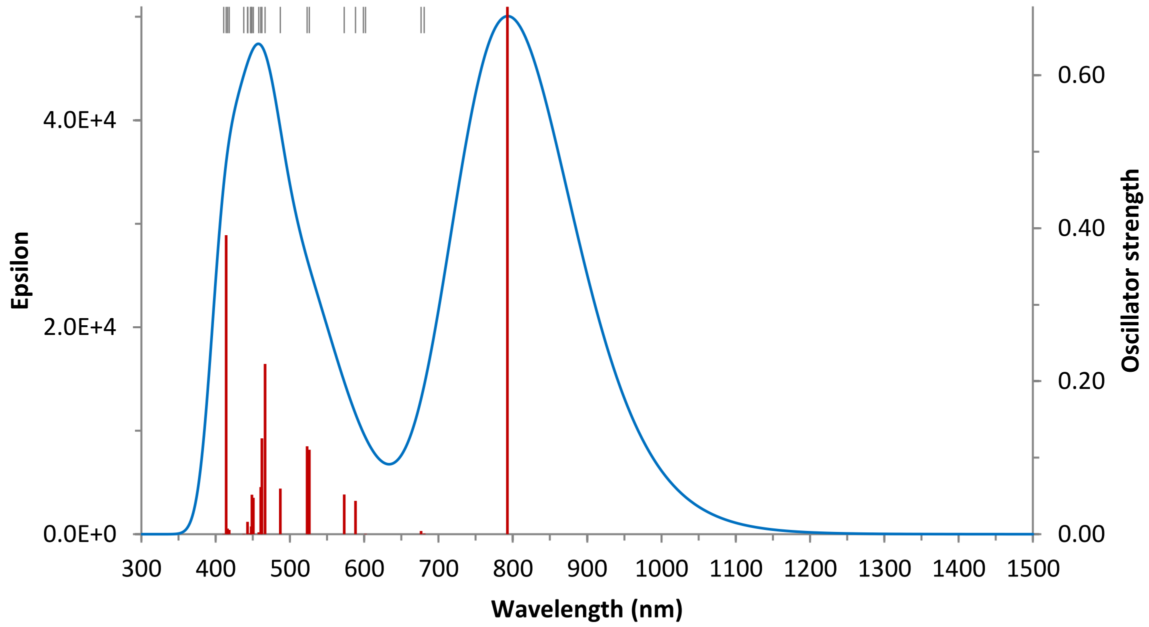

Figure S75. Simulated electronic absorption spectrum of **1e**–H^+ (TD/PCM(dichloromethane)GD3BJ-B3LYP/6-31G(d,p)).

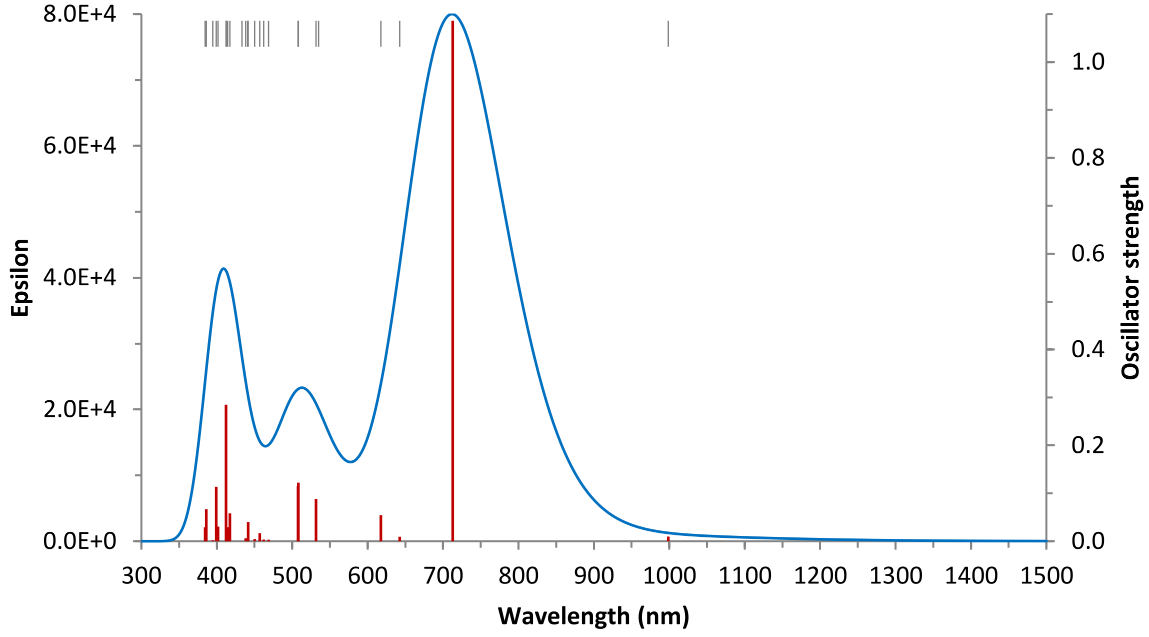

Figure S76. Simulated electronic absorption spectrum of **1f'** (TD/PCM(dichloromethane)GD3BJ-B3LYP/6-31G(d,p)).

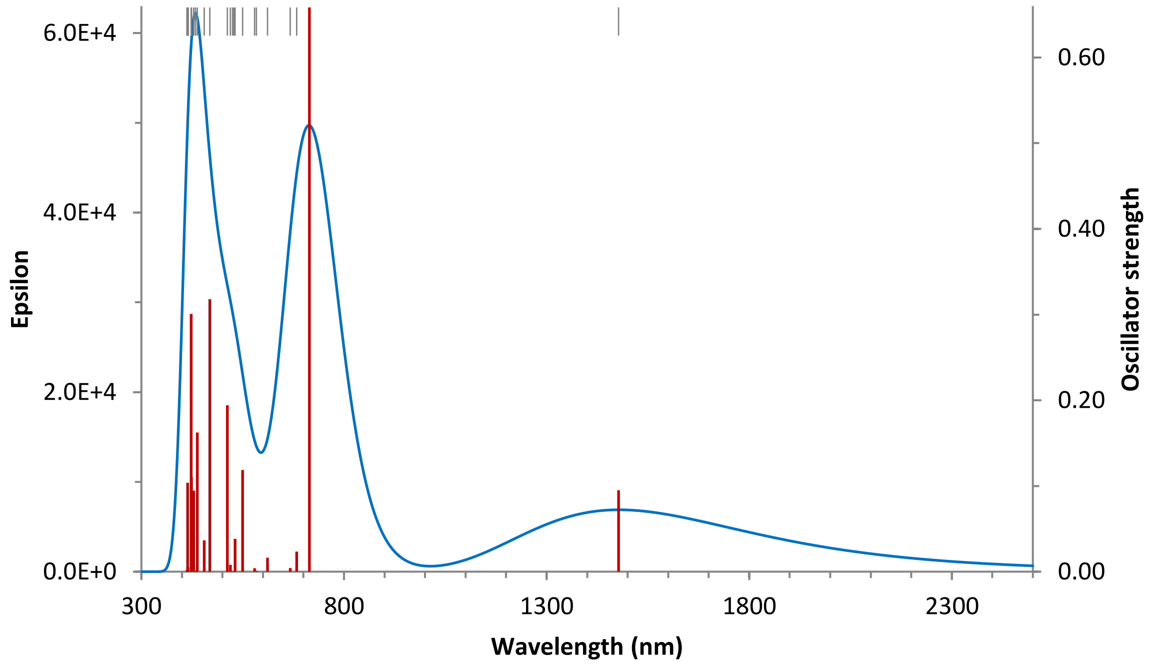

Figure S77. Simulated electronic absorption spectrum of **1f'**−H^+ (TD/PCM(dichloromethane)GD3BJ-B3LYP/6-31G(d,p)).

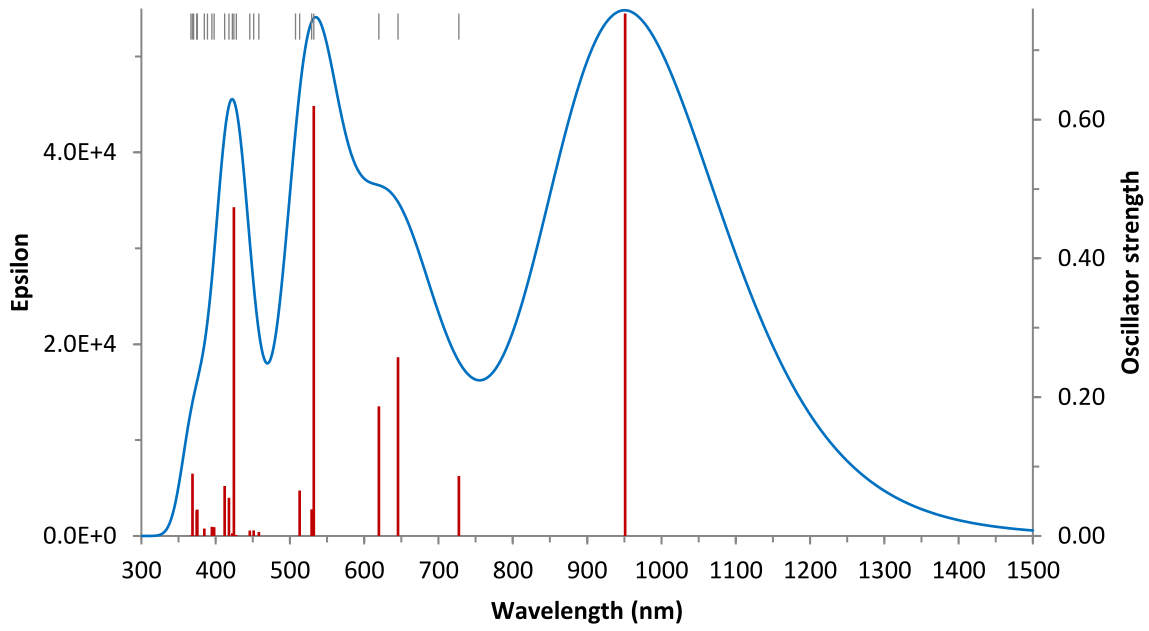

Figure S78. Simulated electronic absorption spectrum of **2a** (TD/PCM(dichloromethane)GD3BJ-B3LYP/6-31G(d,p)).

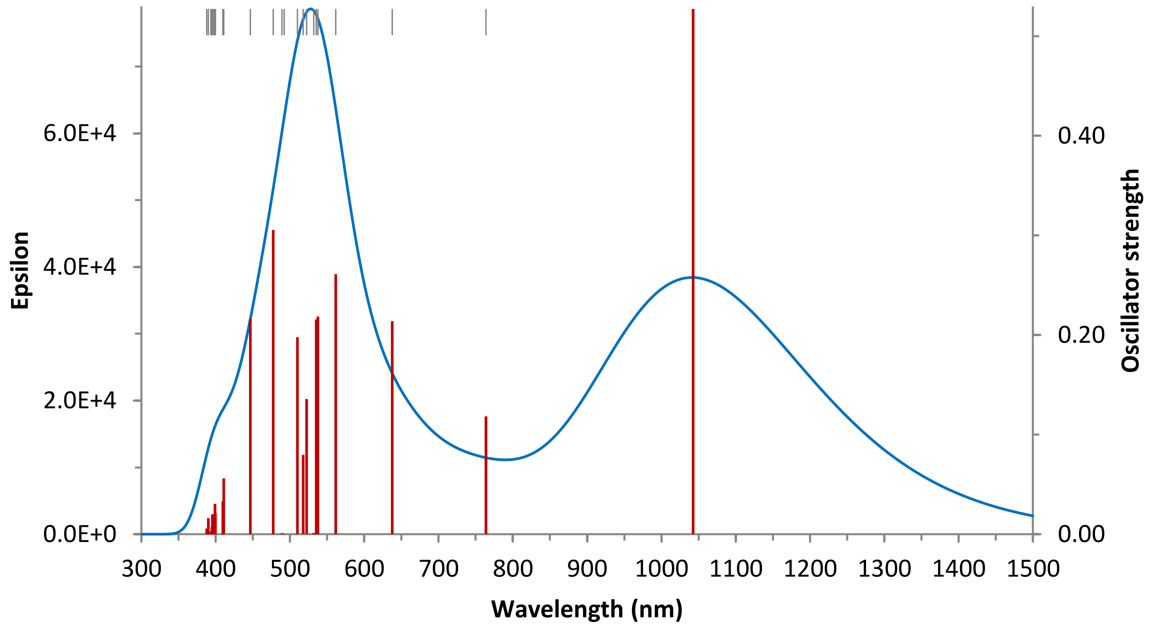

Figure S79. Simulated electronic absorption spectrum of **2a**–H^+ (TD/PCM(dichloromethane)GD3BJ-B3LYP/6-31G(d,p)).

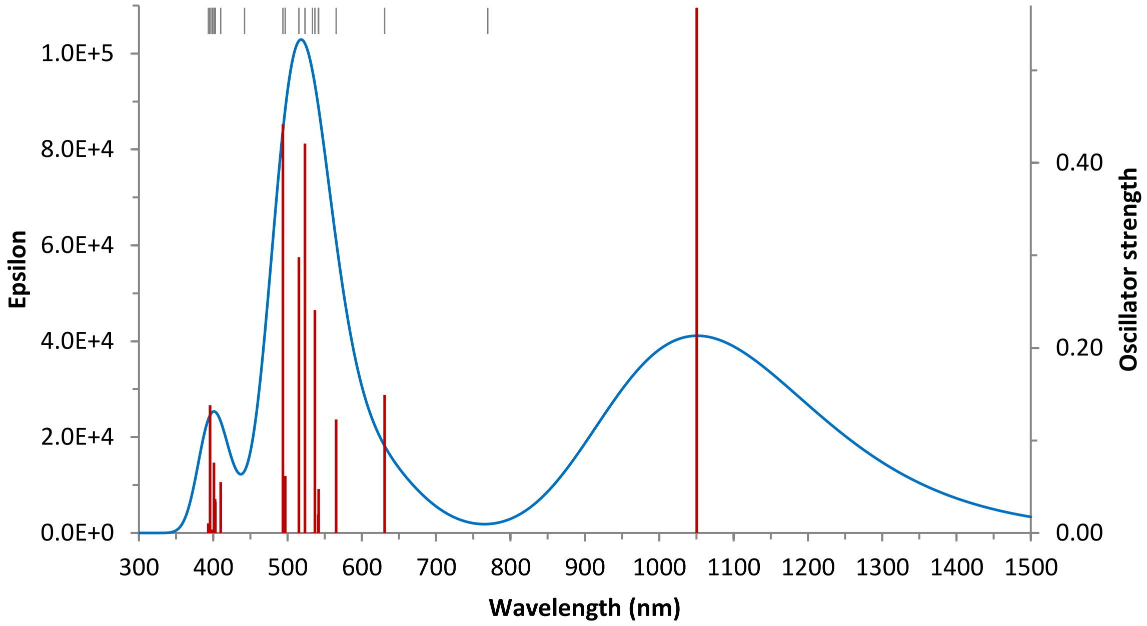

Figure S80. Simulated electronic absorption spectrum of **2a**–H⁺ (*E-anti*) (TD/PCM(dichloromethane)GD3BJ-B3LYP/6-31G(d,p)).

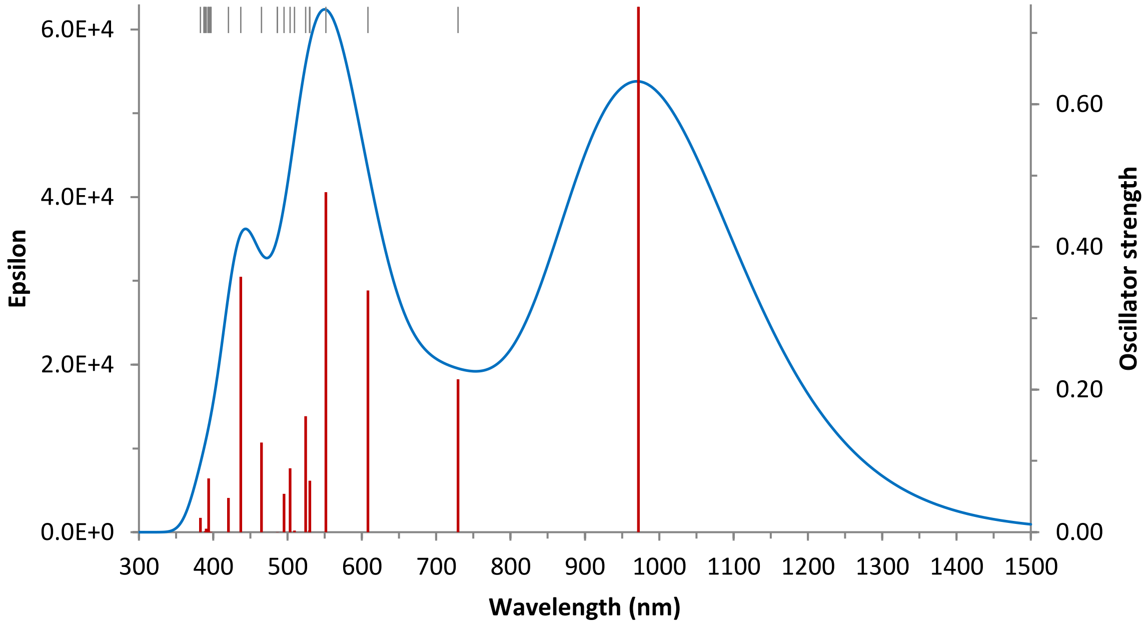

Figure S81. Simulated electronic absorption spectrum of **2a**–H⁺ (*Z-syn*) (TD/PCM(dichloromethane)GD3BJ-B3LYP/6-31G(d,p)).

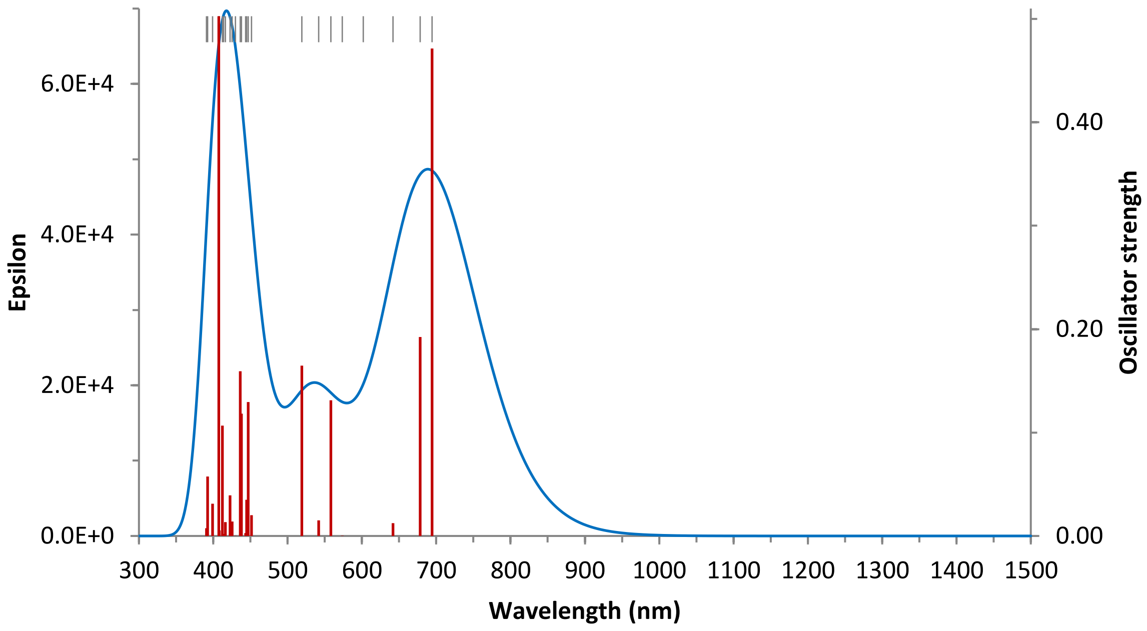

Figure S82. Simulated electronic absorption spectrum of **2a**–$3H^{3+}$ (TD/PCM(dichloromethane)GD3BJ-B3LYP/6-31G(d,p)).

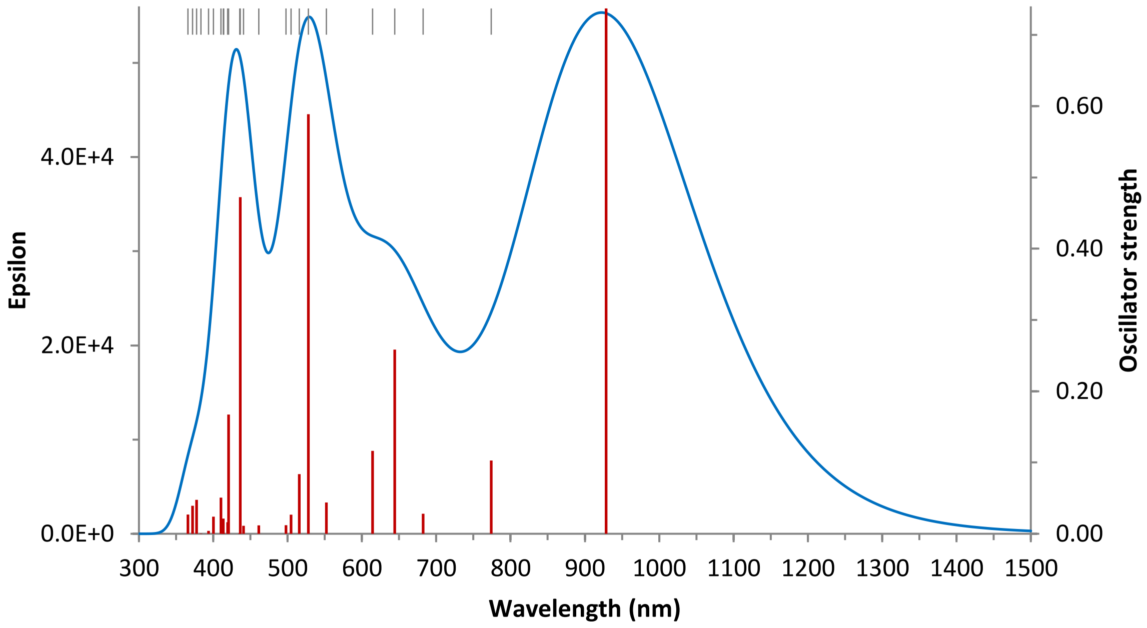

Figure S83. Simulated electronic absorption spectrum of **2b** (TD/PCM(dichloromethane)GD3BJ-B3LYP/6-31G(d,p)).

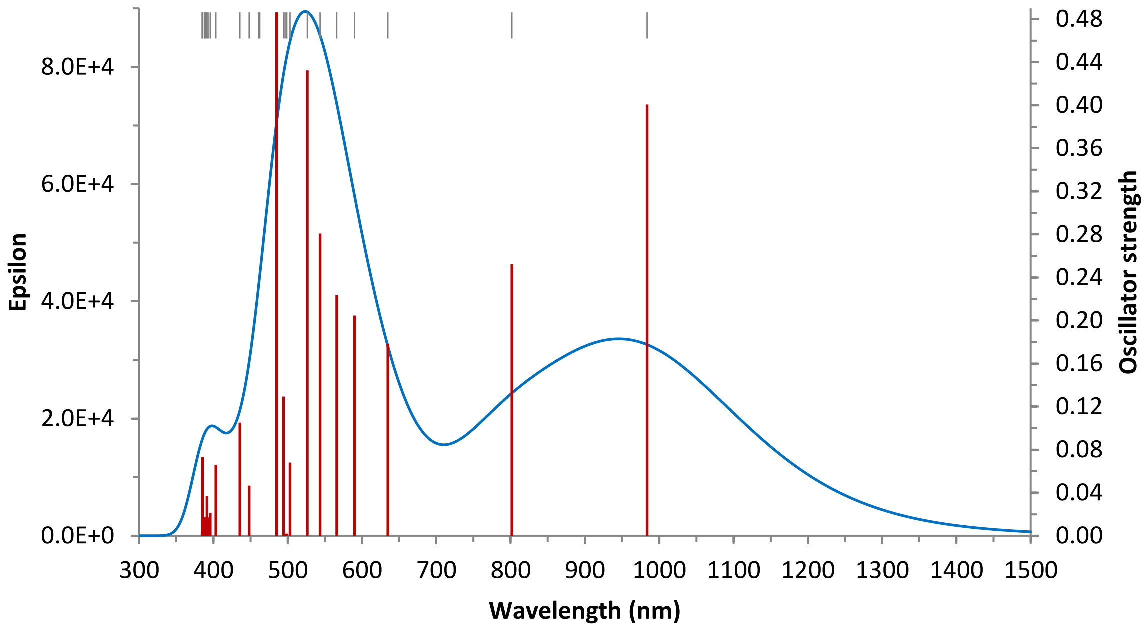

Figure S84. Simulated electronic absorption spectrum of **2b**–H^{+} (TD/PCM(dichloromethane)GD3BJ-B3LYP/6-31G(d,p)).

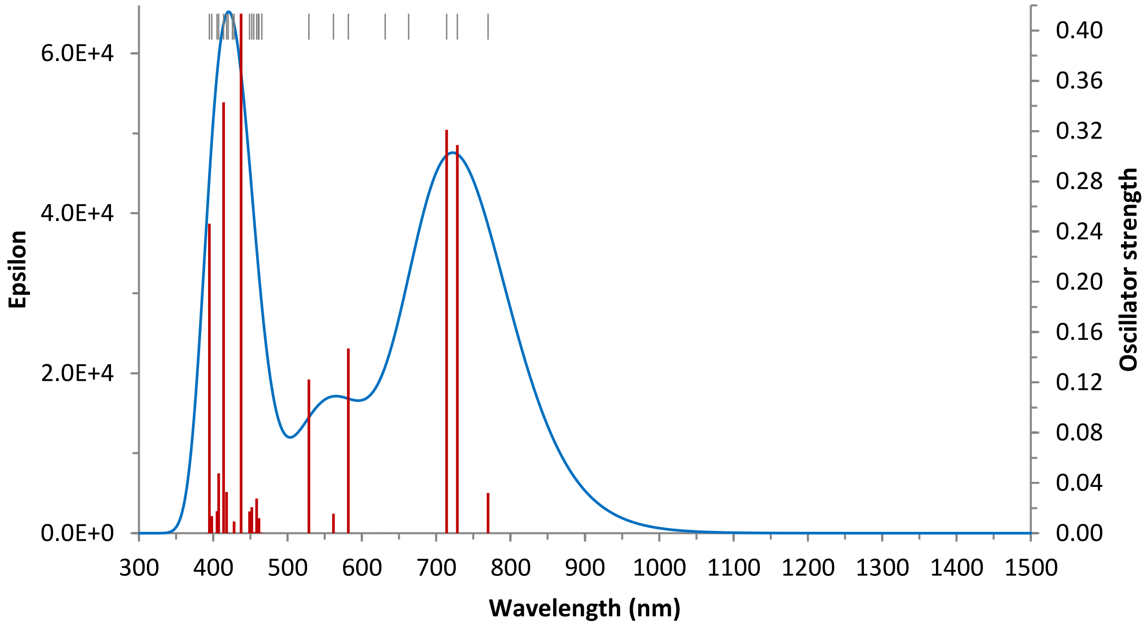

Figure S85. Simulated electronic absorption spectrum of **2b**–$4H^{4+}$ (TD/PCM(dichloromethane)GD3BJ-B3LYP/6-31G(d,p)).

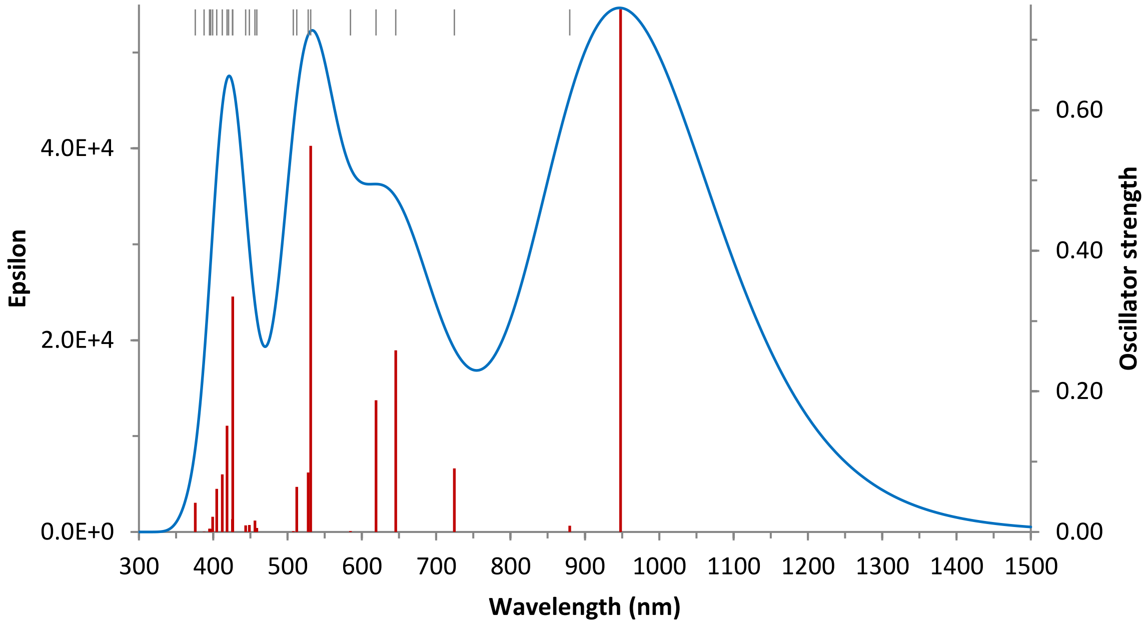

Figure S86. Simulated electronic absorption spectrum of **2c** (TD/PCM(dichloromethane)GD3BJ-B3LYP/6-31G(d,p)).

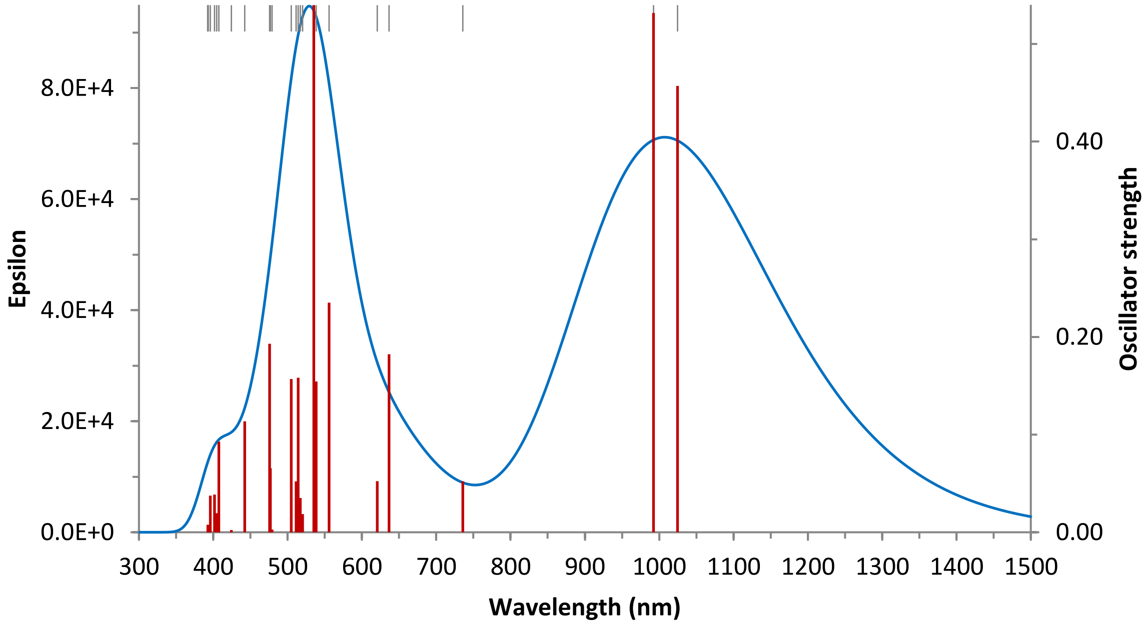

Figure S87. Simulated electronic absorption spectrum of **2c**–1H^+ (TD/PCM(dichloromethane)GD3BJ-B3LYP/6-31G(d,p)).

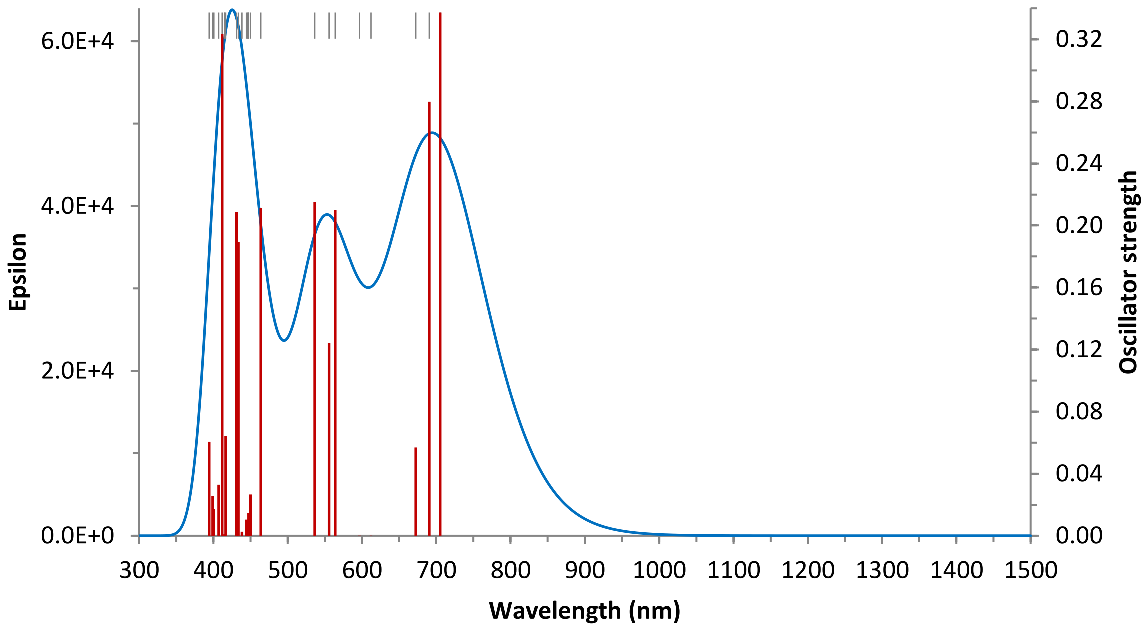

Figure S88. Simulated electronic absorption spectrum of **2c**–$4H^{4+}$ (TD/PCM(dichloromethane)GD3BJ-B3LYP/6-31G(d,p)).

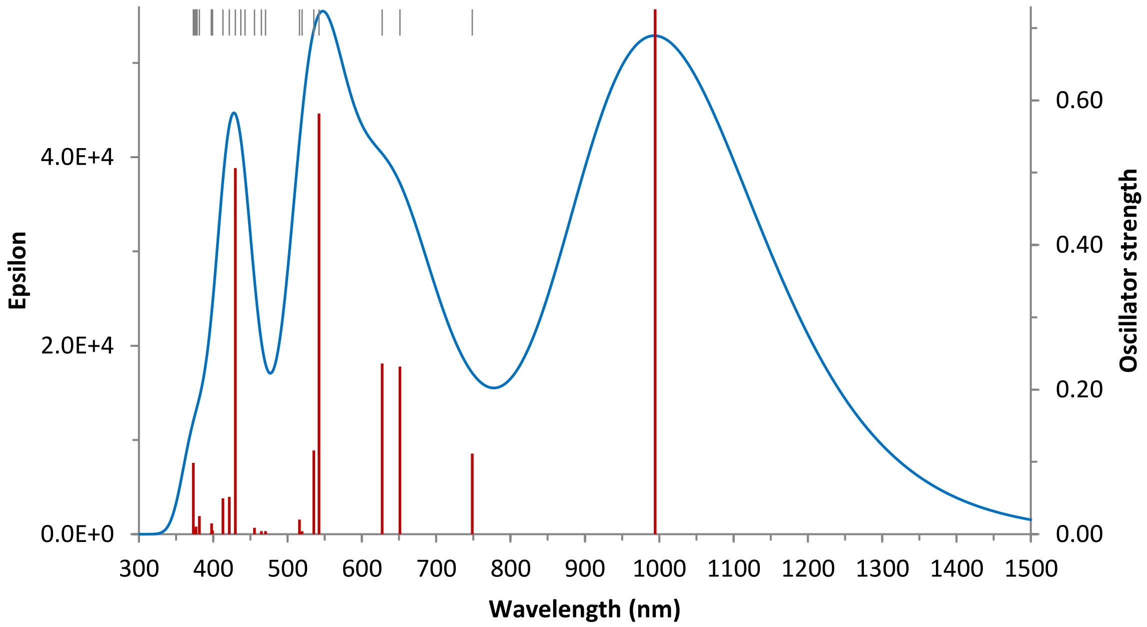

Figure S89. Simulated electronic absorption spectrum of **2d** (TD/PCM(dichloromethane)GD3BJ-B3LYP/6-31G(d,p)).

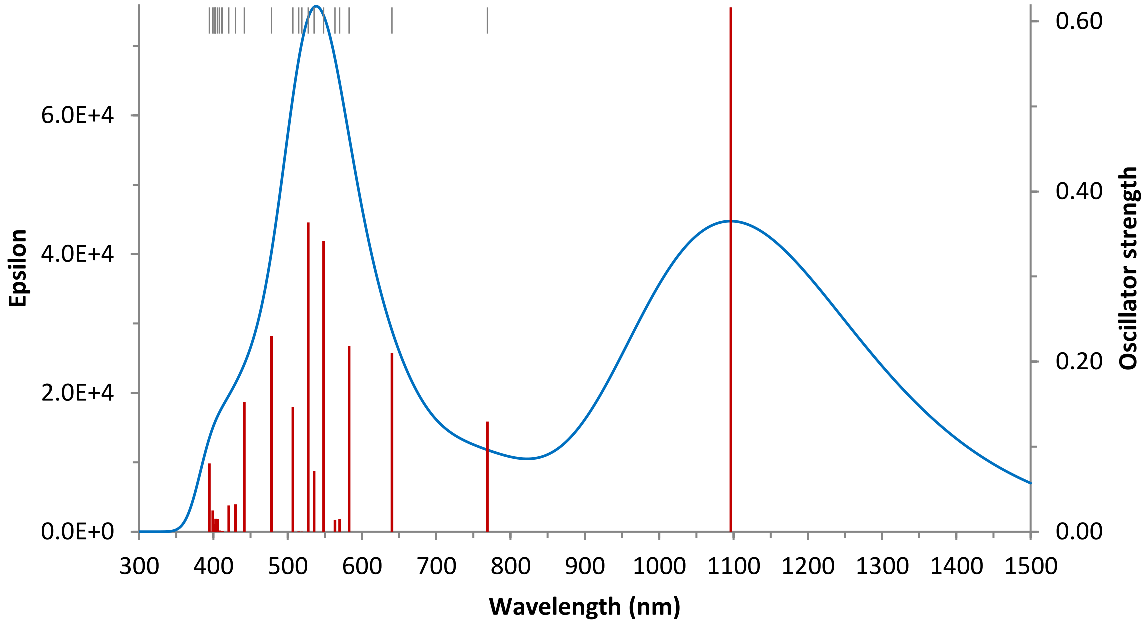

Figure S90. Simulated electronic absorption spectrum of **2d**−1H⁺ (TD/PCM(dichloromethane)GD3BJ-B3LYP/6-31G(d,p)).

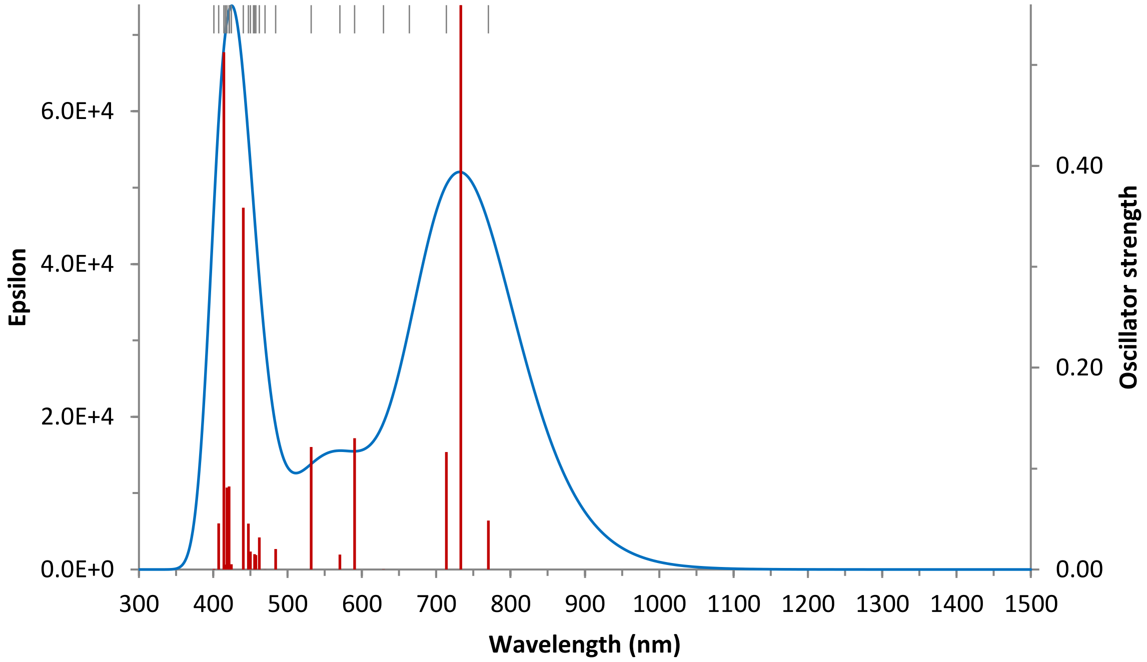

Figure S91. Simulated electronic absorption spectrum of **2d**−3H^{3+} (TD/PCM(dichloromethane)GD3BJ-B3LYP/6-31G(d,p)).

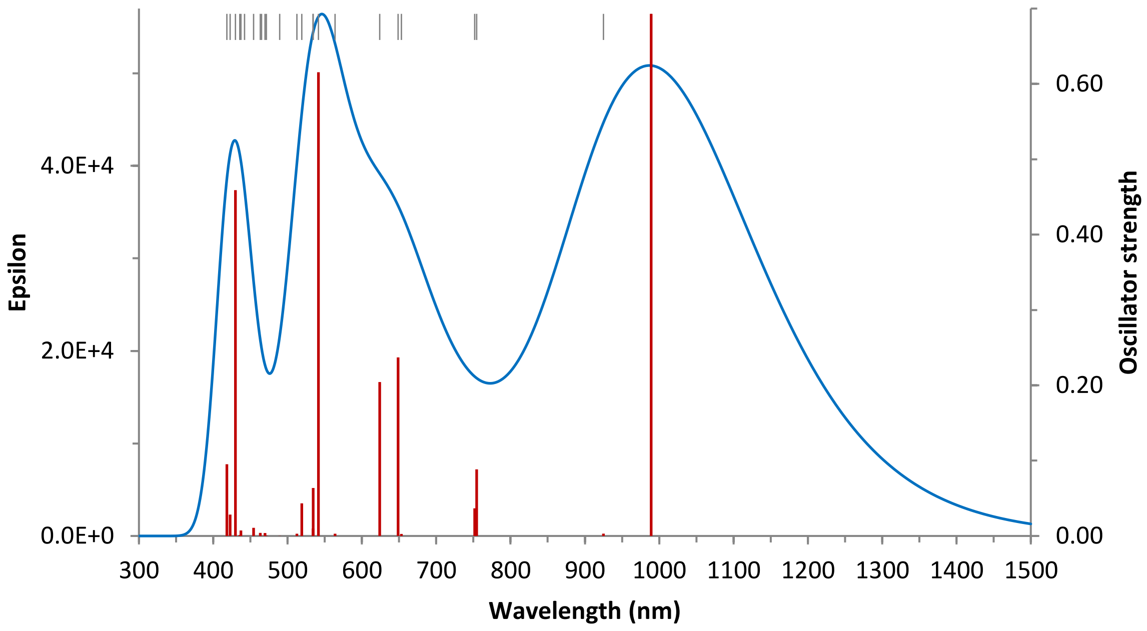

Figure S92. Simulated electronic absorption spectrum of **2e** (TD/PCM(dichloromethane)GD3BJ-B3LYP/6-31G(d,p)).

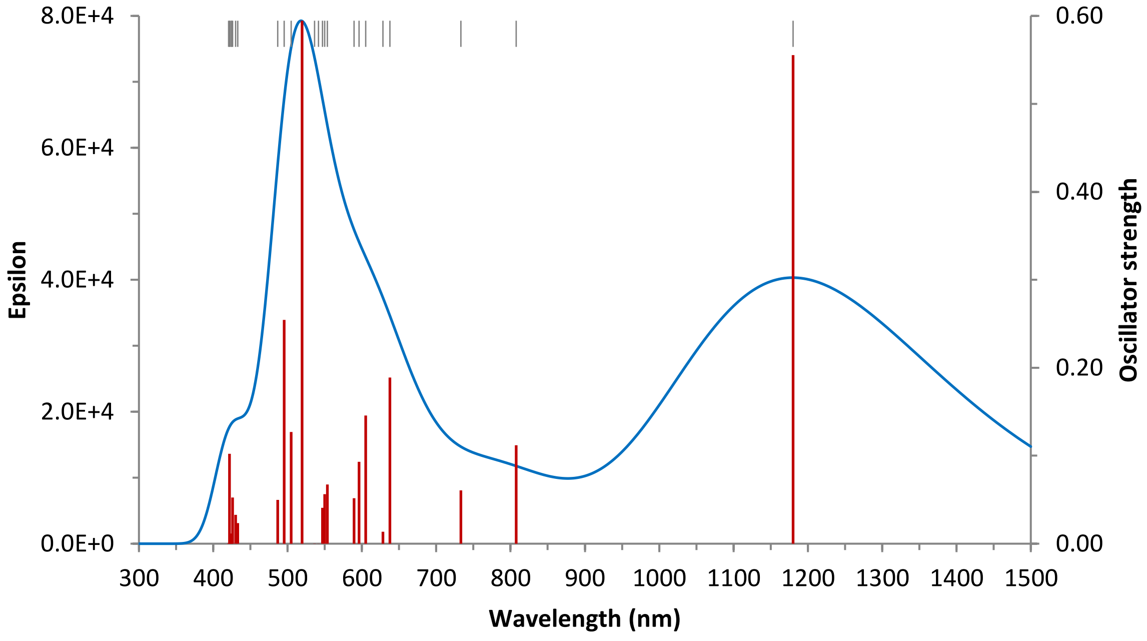

Figure S93. Simulated electronic absorption spectrum of **2e**–1H^{+} (TD/PCM(dichloromethane)GD3BJ-B3LYP/6-31G(d,p)).

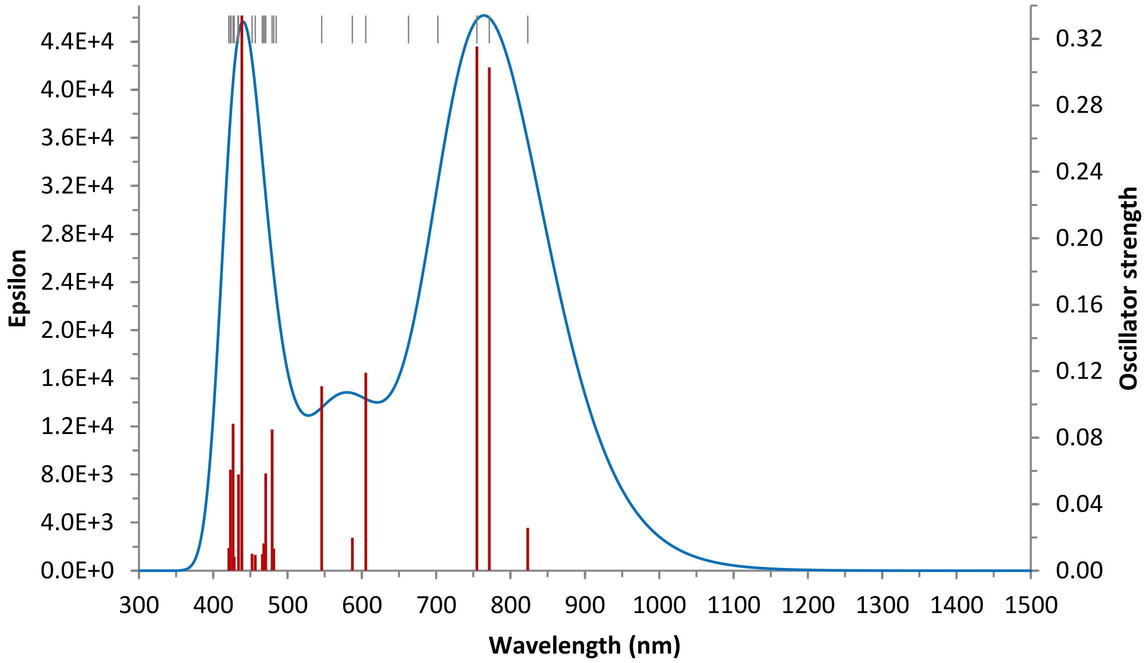

Figure S94. Simulated electronic absorption spectrum of **2e**–$3H^{3+}$ (TD/PCM(dichloromethane)GD3BJ-B3LYP/6-31G(d,p)).

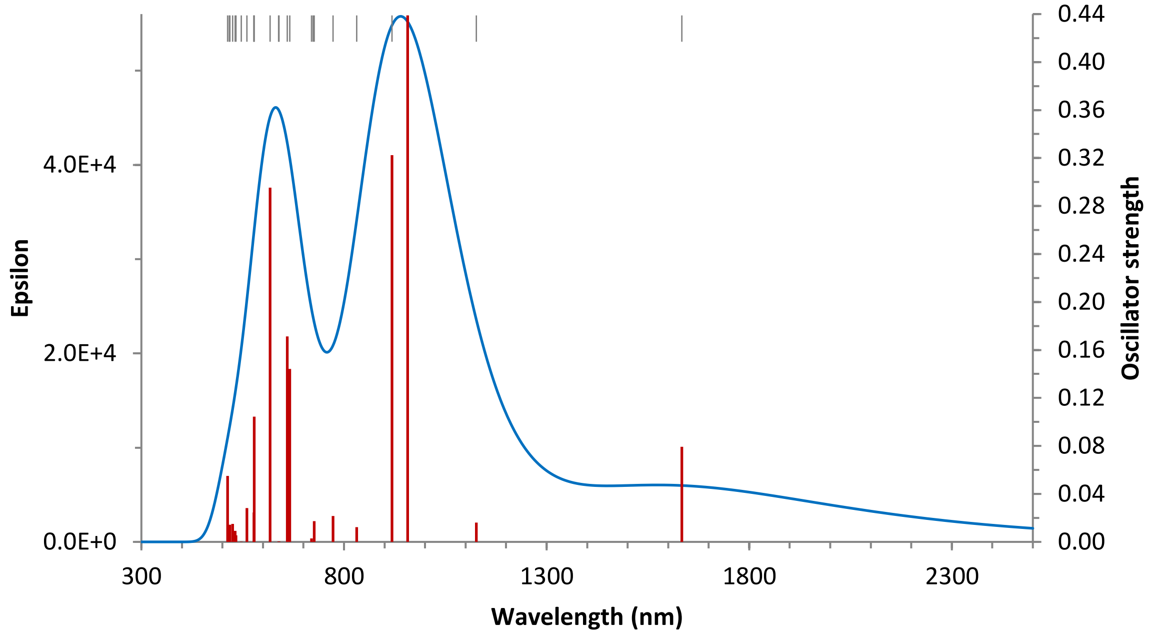

Figure S95. Simulated electronic absorption spectrum of **2a**$^{+•}$ (TD/PCM(dichloromethane)GD3BJ-UB3LYP/6-31G(d,p)).

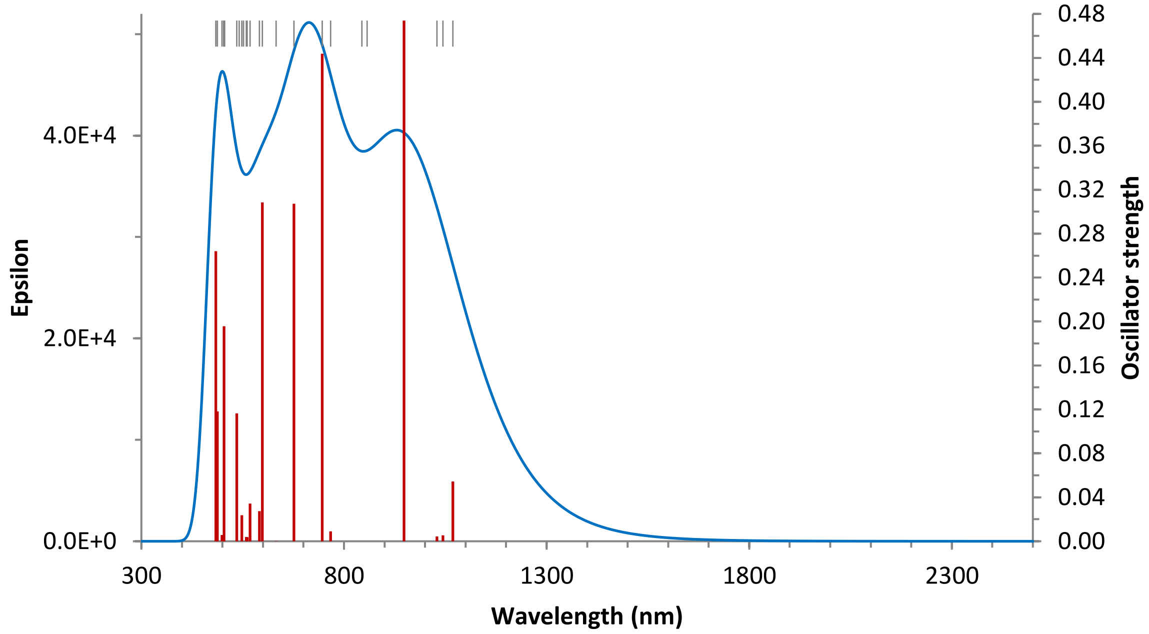

Figure S96. Simulated electronic absorption spectrum of **2d**$^{2+}$ (closed-shell configuration) (TD/PCM(dichloromethane)GD3BJ-B3LYP/6-31G(d,p)).

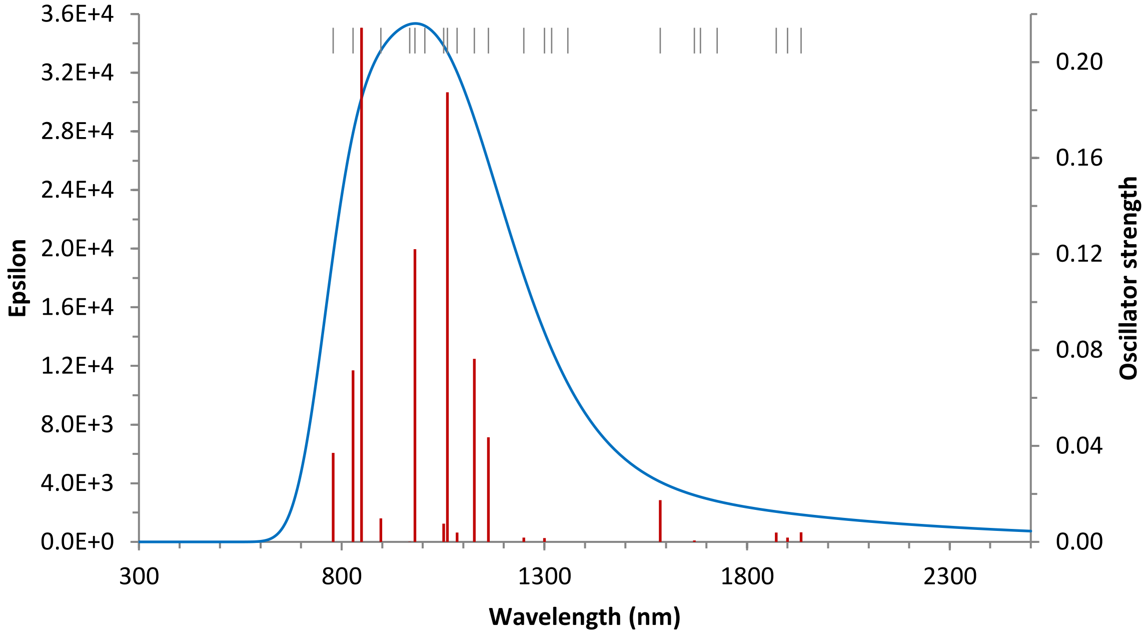

Figure S97. Simulated electronic absorption spectrum of **2a**$^{3+\bullet}$ (TD/PCM(dichloromethane)GD3BJ-UB3LYP/6-31G(d,p)).

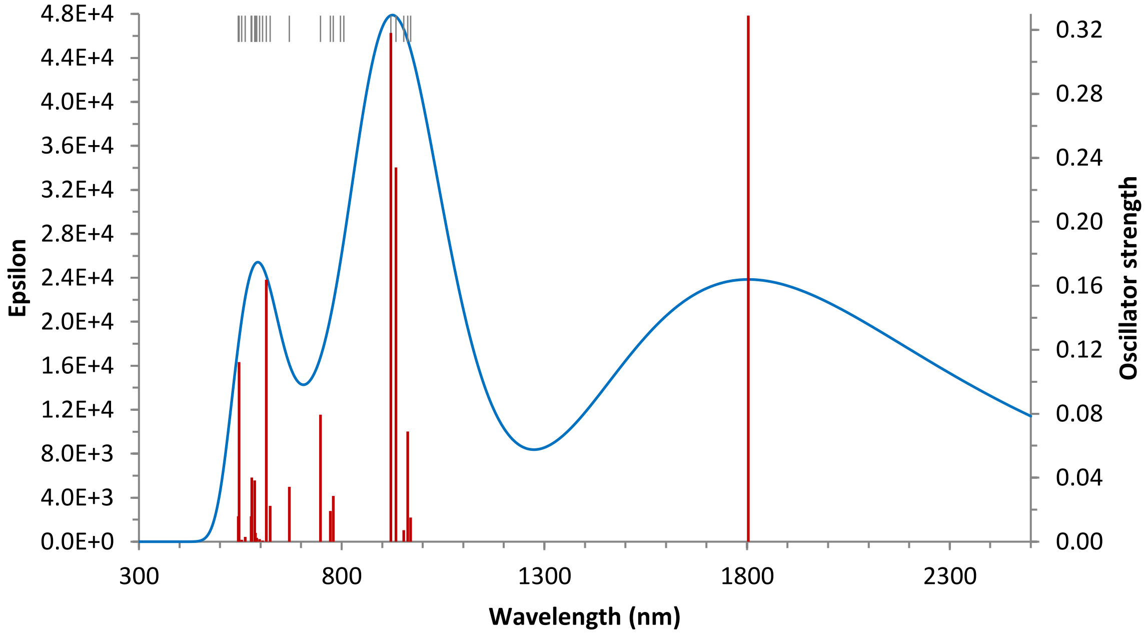

Figure S98. Simulated electronic absorption spectrum of protonated radical, **2a**–H$^{\bullet++}$ (TD/PCM(dichloromethane)GD3BJ-UB3LYP/6-31G(d,p)).

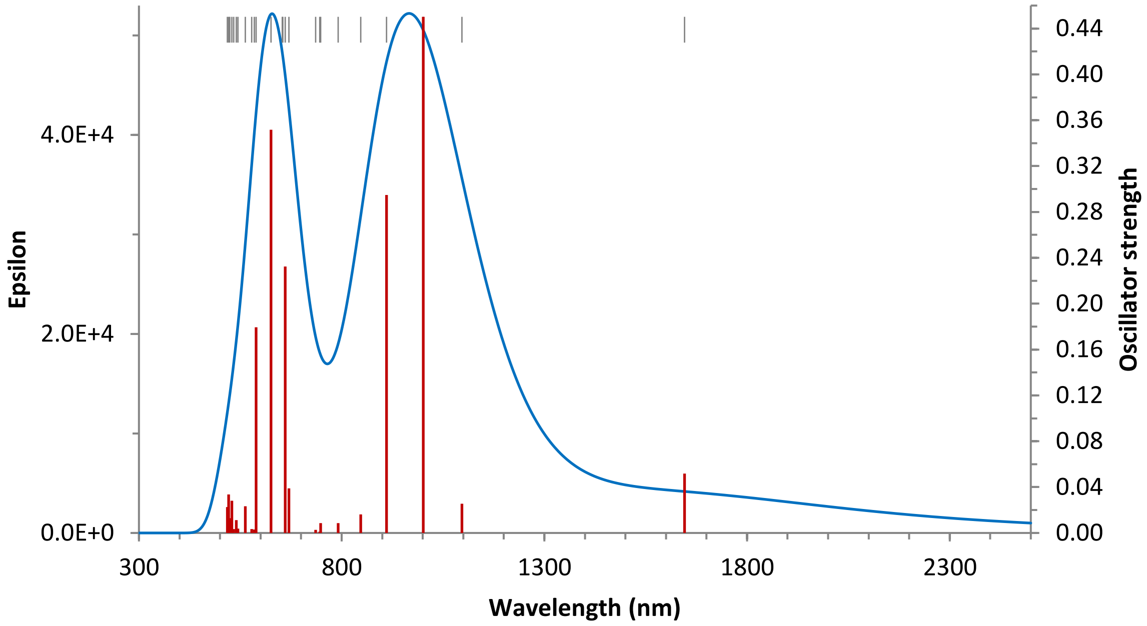

Figure S99. Simulated electronic absorption spectrum of **2d**$^{+\bullet}$ (TD/PCM(dichloromethane)GD3BJ-UB3LYP/6-31G(d,p)).

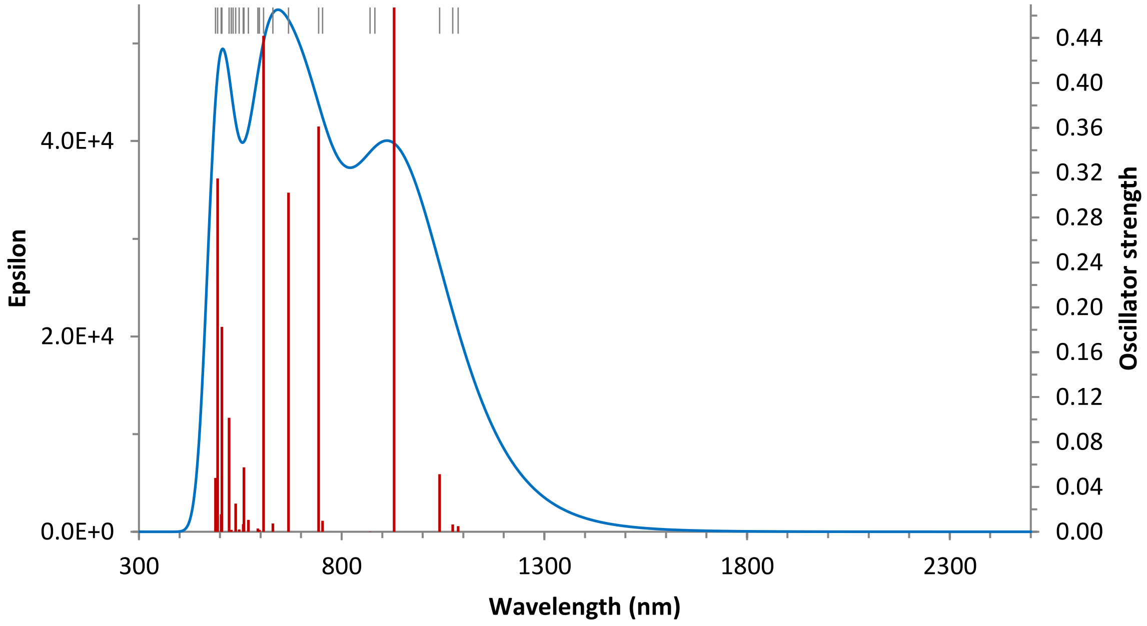

Figure S100. Simulated electronic absorption spectrum of **2d**$^{2+}$ (closed-shell configuration) (TD/PCM(dichloromethane)GD3BJ-B3LYP/6-31G(d,p)).

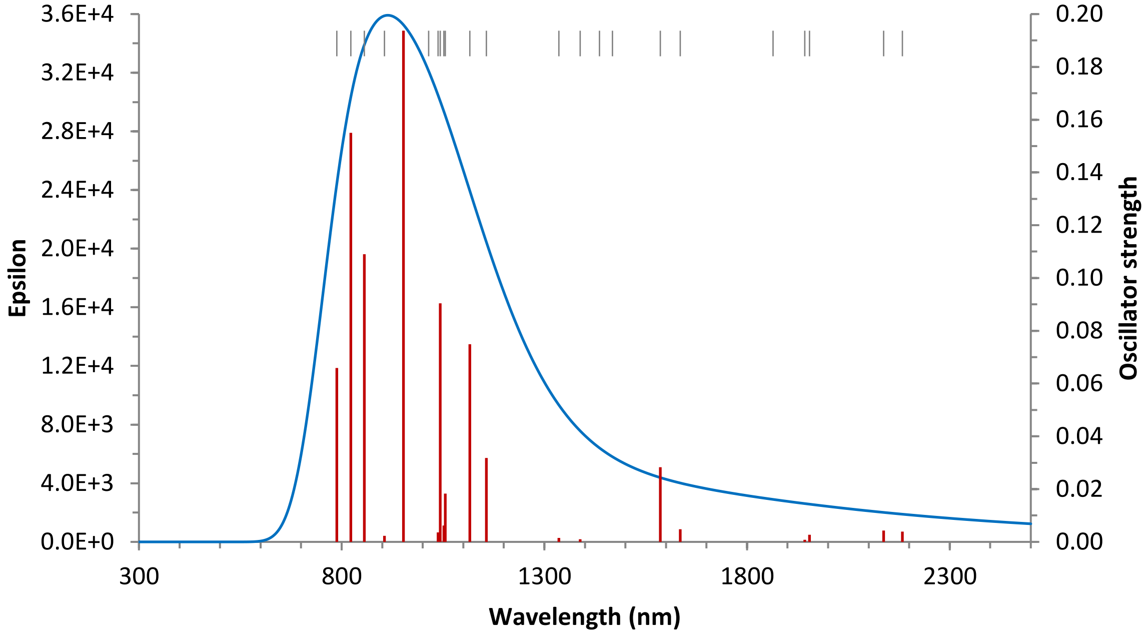

Figure S101. Simulated electronic absorption spectrum of **2d**$^{3+\bullet}$ (TD/PCM(dichloromethane)GD3BJ-UB3LYP/6-31G(d,p)).

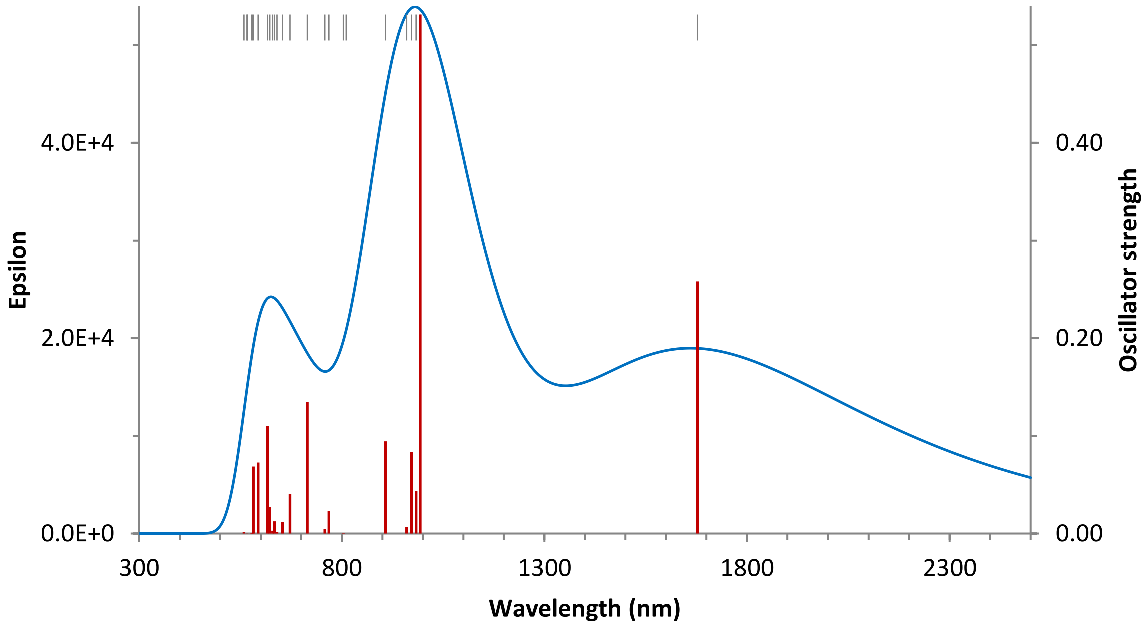

Figure S102. Simulated electronic absorption spectrum of protonated radical, **2d**–H$^{\bullet++}$ (TD/PCM(dichloromethane)GD3BJ-UB3LYP/6-31G(d,p)).

Additional Tables

Table S1. Crystal data and structure refinement for **1a**·$0.5C_6H_{14}$·CH_3OH

Identification code	ym115-2	
Empirical formula	C_{79} H_{69} N_4 O_5	
Formula weight	1154.38	
Temperature	80(2) K	
Wavelength	1.54184 Å	
Crystal system	Triclinic	
Space group	P-1	
Unit cell dimensions	a = 17.336(10) Å	α= 92.02(5)°.
	b = 17.795(10) Å	β= 97.60(5)°.
	c = 21.42(2) Å	γ = 104.28(5)°.
Volume	6331(8) Å^3	
Z	4	
Density (calculated)	1.211 Mg/m^3	
Absorption coefficient	0.591 mm^{-1}	
F(000)	2444	
Crystal size	0.570 x 0.250 x 0.030 mm^3	
Theta range for data collection	3.702 to 66.997°.	
Index ranges	-20<=h<=20, -21<=k<=19, -20<=l<=25	
Reflections collected	47155	
Independent reflections	22202 [R(int) = 0.1942]	
Completeness to theta = 67.000°	98.3 %	
Absorption correction	None	
Refinement method	Full-matrix least-squares on F^2	
Data / restraints / parameters	22202 / 28 / 1420	
Goodness-of-fit on F^2	0.980	
Final R indices [I>2sigma(I)]	R1 = 0.1568, wR2 = 0.2860	
R indices (all data)	R1 = 0.3768, wR2 = 0.4110	
Extinction coefficient	n/a	
Largest diff. peak and hole	0.295 and -0.306 e.Å^{-3}	

Table S2. Crystal data and structure refinement for **1b**·3$CHCl_3$.

Identification code	ymo67a	
Empirical formula	C_{80} H_{66} Cl_9 N_5 O_4	
Formula weight	1480.42	
Temperature	100(2) K	
Wavelength	1.54184 Å	
Crystal system	Monoclinic	
Space group	$P2_1/c$	
Unit cell dimensions	*a* = 9.765(3) Å	α= 90°.
	b = 24.206(8) Å	β= 93.26(2)°.
	c = 30.365(8) Å	γ = 90°.
Volume	7166(4) $Å^3$	
Z	4	
Density (calculated)	1.372 Mg/m^3	
Absorption coefficient	3.655 mm^{-1}	
F(000)	3064	
Crystal size	0.370 x 0.040 x 0.040 mm^3	
Theta range for data collection	2.336 to 75.751°.	
Index ranges	-11<=h<=10, -29<=k<=29, -37<=l<=31	
Reflections collected	30591	
Independent reflections	14384 [R(int) = 0.0870]	
Completeness to theta = 68.000°	99.9 %	
Absorption correction	Analytical	
Max. and min. transmission	0.880 and 0.513	
Refinement method	Full-matrix least-squares on F^2	
Data / restraints / parameters	14384 / 0 / 894	
Goodness-of-fit on F^2	0.835	
Final R indices [I>2sigma(I)]	R1 = 0.0574, wR2 = 0.0818	
R indices (all data)	R1 = 0.1442, wR2 = 0.0904	
Extinction coefficient	n/a	
Largest diff. peak and hole	0.492 and -0.447 e.$Å^{-3}$	

Table S3. Crystal data and structure refinement for **1e**·$0.5C_6H_{14}$·$CHCl_3$.

Identification code	ym204a	
Empirical formula	C_{79} H_{64} Cl_3 N_6 O_8	
Formula weight	1331.71	
Temperature	100(2) K	
Wavelength	1.54184 Å	
Crystal system	Monoclinic	
Space group	$P2_1/c$	
Unit cell dimensions	*a* = 24.954(4) Å	α= 90°.
	b = 9.5250(11) Å	β= 107.66(3)°.
	c = 29.697(4) Å	γ= 90°.
Volume	6725.9(19) $Å^3$	
Z	4	
Density (calculated)	1.315 Mg/m^3	
Absorption coefficient	1.745 mm^{-1}	
F(000)	2780	
Crystal size	0.470 x 0.056 x 0.013 mm^3	
Theta range for data collection	3.113 to 71.262°.	
Index ranges	-30<=h<=29, -11<=k<=11, -30<=l<=36	
Reflections collected	54813	
Independent reflections	12742 [R(int) = 0.0458]	
Completeness to theta = 67.000°	99.9 %	
Absorption correction	Gaussian	
Max. and min. transmission	1.000 and 0.491	
Refinement method	Full-matrix least-squares on F^2	
Data / restraints / parameters	12742 / 27 / 928	
Goodness-of-fit on F^2	1.221	
Final R indices [I>2sigma(I)]	R1 = 0.0857, wR2 = 0.2101	
R indices (all data)	R1 = 0.1017, wR2 = 0.2177	
Extinction coefficient	n/a	
Largest diff. peak and hole	0.675 and -0.658 e.$Å^{-3}$	

Table S4. Crystal data and structure refinement for **2e**·4$CHCl_3$

Identification code	ym244a	
Empirical formula	$C_{83} H_{66} D_4 Cl_{12} N8 O_8$	
Formula weight	1736.89	
Temperature	120(2) K	
Wavelength	1.5418 Å	
Crystal system	Monoclinic	
Space group	$P2_1/n$	
Unit cell dimensions	a = 21.760(7) Å	α= 90°.
	b = 14.957(5) Å	β= 94.58(5)°.
	c = 24.800(7) Å	γ = 90°.
Volume	8046(4) $Å^3$	
Z	4	
Density (calculated)	1.434 Mg/m^3	
Absorption coefficient	4.286 mm^{-1}	
F(000)	3568	
Crystal size	0.600 x 0.110 x 0.100 mm^3	
Theta range for data collection	2.601 to 67.000°.	
Index ranges	-20<=h<=25, -17<=k<=17, -29<=l<=29	
Reflections collected	40841	
Independent reflections	14342 [R(int) = 0.1737]	
Completeness to theta = 67.000°	99.9 %	
Absorption correction	Analytical	
Max. and min. transmission	0.725 and 0.327	
Refinement method	Full-matrix least-squares on F^2	
Data / restraints / parameters	14342 / 0 / 1009	
Goodness-of-fit on F^2	1.007	
Final R indices [I>2sigma(I)]	R1 = 0.0978, wR2 = 0.2165	
R indices (all data)	R1 = 0.1953, wR2 = 0.2984	
Extinction coefficient	n/a	
Largest diff. peak and hole	0.500 and -0.614 e.$Å^{-3}$	

Table S5. Crystal data and structure refinement for **1a**–H^+·4$CDCl_3$·Br.

Identification code	ym178	
Empirical formula	C_{79} H_{59} D_4 Br Cl_{12} N_4 O_4	
Formula weight	1641.66	
Temperature	100(2) K	
Wavelength	0.71073 Å	
Crystal system	Triclinic	
Space group	P-1	
Unit cell dimensions	a = 10.492(3) Å	α= 93.69(3)°.
	b = 17.053(4) Å	β= 98.39(3)°.
	c = 22.142(5) Å	γ = 105.31(3)°.
Volume	3758.1(17) $Å^3$	
Z	2	
Density (calculated)	1.451 Mg/m^3	
Absorption coefficient	1.027 mm^{-1}	
F(000)	1672	
Crystal size	0.500 x 0.300 x 0.300 mm^3	
Theta range for data collection	2.879 to 25.500°.	
Index ranges	-10<=h<=12, -20<=k<=20, -26<=l<=26	
Reflections collected	38738	
Independent reflections	13962 [R(int) = 0.1104]	
Completeness to theta = 25.500°	99.8 %	
Absorption correction	Semi-empirical from equivalents	
Max. and min. transmission	1.00000 and 0.93164	
Refinement method	Full-matrix least-squares on F^2	
Data / restraints / parameters	13962 / 0 / 909	
Goodness-of-fit on F^2	1.039	
Final R indices [I>2sigma(I)]	R1 = 0.0845, wR2 = 0.2001	
R indices (all data)	R1 = 0.1526, wR2 = 0.2461	
Extinction coefficient	n/a	
Largest diff. peak and hole	1.055 and -1.082 e.$Å^{-3}$	

Table S6. Crystal data and structure refinement for **1b**–$H^+ \cdot 3.15CDCl_3 \cdot Cl \cdot 0.6H_2O$

Identification code	ym179a	
Empirical formula	$C_{80.15}$ $H_{64.60}$ $Cl_{10.45}$ $D_{3.15}$ N_5 $O_{4.60}$	
Formula weight	1548.16	
Temperature	100(2) K	
Wavelength	1.54184 Å	
Crystal system	Monoclinic	
Space group	C2/c	
Unit cell dimensions	*a* = 20.023(5) Å	α= 90°.
	b = 23.518(7) Å	β= 97.91(2)°.
	c = 33.749(9) Å	γ = 90°.
Volume	15741(7) Å^3	
Z	8	
Density (calculated)	1.307 Mg/m^3	
Absorption coefficient	3.797 mm^{-1}	
F(000)	6385	
Crystal size	0.320 x 0.100 x 0.060 mm^3	
Theta range for data collection	3.724 to 66.996°.	
Index ranges	-23<=h<=11, -28<=k<=27, -39<=l<=40	
Reflections collected	33630	
Independent reflections	13792 [R(int) = 0.0749]	
Completeness to theta = 67.000°	98.2 %	
Absorption correction	Analytical	
Max. and min. transmission	0.842 and 0.577	
Refinement method	Full-matrix least-squares on F^2	
Data / restraints / parameters	13792 / 150 / 1005	
Goodness-of-fit on F^2	1.020	
Final R indices [I>2sigma(I)]	R1 = 0.0786, wR2 = 0.1955	
R indices (all data)	R1 = 0.1334, wR2 = 0.2301	
Extinction coefficient	n/a	
Largest diff. peak and hole	0.570 and -0.443 e.Å^{-3}	

Table S7. Crystal data and structure refinement for disordered co-crystal of **2d** and **3d**. The data were not deposited because of their low quality.

Identification code	ym294	
Empirical formula	$C_{100}H_{100}N_4O_4F_5ClSb$	
Formula weight	1674.03	
Temperature	100(2) K	
Wavelength	1.54184 Å	
Crystal system	Monoclinic	
Space group	$P2_1/n$	
Unit cell dimensions	*a* = 8.6176(5) Å	α= 90°.
	b = 22.4452(19) Å	β= 90.252(7)°.
	c = 42.943(3) Å	γ = 90°.
Volume	8306.1(10) $Å^3$	
Z	4	
Density (calculated)	1.339 Mg/m^3	
Absorption coefficient	3.477 mm^{-1}	
F(000)	3492.0	
Crystal size	n/a	
Theta range for data collection	4.116 to 178.592°.	
Index ranges	$-10 \leq h \leq 11$, $-25 \leq k \leq 24$, $-25 \leq l \leq 24$	
Reflections collected	40808	
Independent reflections	8430 [R_{int} = 0.5266, R_{sigma} = 0.2883]	
Completeness to theta = 68.000°	n/a	
Absorption correction	n/a	
Max. and min. transmission	n/a	
Refinement method	n/a	
Data / restraints / parameters	8430/13/979	
Goodness-of-fit on F^2	5.322	
Final R indices [I>2sigma(I)]	R1 = 0.8317, wR2 = 0.9585	
R indices (all data)	R1 = 0.8592, wR2 = 0.9741	
Extinction coefficient	n/a	
Largest diff. peak and hole	3.29 and -1.96 e.$Å^{-3}$	

Table S8. Quantum yields of dipyrrin **1a**–**1e** in different solvents.

QY (%)	toluene	dichloromethane	benzonitrile
**1a**	0.7	1.1	2.1
**1b**	0.8	0.5	< 0.1
**1c**	< 0.1	< 0.1	< 0.1
**1d**	< 0.1	< 0.1	< 0.1
**1e**	0.1	< 0.1	< 0.1

Table S9. Photophysical Properties.

	1a in tol	1b in tol	1a in BCN	1a in DCM	1b in DCM
**QY (%)**	0.7	0.8	2.1	1.1	0.5
**k_r (s^{-1})**	3.43E+06	3.40E+06	6.38E+06	4.68E+07	1.73E+07
**k_{nr} (s^{-1})**	4.87E+08	4.22E+08	2.97E+08	4.21E+08	3.66E+08
**Singlet lifetime (ns)**	2.04	2.35	3.29	2.35	2.72

Table S10. Redox potentials (in volts vs. ferrocene internal standard) from differential pulse voltammetry of dichloromethane solutions with tetrabutylammonium hexaflurophosphate as a supporting electrolyte, glassy carbon working electrode, platinum wire counter electrode, and silver chloride reference electrode.

code	E_{Ox1}	E_{Ox2}	E_{Ox3}	E_{Ox4}	E_{Ox5}	E_{Red1}	E_{Red2}	E_{Red3}	E_{Red4}	E_{Red5}	ΔE
**1a**	0.82	1.15[a]	—	—	—	−0.94	−1.28	−1.83	−2.22	—	1.76
**1b**	0.62[a]	0.89	1.00[a]	1.16	—	−1.02	−1.33	−1.88	−2.27	—	1.62
**1c**	0.43	0.80	1.17	—	—	−0.96	−1.29	−1.85	−2.31[a]	—	1.39
**1d**	0.89	1.23[a]	—	—	—	−0.77	−1.13	−1.80	−2.21[a]	—	1.66
**1e**	0.88	1.25[a]	1.48[a]	—	—	−0.78	−1.03	−1.59	−1.87	−2.12[a]	1.66
**2a**	0.19	0.30	1.03	1.42[a]	—	−1.04	−1.31	−1.88	−2.25	—	1.23
**2b**	0.19	0.30	0.58	0.89[a]	1.04[a]	−1.06	−1.34	−1.94	—	—	1.25
**2c**	0.21	0.33	0.49	1.02[a]	1.34[a]	−1.05	−1.34	−1.95	—	—	1.26
**2d**	0.24	0.33	1.14[a]	—	—	−0.90	−1.20	−1.89	—	—	1.14
**2e**	0.26	0.36	1.13	—	—	−0.92	−1.13	−1.62	−1.97	−2.20[a]	1.18

[a] Irreversible couple

Table S11. Computational details obtained at B3LYP-GD3BJ/6-31G(d,p)/PCM(dichloromethane) level of theory.

Code[a]	Formula	Charge	SCF E[b]	ZPV[c]	lowest freq. [d]	H[e]	G[f]
			a.u.	a.u.	cm^{-1}	a.u.	a.u.
**1a**	C75H58N4O4	0	-3413.473263	1.138495	7.91	-3412.264795	-3412.445437
**1a–H⁺**	C75H59N4O4	1	-3413.927716	1.152713	6.14	-3412.704862	-3412.886082
**1b**	C77H63N5O4	0	-3547.468485	1.211591	6.70	-3546.182335	-3546.374143
**1b–H⁺**	C77H64N5O4	1	-3547.933362	1.226208	7.53	-3546.63255	-3546.823428
**1b–H_2^{2+}**	C77H65N5O4	2	-3548.348604	1.242211	5.61	-3547.03235	-3547.221115
**1c**	C83H67N5O4	0	-3778.558138	1.292516	6.79	-3777.18633	-3777.389375
**1c–H⁺**	C83H68N5O4	1	-3779.017316	1.306244	4.20	-3777.631429	-3777.836698
**1c–H_2^{2+}**	C83H69N5O4	2	-3779.448648	1.322478	6.54	-3778.047123	-3778.24782
**1d**	C75H53F5N4O4	0	-3909.592197	1.097446	7.60	-3908.420007	-3908.611356
**1d–H⁺**	C75H54F5N4O4	1	-3910.041588	1.110632	5.32	-3908.855748	-3909.050355
**1e**	C75H56N6O8	0	-3822.474715	1.142817	7.13	-3821.25651	-3821.451047
**1e-H**	C75H57N6O8	1	-3822.922289	1.156443	5.36	-3821.69013	-3821.886197
**1f'**	C91H70N4O6	0	-4180.964823	1.379421	5.32	-4179.500144	-4179.715359
**1f'–H⁺**	C91H71N4O6	1	-4181.413750	1.392694	5.54	-4179.935416	-4180.151698
**2a**	C79H68N6O4	0	-3681.463250	1.284142	5.08	-3680.099924	-3680.302986
**2a–H⁺**	C79H69N6O4	1	-3681.926203	1.298653	6.42	-3680.548153	-3680.750973
**2a–H_3^{3+}**	C79H71N6O4	3	-3682.770858	1.331048	7.01	-3681.361623	-3681.558868
**2b**	C81H73N7O4	0	-3815.457047	1.357099	6.14	-3814.016031	-3814.231117
**2b–H⁺**	C81H74N7O4	1	-3815.927672	1.37175	5.22	-3814.472033	-3814.685424
**2b—H_4^{4+}**	C81H77N7O4	4	-3817.182534	1.422200	10.63	-3815.679168	-3815.877396
**2c**	C87H77N7O4	0	-4046.547905	1.438233	6.74	-4045.021262	-4045.245386
**2c–H⁺**	C87H78N7O4	1	-4047.013336	1.452513	4.96	-4045.472028	-4045.698496
**2c–H_4^{4+}**	C87H81N7O4	4	-4048.285218	1.502439	5.12	-4046.696404	-4046.909179
**2d**	C79H63F5N6O4	0	-4177.583583	1.243168	6.20	-4176.256507	-4176.469654
**2d–H⁺**	C79H64F5N6O4	1	-4178.042650	1.257258	5.56	-4176.701264	-4176.91405
**2d–H_3^{3+}**	C79H66F5N6O4	3	-4178.883414	1.291116	8.01	-4177.51008	-4177.712942
**2e**	C79H66N8O8	0	-4090.465704	1.288466	5.38	-4089.092588	-4089.309789
**2e–H⁺**	C79H67N8O8	1	-4090.924162	1.303232	6.16	-4089.536322	-4089.750998
**2e–H_3^{3+}**	C79H69N8O8	3	-4091.764458	1.337289	9.75	-4090.344439	-4090.549931

[a] Structure code (see the zip file for Cartesian coordinates). [b] SCF electronic energy. [c] Zero-point vibrational energy. [d] Lowest vibrational frequency. [e] Enthalpy. [f] Gibbs free energy.

Table S12. Energy comparison of protonated dipyrrins.

code	SCF E[a]	ZPV[b]	lowest freq. [c]	H[d]	G[e]	ΔSCFrel
	a.u.	a.u.	cm^{-1}	a.u.	a.u.	kcal/mol
**1a–H⁺** (*E-anti*)	-3413.92816	1.152328	0.95	-3412.70549	-3412.88863	0.00
**1a**–H⁺ (*Z-anti*)	-3413.92772	1.152713	6.14	-3412.70486	-3412.88608	0.28
**1a**–H⁺ (*Z-syn*)	-3413.92647	1.153524	8.81	-3412.70286	-3412.88449	1.06
**2a**–H⁺ (*Z-anti*)	-3681.9262	1.298653	6.42	-3680.54815	-3680.75097	0.00
**2a**–H⁺ (*E-anti*)	-3681.92544	1.298484	7.86	-3680.54756	-3680.74938	0.48
**2a**–H⁺ (*Z-syn*)	-3681.92426	1.299456	8.28	-3680.54564	-3680.74716	1.22
**1b**–H⁺ (*Z-anti*)	-3547.93336	1.226208	7.53	-3546.63255	-3546.82343	0.00
**1b**–H⁺ (*E-anti*)	-3547.93306	1.225909	8.74	-3546.63248	-3546.82327	0.19
**1b**–H⁺ (*Z-syn*)	-3547.9301	1.226338	7.11	-3546.62913	-3546.82071	2.05

[a] SCF electronic energy. [b] Zero-point vibrational energy. [c] Lowest vibrational frequency. [d] Enthalpy. [e] Gibbs free energy.

Table S13. Computational details for the oxidation levels of **2a** and **2d**.

Code	Formula	SCF E[a]	ZPV[a]	lowest freq. [a]	H[a]	G[a]
		a.u.	a.u.	cm–1	a.u.	a.u.
**2a**$^{+\bullet}$	C79H68N6O4	-3681.288496	1.285381	7.18	-3679.924028	-3680.12747
**2a**$^{2+}$	C79H68N6O4	-3681.088678	1.287601	8.59	-3679.722405	-3679.92221
**2a**$^{3+\bullet}$	C79H68N6O4	-3680.853601	1.288692	9.69	-3679.48699	-3679.68307
**2a**–H$^{\bullet 2+}$	C79H69N6O4	-3681.731113	1.299781	6.68	-3680.352185	-3680.5542
**2d**$^{+\bullet}$	C79H63F5N6O4	-4177.40676	1.244716	7.85	-4176.078504	-4176.28926
**2d**$^{2+}$	C79H63F5N6O4	-4177.204676	1.246611	8.59	-4175.874629	-4176.08576
**2d**$^{3+\bullet}$	C79H63F5N6O4	-4176.966229	1.248026	9.12	-4175.635751	-4175.84084
**2d**–H$^{\bullet 2+}$	C79H64F5N6O4	-4177.846533	1.25897	8.56	-4176.503957	-4176.71381

[a] SCF electronic energy. [b] Zero-point vibrational energy. [c] Lowest vibrational frequency. [d] Enthalpy. [e] Gibbs free energy.

Table S14. MO composition analysis of the selected molecular orbitals of **1a**–**1e** obtained at B3LYP-GD3BJ/6-31G(d,p)/PCM(dichloromethane) level of theory.

	**1a**				**1b**				**1c**				**1d**				**1e**			
**MO**	Energy	D	M	A	Energy	D	M	A	Energy	D	M	A	Energy	D	M	A	Energy	D	M	A
**L+4**	-0.78	87	12	1	-0.75	91	6	3	-0.89	15	82	3	-1.1	50	22	28	-2.38	82	2	16
**L+3**	-1.04	63	2	35	-1.01	65	2	33	-1.03	61	6	33	-1.18	20	73	7	-2.85	92	5	3
**L+2**	-2.29	82	1	17	-2.23	80	3	17	-2.26	81	2	17	-2.41	83	2	16	-2.98	6	94	0
**L+1**	-2.77	96	0	4	-2.73	96	0	4	-2.75	96	0	4	-2.85	96	0	3	-3.27	2	97	0
**LUMO**	-3.47	96	1	3	-3.37	92	5	2	-3.44	96	1	3	-3.62	95	2	4	-3.61	94	3	3
**HOMO**	-5.49	82	0	18	-5.4	59	29	12	-5.12	1	99	0	-5.58	81	0	19	-5.59	81	0	19
**H-1**	-6.08	97	0	3	-5.54	32	61	7	-5.47	81	0	18	-6.2	97	0	3	-6.21	96	0	3
**H-2**	-6.41	96	0	4	-5.98	97	0	2	-6.05	97	0	3	-6.47	100	0	0	-6.47	100	0	0
**H-3**	-6.46	100	0	0	-6.37	96	1	3	-6.39	95	0	4	-6.47	100	0	0	-6.47	100	0	0
**H-4**	-6.46	100	0	0	-6.45	100	0	0	-6.45	100	0	0	-6.5	97	0	3	-6.51	96	0	4

In the table, D stands for NMI-dipyrrin, M stands for *meso*-aryl, A stands for alpha subsitituent and their composition values are given in percent.

Table S15. MO composition analysis of the selected molecular orbitals of **1a**–**1e**–H^+ obtained at B3LYP-GD3BJ/6-31G(d,p)/PCM(dichloromethane) level of theory.

	**1a**–H^+				**1b**–H^+				**1c**–H^+				**1d**–H^+				**1e**–H^+			
**MO**	Energy	D	M	A	Energy	D	M	A	Energy	D	M	A	Energy	D	M	A	Energy	D	M	A
**L+4**	-1.57	49	32	19	-1.41	58	20	22	-1.66	38	45	17	-1.82	33	54	13	-3.01	66	22	12
**L+3**	-1.86	69	1	30	-1.73	71	1	28	-1.77	66	7	27	-1.92	69	1	30	-3.24	12	87	1
**L+2**	-3.03	85	4	11	-2.92	83	6	11	-2.96	84	6	10	-3.1	86	4	11	-3.33	96	1	3
**L+1**	-3.25	97	0	3	-3.15	97	0	3	-3.17	96	0	3	-3.31	97	0	3	-3.53	25	75	0
**LUMO**	-4.22	84	10	6	-3.93	76	21	3	-4.06	75	21	4	-4.42	86	7	7	-4.52	76	17	7
**HOMO**	-6.28	76	1	23	-6.11	31	62	8	-5.54	10	88	2	-6.32	75	1	23	-6.36	76	1	23
**H-1**	-6.61	100	0	0	-6.19	76	1	22	-6.2	76	1	23	-6.62	99	0	0	-6.63	99	0	0
**H-2**	-6.64	100	0	0	-6.6	99	0	0	-6.59	100	0	0	-6.65	99	0	0	-6.64	99	0	0
**H-3**	-6.87	100	0	0	-6.61	100	0	0	-6.61	100	0	0	-6.89	100	0	0	-6.89	100	0	0
**H-4**	-6.89	98	1	1	-6.84	100	0	0	-6.79	65	22	13	-6.91	100	0	0	-6.91	100	0	0

In the table, D stands for NMI-dipyrrin, M stands for *meso*-aryl, A stands for alpha subsitituent and their composition values are given in percent.

Table S16. MO composition analysis of the selected molecular orbitals of **2a**–**2e** obtained at B3LYP-GD3BJ/6-31G(d,p)/PCM(dichloromethane) level of theory.

	**2a**				**2b**				**2c**				**2d**				**2e**			
**MO**	Energy	D	M	A	Energy	D	M	A	Energy	D	M	A	Energy	D	M	A	Energy	D	M	A
**L+4**	-0.69	85	14	1	-0.65	97	2	1	-0.78	41	50	9	-0.89	73	9	18	-2.16	81	2	17
**L+3**	-0.83	79	2	19	-0.79	84	1	15	-0.84	47	42	11	-1.06	7	92	1	-2.73	94	2	4
**L+2**	-2.06	81	1	18	-2.02	80	3	17	-2.05	81	2	18	-2.18	81	2	17	-2.9	3	97	0
**L+1**	-2.65	95	0	4	-2.62	95	0	4	-2.64	95	0	4	-2.72	95	0	4	-3.19	1	98	0
**LUMO**	-3.35	96	1	3	-3.27	94	3	3	-3.33	96	1	3	-3.48	94	1	5	-3.47	94	2	4
**HOMO**	-4.87	46	0	54	-4.84	45	1	54	-4.85	46	0	54	-4.92	45	0	55	-4.94	44	0	56
**H-1**	-5.39	18	0	82	-5.26	12	35	53	-5.09	1	99	0	-5.46	18	0	82	-5.44	16	1	83
**H-2**	-5.89	84	0	16	-5.5	13	56	31	-5.37	18	1	82	-5.98	82	0	19	-5.98	80	0	20
**H-3**	-6.08	67	0	33	-5.83	83	2	15	-5.87	85	0	15	-6.17	71	0	29	-6.17	74	0	26
**H-4**	-6.27	98	0	2	-6.01	64	0	34	-6.07	65	0	35	-6.35	99	0	1	-6.36	99	0	1

In the table, D stands for NMI-dipyrrin, M stands for *meso*-aryl, A stands for alpha subsitituent and their composition values are given in percent.

Table S17. MO composition analysis of the selected molecular orbitals of **2a**–**2e**–H^+ obtained at B3LYP-GD3BJ/6-31G(d,p)/PCM(dichloromethane) level of theory.

	**2a**–H^+				**2b**–H^+				**2c**–H^+				**2d**–H^+				**2e**–H^+			
**MO**	Energy	D	M	A	Energy	D	M	A	Energy	D	M	A	Energy	D	M	A	Energy	D	M	A
**L+4**	-1.34	59	31	10	-1.21	71	18	11	-1.44	45	43	12	-1.59	37	53	10	-2.78	70	15	15
**L+3**	-1.58	77	1	22	-1.48	79	2	19	-1.53	69	13	18	-1.64	74	4	22	-3.09	4	95	1
**L+2**	-2.78	82	5	14	-2.7	80	6	14	-2.74	81	7	13	-2.86	82	5	13	-3.18	95	1	4
**L+1**	-3.11	96	0	4	-3.04	97	0	3	-3.06	96	0	3	-3.16	96	0	4	-3.38	28	72	0
**LUMO**	-3.98	84	7	9	-3.79	80	15	5	-3.89	81	12	7	-4.13	84	5	12	-4.25	70	17	13
**HOMO**	-5.4	33	0	66	-5.35	32	0	67	-5.35	7	81	12	-5.44	34	0	65	-5.47	33	0	66
**H-1**	-5.88	17	4	79	-5.59	14	28	58	-5.36	31	5	64	-5.99	18	3	79	-6.02	18	3	79
**H-2**	-6.57	92	0	8	-6.2	22	49	30	-5.85	15	13	72	-6.59	98	0	2	-6.59	99	0	1
**H-3**	-6.61	85	0	14	-6.52	72	0	28	-6.54	76	0	24	-6.63	97	0	3	-6.63	98	0	2
**H-4**	-6.63	90	0	10	-6.58	97	0	3	-6.58	94	0	6	-6.67	72	0	28	-6.7	70	0	30

In the table, D stands for NMI-dipyrrin, M stands for *meso*-aryl, A stands for alpha subsitituent and their composition values are given in percent.

Table S18. Electronic transitions calculated for **1a** using the TD/PCM(dichloromethane)GD3BJ-B3LYP/6-31G(d,p) level of theory.

No.	Energy (cm^{-1})	λ (nm)	f[a]	Major excitations[b]
1	14168	705.8	1.221	HOMO»LUMO (98%)
2	16311	613.1	0.099	H–1»LUMO (95%)
3	18896	529.2	0.065	H–2»LUMO (41%)<br>HOMO»L+1 (50%)
4	19780	505.6	0.256	H–2»LUMO (49%)<br>HOMO»L+1 (46%)
5	21394	467.4	0.004	H–4»LUMO (43%)<br>H–3»LUMO (54%)
6	21699	460.9	0.003	H–4»LUMO (54%)<br>H–3»LUMO (42%)
7	22088	452.7	0.013	H–1»L+1 (72%)<br>H–1»L+2 (12%)
8	22737	439.8	0.001	H–7»LUMO (91%)
9	22810	438.4	0.046	H–8»LUMO (12%)<br>H–1»L+1 (10%)<br>HOMO»L+2 (70%)
10	23105	432.8	0.010	H–5»LUMO (81%)
11	23113	432.7	0.003	H–6»LUMO (87%)
12	24151	414.1	0.098	H–8»LUMO (83%)<br>HOMO»L+2 (11%)
13	24180	413.6	0.042	H–2»L+1 (77%)
14	24998	400.0	0.109	H–11»LUMO (13%)<br>H–9»LUMO (80%)
15	25546	391.5	0.011	H–10»LUMO (91%)
16	25658	389.7	0.075	H–14»LUMO (26%)<br>H–11»LUMO (55%)
17	26036	384.1	0.024	H–14»LUMO (60%)<br>H–11»LUMO (21%)
18	26220	381.4	0.021	H–1»L+1 (12%)<br>H–1»L+2 (74%)
19	26587	376.1	0.000	H–16»LUMO (80%)
20	26749	373.9	0.051	H–12»LUMO (83%)
21	26869	372.2	0.001	H–4»L+1 (48%)<br>H–3»L+1 (44%)
22	26951	371.0	0.000	H–15»LUMO (63%)<br>H–15»L+1 (14%)
23	27158	368.2	0.002	H–13»LUMO (92%)
24	27394	365.0	0.001	H–4»L+1 (45%)<br>H–3»L+1 (47%)
25	27739	360.5	0.002	H–17»LUMO (82%)

[a] Oscillator strength. [b] Contributions smaller than 10% are not included. H = HOMO, L = LUMO. Orbitals are numbered consecutively regardless of possible degeneracies

Table S19. Electronic transitions calculated for **1a**–H^+ using the TD/PCM(dichloromethane)GD3BJ-B3LYP/6-31G(d,p) level of theory.

No.	Energy (cm^{-1})	λ (nm)	f[a]	Major excitations[b]
1	13960	716.3	0.685	HOMO»LUMO (99%)
2	16964	589.5	0.001	H–1»LUMO (99%)
3	17061	586.1	0.004	H–2»LUMO (99%)
4	18175	550.2	0.084	H–6»LUMO (31%)<br>H–5»LUMO (50%)
5	18731	533.9	0.030	H–7»LUMO (19%)<br>H–6»LUMO (37%)<br>H–4»LUMO (33%)
6	18782	532.4	0.002	H–3»LUMO (89%)
7	18880	529.7	0.006	H–7»LUMO (14%)<br>H–6»LUMO (14%)<br>H–5»LUMO (17%)<br>H–4»LUMO (53%)
8	19652	508.9	0.133	H–7»LUMO (49%)<br>H–6»LUMO (10%)<br>H–5»LUMO (27%)<br>HOMO»L+1 (12%)
9	21463	465.9	0.357	HOMO»L+1 (81%)
10	21952	455.5	0.050	H–8»LUMO (78%)<br>HOMO»L+2 (16%)
11	23088	433.1	0.041	H–9»LUMO (88%)
12	23142	432.1	0.103	H–10»LUMO (82%)
13	23592	423.9	0.172	H–11»LUMO (43%)<br>HOMO»L+2 (36%)
14	23664	422.6	0.265	H–11»LUMO (41%)<br>HOMO»L+2 (35%)
15	23969	417.2	0.003	H–12»LUMO (81%)
16	24000	416.7	0.000	H–13»LUMO (87%)
17	24070	415.5	0.001	H–14»LUMO (21%)<br>H–2»L+1 (60%)
18	24131	414.4	0.002	H–14»LUMO (53%)<br>H–2»L+1 (29%)
19	24178	413.6	0.000	H–1»L+1 (79%)<br>H–1»L+2 (17%)
20	24504	408.1	0.014	H–16»LUMO (10%)<br>H–15»LUMO (78%)
21	24810	403.1	0.022	H–16»LUMO (69%)<br>H–15»LUMO (10%)
22	25169	397.3	0.049	H–7»L+1 (10%)<br>H–6»L+2 (10%)<br>H–5»L+1 (51%)
23	25582	390.9	0.005	H–7»L+1 (15%)<br>H–6»L+1 (29%)<br>H–4»L+1 (26%)
24	25726	388.7	0.002	H–7»L+1 (11%)<br>H–5»L+1 (10%)<br>H–4»L+1 (48%)
25	25774	388.0	0.000	H–3»L+1 (70%)<br>H–3»L+2 (21%)

[a] Oscillator strength. [b] Contributions smaller than 10% are not included. H = HOMO, L = LUMO. Orbitals are numbered consecutively regardless of possible degeneracies.

Table S20. Electronic transitions calculated for **1a**–H+ (*Z-anti*) using the TD/PCM(dichloromethane)GD3BJ-B3LYP/6-31G(d,p) level of theory.

No.	Energy (cm^{-1})	λ (nm)	f[a]	Major excitations[b]
1	13460	743.0	0.492	HOMO»LUMO (100%)
2	16707	598.6	0.002	H–1»LUMO (99%)
3	16785	595.8	0.008	H–2»LUMO (99%)
4	17777	562.5	0.018	H–7»LUMO (32%)<br>H–5»LUMO (64%)
5	18210	549.1	0.015	H–6»LUMO (98%)
6	18535	539.5	0.000	H–3»LUMO (98%)
7	18547	539.2	0.003	H–4»LUMO (95%)
8	19047	525.0	0.172	H–7»LUMO (57%)<br>H–5»LUMO (28%)<br>HOMO»L+1 (13%)
9	21457	466.1	0.647	H–7»LUMO (10%)<br>HOMO»L+1 (79%)
10	22163	451.2	0.017	H–8»LUMO (92%)
11	22358	447.3	0.010	H–9»LUMO (86%)<br>HOMO»L+2 (13%)
12	23310	429.0	0.059	H–12»LUMO (51%)<br>HOMO»L+2 (37%)
13	23638	423.0	0.009	H–10»LUMO (77%)
14	23640	423.0	0.006	H–11»LUMO (81%)
15	23779	420.5	0.000	H–15»LUMO (45%)<br>H–13»LUMO (34%)
16	23793	420.3	0.020	H–14»LUMO (74%)
17	23846	419.4	0.469	H–12»LUMO (41%)<br>HOMO»L+2 (43%)
18	23850	419.3	0.009	H–15»LUMO (31%)<br>H–13»LUMO (61%)
19	24055	415.7	0.000	H–1»L+1 (94%)
20	24126	414.5	0.011	H–2»L+1 (92%)
21	24412	409.6	0.011	H–16»LUMO (96%)
22	25038	399.4	0.082	H–7»L+1 (26%)<br>H–5»L+1 (59%)
23	25566	391.1	0.003	H–6»L+1 (75%)
24	25670	389.6	0.000	H–3»L+1 (85%)
25	25682	389.4	0.001	H–4»L+1 (85%)

[a] Oscillator strength. [b] Contributions smaller than 10% are not included. H = HOMO, L = LUMO. Orbitals are numbered consecutively regardless of possible degeneracies.

Table S21. Electronic transitions calculated for **1a**–H^+ (*Z-syn*) using the TD/PCM(dichloromethane)GD3BJ-B3LYP/6-31G(d,p) level of theory.

No.	Energy (cm^{-1})	λ (nm)	f[a]	Major excitations[b]
1	14278	700.4	1.181	HOMO»LUMO (100%)
2	17182	582.0	0.000	H–1»LUMO (99%)
3	17202	581.3	0.001	H–2»LUMO (99%)
4	18456	541.8	0.040	H–7»LUMO (15%)<br>H–5»LUMO (80%)
5	18992	526.5	0.002	H–4»LUMO (97%)
6	18996	526.4	0.000	H–3»LUMO (97%)
7	19344	517.0	0.165	H–6»LUMO (95%)
8	20307	492.4	0.015	H–7»LUMO (67%)<br>H–5»LUMO (17%)<br>HOMO»L+1 (15%)
9	21726	460.3	0.134	H–7»LUMO (15%)<br>HOMO»L+1 (78%)
10	22028	454.0	0.027	H–8»LUMO (86%)
11	23324	428.7	0.054	H–10»LUMO (35%)<br>HOMO»L+2 (56%)
12	23432	426.8	0.354	H–9»LUMO (90%)
13	23806	420.1	0.104	H–10»LUMO (55%)<br>HOMO»L+2 (32%)
14	24150	414.1	0.001	H–14»LUMO (54%)<br>H–12»LUMO (35%)
15	24151	414.1	0.000	H–15»LUMO (52%)<br>H–13»LUMO (26%)<br>H–11»LUMO (11%)
16	24221	412.9	0.044	H–11»LUMO (82%)
17	24299	411.5	0.003	H–14»LUMO (33%)<br>H–12»LUMO (64%)
18	24331	411.0	0.004	H–15»LUMO (28%)<br>H–13»LUMO (69%)
19	24359	410.5	0.001	H–2»L+2 (13%)<br>H–1»L+1 (85%)
20	24372	410.3	0.000	H–2»L+1 (85%)<br>H–1»L+2 (13%)
21	25202	396.8	0.007	H–18»LUMO (17%)<br>H–16»LUMO (71%)
22	25536	391.6	0.023	H–7»L+1 (10%)<br>H–6»L+2 (12%)<br>H–5»L+1 (70%)
23	25961	385.2	0.001	H–4»L+1 (76%)<br>H–3»L+2 (16%)
24	25966	385.1	0.000	H–4»L+2 (16%)<br>H–3»L+1 (77%)
25	26153	382.4	0.057	H–6»L+1 (57%)<br>H–5»L+2 (22%)

[a] Oscillator strength. [b] Contributions smaller than 10% are not included. H = HOMO, L = LUMO. Orbitals are numbered consecutively regardless of possible degeneracies.

Table S22. Electronic transitions calculated for **1b** using the TD/PCM(dichloromethane)GD3BJ-B3LYP/6-31G(d,p) level of theory.

No.	Energy (cm^{-1})	λ (nm)	f[a]	Major excitations[b]
1	13583	736.2	0.299	H–1»LUMO (27%)<br>HOMO»LUMO (73%)
2	14570	686.3	0.940	H–1»LUMO (72%)<br>HOMO»LUMO (26%)
3	16288	614.0	0.077	H–2»LUMO (94%)
4	18370	544.4	0.060	H–1»L+1 (14%)<br>HOMO»L+1 (83%)
5	19290	518.4	0.055	H–3»LUMO (33%)<br>H–1»L+1 (55%)
6	20040	499.0	0.193	H–3»LUMO (55%)<br>H–1»L+1 (29%)
7	21628	462.4	0.008	H–2»L+1 (75%)<br>H–2»L+2 (13%)
8	22127	451.9	0.004	H–4»LUMO (85%)
9	22345	447.5	0.086	HOMO»L+2 (69%)
10	22442	445.6	0.011	H–5»LUMO (88%)
11	23110	432.7	0.041	H–6»LUMO (26%)<br>H–1»L+2 (55%)
12	23404	427.3	0.000	H–7»LUMO (91%)
13	23640	423.0	0.128	H–6»LUMO (58%)<br>H–1»L+2 (32%)
14	23774	420.6	0.000	H–8»LUMO (90%)
15	24212	413.0	0.044	H–3»L+1 (67%)
16	24277	411.9	0.069	H–9»LUMO (75%)
17	25258	395.9	0.128	H–12»LUMO (15%)<br>H–10»LUMO (75%)
18	25638	390.0	0.017	H–2»L+1 (13%)<br>H–2»L+2 (76%)
19	26023	384.3	0.022	H–11»LUMO (85%)
20	26298	380.3	0.061	H–12»LUMO (70%)<br>H–10»LUMO (15%)
21	27059	369.6	0.004	H–15»LUMO (73%)<br>H–15»L+1 (10%)
22	27120	368.7	0.044	H–13»LUMO (80%)
23	27193	367.7	0.002	H–5»L+1 (85%)
24	27425	364.6	0.000	H–16»LUMO (57%)<br>H–16»L+1 (16%)
25	27649	361.7	0.002	H–4»L+1 (86%)

[a] Oscillator strength. [b] Contributions smaller than 10% are not included. H = HOMO, L = LUMO. Orbitals are numbered consecutively regardless of possible degeneracies.

Table S23. Electronic transitions calculated for **1b**–H^+ using the TD/PCM(dichloromethane)GD3BJ-/6-31G(d,p) level of theory.

No.	Energy (cm^{-1})	λ (nm)	f[a]	Major excitations[b]
1	14977	667.7	0.503	H–1»LUMO (99%)
2	15899	629.0	0.681	HOMO»LUMO (99%)
3	19088	523.9	0.003	H–2»LUMO (92%)
4	19128	522.8	0.002	H–3»LUMO (94%)
5	19344	517.0	0.026	H–6»LUMO (87%)
6	19949	501.3	0.020	H–7»LUMO (75%)
7	20683	483.5	0.083	H–1»L+1 (11%)<br>HOMO»L+1 (82%)
8	20796	480.9	0.000	H–4»LUMO (93%)
9	20802	480.7	0.002	H–5»LUMO (87%)
10	21315	469.2	0.257	H–1»L+1 (72%)<br>HOMO»L+1 (10%)
11	22372	447.0	0.088	H–8»LUMO (28%)<br>HOMO»L+2 (64%)
12	23026	434.3	0.038	H–9»LUMO (36%)<br>H–1»L+2 (60%)
13	23613	423.5	0.409	H–8»LUMO (51%)<br>HOMO»L+2 (31%)
14	24047	415.9	0.306	H–9»LUMO (60%)<br>H–1»L+2 (33%)
15	24699	404.9	0.000	H–3»L+1 (89%)
16	24790	403.4	0.023	H–10»LUMO (95%)
17	24935	401.0	0.001	H–2»L+1 (80%)<br>H–2»L+2 (16%)
18	25109	398.3	0.016	H–6»L+1 (68%)<br>H–6»L+2 (20%)
19	25306	395.2	0.032	H–13»LUMO (28%)<br>H–12»LUMO (39%)<br>H–7»L+1 (20%)
20	25383	394.0	0.010	H–13»LUMO (23%)<br>H–7»L+1 (46%)
21	25489	392.3	0.001	H–16»LUMO (13%)<br>H–14»LUMO (45%)
22	25498	392.2	0.000	H–16»LUMO (12%)<br>H–15»LUMO (26%)<br>H–14»LUMO (26%)
23	25672	389.5	0.000	H–11»LUMO (90%)
24	25768	388.1	0.035	H–13»LUMO (43%)<br>H–12»LUMO (44%)
25	26111	383.0	0.009	H–16»LUMO (44%)<br>H–15»LUMO (47%)

[a] Oscillator strength. [b] Contributions smaller than 10% are not included. H = HOMO, L = LUMO. Orbitals are numbered consecutively regardless of possible degeneracies.

Table S24. Electronic transitions calculated for **1b**–H^+ (*Z-anti*) using the TD/PCM(dichloromethane) GD3BJ-B3LYP/6-31G(d,p) level of theory.

No.	Energy (cm^{-1})	λ (nm)	f[a]	Major excitations[b]
1	14388	695.0	0.337	H–1»LUMO (99%)
2	15859	630.5	0.880	HOMO»LUMO (99%)
3	18898	529.1	0.002	H–2»LUMO (96%)
4	18978	526.9	0.000	H–3»LUMO (98%)
5	19193	521.0	0.005	H–7»LUMO (92%)
6	19218	520.4	0.041	H–6»LUMO (81%)
7	20633	484.7	0.000	H–4»LUMO (97%)
8	20640	484.5	0.030	H–5»LUMO (84%)
9	20682	483.5	0.298	H–8»LUMO (12%)<br>H–5»LUMO (11%)<br>H–1»L+1 (70%)
10	20702	483.0	0.057	HOMO»L+1 (96%)
11	22274	449.0	0.154	H–8»LUMO (65%)<br>H–1»L+1 (15%)<br>HOMO»L+2 (14%)
12	23439	426.6	0.179	H–9»LUMO (17%)<br>H–1»L+2 (81%)
13	23529	425.0	0.004	HOMO»L+2 (84%)
14	24358	410.5	0.206	H–11»LUMO (10%)<br>H–9»LUMO (71%)<br>H–1»L+2 (13%)
15	24460	408.8	0.000	H–2»L+1 (94%)
16	24534	407.6	0.006	H–3»L+1 (92%)
17	24690	405.0	0.077	H–6»L+1 (77%)
18	24867	402.1	0.004	H–7»L+1 (78%)
19	24931	401.1	0.149	H–11»LUMO (84%)
20	25298	395.3	0.000	H–16»LUMO (22%)<br>H–12»LUMO (56%)
21	25298	395.3	0.001	H–15»LUMO (26%)<br>H–13»LUMO (58%)
22	25319	395.0	0.029	H–14»LUMO (17%)<br>H–10»LUMO (69%)
23	25387	393.9	0.003	H–16»LUMO (35%)<br>H–12»LUMO (42%)<br>H–4»L+1 (10%)
24	25393	393.8	0.000	H–15»LUMO (39%)<br>H–13»LUMO (41%)<br>H–5»L+1 (11%)
25	25573	391.0	0.000	H–14»LUMO (72%)<br>H–10»LUMO (10%)

[a] Oscillator strength. [b] Contributions smaller than 10% are not included. H = HOMO, L = LUMO. Orbitals are numbered consecutively regardless of possible degeneracies.

Table S25. Electronic transitions calculated for **1b**–H^+ (*Z-syn*) using the TD/PCM(dichloromethane) GD3BJ-B3LYP/6-31G(d,p) level of theory.

No.	Energy (cm^{-1})	λ (nm)	f[a]	Major excitations[b]
1	14945	669.1	0.993	H–1»LUMO (100%)
2	15396	649.5	0.289	HOMO»LUMO (100%)
3	19097	523.6	0.002	H–2»LUMO (97%)
4	19106	523.4	0.001	H–3»LUMO (97%)
5	19411	515.2	0.002	H–6»LUMO (86%)
6	20084	497.9	0.002	H–7»LUMO (58%)<br>HOMO»L+1 (38%)
7	20624	484.9	0.260	H–7»LUMO (35%)<br>HOMO»L+1 (60%)
8	20799	480.8	0.003	H–4»LUMO (92%)
9	20808	480.6	0.001	H–5»LUMO (93%)
10	21250	470.6	0.095	H–8»LUMO (10%)<br>H–1»L+1 (82%)
11	22271	449.0	0.009	H–8»LUMO (33%)<br>HOMO»L+2 (57%)
12	23106	432.8	0.062	H–9»LUMO (27%)<br>H–1»L+2 (71%)
13	23675	422.4	0.683	H–8»LUMO (47%)<br>HOMO»L+2 (41%)
14	23992	416.8	0.131	H–9»LUMO (67%)<br>H–1»L+2 (25%)
15	24959	400.7	0.000	H–3»L+2 (11%)<br>H–2»L+1 (82%)
16	24969	400.5	0.000	H–3»L+1 (83%)<br>H–2»L+2 (11%)
17	25106	398.3	0.038	H–11»LUMO (76%)<br>H–6»L+1 (12%)
18	25259	395.9	0.002	H–14»LUMO (13%)<br>H–10»LUMO (78%)
19	25261	395.9	0.001	H–11»LUMO (13%)<br>H–7»L+2 (11%)<br>H–6»L+1 (65%)
20	25491	392.3	0.000	H–15»LUMO (63%)
21	25497	392.2	0.000	H–16»LUMO (62%)
22	25717	388.8	0.006	H–14»LUMO (50%)<br>H–7»L+1 (26%)<br>H–6»L+2 (15%)
23	25755	388.3	0.003	H–12»LUMO (91%)
24	25770	388.0	0.000	H–13»LUMO (90%)
25	25902	386.1	0.024	H–14»LUMO (32%)<br>H–7»L+1 (35%)<br>H–6»L+2 (16%)

[a] Oscillator strength. [b] Contributions smaller than 10% are not included. H = HOMO, L = LUMO. Orbitals are numbered consecutively regardless of possible degeneracies.

Table S26. Electronic transitions calculated for **1b**–2H^{2+} using the TD/PCM(dichloromethane)GD3BJ-B3LYP/6-31G(d,p) level of theory.

No.	Energy (cm^{-1})	λ (nm)	f[a]	Major excitations[b]
1	13343	749.5	0.729	HOMO»LUMO (99%)
2	15275	654.7	0.005	H–1»LUMO (99%)
3	15520	644.3	0.001	H–2»LUMO (99%)
4	17142	583.4	0.000	H–3»LUMO (98%)
5	17436	573.5	0.001	H–4»LUMO (98%)
6	17681	565.6	0.062	H–7»LUMO (13%)<br>H–5»LUMO (74%)
7	18189	549.8	0.076	H–7»LUMO (13%)<br>H–6»LUMO (82%)
8	19616	509.8	0.091	H–7»LUMO (61%)<br>H–5»LUMO (19%)<br>HOMO»L+1 (12%)
9	21167	472.4	0.036	H–8»LUMO (88%)<br>HOMO»L+2 (10%)
10	21556	463.9	0.369	H–7»LUMO (10%)<br>HOMO»L+1 (84%)
11	22050	453.5	0.012	H–9»LUMO (98%)
12	22833	438.0	0.058	H–12»LUMO (31%)<br>H–11»LUMO (11%)<br>H–10»LUMO (52%)
13	22846	437.7	0.039	H–12»LUMO (28%)<br>H–11»LUMO (19%)<br>H–10»LUMO (39%)
14	22984	435.1	0.000	H–12»LUMO (26%)<br>H–11»LUMO (69%)
15	23117	432.6	0.038	H–14»LUMO (81%)
16	23137	432.2	0.032	H–13»LUMO (69%)<br>HOMO»L+2 (17%)
17	23166	431.7	0.227	H–15»LUMO (29%)<br>H–13»LUMO (14%)<br>HOMO»L+2 (30%)
18	23234	430.4	0.008	H–1»L+1 (80%)<br>H–1»L+2 (12%)
19	23427	426.9	0.073	H–15»LUMO (31%)<br>H–2»L+1 (43%)<br>HOMO»L+2 (11%)
20	23467	426.1	0.103	H–15»LUMO (27%)<br>H–2»L+1 (39%)<br>HOMO»L+2 (19%)
21	24851	402.4	0.000	H–3»L+1 (79%)<br>H–3»L+2 (16%)
22	25097	398.5	0.000	H–4»L+1 (75%)<br>H–4»L+2 (20%)
23	25328	394.8	0.037	H–17»LUMO (19%)<br>H–6»L+2 (12%)<br>H–5»L+1 (54%)
24	25481	392.4	0.021	H–16»LUMO (68%)<br>H–6»L+1 (14%)
25	25691	389.2	0.000	H–1»L+1 (13%)<br>H–1»L+2 (85%)

[a] Oscillator strength. [b] Contributions smaller than 10% are not included. H = HOMO, L = LUMO. Orbitals are numbered consecutively regardless of possible degeneracies.

Table S27. Electronic transitions calculated for **1c** using the TD/PCM(dichloromethane)GD3BJ-B3LYP/6-31G(d,p) level of theory.

No.	Energy (cm^{-1})	λ (nm)	f[a]	Major excitations[b]
1	10726	932.4	0.022	HOMO»LUMO (100%)
2	14211	703.7	1.200	H–1»LUMO (98%)
3	16317	612.9	0.082	H–2»LUMO (89%)
4	16438	608.4	0.014	HOMO»L+1 (94%)
5	18929	528.3	0.066	H–3»LUMO (37%)<br>H–1»L+1 (55%)
6	19805	504.9	0.231	H–3»LUMO (53%)<br>H–1»L+1 (41%)
7	20472	488.5	0.039	HOMO»L+2 (98%)
8	21566	463.7	0.004	H–5»LUMO (12%)<br>H–4»LUMO (85%)
9	21879	457.1	0.003	H–5»LUMO (82%)<br>H–4»LUMO (11%)
10	22046	453.6	0.012	H–2»L+1 (71%)<br>H–2»L+2 (12%)
11	22775	439.1	0.041	H–9»LUMO (10%)<br>H–6»LUMO (19%)<br>H–1»L+2 (55%)
12	22885	437.0	0.000	H–7»LUMO (90%)
13	22955	435.6	0.010	H–6»LUMO (64%)<br>H–1»L+2 (24%)
14	23271	429.7	0.000	H–8»LUMO (90%)
15	24128	414.5	0.061	H–9»LUMO (77%)<br>H–1»L+2 (11%)
16	24190	413.4	0.038	H–3»L+1 (77%)
17	24594	406.6	0.048	H–12»LUMO (12%)<br>H–11»LUMO (44%)<br>H–10»LUMO (36%)
18	24644	405.8	0.009	H–12»LUMO (15%)<br>H–11»LUMO (20%)<br>H–10»LUMO (58%)
19	25012	399.8	0.095	H–14»LUMO (14%)<br>H–12»LUMO (58%)<br>H–11»LUMO (19%)
20	25686	389.3	0.002	H–13»LUMO (95%)
21	25898	386.1	0.095	H–14»LUMO (74%)
22	26146	382.5	0.019	H–2»L+1 (12%)<br>H–2»L+2 (73%)
23	26670	375.0	0.000	H–18»LUMO (81%)
24	26786	373.3	0.069	H–15»LUMO (89%)
25	26980	370.6	0.002	H–5»L+1 (77%)<br>H–4»L+1 (14%)

[a] Oscillator strength. [b] Contributions smaller than 10% are not included. H = HOMO, L = LUMO. Orbitals are numbered consecutively regardless of possible degeneracies.

Table S28. Electronic transitions calculated for **1c**–H$^+$ using the TD/PCM(dichloromethane)GD3BJ-B3LYP/6-31G(d,p) level of theory.

No.	Energy (cm^{-1})	λ (nm)	f[a]	Major excitations[b]
1	10856	921.1	0.824	HOMO»LUMO (101%)
2	14324	698.1	0.568	H–1»LUMO (99%)
3	16759	596.7	0.038	HOMO»L+1 (98%)
4	18062	553.6	0.005	H–2»LUMO (95%)
5	18177	550.1	0.011	H–3»LUMO (95%)
6	18357	544.7	0.194	HOMO»L+2 (82%)
7	18543	539.3	0.039	H–7»LUMO (49%)<br>H–4»LUMO (42%)
8	19208	520.6	0.054	H–8»LUMO (41%)<br>H–7»LUMO (35%)<br>H–4»LUMO (16%)
9	19827	504.4	0.000	H–5»LUMO (97%)
10	19916	502.1	0.000	H–6»LUMO (95%)
11	19980	500.5	0.017	H–8»LUMO (40%)<br>H–4»LUMO (26%)<br>H–1»L+1 (18%)
12	21516	464.8	0.367	H–8»LUMO (10%)<br>H–1»L+1 (77%)
13	22424	446.0	0.001	H–9»LUMO (66%)<br>H–1»L+2 (32%)
14	23224	430.6	0.013	H–10»LUMO (93%)
15	23509	425.4	0.367	H–12»LUMO (10%)<br>H–9»LUMO (25%)<br>H–1»L+2 (53%)
16	23615	423.5	0.045	H–11»LUMO (89%)
17	23958	417.4	0.050	H–12»LUMO (86%)
18	24334	411.0	0.054	H–13»LUMO (90%)
19	24495	408.2	0.001	H–3»L+1 (89%)
20	24711	404.7	0.001	H–2»L+1 (75%)<br>H–2»L+2 (20%)
21	24763	403.8	0.000	H–15»LUMO (71%)<br>H–14»LUMO (12%)
22	24770	403.7	0.000	H–15»LUMO (12%)<br>H–14»LUMO (74%)
23	24881	401.9	0.034	H–8»L+1 (11%)<br>H–7»L+1 (14%)<br>H–7»L+2 (13%)<br>H–4»L+1 (51%)
24	24892	401.7	0.000	H–17»LUMO (11%)<br>H–16»LUMO (60%)<br>H–14»LUMO (10%)
25	25252	396.0	0.008	H–8»L+1 (28%)<br>H–7»L+1 (29%)

[a] Oscillator strength. [b] Contributions smaller than 10% are not included. H = HOMO, L = LUMO. Orbitals are numbered consecutively regardless of possible degeneracies.

Table S29. Electronic transitions calculated for **1c**–$2H^{2+}$ using the TD/PCM(dichloromethane)GD3BJ-B3LYP/6-31G(d,p) level of theory.

No.	Energy (cm^{-1})	λ (nm)	f[a]	Major excitations[b]
1	13709	729.4	0.687	HOMO»LUMO (99%)
2	16346	611.8	0.005	H–1»LUMO (99%)
3	16595	602.6	0.001	H–2»LUMO (99%)
4	17860	559.9	0.157	H–6»LUMO (20%)<br>H–5»LUMO (62%)
5	18183	550.0	0.009	H–3»LUMO (88%)
6	18435	542.4	0.003	H–4»LUMO (93%)
7	18505	540.4	0.055	H–7»LUMO (19%)<br>H–6»LUMO (62%)<br>H–5»LUMO (11%)
8	19033	525.4	0.275	H–7»LUMO (68%)<br>H–5»LUMO (17%)
9	21204	471.6	0.270	H–9»LUMO (13%)<br>HOMO»L+1 (81%)
10	21656	461.8	0.039	H–8»LUMO (84%)<br>HOMO»L+2 (14%)
11	22450	445.4	0.202	H–9»LUMO (83%)<br>HOMO»L+1 (10%)
12	22726	440.0	0.040	H–10»LUMO (95%)
13	23370	427.9	0.353	H–8»LUMO (12%)<br>HOMO»L+2 (72%)
14	23554	424.6	0.079	H–12»LUMO (90%)
15	23588	424.0	0.000	H–14»LUMO (36%)<br>H–13»LUMO (14%)<br>H–11»LUMO (36%)
16	23690	422.1	0.005	H–11»LUMO (40%)<br>H–1»L+1 (43%)
17	23718	421.6	0.004	H–14»LUMO (19%)<br>H–11»LUMO (22%)<br>H–1»L+1 (41%)
18	23802	420.1	0.001	H–14»LUMO (25%)<br>H–13»LUMO (63%)
19	24035	416.1	0.000	H–2»L+1 (80%)<br>H–2»L+2 (18%)
20	24097	415.0	0.015	H–15»LUMO (91%)
21	24953	400.8	0.013	H–16»LUMO (74%)<br>H–5»L+1 (10%)
22	25139	397.8	0.064	H–16»LUMO (14%)<br>H–6»L+1 (18%)<br>H–5»L+1 (36%)<br>H–3»L+1 (10%)
23	25321	394.9	0.003	H–3»L+1 (68%)<br>H–3»L+2 (12%)
24	25626	390.2	0.001	H–4»L+1 (66%)<br>H–4»L+2 (21%)
25	25674	389.5	0.009	H–7»L+1 (31%)<br>H–6»L+1 (20%)<br>H–5»L+2 (13%)

[a] Oscillator strength. [b] Contributions smaller than 10% are not included. H = HOMO, L = LUMO. Orbitals are numbered consecutively regardless of possible degeneracies.

Table S30. Electronic transitions calculated for **1d** using the TD/PCM(dichloromethane)GD3BJ-B3LYP/6-31G(d,p) level of theory.

No.	Energy (cm^{-1})	λ (nm)	f[a]	Major excitations[b]
1	13841	722.5	1.211	HOMO»LUMO (98%)
2	16015	624.4	0.091	H–1»LUMO (96%)
3	18665	535.8	0.033	H–4»LUMO (63%)<br>HOMO»L+1 (27%)
4	19669	508.4	0.294	H–4»LUMO (26%)<br>HOMO»L+1 (68%)
5	20313	492.3	0.006	H–3»LUMO (84%)<br>H–2»LUMO (10%)
6	20619	485.0	0.002	H–3»LUMO (11%)<br>H–2»LUMO (85%)
7	21779	459.2	0.000	H–6»LUMO (95%)
8	22143	451.6	0.002	H–5»LUMO (89%)
9	22158	451.3	0.025	H–1»L+1 (46%)<br>H–1»L+2 (10%)<br>HOMO»L+2 (27%)
10	22659	441.3	0.028	H–8»LUMO (15%)<br>H–1»L+1 (30%)<br>HOMO»L+2 (48%)
11	23019	434.4	0.014	H–7»LUMO (84%)
12	23713	421.7	0.148	H–8»LUMO (77%)<br>HOMO»L+2 (14%)
13	24113	414.7	0.033	H–4»L+1 (73%)
14	24490	408.3	0.073	H–11»LUMO (20%)<br>H–9»LUMO (69%)
15	24678	405.2	0.002	H–10»LUMO (97%)
16	25048	399.2	0.097	H–11»LUMO (69%)<br>H–9»LUMO (23%)
17	25904	386.0	0.000	H–15»LUMO (84%)
18	26115	382.9	0.003	H–12»LUMO (94%)
19	26179	382.0	0.040	H–13»LUMO (74%)<br>H–1»L+2 (11%)
20	26261	380.8	0.001	H–2»L+1 (82%)
21	26312	380.1	0.000	H–14»LUMO (70%)<br>H–14»L+1 (12%)
22	26357	379.4	0.038	H–13»LUMO (13%)<br>H–1»L+1 (13%)<br>H–1»L+2 (63%)
23	26822	372.8	0.001	H–3»L+1 (81%)
24	26846	372.5	0.002	H–16»LUMO (80%)
25	27476	364.0	0.001	H–23»LUMO (11%)<br>H–21»LUMO (45%)<br>H–20»LUMO (36%)

[a] Oscillator strength. [b] Contributions smaller than 10% are not included. H = HOMO, L = LUMO. Orbitals are numbered consecutively regardless of possible degeneracies.

Table S31. Electronic transitions calculated for **1d**–H+ using the TD/PCM(dichloromethane)GD3BJ-B3LYP/6-31G(d,p) level of theory.

No.	Energy (cm−1)	λ (nm)	f[a]	Major excitations[b]
1	13142	760.9	0.784	HOMO»LUMO (100%)
2	15494	645.4	0.001	H–1»LUMO (99%)
3	15600	641.0	0.004	H–2»LUMO (99%)
4	17366	575.8	0.012	H–5»LUMO (11%)<br>H–3»LUMO (83%)
5	17416	574.2	0.027	H–5»LUMO (39%)<br>H–4»LUMO (34%)<br>H–3»LUMO (14%)
6	17473	572.3	0.025	H–5»LUMO (26%)<br>H–4»LUMO (62%)
7	17931	557.7	0.068	H–7»LUMO (12%)<br>H–6»LUMO (81%)
8	19540	511.8	0.080	H–7»LUMO (64%)<br>H–5»LUMO (16%)<br>HOMO»L+1 (13%)
9	21054	475.0	0.044	H–8»LUMO (85%)
10	21400	467.3	0.337	H–7»LUMO (10%)<br>HOMO»L+1 (78%)
11	21973	455.1	0.045	H–16»LUMO (16%)<br>H–11»LUMO (42%)<br>H–10»LUMO (10%)<br>H–9»LUMO (24%)
12	22167	451.1	0.013	H–10»LUMO (80%)<br>H–9»LUMO (16%)
13	22524	444.0	0.127	H–11»LUMO (38%)<br>H–9»LUMO (52%)
14	22753	439.5	0.001	H–12»LUMO (93%)
15	22915	436.4	0.000	H–13»LUMO (92%)
16	23000	434.8	0.002	H–14»LUMO (89%)
17	23152	431.9	0.226	H–15»LUMO (57%)<br>HOMO»L+2 (34%)
18	23200	431.0	0.049	H–16»LUMO (46%)<br>H–15»LUMO (14%)<br>HOMO»L+2 (19%)
19	23465	426.2	0.213	H–16»LUMO (32%)<br>H–15»LUMO (24%)<br>HOMO»L+2 (31%)
20	23695	422.0	0.001	H–2»L+1 (84%)<br>H–2»L+2 (10%)
21	23715	421.7	0.001	H–1»L+1 (76%)<br>H–1»L+2 (18%)
22	25254	396.0	0.014	H–5»L+1 (27%)<br>H–4»L+1 (43%)
23	25306	395.2	0.014	H–5»L+1 (18%)<br>H–4»L+1 (34%)<br>H–3»L+1 (17%)
24	25335	394.7	0.007	H–5»L+1 (11%)<br>H–3»L+1 (54%)<br>H–3»L+2 (16%)
25	25459	392.8	0.019	H–18»LUMO (14%)<br>H–17»LUMO (46%)<br>H–6»L+1 (17%)

[a] Oscillator strength. [b] Contributions smaller than 10% are not included. H = HOMO, L = LUMO. Orbitals are numbered consecutively regardless of possible degeneracies.

Table S32. Electronic transitions calculated for **1e** using the TD/PCM(dichloromethane)GD3BJ-B3LYP/6-31G(d,p) level of theory.

No.	Energy (cm^{-1})	λ (nm)	f[a]	Major excitations[b]
1	13908	719.0	1.158	HOMO»LUMO (98%)
2	14702	680.2	0.027	HOMO»L+1 (95%)
3	16181	618.0	0.086	H–1»LUMO (95%)
4	17379	575.4	0.027	HOMO»L+2 (95%)
5	18776	532.6	0.035	H–4»LUMO (58%)<br>HOMO»L+3 (32%)
6	19392	515.7	0.001	H–1»L+1 (94%)
7	19732	506.8	0.279	H–4»LUMO (30%)<br>HOMO»L+3 (62%)
8	20407	490.0	0.005	H–3»LUMO (59%)<br>H–2»LUMO (35%)
9	20731	482.4	0.002	H–3»LUMO (38%)<br>H–2»LUMO (58%)
10	21871	457.2	0.000	H–6»LUMO (94%)
11	22033	453.9	0.001	H–4»L+1 (21%)<br>H–1»L+2 (73%)
12	22081	452.9	0.001	H–4»L+1 (68%)<br>H–1»L+2 (20%)
13	22257	449.3	0.002	H–5»LUMO (87%)
14	22268	449.1	0.023	H–7»LUMO (10%)<br>H–1»L+3 (46%)<br>HOMO»L+4 (16%)
15	22781	439.0	0.020	H–7»LUMO (44%)<br>H–1»L+3 (24%)<br>HOMO»L+4 (14%)
16	22917	436.3	0.030	H–7»LUMO (36%)<br>HOMO»L+4 (52%)
17	23615	423.5	0.000	H–7»L+1 (14%)<br>H–2»L+1 (79%)
18	23715	421.7	0.000	H–3»L+1 (98%)
19	23907	418.3	0.155	H–8»LUMO (75%)<br>HOMO»L+4 (12%)
20	24000	416.7	0.019	H–4»L+2 (34%)<br>H–4»L+3 (42%)
21	24123	414.5	0.015	H–7»L+1 (71%)<br>H–2»L+1 (12%)
22	24750	404.0	0.002	H–10»LUMO (87%)<br>H–9»LUMO (10%)
23	24864	402.2	0.092	H–10»LUMO (11%)<br>H–9»LUMO (78%)
24	24898	401.6	0.004	H–4»L+2 (52%)<br>H–4»L+3 (31%)
25	25506	392.1	0.000	H–5»L+1 (99%)

[a] Oscillator strength. [b] Contributions smaller than 10% are not included. H = HOMO, L = LUMO. Orbitals are numbered consecutively regardless of possible degeneracies.

Table S33. Electronic transitions calculated for **1e**–H$^+$ using the TD/PCM(dichloromethane)GD3BJ-B3LYP/6-31G(d,p) level of theory.

No.	Energy (cm^{-1})	λ (nm)	f[a]	Major excitations[b]
1	12615	792.7	0.689	HOMO»LUMO (100%)
2	14691	680.7	0.001	H–1»LUMO (99%)
3	14783	676.5	0.004	H–2»LUMO (99%)
4	16620	601.7	0.001	H–3»LUMO (99%)
5	16699	598.8	0.000	H–4»LUMO (98%)
6	17001	588.2	0.044	H–7»LUMO (10%)<br>H–5»LUMO (82%)
7	17451	573.0	0.052	H–6»LUMO (90%)
8	19008	526.1	0.110	H–7»LUMO (10%)<br>HOMO»L+1 (80%)
9	19118	523.1	0.115	H–7»LUMO (67%)<br>H–5»LUMO (11%)<br>HOMO»L+1 (13%)
10	20532	487.0	0.060	H–8»LUMO (89%)
11	21437	466.5	0.223	HOMO»L+2 (49%)<br>HOMO»L+3 (36%)
12	21634	462.2	0.125	H–10»LUMO (39%)<br>HOMO»L+2 (19%)<br>HOMO»L+3 (34%)
13	21708	460.7	0.062	H–10»LUMO (57%)<br>HOMO»L+2 (20%)<br>HOMO»L+3 (17%)
14	21834	458.0	0.002	H–9»LUMO (99%)
15	22187	450.7	0.048	H–12»LUMO (71%)<br>H–11»LUMO (23%)
16	22282	448.8	0.052	H–13»LUMO (12%)<br>H–12»LUMO (22%)<br>H–11»LUMO (60%)
17	22331	447.8	0.010	H–13»LUMO (80%)<br>H–11»LUMO (11%)
18	22379	446.9	0.001	H–14»LUMO (92%)
19	22558	443.3	0.000	H–1»L+1 (79%)<br>H–1»L+2 (16%)
20	22580	442.9	0.016	H–15»LUMO (93%)
21	22841	437.8	0.000	H–2»L+1 (70%)<br>H–2»L+2 (25%)
22	23910	418.2	0.006	H–1»L+1 (19%)<br>H–1»L+2 (68%)
23	24037	416.0	0.007	H–2»L+1 (28%)<br>H–2»L+2 (66%)
24	24147	414.1	0.391	HOMO»L+4 (87%)
25	24342	410.8	0.000	H–3»L+1 (65%)<br>H–3»L+2 (26%)

[a] Oscillator strength. [b] Contributions smaller than 10% are not included. H = HOMO, L = LUMO. Orbitals are numbered consecutively regardless of possible degeneracies.

Table S34. Electronic transitions calculated for **1f** using the TD/PCM(dichloromethane)GD3BJ-B3LYP/6-31G(d,p) level of theory.

No.	Energy (cm^{-1})	λ (nm)	f[a]	Major excitations[b]
1	10013	998.7	0.010	HOMO»LUMO (100%)
2	14029	712.8	1.086	H–1»LUMO (98%)
3	15564	642.5	0.009	HOMO»L+1 (98%)
4	16193	617.5	0.055	H–2»LUMO (95%)
5	18694	534.9	0.000	H–3»LUMO (98%)
6	18812	531.6	0.088	H–4»LUMO (27%)<br>H–1»L+1 (63%)
7	19680	508.1	0.122	H–4»LUMO (43%)<br>H–1»L+1 (20%)<br>HOMO»L+2 (32%)
8	19698	507.7	0.115	H–4»LUMO (18%)<br>H–1»L+1 (13%)<br>HOMO»L+2 (66%)
9	21340	468.6	0.003	H–6»LUMO (23%)<br>H–5»LUMO (73%)
10	21637	462.2	0.003	H–6»LUMO (69%)<br>H–5»LUMO (20%)
11	21886	456.9	0.017	H–7»LUMO (10%)<br>H–2»L+1 (62%)<br>H–2»L+2 (11%)
12	22214	450.2	0.004	H–7»LUMO (70%)<br>H–2»L+1 (12%)
13	22646	441.6	0.040	H–8»LUMO (14%)<br>H–1»L+2 (63%)
14	22679	440.9	0.004	H–9»LUMO (73%)
15	22814	438.3	0.006	H–8»LUMO (82%)
16	23075	433.4	0.000	H–10»LUMO (81%)<br>H–9»LUMO (10%)
17	23961	417.3	0.058	H–11»LUMO (76%)<br>H–1»L+2 (10%)
18	24135	414.3	0.029	H–4»L+1 (66%)
19	24238	412.6	0.003	H–3»L+1 (88%)
20	24259	412.2	0.285	HOMO»L+3 (91%)
21	24900	401.6	0.031	H–13»LUMO (84%)
22	25048	399.2	0.114	H–12»LUMO (85%)
23	25340	394.6	0.002	H–14»LUMO (91%)
24	25902	386.1	0.067	H–15»LUMO (33%)<br>H–2»L+2 (47%)
25	26005	384.5	0.029	H–15»LUMO (49%)<br>H–2»L+2 (31%)

[a] Oscillator strength. [b] Contributions smaller than 10% are not included. H = HOMO, L = LUMO. Orbitals are numbered consecutively regardless of possible degeneracies.

Table S35. Electronic transitions calculated for **1f**–H^+ using the TD/PCM(dichloromethane)GD3BJ-B3LYP/6-31G(d,p) level of theory.

No.	Energy (cm^{-1})	λ (nm)	f[a]	Major excitations[b]
1	6768	1477.6	0.095	HOMO»LUMO (100%)
2	13993	714.6	0.658	H–1»LUMO (99%)
3	14637	683.2	0.023	HOMO»L+1 (89%)
4	14986	667.3	0.004	H–2»LUMO (94%)
5	16361	611.2	0.016	HOMO»L+2 (95%)
6	17129	583.8	0.001	H–3»LUMO (99%)
7	17262	579.3	0.004	H–4»LUMO (99%)
8	18191	549.7	0.119	H–9»LUMO (26%)<br>H–7»LUMO (39%)<br>H–6»LUMO (24%)
9	18826	531.2	0.038	H–10»LUMO (24%)<br>H–9»LUMO (59%)<br>H–6»LUMO (13%)
10	18933	528.2	0.000	H–5»LUMO (97%)
11	19045	525.1	0.001	H–7»LUMO (40%)<br>H–6»LUMO (54%)
12	19244	519.7	0.008	H–8»LUMO (96%)
13	19525	512.2	0.194	H–10»LUMO (57%)<br>H–7»LUMO (15%)
14	21329	468.9	0.318	H–1»L+1 (83%)
15	21976	455.0	0.037	H–11»LUMO (80%)<br>H–1»L+2 (17%)
16	22831	438.0	0.162	H–12»LUMO (75%)
17	23073	433.4	0.001	H–2»L+1 (95%)
18	23302	429.1	0.095	H–13»LUMO (80%)<br>H–1»L+2 (14%)
19	23601	423.7	0.110	H–16»LUMO (10%)<br>H–14»LUMO (46%)<br>H–1»L+2 (26%)
20	23655	422.7	0.301	H–14»LUMO (25%)<br>H–13»LUMO (12%)<br>H–1»L+2 (36%)
21	24064	415.6	0.000	H–17»LUMO (84%)
22	24093	415.1	0.005	H–15»LUMO (87%)
23	24151	414.1	0.104	HOMO»L+3 (88%)
24	24204	413.2	0.001	H–18»LUMO (23%)<br>H–4»L+1 (56%)
25	24253	412.3	0.002	H–18»LUMO (15%)<br>H–3»L+1 (63%)<br>H–3»L+2 (13%)

[a] Oscillator strength. [b] Contributions smaller than 10% are not included. H = HOMO, L = LUMO. Orbitals are numbered consecutively regardless of possible degeneracies.

Table S36. Electronic transitions calculated for **2a**–$2H^{2+}$ using the TD/PCM(dichloromethane)GD3BJ-B3LYP/6-31G(d,p) level of theory.

No.	Energy (cm^{-1})	λ (nm)	f[a]	Major excitations[b]
1	10513	951.2	0.753	HOMO»LUMO (100%)
2	13749	727.4	0.086	H–1»LUMO (96%)
3	15493	645.4	0.258	H–2»LUMO (14%)<br>HOMO»L+1 (83%)
4	16138	619.7	0.187	H–2»LUMO (72%)<br>HOMO»L+1 (15%)
5	18795	532.1	0.620	H–3»LUMO (59%)<br>H–1»L+1 (27%)
6	18898	529.1	0.038	H–4»LUMO (51%)<br>H–3»LUMO (19%)<br>H–1»L+1 (19%)
7	19492	513.0	0.066	H–4»LUMO (33%)<br>H–1»L+1 (50%)
8	19706	507.5	0.000	HOMO»L+2 (87%)
9	21829	458.1	0.006	H–2»L+1 (77%)
10	22166	451.1	0.008	H–6»LUMO (69%)<br>H–5»LUMO (28%)
11	22425	445.9	0.008	H–6»LUMO (28%)<br>H–5»LUMO (69%)
12	23374	427.8	0.001	H–9»LUMO (86%)
13	23556	424.5	0.474	H–8»LUMO (18%)<br>H–7»LUMO (40%)<br>H–1»L+2 (33%)
14	23672	422.4	0.004	H–8»LUMO (57%)<br>H–7»LUMO (33%)
15	23930	417.9	0.055	H–8»LUMO (11%)<br>H–7»LUMO (13%)<br>H–4»L+1 (29%)<br>H–1»L+2 (25%)
16	24266	412.1	0.072	H–4»L+1 (51%)<br>H–3»L+1 (18%)<br>H–1»L+2 (21%)
17	25131	397.9	0.013	H–3»L+1 (54%)<br>H–1»L+2 (16%)
18	25323	394.9	0.013	H–10»LUMO (83%)
19	25711	388.9	0.000	H–11»LUMO (92%)
20	25993	384.7	0.011	H–15»LUMO (91%)
21	26644	375.3	0.038	H–13»LUMO (11%)<br>H–12»LUMO (25%)<br>H–2»L+2 (40%)
22	26708	374.4	0.037	H–12»LUMO (55%)<br>H–2»L+2 (32%)
23	27003	370.3	0.000	H–17»LUMO (81%)<br>H–17»L+1 (10%)
24	27116	368.8	0.090	H–13»LUMO (76%)
25	27273	366.7	0.001	H–16»LUMO (60%)<br>H–16»L+1 (12%)<br>H–14»LUMO (12%)

[a] Oscillator strength. [b] Contributions smaller than 10% are not included. H = HOMO, L = LUMO. Orbitals are numbered consecutively regardless of possible degeneracies.

No.	Energy (cm^{-1})	λ (nm)	f[a]	Major excitations[b]
24	27116	368.8	0.090	H–13»LUMO (76%)
25	27273	366.7	0.001	H–16»LUMO (60%)<br>H–16»L+1 (12%)<br>H–14»LUMO (12%)

[a] Oscillator strength. [b] Contributions smaller than 10% are not included. H = HOMO, L = LUMO. Orbitals are numbered consecutively regardless of possible degeneracies.

Table S37. Electronic transitions calculated for **2a**–H^+ using the TD/PCM(dichloromethane)GD3BJ-B3LYP/6-31G(d,p) level of theory.

No.	Energy (cm^{-1})	λ (nm)	f[a]	Major excitations[b]
1	9591	1042.7	0.527	HOMO»LUMO (100%)
2	13092	763.8	0.118	H–1»LUMO (98%)
3	15683	637.6	0.214	HOMO»L+1 (97%)
4	17805	561.6	0.261	H–4»LUMO (13%)<br>H–3»LUMO (30%)<br>H–2»LUMO (32%)<br>HOMO»L+2 (22%)
5	18602	537.6	0.219	H–2»LUMO (50%)<br>HOMO»L+2 (35%)
6	18681	535.3	0.216	H–8»LUMO (14%)<br>H–3»LUMO (40%)<br>H–2»LUMO (14%)<br>HOMO»L+2 (22%)
7	18798	532.0	0.001	H–4»LUMO (74%)<br>H–3»LUMO (20%)
8	19142	522.4	0.136	H–1»L+1 (79%)
9	19314	517.8	0.080	H–9»LUMO (15%)<br>H–8»LUMO (11%)<br>H–7»LUMO (62%)
10	19607	510.0	0.198	H–9»LUMO (14%)<br>H–8»LUMO (51%)<br>H–1»L+1 (14%)
11	20317	492.2	0.000	H–5»LUMO (97%)
12	20440	489.2	0.001	H–6»LUMO (96%)
13	20945	477.4	0.306	H–9»LUMO (61%)<br>H–7»LUMO (23%)
14	22388	446.7	0.217	H–1»L+2 (92%)
15	24338	410.9	0.056	H–10»LUMO (86%)
16	24414	409.6	0.033	H–11»LUMO (92%)
17	25021	399.7	0.021	H–14»LUMO (46%)<br>H–2»L+1 (29%)
18	25060	399.0	0.031	H–14»LUMO (26%)<br>H–2»L+1 (39%)
19	25106	398.3	0.003	H–15»LUMO (61%)
20	25217	396.6	0.020	H–13»LUMO (16%)<br>H–12»LUMO (38%)<br>H–3»L+1 (28%)
21	25280	395.6	0.020	H–12»LUMO (28%)<br>H–3»L+1 (40%)
22	25328	394.8	0.007	H–13»LUMO (33%)<br>H–12»LUMO (22%)<br>H–4»L+1 (18%)
23	25410	393.6	0.003	H–13»LUMO (19%)<br>H–4»L+1 (57%)
24	25630	390.2	0.016	H–17»LUMO (14%)<br>H–16»LUMO (24%)<br>H–8»L+1 (11%)<br>H–7»L+1 (21%)
25	25780	387.9	0.006	H–16»LUMO (55%)<br>H–8»L+1 (16%)

[a] Oscillator strength. [b] Contributions smaller than 10% are not included. H = HOMO, L = LUMO. Orbitals are numbered consecutively regardless of possible degeneracies.

Table S38. Electronic transitions calculated for **2a**–H+ (*Z-anti*) using the TD/PCM(dichloromethane) GD3BJ-B3LYP/6-31G(d,p) level of theory.

No.	Energy (cm^{-1})	λ (nm)	f[a]	Major excitations[b]
1	9519	1050.5	0.568	HOMO»LUMO (100%)
2	12998	769.4	0.000	H–1»LUMO (98%)
3	15858	630.6	0.149	HOMO»L+1 (96%)
4	17689	565.3	0.123	H–4»LUMO (41%)<br>H–2»LUMO (45%)
5	18456	541.8	0.048	H–4»LUMO (35%)<br>H–2»LUMO (54%)
6	18476	541.3	0.020	H–3»LUMO (96%)
7	18630	536.8	0.241	H–8»LUMO (50%)<br>H–4»LUMO (21%)<br>HOMO»L+2 (27%)
8	18745	533.5	0.001	H–9»LUMO (38%)<br>H–5»LUMO (56%)
9	19107	523.4	0.421	H–8»LUMO (36%)<br>HOMO»L+2 (57%)
10	19410	515.2	0.298	H–1»L+1 (93%)
11	20128	496.8	0.000	H–6»LUMO (97%)
12	20129	496.8	0.062	H–7»LUMO (94%)
13	20255	493.7	0.442	H–9»LUMO (54%)<br>H–5»LUMO (39%)
14	22619	442.1	0.000	H–1»L+2 (96%)
15	24390	410.0	0.055	H–10»LUMO (96%)
16	24831	402.7	0.034	H–14»LUMO (25%)<br>H–13»LUMO (24%)<br>H–11»LUMO (43%)
17	24841	402.6	0.002	H–15»LUMO (73%)<br>H–12»LUMO (14%)
18	24851	402.4	0.037	H–16»LUMO (17%)<br>H–14»LUMO (39%)<br>H–13»LUMO (23%)
19	24941	400.9	0.076	H–2»L+1 (76%)
20	25064	399.0	0.001	H–13»LUMO (38%)<br>H–11»LUMO (45%)
21	25064	399.0	0.004	H–15»LUMO (13%)<br>H–12»LUMO (78%)
22	25110	398.3	0.004	H–3»L+1 (89%)
23	25273	395.7	0.138	H–16»LUMO (28%)<br>H–4»L+1 (57%)
24	25302	395.2	0.000	H–18»LUMO (22%)<br>H–17»LUMO (30%)<br>H–9»L+1 (19%)<br>H–5»L+1 (20%)
25	25424	393.3	0.010	H–17»LUMO (48%)<br>H–9»L+1 (15%)<br>H–5»L+1 (27%)

[a] Oscillator strength. [b] Contributions smaller than 10% are not included. H = HOMO, L = LUMO. Orbitals are numbered consecutively regardless of possible degeneracies.

Table S39. Electronic transitions calculated for **2a**–H+ (*Z-syn*) using the TD/PCM(dichloromethane)GD3BJ-B3LYP/6-31G(d,p) level of theory.

No.	Energy (cm–1)	λ (nm)	f[a]	Major excitations[b]
1	10288	972.0	0.737	HOMO»LUMO (101%)
2	13711	729.3	0.214	H–1»LUMO (96%)
3	16444	608.1	0.339	HOMO»L+1 (96%)
4	18131	551.5	0.477	H–4»LUMO (19%)<br>H–2»LUMO (56%)<br>HOMO»L+2 (23%)
5	18870	529.9	0.072	H–4»LUMO (15%)<br>H–2»LUMO (42%)<br>HOMO»L+2 (39%)
6	18885	529.5	0.003	H–3»LUMO (98%)
7	19073	524.3	0.163	H–4»LUMO (64%)<br>HOMO»L+2 (31%)
8	19635	509.3	0.002	H–9»LUMO (13%)<br>H–7»LUMO (83%)
9	19865	503.4	0.090	H–8»LUMO (21%)<br>H–1»L+1 (71%)
10	20193	495.2	0.054	H–8»LUMO (70%)<br>H–1»L+1 (25%)
11	20569	486.2	0.000	H–5»LUMO (95%)
12	20570	486.2	0.000	H–6»LUMO (95%)
13	21511	464.9	0.126	H–9»LUMO (81%)<br>H–7»LUMO (13%)
14	22883	437.0	0.358	H–1»L+2 (93%)
15	23786	420.4	0.048	H–10»LUMO (92%)
16	25190	397.0	0.001	H–15»LUMO (45%)<br>H–13»LUMO (25%)<br>H–2»L+1 (14%)
17	25231	396.3	0.000	H–16»LUMO (54%)<br>H–14»LUMO (25%)
18	25320	394.9	0.000	H–17»LUMO (12%)<br>H–15»LUMO (10%)<br>H–2»L+1 (66%)
19	25394	393.8	0.075	H–11»LUMO (95%)
20	25448	393.0	0.003	H–3»L+1 (88%)
21	25609	390.5	0.005	H–17»LUMO (22%)<br>H–4»L+1 (66%)
22	25690	389.3	0.002	H–12»LUMO (86%)
23	25761	388.2	0.000	H–15»LUMO (29%)<br>H–13»LUMO (67%)
24	25814	387.4	0.000	H–16»LUMO (23%)<br>H–14»LUMO (64%)
25	26128	382.7	0.020	H–9»L+1 (13%)<br>H–8»L+2 (13%)<br>H–7»L+1 (67%)

[a] Oscillator strength. [b] Contributions smaller than 10% are not included. H = HOMO, L = LUMO. Orbitals are numbered consecutively regardless of possible degeneracies.

Table S40. Electronic transitions calculated for **2a**–3H^{3+} using the TD/PCM(dichloromethane)GD3BJ-B3LYP/6-31G(d,p) level of theory.

No.	Energy (cm^{-1})	λ (nm)	f[a]	Major excitations[b]
1	14401	694.4	0.471	H–1»LUMO (40%)<br>HOMO»LUMO (58%)
2	14741	678.4	0.192	H–1»LUMO (58%)<br>HOMO»LUMO (41%)
3	15580	641.8	0.012	H–2»LUMO (98%)
4	16616	601.8	0.000	H–3»LUMO (99%)
5	17435	573.5	0.000	H–4»LUMO (98%)
6	17914	558.2	0.131	H–6»LUMO (72%)<br>H–5»LUMO (24%)
7	18456	541.8	0.015	H–7»LUMO (35%)<br>H–6»LUMO (21%)<br>H–5»LUMO (42%)
8	19261	519.2	0.165	H–7»LUMO (59%)<br>H–5»LUMO (32%)
9	22150	451.5	0.020	H–8»LUMO (73%)
10	22372	447.0	0.130	H–1»L+1 (16%)<br>HOMO»L+1 (47%)<br>HOMO»L+2 (16%)
11	22478	444.9	0.035	H–10»LUMO (23%)<br>H–1»L+1 (27%)<br>HOMO»L+1 (13%)<br>HOMO»L+2 (24%)
12	22545	443.6	0.003	H–9»LUMO (86%)
13	22835	437.9	0.118	H–10»LUMO (31%)<br>H–2»L+1 (33%)<br>H–1»L+1 (19%)
14	22921	436.3	0.159	H–10»LUMO (12%)<br>H–2»L+1 (60%)<br>H–1»L+1 (13%)
15	23265	429.8	0.000	H–11»LUMO (85%)
16	23507	425.4	0.014	H–12»LUMO (78%)
17	23664	422.6	0.039	H–15»LUMO (36%)<br>H–13»LUMO (20%)<br>H–12»LUMO (13%)<br>H–1»L+2 (11%)
18	24021	416.3	0.013	HOMO»L+1 (34%)<br>HOMO»L+2 (53%)
19	24165	413.8	0.002	H–3»L+1 (47%)<br>H–3»L+2 (47%)
20	24253	412.3	0.107	H–14»LUMO (35%)<br>H–13»LUMO (39%)
21	24498	408.2	0.005	H–4»L+1 (87%)
22	24543	407.4	0.503	H–14»LUMO (15%)<br>H–10»LUMO (13%)<br>H–1»L+2 (58%)
23	25059	399.1	0.031	H–15»LUMO (42%)<br>H–14»LUMO (27%)<br>H–13»LUMO (17%)
24	25486	392.4	0.058	H–7»L+1 (45%)<br>H–5»L+1 (33%)
25	25585	390.9	0.008	H–2»L+2 (89%)

[a] Oscillator strength. [b] Contributions smaller than 10% are not included. H = HOMO, L = LUMO. Orbitals are numbered consecutively regardless of possible degeneracies.

Table S41. Electronic transitions calculated for **2b** using the TD/PCM(dichloromethane)GD3BJ-B3LYP/6-31G(d,p) level of theory.

No.	Energy (cm^{-1})	λ (nm)	f[a]	Major excitations[b]
1	10769	928.6	0.737	HOMO»LUMO (100%)
2	12920	774.0	0.103	H–1»LUMO (93%)
3	14655	682.4	0.028	H–2»LUMO (90%)
4	15525	644.1	0.258	HOMO»L+1 (88%)
5	16277	614.4	0.116	H–3»LUMO (75%)<br>HOMO»L+1 (10%)
6	18109	552.2	0.044	H–1»L+1 (92%)
7	18940	528.0	0.589	H–4»LUMO (83%)
8	19390	515.7	0.084	H–5»LUMO (72%)<br>H–2»L+1 (16%)
9	19815	504.7	0.027	HOMO»L+2 (95%)
10	20087	497.8	0.012	H–5»LUMO (15%)<br>H–2»L+1 (77%)
11	21681	461.2	0.012	H–3»L+1 (77%)
12	22690	440.7	0.011	H–7»LUMO (34%)<br>H–6»LUMO (62%)
13	22919	436.3	0.472	H–1»L+2 (91%)
14	22957	435.6	0.001	H–7»LUMO (60%)<br>H–6»LUMO (33%)
15	23776	420.6	0.167	H–8»LUMO (78%)
16	23847	419.3	0.016	H–10»LUMO (85%)
17	24152	414.0	0.001	H–9»LUMO (87%)
18	24186	413.5	0.021	H–5»L+1 (74%)
19	24370	410.3	0.051	H–4»L+1 (54%)<br>H–3»L+1 (10%)<br>H–2»L+2 (27%)
20	24985	400.2	0.024	H–11»LUMO (87%)
21	25405	393.6	0.004	H–8»LUMO (10%)<br>H–4»L+1 (26%)<br>H–2»L+2 (50%)
22	26082	383.4	0.000	H–12»LUMO (91%)
23	26486	377.6	0.048	H–3»L+2 (73%)
24	26871	372.1	0.039	H–13»LUMO (83%)
25	27328	365.9	0.027	H–18»LUMO (41%)<br>H–14»LUMO (29%)

[a] Oscillator strength. [b] Contributions smaller than 10% are not included. H = HOMO, L = LUMO. Orbitals are numbered consecutively regardless of possible degeneracies.

Table S42. Electronic transitions calculated for **2b**–H^+ using the TD/PCM(dichloromethane)GD3BJ-B3LYP/6-31G(d,p) level of theory.

No.	Energy (cm^{-1})	λ (nm)	f[a]	Major excitations[b]
1	10166	983.7	0.401	HOMO»LUMO (99%)
2	12474	801.7	0.252	H–1»LUMO (98%)
3	15755	634.7	0.179	HOMO»L+1 (95%)
4	16951	590.0	0.205	H–2»LUMO (86%)<br>H–1»L+1 (13%)
5	17671	565.9	0.224	H–2»LUMO (12%)<br>H–1»L+1 (83%)
6	18399	543.5	0.281	H–3»LUMO (43%)<br>HOMO»L+2 (52%)
7	18999	526.3	0.432	H–3»LUMO (46%)<br>HOMO»L+2 (39%)
8	19879	503.0	0.068	H–7»LUMO (75%)
9	20053	498.7	0.002	H–4»LUMO (85%)
10	20146	496.4	0.002	H–5»LUMO (90%)
11	20233	494.2	0.129	H–9»LUMO (69%)
12	20620	485.0	0.486	H–1»L+2 (92%)
13	21630	462.3	0.000	H–6»LUMO (92%)
14	21684	461.2	0.000	H–9»LUMO (11%)<br>H–8»LUMO (84%)
15	22320	448.0	0.047	H–2»L+1 (94%)
16	22960	435.5	0.105	H–10»LUMO (84%)
17	24803	403.2	0.066	H–7»L+1 (23%)<br>H–3»L+1 (49%)<br>H–2»L+2 (17%)
18	25275	395.6	0.021	H–11»LUMO (94%)
19	25464	392.7	0.017	H–12»LUMO (10%)<br>H–7»L+1 (46%)<br>H–3»L+1 (19%)
20	25560	391.2	0.037	H–12»LUMO (30%)<br>H–9»L+1 (16%)<br>H–2»L+2 (22%)
21	25671	389.5	0.017	H–12»LUMO (25%)<br>H–4»L+1 (58%)
22	25769	388.1	0.001	H–9»L+1 (42%)<br>H–5»L+1 (16%)
23	25793	387.7	0.016	H–12»LUMO (11%)<br>H–5»L+1 (56%)<br>H–4»L+1 (11%)
24	25963	385.2	0.073	H–5»L+1 (20%)<br>H–3»L+1 (13%)<br>H–2»L+2 (27%)
25	25994	384.7	0.003	H–15»LUMO (67%)

[a] Oscillator strength. [b] Contributions smaller than 10% are not included. H = HOMO, L = LUMO. Orbitals are numbered consecutively regardless of possible degeneracies.

Table S43. Electronic transitions calculated for **2b**–4H^{4+} using the TD/PCM(dichloromethane)GD3BJ-B3LYP/6-31G(d,p) level of theory.

No.	Energy (cm^{-1})	λ (nm)	f[a]	Major excitations[b]
1	12992	769.7	0.032	HOMO»LUMO (99%)
2	13729	728.4	0.309	H–2»LUMO (16%)<br>H–1»LUMO (83%)
3	14006	714.0	0.321	H–2»LUMO (83%)<br>H–1»LUMO (16%)
4	15086	662.9	0.000	H–3»LUMO (99%)
5	15840	631.3	0.000	H–4»LUMO (99%)
6	17190	581.7	0.147	H–6»LUMO (52%)<br>H–5»LUMO (44%)
7	17803	561.7	0.016	H–7»LUMO (41%)<br>H–6»LUMO (32%)<br>H–5»LUMO (26%)
8	18916	528.6	0.122	H–7»LUMO (55%)<br>H–6»LUMO (14%)<br>H–5»LUMO (28%)
9	21489	465.4	0.000	H–8»LUMO (93%)
10	21670	461.5	0.012	H–9»LUMO (87%)
11	21707	460.7	0.012	HOMO»L+1 (48%)<br>HOMO»L+2 (43%)
12	21818	458.3	0.028	H–10»LUMO (70%)
13	22004	454.5	0.001	H–11»LUMO (81%)
14	22132	451.8	0.021	H–1»L+1 (71%)
15	22280	448.8	0.017	H–12»LUMO (88%)
16	22862	437.4	0.413	H–2»L+1 (75%)
17	23363	428.0	0.009	HOMO»L+1 (43%)<br>HOMO»L+2 (52%)
18	23484	425.8	0.000	H–3»L+1 (45%)<br>H–3»L+2 (51%)
19	23809	420.0	0.000	H–4»L+1 (89%)
20	23929	417.9	0.033	H–13»LUMO (92%)
21	24161	413.9	0.343	H–10»LUMO (10%)<br>H–2»L+2 (54%)<br>H–1»L+2 (24%)
22	24556	407.2	0.048	H–14»LUMO (84%)
23	24689	405.0	0.018	H–2»L+2 (28%)<br>H–1»L+2 (62%)
24	25129	397.9	0.014	H–15»LUMO (94%)
25	25329	394.8	0.246	H–16»LUMO (92%)

[a] Oscillator strength. [b] Contributions smaller than 10% are not included. H = HOMO, L = LUMO. Orbitals are numbered consecutively regardless of possible degeneracies.

Table S44. Electronic transitions calculated for **2c** using the TD/PCM(dichloromethane)GD3BJ-B3LYP/6-31G(d,p) level of theory.

No.	Energy (cm^{-1})	λ (nm)	f[a]	Major excitations[b]
1	10548	948.0	0.743	HOMO»LUMO (100%)
2	11368	879.6	0.009	H–1»LUMO (100%)
3	13805	724.4	0.090	H–2»LUMO (96%)
4	15491	645.5	0.258	H–3»LUMO (13%)<br>HOMO»L+1 (83%)
5	16155	619.0	0.187	H–3»LUMO (73%)<br>HOMO»L+1 (14%)
6	17106	584.6	0.001	H–1»L+1 (100%)
7	18830	531.1	0.549	H–4»LUMO (50%)<br>H–2»L+1 (33%)
8	18950	527.7	0.085	H–5»LUMO (46%)<br>H–4»LUMO (29%)<br>H–2»L+1 (14%)
9	19518	512.4	0.064	H–5»LUMO (32%)<br>H–2»L+1 (46%)<br>HOMO»L+2 (15%)
10	19697	507.7	0.001	HOMO»L+2 (81%)
11	21805	458.6	0.006	H–3»L+1 (77%)
12	21926	456.1	0.016	H–1»L+2 (97%)
13	22293	448.6	0.010	H–7»LUMO (69%)<br>H–6»LUMO (27%)
14	22544	443.6	0.009	H–7»LUMO (28%)<br>H–6»LUMO (69%)
15	23463	426.2	0.335	H–8»LUMO (71%)<br>H–2»L+2 (19%)
16	23485	425.8	0.018	H–10»LUMO (88%)
17	23776	420.6	0.000	H–9»LUMO (90%)
18	23893	418.5	0.151	H–8»LUMO (13%)<br>H–5»L+1 (28%)<br>H–2»L+2 (40%)
19	24264	412.1	0.082	H–5»L+1 (53%)<br>H–4»L+1 (16%)<br>H–2»L+2 (21%)
20	24712	404.7	0.061	H–12»LUMO (69%)<br>H–4»L+1 (16%)
21	25052	399.2	0.022	H–13»LUMO (48%)<br>H–12»LUMO (10%)<br>H–11»LUMO (20%)
22	25221	396.5	0.004	H–12»LUMO (13%)<br>H–11»LUMO (15%)<br>H–4»L+1 (36%)<br>H–2»L+2 (10%)
23	25318	395.0	0.005	H–13»LUMO (27%)<br>H–11»LUMO (65%)
24	25798	387.6	0.000	H–14»LUMO (93%)
25	26609	375.8	0.042	H–3»L+2 (64%)

[a] Oscillator strength. [b] Contributions smaller than 10% are not included. H = HOMO, L = LUMO. Orbitals are numbered consecutively regardless of possible degeneracies.

Table S45. Electronic transitions calculated for **2c**–H$^+$ using the TD/PCM(dichloromethane)GD3BJ-B3LYP/6-31G(d,p) level of theory.

No.	Energy (cm^{-1})	λ (nm)	f[a]	Major excitations[b]
1	9759	1024.7	0.457	H–1»LUMO (99%)
2	10077	992.3	0.532	HOMO»LUMO (100%)
3	13591	735.8	0.052	H–2»LUMO (97%)
4	15711	636.5	0.182	H–1»L+1 (97%)
5	16113	620.6	0.052	HOMO»L+1 (98%)
6	17991	555.8	0.235	H–3»LUMO (58%)<br>H–1»L+2 (31%)
7	18572	538.5	0.154	HOMO»L+2 (93%)
8	18683	535.2	0.540	H–9»LUMO (15%)<br>H–3»LUMO (23%)<br>H–1»L+2 (55%)
9	19223	520.2	0.019	H–4»LUMO (74%)<br>H–3»LUMO (15%)
10	19340	517.1	0.035	H–5»LUMO (68%)
11	19446	514.2	0.158	H–9»LUMO (19%)<br>H–5»LUMO (24%)<br>H–2»L+1 (41%)
12	19552	511.4	0.052	H–10»LUMO (19%)<br>H–9»LUMO (10%)<br>H–7»LUMO (35%)<br>H–2»L+1 (24%)
13	19805	504.9	0.157	H–10»LUMO (26%)<br>H–9»LUMO (29%)<br>H–7»LUMO (13%)<br>H–2»L+1 (23%)
14	20871	479.1	0.003	H–6»LUMO (93%)
15	20971	476.8	0.066	H–8»LUMO (66%)<br>H–7»LUMO (18%)
16	21018	475.8	0.193	H–10»LUMO (37%)<br>H–8»LUMO (28%)<br>H–7»LUMO (21%)
17	22608	442.3	0.114	H–2»L+2 (90%)
18	23565	424.4	0.002	H–11»LUMO (98%)
19	24539	407.5	0.093	H–14»LUMO (19%)<br>H–12»LUMO (67%)
20	24714	404.6	0.020	H–13»LUMO (81%)
21	24896	401.7	0.039	H–14»LUMO (36%)<br>H–13»LUMO (16%)<br>H–3»L+1 (36%)
22	25251	396.0	0.038	H–14»LUMO (12%)<br>H–9»L+1 (37%)<br>H–7»L+1 (11%)
23	25427	393.3	0.001	H–17»LUMO (74%)
24	25467	392.7	0.008	H–4»L+1 (62%)<br>H–3»L+1 (13%)
25	25484	392.4	0.000	H–19»LUMO (58%)<br>H–15»LUMO (16%)

[a] Oscillator strength. [b] Contributions smaller than 10% are not included. H = HOMO, L = LUMO. Orbitals are numbered consecutively regardless of possible degeneracies.

Table S46. Electronic transitions calculated for **2c**–4H^{4+} using the TD/PCM(dichloromethane)GD3BJ-B3LYP/6-31G(d,p) level of theory.

No.	Energy (cm^{-1})	λ (nm)	f[a]	Major excitations[b]
1	14181	705.2	0.337	H–1»LUMO (17%)<br>HOMO»LUMO (74%)
2	14482	690.5	0.280	H–2»LUMO (16%)<br>H–1»LUMO (59%)<br>HOMO»LUMO (25%)
3	14873	672.4	0.057	H–2»LUMO (75%)<br>H–1»LUMO (24%)
4	16338	612.1	0.000	H–3»LUMO (99%)
5	16759	596.7	0.000	H–4»LUMO (98%)
6	17733	563.9	0.210	H–6»LUMO (47%)<br>H–5»LUMO (48%)
7	17997	555.7	0.124	H–6»LUMO (50%)<br>H–5»LUMO (42%)
8	18644	536.4	0.215	H–7»LUMO (87%)
9	21563	463.8	0.211	H–8»LUMO (82%)
10	22228	449.9	0.027	H–11»LUMO (12%)<br>H–10»LUMO (35%)<br>H–1»L+1 (27%)
11	22353	447.4	0.015	H–1»L+1 (11%)<br>HOMO»L+1 (37%)<br>HOMO»L+2 (37%)
12	22389	446.6	0.001	H–9»LUMO (81%)
13	22417	446.1	0.004	H–10»LUMO (35%)<br>H–2»L+1 (19%)<br>H–1»L+1 (17%)<br>HOMO»L+1 (11%)
14	22504	444.4	0.010	H–11»LUMO (39%)<br>H–2»L+1 (29%)
15	22811	438.4	0.003	H–12»LUMO (85%)
16	23066	433.5	0.190	H–13»LUMO (48%)<br>H–11»LUMO (11%)<br>H–2»L+1 (15%)
17	23198	431.1	0.209	H–13»LUMO (44%)<br>H–2»L+1 (21%)<br>H–1»L+1 (12%)
18	24011	416.5	0.064	HOMO»L+1 (35%)<br>HOMO»L+2 (54%)
19	24087	415.2	0.000	H–3»L+1 (46%)<br>H–3»L+2 (48%)
20	24092	415.1	0.001	H–4»L+1 (86%)
21	24288	411.7	0.323	H–11»LUMO (12%)<br>H–2»L+2 (24%)<br>H–1»L+2 (40%)
22	24565	407.1	0.033	H–17»LUMO (12%)<br>H–16»LUMO (10%)<br>H–14»LUMO (52%)
23	24964	400.6	0.017	H–2»L+2 (57%)<br>H–1»L+2 (35%)
24	25066	398.9	0.026	H–17»LUMO (21%)<br>H–16»LUMO (19%)<br>H–15»LUMO (12%)<br>H–14»LUMO (32%)
25	25365	394.3	0.061	H–15»LUMO (45%)<br>H–7»L+1 (19%)

[a] Oscillator strength. [b] Contributions smaller than 10% are not included. H = HOMO, L = LUMO. Orbitals are numbered consecutively regardless of possible degeneracies.

Table S47. Electronic transitions calculated for **2d** using the TD/PCM(dichloromethane)GD3BJ-B3LYP/6-31G(d,p) level of theory.

No.	Energy (cm^{-1})	λ (nm)	f[a]	Major excitations[b]
1	10055	994.6	0.726	HOMO»LUMO (100%)
2	13361	748.5	0.112	H–1»LUMO (96%)
3	15357	651.2	0.232	H–2»LUMO (18%)<br>HOMO»L+1 (78%)
4	15942	627.3	0.236	H–3»LUMO (11%)<br>H–2»LUMO (65%)<br>HOMO»L+1 (19%)
5	18440	542.3	0.582	H–3»LUMO (75%)<br>H–2»LUMO (13%)
6	18682	535.3	0.116	H–4»LUMO (68%)<br>H–1»L+1 (22%)
7	19249	519.5	0.004	H–1»L+1 (18%)<br>HOMO»L+2 (80%)
8	19379	516.0	0.020	H–4»LUMO (20%)<br>H–1»L+1 (56%)<br>HOMO»L+2 (14%)
9	21261	470.3	0.004	H–6»LUMO (89%)
10	21518	464.7	0.004	H–5»LUMO (89%)
11	21955	455.5	0.009	H–3»L+1 (12%)<br>H–2»L+1 (72%)
12	22588	442.7	0.000	H–8»LUMO (93%)
13	22880	437.1	0.000	H–7»LUMO (93%)
14	23272	429.7	0.506	H–9»LUMO (53%)<br>H–1»L+2 (42%)
15	23722	421.5	0.052	H–9»LUMO (34%)<br>H–4»L+1 (16%)<br>H–1»L+2 (29%)
16	24212	413.0	0.050	H–4»L+1 (66%)<br>H–1»L+2 (15%)
17	25061	399.0	0.005	H–10»LUMO (51%)<br>H–3»L+1 (28%)
18	25139	397.8	0.015	H–12»LUMO (16%)<br>H–11»LUMO (65%)
19	25167	397.3	0.002	H–10»LUMO (35%)<br>H–3»L+1 (30%)
20	26222	381.4	0.025	H–12»LUMO (72%)<br>H–11»LUMO (18%)
21	26436	378.3	0.000	H–17»LUMO (81%)
22	26534	376.9	0.011	H–14»LUMO (41%)<br>H–2»L+2 (37%)
23	26618	375.7	0.007	H–13»LUMO (84%)
24	26739	374.0	0.000	H–15»LUMO (72%)<br>H–15»L+1 (12%)
25	26799	373.2	0.099	H–14»LUMO (47%)<br>H–2»L+2 (27%)

[a] Oscillator strength. [b] Contributions smaller than 10% are not included. H = HOMO, L = LUMO. Orbitals are numbered consecutively regardless of possible degeneracies.

Table S48. Electronic transitions calculated for **2d**–H$^+$ using the TD/PCM(dichloromethane)GD3BJ-B3LYP/6-31G(d,p) level of theory.

No.	Energy (cm^{-1})	λ (nm)	f[a]	Major excitations[b]
1	9120	1096.5	0.616	HOMO»LUMO (101%)
2	13008	768.8	0.130	H–1»LUMO (98%)
3	15617	640.3	0.210	HOMO»L+1 (96%)
4	17163	582.6	0.218	H–4»LUMO (40%)<br>H–3»LUMO (13%)<br>H–2»LUMO (30%)<br>HOMO»L+2 (14%)
5	17547	569.9	0.015	H–4»LUMO (14%)<br>H–3»LUMO (15%)<br>H–2»LUMO (67%)
6	17743	563.6	0.014	H–4»LUMO (25%)<br>H–3»LUMO (70%)
7	18237	548.3	0.342	H–8»LUMO (31%)<br>H–7»LUMO (10%)<br>HOMO»L+2 (48%)
8	18674	535.5	0.071	H–7»LUMO (81%)
9	18953	527.6	0.364	H–8»LUMO (59%)<br>HOMO»L+2 (28%)
10	19265	519.1	0.000	H–5»LUMO (97%)
11	19424	514.8	0.000	H–6»LUMO (97%)
12	19726	506.9	0.146	H–1»L+1 (95%)
13	20915	478.1	0.230	H–9»LUMO (85%)
14	22646	441.6	0.152	H–1»L+2 (90%)
15	23272	429.7	0.032	H–10»LUMO (89%)
16	23774	420.6	0.031	H–11»LUMO (94%)
17	24258	412.2	0.001	H–14»LUMO (31%)<br>H–13»LUMO (56%)
18	24330	411.0	0.000	H–16»LUMO (11%)<br>H–15»LUMO (62%)<br>H–12»LUMO (11%)
19	24504	408.1	0.001	H–12»LUMO (87%)
20	24647	405.7	0.015	H–16»LUMO (13%)<br>H–15»LUMO (15%)<br>H–14»LUMO (36%)<br>H–13»LUMO (20%)
21	24816	403.0	0.015	H–2»L+1 (80%)
22	24858	402.3	0.009	H–17»LUMO (81%)
23	24949	400.8	0.008	H–16»LUMO (29%)<br>H–3»L+1 (42%)
24	25055	399.1	0.025	H–16»LUMO (21%)<br>H–3»L+1 (46%)
25	25349	394.5	0.080	H–7»L+1 (11%)<br>H–4»L+1 (66%)

[a] Oscillator strength. [b] Contributions smaller than 10% are not included. H = HOMO, L = LUMO. Orbitals are numbered consecutively regardless of possible degeneracies.

Table S49. Electronic transitions calculated for **2d**–$3H^{3+}$ using the TD/PCM(dichloromethane)GD3BJ-B3LYP/6-31G(d,p) level of theory.

No.	Energy (cm^{-1})	λ (nm)	f[a]	Major excitations[b]
1	12985	770.1	0.049	HOMO»LUMO (99%)
2	13641	733.1	0.559	H–1»LUMO (97%)
3	14013	713.6	0.116	H–2»LUMO (98%)
4	15064	663.8	0.000	H–3»LUMO (99%)
5	15897	629.0	0.000	H–4»LUMO (99%)
6	16945	590.1	0.130	H–6»LUMO (49%)<br>H–5»LUMO (47%)
7	17533	570.4	0.015	H–7»LUMO (37%)<br>H–6»LUMO (35%)<br>H–5»LUMO (26%)
8	18806	531.7	0.121	H–7»LUMO (58%)<br>H–6»LUMO (13%)<br>H–5»LUMO (25%)
9	20663	484.0	0.020	H–12»LUMO (26%)<br>H–8»LUMO (61%)
10	21292	469.7	0.000	H–9»LUMO (84%)<br>H–8»LUMO (10%)
11	21647	462.0	0.032	H–10»LUMO (76%)
12	21867	457.3	0.014	H–13»LUMO (10%)<br>H–12»LUMO (48%)<br>H–8»LUMO (19%)
13	21950	455.6	0.015	HOMO»L+1 (55%)<br>HOMO»L+2 (42%)
14	22022	454.1	0.000	H–11»LUMO (86%)
15	22224	450.0	0.018	H–13»LUMO (79%)
16	22359	447.2	0.046	H–2»L+1 (45%)<br>H–1»L+1 (42%)
17	22705	440.4	0.359	H–2»L+1 (45%)<br>H–1»L+1 (35%)
18	23563	424.4	0.005	HOMO»L+1 (42%)<br>HOMO»L+2 (51%)
19	23709	421.8	0.003	H–3»L+1 (44%)<br>H–3»L+2 (51%)
20	23736	421.3	0.082	H–15»LUMO (14%)<br>H–14»LUMO (67%)
21	23902	418.4	0.081	H–15»LUMO (70%)<br>H–14»LUMO (17%)
22	24019	416.3	0.005	H–4»L+1 (89%)
23	24141	414.2	0.513	H–2»L+2 (12%)<br>H–1»L+2 (65%)
24	24554	407.3	0.046	H–16»LUMO (87%)
25	24949	400.8	0.001	H–17»LUMO (95%)

[a] Oscillator strength. [b] Contributions smaller than 10% are not included. H = HOMO, L = LUMO. Orbitals are numbered consecutively regardless of possible degeneracies.

Table S50. Electronic transitions calculated for **2e** using the TD/PCM(dichloromethane)GD3BJ-B3LYP/6-31G(d,p) level of theory.

No.	Energy (cm^{-1})	λ (nm)	f[a]	Major excitations[b]
1	10109	989.2	0.693	HOMO»LUMO (100%)
2	10811	925.0	0.003	HOMO»L+1 (96%)
3	13256	754.4	0.088	H–1»LUMO (72%)<br>HOMO»L+2 (25%)
4	13303	751.7	0.037	H–1»LUMO (25%)<br>HOMO»L+2 (71%)
5	15311	653.1	0.003	H–1»L+1 (94%)
6	15416	648.7	0.237	H–2»LUMO (14%)<br>HOMO»L+3 (79%)
7	16027	623.9	0.204	H–3»LUMO (12%)<br>H–2»LUMO (67%)<br>HOMO»L+3 (15%)
8	17735	563.9	0.003	H–1»L+2 (96%)
9	18467	541.5	0.616	H–3»LUMO (74%)<br>H–2»LUMO (15%)
10	18712	534.4	0.064	H–4»LUMO (32%)<br>H–2»L+1 (34%)<br>H–1»L+3 (24%)
11	18719	534.2	0.010	H–4»LUMO (23%)<br>H–2»L+1 (52%)<br>H–1»L+3 (14%)
12	19264	519.1	0.043	H–4»LUMO (32%)<br>H–1»L+3 (55%)
13	19511	512.5	0.003	HOMO»L+4 (90%)
14	20434	489.4	0.000	H–3»L+1 (86%)
15	21212	471.4	0.000	H–2»L+2 (87%)
16	21294	469.6	0.004	H–6»LUMO (85%)<br>H–5»LUMO (12%)
17	21508	464.9	0.000	H–4»L+1 (94%)
18	21586	463.3	0.004	H–6»LUMO (12%)<br>H–5»LUMO (85%)
19	22014	454.3	0.011	H–3»L+3 (14%)<br>H–2»L+3 (71%)
20	22621	442.1	0.000	H–8»LUMO (93%)
21	22874	437.2	0.007	H–3»L+2 (87%)
22	22953	435.7	0.001	H–7»LUMO (93%)
23	23265	429.8	0.459	H–9»LUMO (57%)<br>H–1»L+4 (37%)
24	23658	422.7	0.028	H–9»LUMO (23%)<br>H–4»L+2 (26%)<br>H–4»L+3 (20%)<br>H–1»L+4 (14%)
25	23906	418.3	0.095	H–4»L+2 (42%)<br>H–1»L+4 (31%)

[a] Oscillator strength. [b] Contributions smaller than 10% are not included. H = HOMO, L = LUMO. Orbitals are numbered consecutively regardless of possible degeneracies.

Table S51. Electronic transitions calculated for **2e**–H^+ using the TD/PCM(dichloromethane)GD3BJ-B3LYP/6-31G(d,p) level of theory.

No.	Energy (cm^{-1})	λ (nm)	f[a]	Major excitations[b]
1	8474	1180.1	0.555	HOMO»LUMO (101%)
2	12383	807.6	0.112	H–1»LUMO (96%)
3	13640	733.2	0.061	HOMO»L+1 (93%)
4	15683	637.6	0.189	HOMO»L+2 (84%)<br>HOMO»L+3 (12%)
5	15917	628.3	0.014	HOMO»L+2 (12%)<br>HOMO»L+3 (82%)
6	16526	605.1	0.146	H–4»LUMO (19%)<br>H–2»LUMO (71%)
7	16775	596.1	0.093	H–4»LUMO (29%)<br>H–3»LUMO (42%)<br>H–2»LUMO (25%)
8	16965	589.4	0.052	H–4»LUMO (43%)<br>H–3»LUMO (51%)
9	18066	553.5	0.067	H–8»LUMO (41%)<br>H–7»LUMO (42%)<br>H–1»L+1 (11%)
10	18185	549.9	0.056	H–8»LUMO (37%)<br>H–1»L+1 (46%)
11	18283	547.0	0.041	H–8»LUMO (12%)<br>H–7»LUMO (40%)<br>H–1»L+1 (38%)
12	18465	541.6	0.000	H–5»LUMO (98%)
13	18646	536.3	0.000	H–6»LUMO (97%)
14	19249	519.5	0.595	HOMO»L+4 (88%)
15	19810	504.8	0.127	H–1»L+2 (96%)
16	20186	495.4	0.254	H–9»LUMO (79%)
17	20546	486.7	0.050	H–1»L+3 (88%)
18	23097	433.0	0.023	H–10»LUMO (95%)
19	23251	430.1	0.033	H–4»L+1 (49%)<br>H–2»L+1 (29%)
20	23474	426.0	0.053	H–12»LUMO (17%)<br>H–2»L+1 (28%)<br>H–1»L+4 (39%)
21	23534	424.9	0.010	H–12»LUMO (15%)<br>H–11»LUMO (69%)
22	23562	424.4	0.012	H–12»LUMO (34%)<br>H–11»LUMO (25%)<br>H–4»L+1 (14%)<br>H–2»L+1 (16%)
23	23665	422.6	0.008	H–13»LUMO (82%)
24	23707	421.8	0.102	H–12»LUMO (28%)<br>H–4»L+1 (14%)<br>H–1»L+4 (37%)
25	23772	420.7	0.001	H–15»LUMO (28%)<br>H–14»LUMO (58%)

[a] Oscillator strength. [b] Contributions smaller than 10% are not included. H = HOMO, L = LUMO. Orbitals are numbered consecutively regardless of possible degeneracies.

Table S52. Electronic transitions calculated for **2e**–3H^{3+} using the TD/PCM(dichloromethane)GD3BJ-B3LYP/6-31G(d,p) level of theory.

No.	Energy (cm^{-1})	λ (nm)	f[a]	Major excitations[b]
1	12149	823.1	0.026	HOMO»LUMO (100%)
2	12964	771.4	0.303	H–1»LUMO (91%)
3	13251	754.7	0.315	H–2»LUMO (91%)
4	14241	702.2	0.000	H–3»LUMO (99%)
5	15089	662.7	0.000	H–4»LUMO (99%)
6	16524	605.2	0.119	H–6»LUMO (38%)<br>H–5»LUMO (58%)
7	17033	587.1	0.020	H–7»LUMO (33%)<br>H–6»LUMO (46%)<br>H–5»LUMO (20%)
8	18319	545.9	0.111	H–7»LUMO (62%)<br>H–6»LUMO (14%)<br>H–5»LUMO (21%)
9	20631	484.7	0.000	H–8»LUMO (95%)
10	20774	481.4	0.013	H–9»LUMO (76%)<br>H–2»L+1 (11%)
11	20870	479.2	0.085	H–9»LUMO (15%)<br>H–2»L+1 (43%)<br>H–1»L+1 (18%)<br>HOMO»L+1 (11%)
12	21252	470.5	0.059	H–12»LUMO (10%)<br>H–10»LUMO (71%)
13	21281	469.9	0.007	HOMO»L+1 (38%)<br>HOMO»L+2 (30%)<br>HOMO»L+3 (11%)
14	21369	468.0	0.016	H–11»LUMO (91%)
15	21458	466.0	0.010	H–12»LUMO (81%)<br>H–10»LUMO (11%)
16	21906	456.5	0.010	H–2»L+1 (16%)<br>H–1»L+1 (37%)<br>H–1»L+2 (36%)
17	22112	452.2	0.010	HOMO»L+1 (45%)<br>HOMO»L+2 (38%)<br>HOMO»L+3 (12%)
18	22806	438.5	0.334	H–2»L+2 (58%)<br>H–1»L+2 (21%)
19	23041	434.0	0.058	H–13»LUMO (89%)
20	23084	433.2	0.000	H–3»L+1 (36%)<br>H–3»L+2 (42%)<br>H–3»L+3 (17%)
21	23357	428.1	0.008	H–2»L+2 (16%)<br>H–1»L+1 (32%)<br>H–1»L+2 (35%)
22	23437	426.7	0.088	H–2»L+3 (20%)<br>H–1»L+3 (11%)<br>HOMO»L+2 (10%)<br>HOMO»L+3 (37%)
23	23593	423.9	0.001	H–4»L+1 (45%)<br>H–4»L+2 (45%)
24	23634	423.1	0.061	H–2»L+3 (29%)<br>H–1»L+3 (13%)<br>HOMO»L+2 (13%)<br>HOMO»L+3 (31%)
25	23756	420.9	0.014	H–14»LUMO (86%)

[a] Oscillator strength. [b] Contributions smaller than 10% are not included. H = HOMO, L = LUMO. Orbitals are numbered consecutively regardless of possible degeneracies.

Table S53. Electronic transitions calculated for **2a**$^{+\bullet}$ using the TD/PCM(dichloromethane)GD3BJ-B3LYP/6-31G(d,p) level of theory.

No.	Energy (cm^{-1})	λ (nm)	f[a]	Major excitations[b]
1	6121	1633.7	0.079	HOMO(B)»LUMO(B) (93%)
2	8876	1126.6	0.016	H–2(B)»LUMO(B) (18%)<br>H–1(B)»LUMO(B) (70%)
3	10448	957.1	0.439	HOMO(A)»LUMO(A) (83%)
4	10888	918.5	0.322	H–2(B)»LUMO(B) (68%)<br>H–1(B)»LUMO(B) (19%)
5	12029	831.3	0.012	H–7(B)»LUMO(B) (28%)<br>H–6(B)»LUMO(B) (36%)<br>HOMO(B)»L+1(B) (16%)
6	12937	773.0	0.022	H–1(A)»LUMO(A) (10%)<br>H–7(B)»LUMO(B) (10%)<br>H–6(B)»LUMO(B) (13%)<br>HOMO(B)»L+1(B) (59%)
7	13765	726.5	0.017	H–4(B)»LUMO(B) (18%)<br>H–3(B)»LUMO(B) (72%)
8	13817	723.7	0.000	H–4(B)»LUMO(B) (77%)<br>H–3(B)»LUMO(B) (20%)
9	13892	719.8	0.003	H–2(A)»LUMO(A) (43%)<br>H–1(B)»L+1(B) (16%)
10	15011	666.2	0.144	HOMO(A)»L+1(A) (20%)<br>H–8(B)»LUMO(B) (36%)
11	15151	660.0	0.171	H–1(A)»LUMO(A) (11%)<br>HOMO(A)»L+1(A) (19%)<br>H–8(B)»LUMO(B) (28%)
12	15635	639.6	0.000	H–5(B)»LUMO(B) (98%)
13	15658	638.6	0.000	H–7(B)»LUMO(B) (55%)<br>H–6(B)»LUMO(B) (43%)
14	16191	617.6	0.295	H–1(A)»LUMO(A) (44%)<br>HOMO(A)»L+1(A) (20%)<br>H–1(B)»L+1(B) (12%)<br>HOMO(B)»L+1(B) (11%)
15	17288	578.4	0.104	H–2(A)»LUMO(A) (34%)<br>H–2(B)»L+1(B) (23%)<br>H–1(B)»L+1(B) (18%)
16	17307	577.8	0.025	H–5(A)»LUMO(A) (11%)<br>H–8(B)»LUMO(B) (18%)<br>H–2(B)»L+1(B) (19%)<br>H–1(B)»L+1(B) (18%)
17	17315	577.5	0.001	HOMO(A)»L+1(A) (13%)<br>H–9(B)»LUMO(B) (28%)
18	17845	560.4	0.028	H–8(A)»LUMO(A) (24%)<br>H–9(B)»LUMO(B) (22%)
19	18297	546.5	0.000	H–10(B)»LUMO(B) (34%)<br>H–9(B)»LUMO(B) (24%)
20	18746	533.4	0.006	HOMO(A)»L+2(A) (30%)<br>H–16(B)»LUMO(B) (45%)
21	18819	531.4	0.009	HOMO(A)»L+2(A) (29%)<br>H–16(B)»LUMO(B) (49%)
22	19041	525.2	0.015	H–11(B)»LUMO(B) (42%)<br>H–10(B)»LUMO(B) (27%)
23	19290	518.4	0.014	H–11(B)»LUMO(B) (24%)<br>H–10(B)»LUMO(B) (17%)<br>HOMO(B)»L+2(B) (24%)
24	19344	517.0	0.007	H–12(B)»LUMO(B) (57%)<br>H–11(B)»LUMO(B) (16%)<br>HOMO(B)»L+2(B) (16%)
25	19502	512.8	0.055	H–12(B)»LUMO(B) (28%)<br>HOMO(B)»L+2(B) (40%)

[a] Oscillator strength. [b] Contributions smaller than 10% are not included. H = HOMO, L = LUMO. Orbitals are numbered consecutively regardless of possible degeneracies.

Table S54. Electronic transitions calculated for **2a**$^{2+}$ (closed shell) using the TD/PCM(dichloromethane)GD3BJ-B3LYP/6-31G(d,p) level of theory.

No.	Energy (cm^{-1})	λ (nm)	f[a]	Major excitations[b]
1	9358	1068.6	0.054	H–5»LUMO (81%) HOMO»LUMO (18%)
2	9577	1044.2	0.005	H–1»LUMO (99%)
3	9715	1029.3	0.004	H–2»LUMO (99%)
4	10547	948.1	0.474	H–5»LUMO (17%) HOMO»LUMO (85%)
5	11668	857.0	0.000	H–3»LUMO (99%)
6	11847	844.1	0.000	H–4»LUMO (99%)
7	13038	767.0	0.009	H–8»LUMO (15%) H–7»LUMO (82%)
8	13400	746.3	0.444	H–6»LUMO (89%)
9	14783	676.5	0.307	H–8»LUMO (83%) H–7»LUMO (15%)
10	15812	632.4	0.000	H–9»LUMO (89%)
11	16707	598.6	0.308	HOMO»L+1 (65%)
12	16913	591.3	0.027	H–12»LUMO (14%) H–11»LUMO (10%) H–10»LUMO (71%)
13	17601	568.2	0.034	H–11»LUMO (68%) H–10»LUMO (13%)
14	17826	561.0	0.004	H–1»L+1 (97%)
15	17889	559.0	0.004	H–2»L+1 (97%)
16	18114	552.1	0.000	H–12»LUMO (82%) H–10»LUMO (12%)
17	18252	547.9	0.024	H–5»L+1 (79%)
18	18474	541.3	0.000	H–13»LUMO (92%)
19	18678	535.4	0.116	H–15»LUMO (21%) H–14»LUMO (56%)
20	19783	505.5	0.000	H–3»L+1 (95%)
21	19840	504.0	0.196	H–15»LUMO (51%) H–14»LUMO (13%)
22	19886	502.9	0.004	H–4»L+1 (93%)
23	20047	498.8	0.006	H–16»LUMO (83%)
24	20512	487.5	0.118	H–17»LUMO (66%) H–15»LUMO (10%)
25	20676	483.7	0.264	H–17»LUMO (12%) H–6»L+1 (80%)

[a] Oscillator strength. [b] Contributions smaller than 10% are not included. H = HOMO, L = LUMO. Orbitals are numbered consecutively regardless of possible degeneracies.

Table S55. Electronic transitions calculated for **2a**$^{3+\bullet}$ using the TD/PCM(dichloromethane)GD3BJ-B3LYP/6-31G(d,p) level of theory.

No.	Energy (cm^{-1})	λ (nm)	f[a]	Major excitations[b]
1	3500	2856.8	0.000	HOMO(B)»LUMO(B) (100%)
2	3588	2786.8	0.000	H–1(B)»LUMO(B) (100%)
3	5172	1933.3	0.004	HOMO(A)»LUMO(A) (100%)
4	5264	1899.8	0.002	H–4(B)»LUMO(B) (91%)
5	5342	1872.0	0.004	H–1(A)»LUMO(A) (99%)
6	5793	1726.3	0.000	H–2(B)»LUMO(B) (99%)
7	5935	1685.0	0.000	H–3(B)»LUMO(B) (100%)
8	5988	1670.0	0.001	H–4(A)»LUMO(A) (78%)
9	6306	1585.9	0.017	H–5(A)»LUMO(A) (13%)<br>H–5(B)»LUMO(B) (71%)
10	7364	1358.0	0.000	H–2(A)»LUMO(A) (99%)
11	7587	1318.0	0.000	H–3(A)»LUMO(A) (99%)
12	7690	1300.3	0.002	HOMO(B)»L+1(B) (99%)
13	8004	1249.3	0.002	H–1(B)»L+1(B) (99%)
14	8606	1162.0	0.044	H–7(B)»LUMO(B) (30%)<br>H–6(B)»LUMO(B) (37%)
15	8870	1127.3	0.076	H–5(A)»LUMO(A) (33%)<br>H–7(B)»LUMO(B) (19%)
16	9218	1084.8	0.004	H–5(B)»L+1(B) (10%)<br>H–4(B)»L+1(B) (57%)
17	9426	1060.9	0.187	H–5(A)»LUMO(A) (30%)<br>H–7(B)»LUMO(B) (31%)<br>H–4(B)»L+1(B) (17%)
18	9508	1051.7	0.008	H–6(A)»LUMO(A) (19%)<br>H–6(B)»LUMO(B) (46%)<br>H–5(B)»L+1(B) (13%)
19	9948	1005.2	0.000	H–2(B)»L+1(B) (99%)
20	10196	980.8	0.122	H–7(A)»LUMO(A) (55%)<br>H–6(A)»LUMO(A) (13%)
21	10332	967.9	0.000	H–3(B)»L+1(B) (99%)
22	11151	896.7	0.010	H–8(B)»LUMO(B) (63%)
23	11777	849.1	0.214	H–6(A)»LUMO(A) (32%)<br>H–8(B)»LUMO(B) (10%)<br>H–5(B)»L+1(B) (28%)
24	12076	828.1	0.072	H–8(A)»LUMO(A) (50%)<br>H–8(B)»LUMO(B) (17%)
25	12836	779.1	0.037	H–9(A)»LUMO(A) (26%)<br>H–8(A)»LUMO(A) (11%)<br>H–10(B)»LUMO(B) (15%)

[a] Oscillator strength. [b] Contributions smaller than 10% are not included. H = HOMO, L = LUMO. Orbitals are numbered consecutively regardless of possible degeneracies.

Table S56. Electronic transitions calculated for protonated dipyrrin radical **2a**–$H^{•++}$ using the TD/PCM(dichloromethane)GD3BJ-B3LYP/6-31G(d,p) level of theory.

No.	Energy (cm^{-1})	λ (nm)	f[a]	Major excitations[b]
1	5546	1803.1	0.329	HOMO(B)»LUMO(B) (91%)
2	10307	970.2	0.015	H–1(B)»LUMO(B) (90%)
3	10384	963.0	0.069	H–2(A)»LUMO(A) (12%)<br>HOMO(B)»L+1(B) (75%)
4	10491	953.2	0.007	H–2(B)»LUMO(B) (90%)
5	10707	934.0	0.234	HOMO(A)»LUMO(A) (59%)<br>H–4(B)»LUMO(B) (21%)
6	10853	921.4	0.318	HOMO(A)»LUMO(A) (20%)<br>H–4(B)»LUMO(B) (61%)
7	12416	805.4	0.000	H–3(B)»LUMO(B) (99%)
8	12546	797.1	0.000	H–5(B)»LUMO(B) (99%)
9	12831	779.3	0.029	H–8(B)»LUMO(B) (13%)<br>H–7(B)»LUMO(B) (57%)<br>H–6(B)»LUMO(B) (19%)
10	12952	772.1	0.019	H–8(B)»LUMO(B) (28%)<br>H–7(B)»LUMO(B) (33%)<br>H–6(B)»LUMO(B) (29%)
11	13373	747.8	0.079	H–8(B)»LUMO(B) (50%)<br>H–6(B)»LUMO(B) (37%)
12	14904	670.9	0.034	H–2(A)»LUMO(A) (43%)<br>HOMO(B)»L+1(B) (10%)
13	16033	623.7	0.022	H–6(A)»LUMO(A) (12%)<br>H–4(B)»L+1(B) (43%)
14	16284	614.1	0.164	H–7(A)»LUMO(A) (12%)<br>H–2(A)»LUMO(A) (25%)<br>HOMO(A)»L+1(A) (13%)
15	16530	604.9	0.001	H–1(A)»LUMO(A) (92%)
16	16734	597.6	0.002	H–1(B)»L+1(B) (89%)
17	16933	590.6	0.002	H–3(A)»LUMO(A) (92%)
18	17022	587.5	0.006	H–2(B)»L+1(B) (75%)
19	17078	585.5	0.038	HOMO(A)»L+1(A) (12%)<br>H–9(B)»LUMO(B) (24%)<br>H–2(B)»L+1(B) (19%)
20	17293	578.3	0.040	H–9(A)»LUMO(A) (25%)<br>HOMO(A)»L+1(A) (12%)<br>H–12(B)»LUMO(B) (11%)<br>H–8(B)»L+1(B) (13%)
21	17339	576.8	0.016	H–9(B)»LUMO(B) (53%)
22	17789	562.1	0.003	H–12(B)»LUMO(B) (29%)<br>H–11(B)»LUMO(B) (34%)
23	18068	553.5	0.001	H–14(B)»LUMO(B) (20%)<br>H–12(B)»LUMO(B) (25%)<br>H–11(B)»LUMO(B) (29%)
24	18281	547.0	0.112	H–15(B)»LUMO(B) (20%)<br>H–14(B)»LUMO(B) (17%)
25	18348	545.0	0.016	H–4(A)»LUMO(A) (42%)<br>H–10(B)»LUMO(B) (25%)<br>H–3(B)»L+1(B) (16%)

[a] Oscillator strength. [b] Contributions smaller than 10% are not included. H = HOMO, L = LUMO. Orbitals are numbered consecutively regardless of possible degeneracies.

Table S57. Electronic transitions calculated for **2d**$^{+•}$ using the TD/PCM(dichloromethane)GD3BJ-B3LYP/6-31G(d,p) level of theory.

No.	Energy (cm^{-1})	λ (nm)	f[a]	Major excitations[b]
1	6076	1645.9	0.052	HOMO(B)»LUMO(B) (91%)
2	9118	1096.7	0.026	H–2(B)»LUMO(B) (14%)<br>H–1(B)»LUMO(B) (63%)
3	9987	1001.3	0.450	HOMO(A)»LUMO(A) (79%)
4	10980	910.8	0.295	H–4(B)»LUMO(B) (18%)<br>H–2(B)»LUMO(B) (38%)<br>H–1(B)»LUMO(B) (21%)
5	11805	847.1	0.016	H–1(A)»LUMO(A) (13%)<br>H–7(B)»LUMO(B) (36%)<br>HOMO(B)»L+1(B) (38%)
6	12636	791.4	0.009	H–7(B)»LUMO(B) (46%)<br>HOMO(B)»L+1(B) (38%)
7	13365	748.2	0.009	H–4(B)»LUMO(B) (24%)<br>H–3(B)»LUMO(B) (71%)
8	13405	746.0	0.001	H–4(B)»LUMO(B) (50%)<br>H–3(B)»LUMO(B) (10%)<br>H–2(B)»LUMO(B) (39%)
9	13590	735.9	0.003	H–2(A)»LUMO(A) (45%)<br>H–1(B)»L+1(B) (15%)
10	14927	669.9	0.039	H–1(A)»LUMO(A) (17%)<br>HOMO(A)»L+1(A) (28%)<br>H–8(B)»LUMO(B) (15%)
11	15133	660.8	0.232	H–8(B)»LUMO(B) (41%)
12	15270	654.9	0.000	H–6(B)»LUMO(B) (98%)
13	15300	653.6	0.000	H–5(B)»LUMO(B) (99%)
14	15979	625.8	0.352	H–1(A)»LUMO(A) (33%)<br>HOMO(A)»L+1(A) (26%)<br>H–1(B)»L+1(B) (11%)
15	16978	589.0	0.179	H–2(A)»LUMO(A) (33%)<br>H–1(B)»L+1(B) (35%)
16	17107	584.6	0.003	H–8(B)»LUMO(B) (31%)<br>H–2(B)»L+1(B) (21%)
17	17297	578.1	0.003	H–9(A)»LUMO(A) (10%)<br>H–8(A)»LUMO(A) (23%)<br>HOMO(A)»L+1(A) (10%)<br>H–9(B)»LUMO(B) (10%)
18	17782	562.4	0.023	H–8(A)»LUMO(A) (18%)<br>HOMO(A)»L+1(A) (19%)<br>H–9(B)»LUMO(B) (14%)<br>H–7(B)»L+1(B) (12%)
19	18377	544.2	0.004	H–10(B)»LUMO(B) (18%)<br>H–9(B)»LUMO(B) (61%)
20	18514	540.1	0.011	HOMO(A)»L+2(A) (60%)
21	18735	533.7	0.003	H–15(B)»LUMO(B) (84%)
22	18896	529.2	0.028	H–3(A)»LUMO(A) (87%)
23	19081	524.1	0.013	H–4(A)»LUMO(A) (88%)
24	19184	521.3	0.034	H–11(B)»LUMO(B) (51%)<br>H–10(B)»LUMO(B) (17%)
25	19301	518.1	0.023	H–10(B)»LUMO(B) (14%)<br>HOMO(B)»L+2(B) (48%)

[a] Oscillator strength. [b] Contributions smaller than 10% are not included. H = HOMO, L = LUMO. Orbitals are numbered consecutively regardless of possible degeneracies.

Table S58. Electronic transitions calculated for **2d**$^{2+}$ (closed shell) using the TD/PCM(dichloromethane)GD3BJ-B3LYP/6-31G(d,p) level of theory.

No.	Energy (cm^{-1})	λ (nm)	f[a]	Major excitations[b]
1	9196	1087.4	0.005	H–1»LUMO (100%)
2	9311	1074.0	0.007	H–2»LUMO (99%)
3	9600	1041.7	0.051	H–5»LUMO (80%)<br>HOMO»LUMO (18%)
4	10759	929.4	0.467	H–5»LUMO (18%)<br>HOMO»LUMO (84%)
5	11338	882.0	0.000	H–3»LUMO (99%)
6	11493	870.1	0.000	H–4»LUMO (99%)
7	13281	753.0	0.010	H–8»LUMO (22%)<br>H–7»LUMO (73%)
8	13455	743.2	0.361	H–6»LUMO (84%)<br>HOMO»L+1 (12%)
9	14948	669.0	0.302	H–8»LUMO (75%)<br>H–7»LUMO (23%)
10	15864	630.4	0.007	H–9»LUMO (90%)
11	16463	607.4	0.442	H–14»LUMO (10%)<br>HOMO»L+1 (65%)
12	16746	597.2	0.002	H–1»L+1 (98%)
13	16853	593.4	0.003	H–2»L+1 (98%)
14	17546	569.9	0.011	H–11»LUMO (44%)<br>H–5»L+1 (47%)
15	17889	559.0	0.057	H–11»LUMO (36%)<br>H–10»LUMO (14%)<br>H–5»L+1 (41%)
16	17946	557.2	0.007	H–11»LUMO (13%)<br>H–10»LUMO (79%)
17	18277	547.2	0.002	H–12»LUMO (92%)
18	18566	538.6	0.025	H–14»LUMO (13%)<br>H–13»LUMO (78%)
19	18785	532.3	0.000	H–3»L+1 (94%)
20	18948	527.8	0.002	H–4»L+1 (91%)
21	19144	522.3	0.102	H–16»LUMO (64%)<br>H–14»LUMO (16%)
22	19819	504.6	0.183	H–16»LUMO (14%)<br>H–15»LUMO (16%)<br>H–14»LUMO (31%)<br>H–6»L+1 (15%)
23	19887	502.8	0.016	H–16»LUMO (18%)<br>H–15»LUMO (65%)
24	20238	494.1	0.315	H–14»LUMO (13%)<br>H–6»L+1 (77%)
25	20456	488.9	0.048	H–17»LUMO (81%)

[a] Oscillator strength. [b] Contributions smaller than 10% are not included. H = HOMO, L = LUMO. Orbitals are numbered consecutively regardless of possible degeneracies.

Table S59. Electronic transitions calculated for **2d**$^{3+\bullet}$ using the TD/PCM(dichloromethane)GD3BJ-B3LYP/6-31G(d,p) level of theory

No.	Energy (cm^{-1})	λ (nm)	f[a]	Major excitations[b]
1	2822	3543.4	0.001	HOMO(B)»LUMO(B) (100%)
2	2970	3367.3	0.000	H–1(B)»LUMO(B) (100%)
3	4580	2183.2	0.004	HOMO(A)»LUMO(A) (100%)
4	4680	2136.9	0.004	H–1(A)»LUMO(A) (99%)
5	5118	1954.0	0.003	H–4(B)»LUMO(B) (72%)<br>H–2(B)»LUMO(B) (24%)
6	5148	1942.4	0.001	H–4(B)»LUMO(B) (23%)<br>H–2(B)»LUMO(B) (76%)
7	5364	1864.1	0.000	H–3(B)»LUMO(B) (100%)
8	6115	1635.2	0.005	H–4(A)»LUMO(A) (59%)<br>H–5(B)»LUMO(B) (28%)
9	6305	1586.1	0.028	H–5(A)»LUMO(A) (11%)<br>H–4(A)»LUMO(A) (27%)<br>H–5(B)»LUMO(B) (53%)
10	6812	1468.0	0.000	H–2(A)»LUMO(A) (99%)
11	6964	1436.0	0.000	H–3(A)»LUMO(A) (99%)
12	7203	1388.4	0.001	HOMO(B)»L+1(B) (100%)
13	7486	1335.9	0.002	H–1(B)»L+1(B) (99%)
14	8643	1157.0	0.032	H–7(B)»LUMO(B) (28%)<br>H–6(B)»LUMO(B) (43%)
15	8959	1116.2	0.075	H–7(A)»LUMO(A) (10%)<br>H–5(A)»LUMO(A) (36%)<br>H–4(B)»L+1(B) (16%)
16	9473	1055.6	0.018	H–4(B)»L+1(B) (51%)<br>H–2(B)»L+1(B) (23%)
17	9504	1052.1	0.006	H–4(B)»L+1(B) (16%)<br>H–2(B)»L+1(B) (76%)
18	9586	1043.2	0.090	H–5(A)»LUMO(A) (18%)<br>H–7(B)»LUMO(B) (52%)
19	9635	1037.9	0.004	H–6(A)»LUMO(A) (31%)<br>H–7(B)»LUMO(B) (11%)<br>H–6(B)»LUMO(B) (31%)<br>H–5(B)»L+1(B) (19%)
20	9856	1014.6	0.000	H–3(B)»L+1(B) (99%)
21	10499	952.5	0.194	H–7(A)»LUMO(A) (45%)<br>H–6(A)»LUMO(A) (15%)<br>H–5(A)»LUMO(A) (14%)
22	11043	905.6	0.002	H–8(B)»LUMO(B) (78%)
23	11683	855.9	0.109	H–9(A)»LUMO(A) (17%)<br>H–6(A)»LUMO(A) (22%)<br>H–8(B)»LUMO(B) (13%)<br>H–5(B)»L+1(B) (18%)
24	12156	822.7	0.155	H–8(A)»LUMO(A) (50%)<br>H–7(A)»LUMO(A) (17%)
25	12689	788.1	0.066	H–9(A)»LUMO(A) (10%)<br>H–10(B)»LUMO(B) (54%)

[a] Oscillator strength. [b] Contributions smaller than 10% are not included. H = HOMO, L = LUMO. Orbitals are numbered consecutively regardless of possible degeneracies.

Table S60. Electronic transitions calculated for protonated dipyrrin radical **2d**–H$^{\bullet++}$ using the TD/PCM(dichloromethane)GD3BJ-B3LYP/6-31G(d,p) level of theory.

No.	Energy (cm^{-1})	λ (nm)	f[a]	Major excitations[b]
1	5960	1677.7	0.258	HOMO(A)»LUMO(A) (15%)<br>HOMO(B)»LUMO(B) (84%)
2	10063	993.7	0.531	HOMO(A)»LUMO(A) (54%)<br>HOMO(B)»LUMO(B) (10%)<br>HOMO(B)»L+1(B) (21%)
3	10168	983.5	0.044	H–1(B)»LUMO(B) (62%)<br>HOMO(B)»L+1(B) (14%)
4	10285	972.3	0.084	HOMO(A)»LUMO(A) (15%)<br>H–1(B)»LUMO(B) (36%)<br>HOMO(B)»L+1(B) (32%)
5	10416	960.1	0.007	H–2(B)»LUMO(B) (96%)
6	11013	908.0	0.094	H–5(B)»LUMO(B) (81%)
7	12331	811.0	0.000	H–3(B)»LUMO(B) (99%)
8	12437	804.0	0.000	H–4(B)»LUMO(B) (99%)
9	13015	768.3	0.023	H–8(B)»LUMO(B) (10%)<br>H–6(B)»LUMO(B) (74%)
10	13189	758.2	0.005	H–8(B)»LUMO(B) (20%)<br>H–7(B)»LUMO(B) (60%)
11	13986	715.0	0.135	H–8(B)»LUMO(B) (48%)<br>H–7(B)»LUMO(B) (19%)<br>H–5(B)»L+1(B) (10%)
12	14875	672.3	0.041	H–3(A)»LUMO(A) (41%)<br>HOMO(B)»L+1(B) (11%)
13	15291	654.0	0.012	H–1(A)»LUMO(A) (94%)
14	15620	640.2	0.001	H–1(B)»L+1(B) (75%)
15	15769	634.2	0.013	H–2(A)»LUMO(A) (94%)
16	15888	629.4	0.003	H–5(B)»L+1(B) (31%)<br>H–2(B)»L+1(B) (14%)<br>H–1(B)»L+1(B) (21%)
17	16066	622.4	0.027	H–2(B)»L+1(B) (68%)
18	16209	616.9	0.110	H–7(A)»LUMO(A) (14%)<br>H–3(A)»LUMO(A) (12%)<br>H–2(B)»L+1(B) (15%)
19	16846	593.6	0.073	H–8(A)»LUMO(A) (11%)<br>H–7(A)»LUMO(A) (20%)<br>HOMO(A)»L+1(A) (15%)
20	17184	581.9	0.069	H–9(A)»LUMO(A) (29%)<br>HOMO(A)»L+1(A) (11%)<br>H–8(B)»L+1(B) (10%)
21	17256	579.5	0.000	H–4(A)»LUMO(A) (96%)
22	17313	577.6	0.001	H–9(B)»LUMO(B) (91%)
23	17650	566.6	0.000	H–3(B)»L+1(B) (93%)
24	17662	566.2	0.000	H–5(A)»LUMO(A) (94%)
25	17900	558.7	0.001	H–12(B)»LUMO(B) (53%)<br>H–11(B)»LUMO(B) (10%)

[a] Oscillator strength. [b] Contributions smaller than 10% are not included. H = HOMO, L = LUMO. Orbitals are numbered consecutively regardless of possible degeneracies.

NMR Spectra

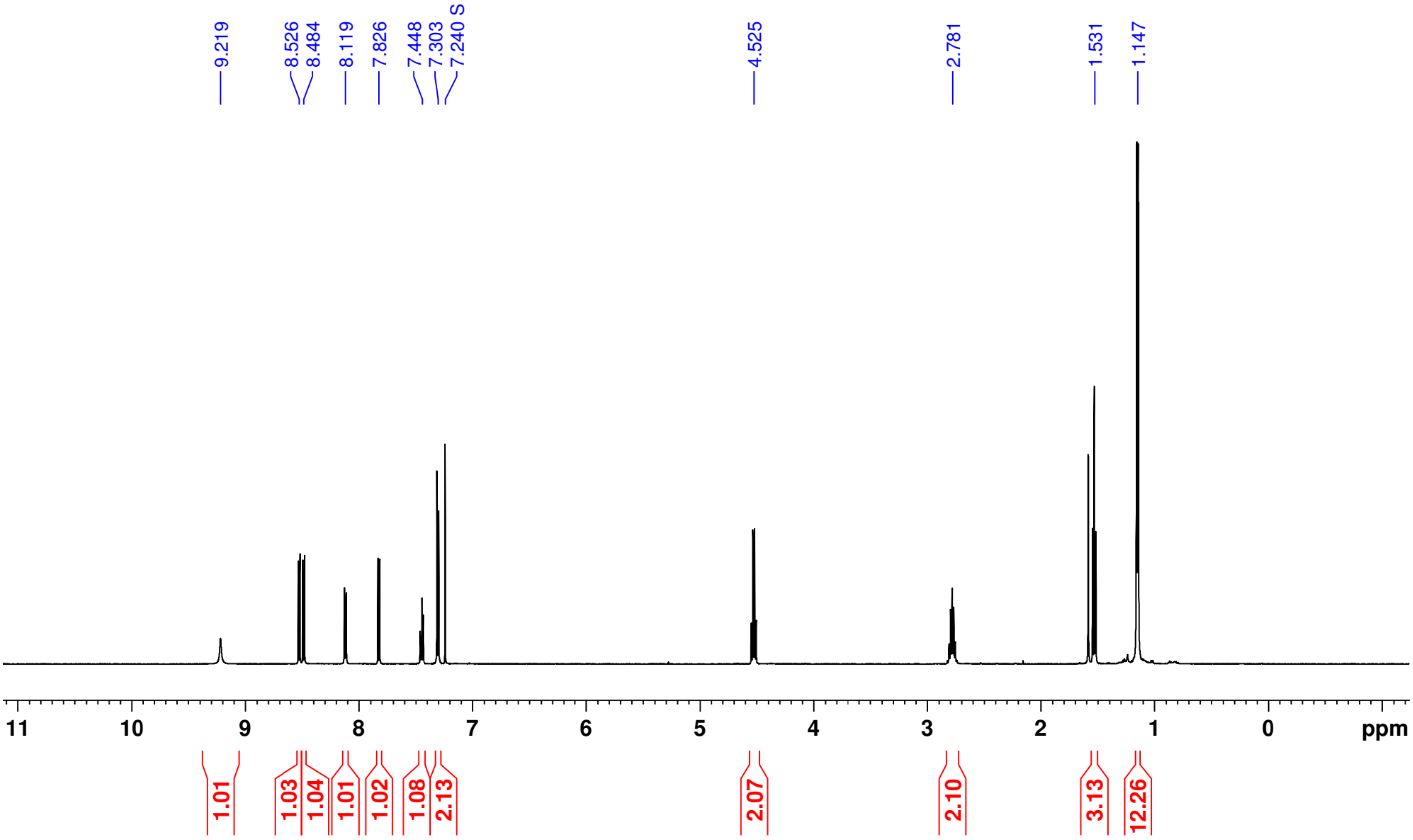

Figure S103. ^{1}H NMR spectrum of **3** (500 MHz, chloroform-*d*, 300 K).

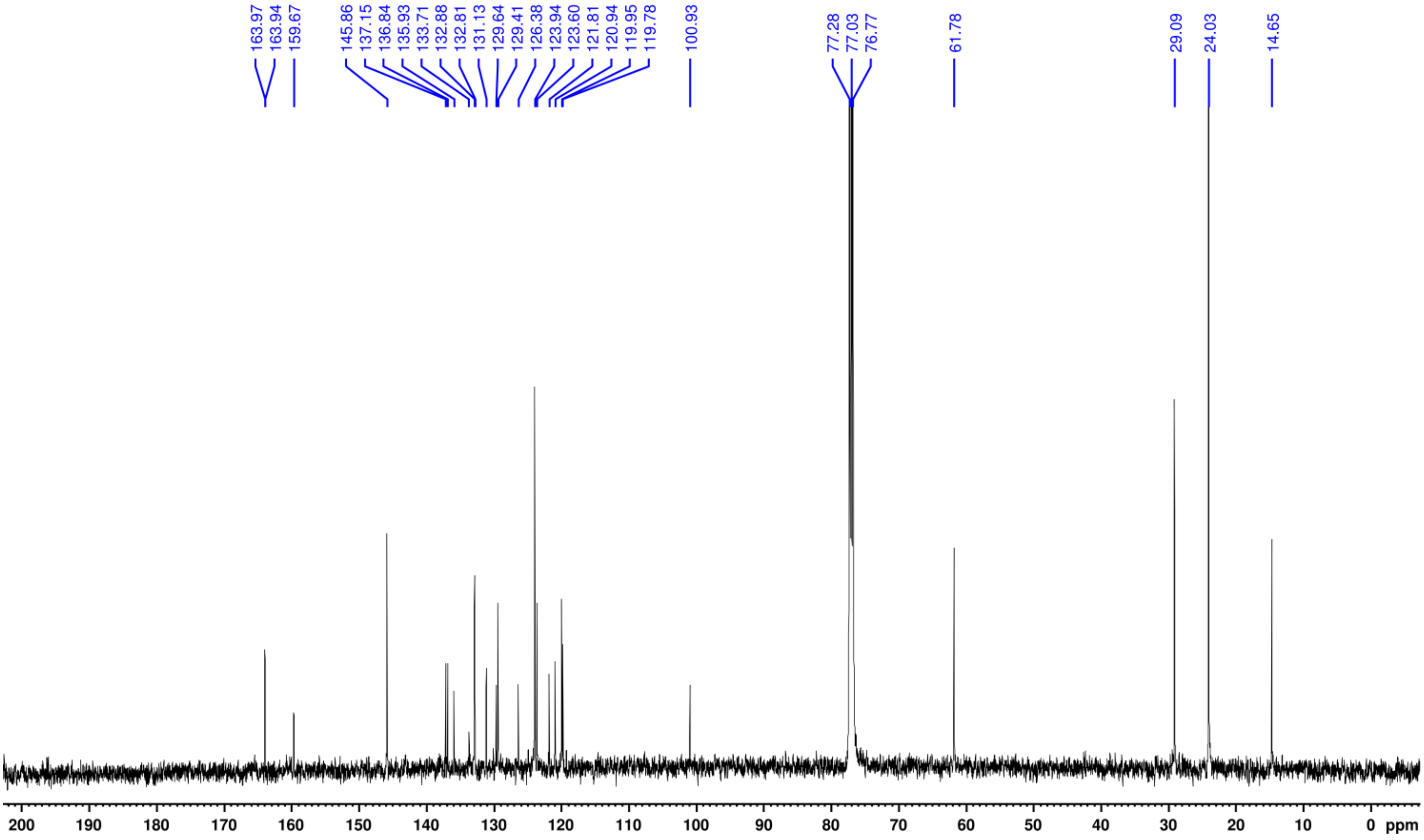

Figure S104. ^{13}C NMR spectrum of **3** (125 MHz, chloroform-*d*, 300 K).

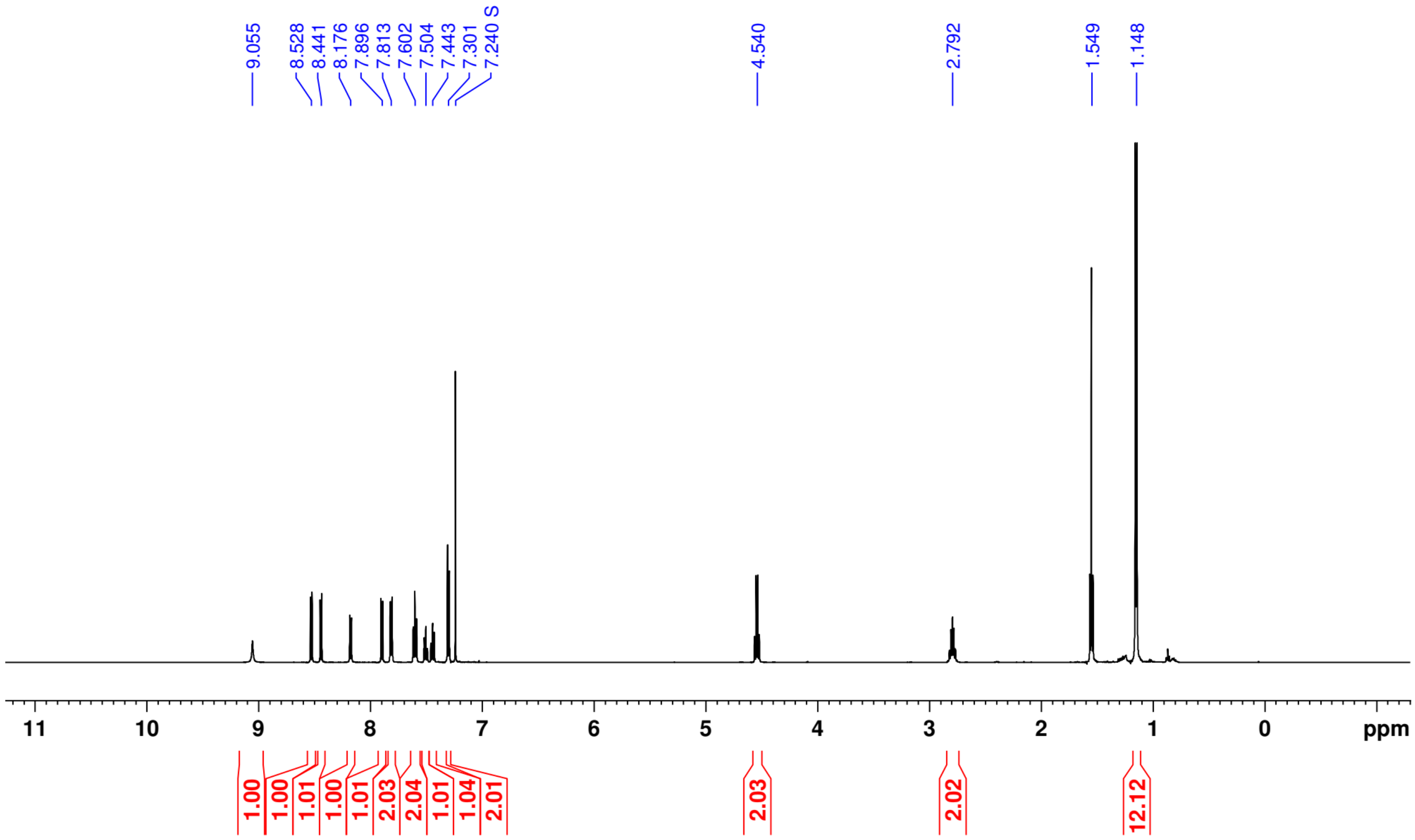

Figure S105. ^{1}H NMR spectrum of **4** (500 MHz, chloroform-*d*, 300 K).

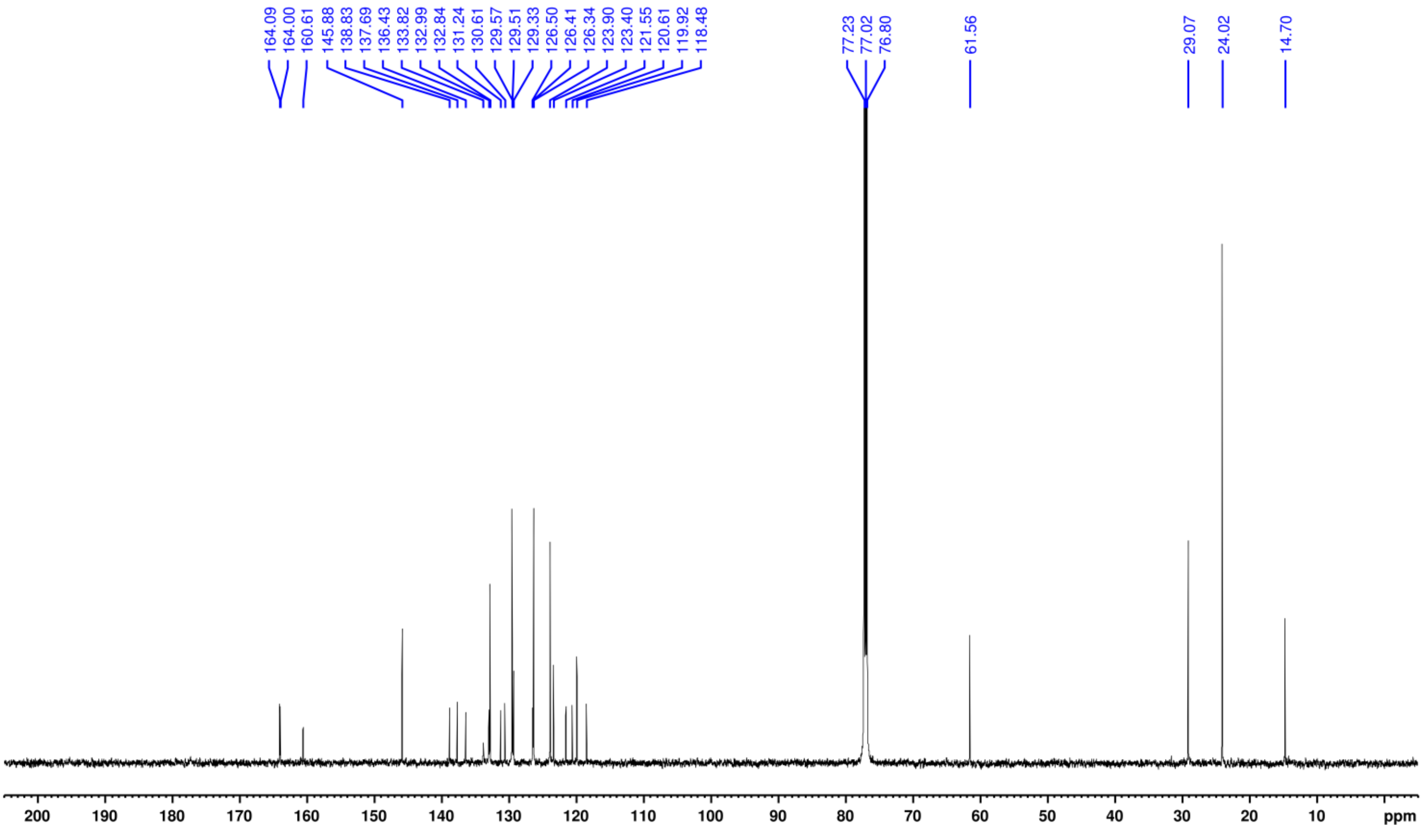

Figure S106. ^{13}C NMR spectrum of **4** (151 MHz, chloroform-*d*, 300 K).

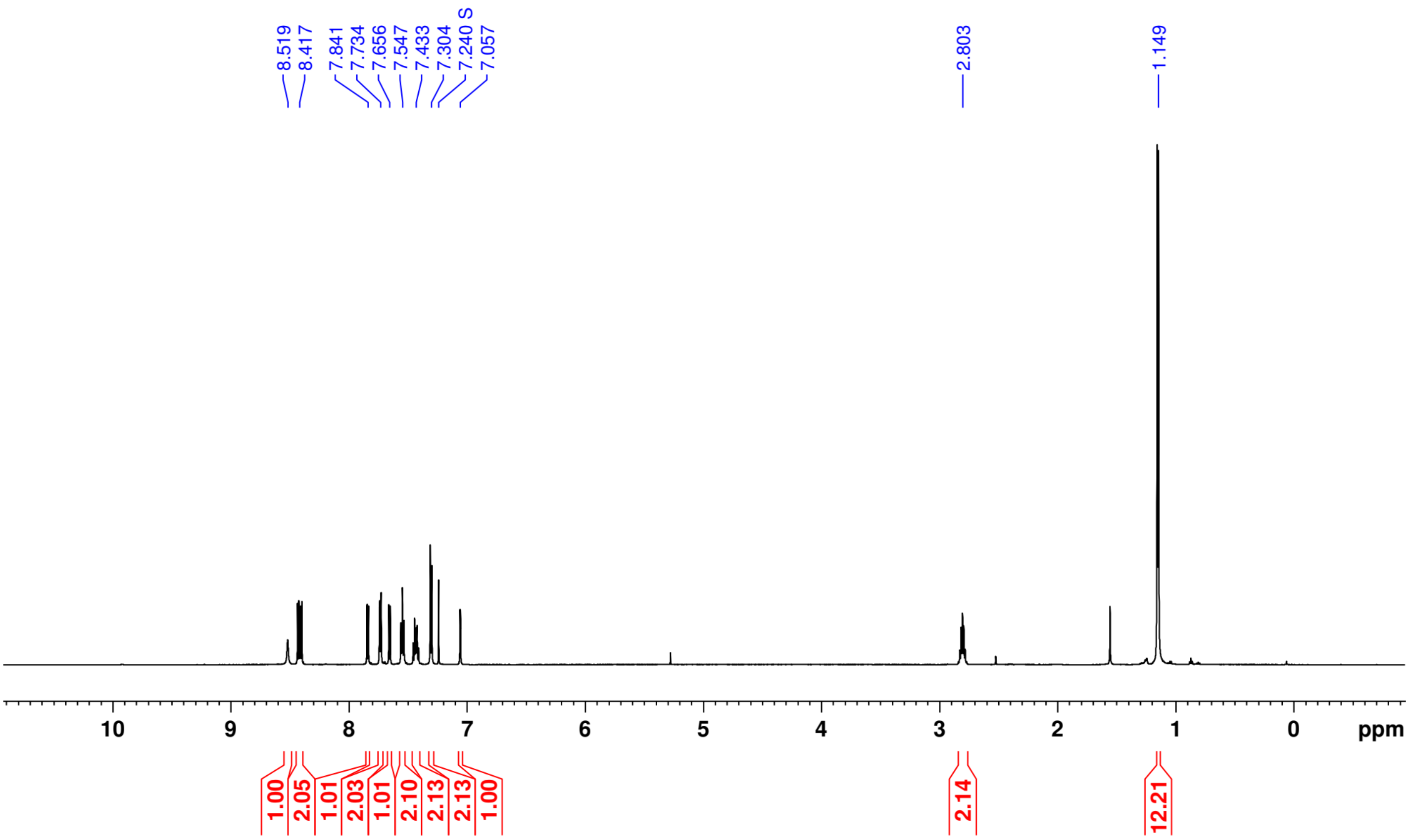

Figure S107. ^{1}H NMR spectrum of **6** (600 MHz, chloroform-*d*, 300 K).

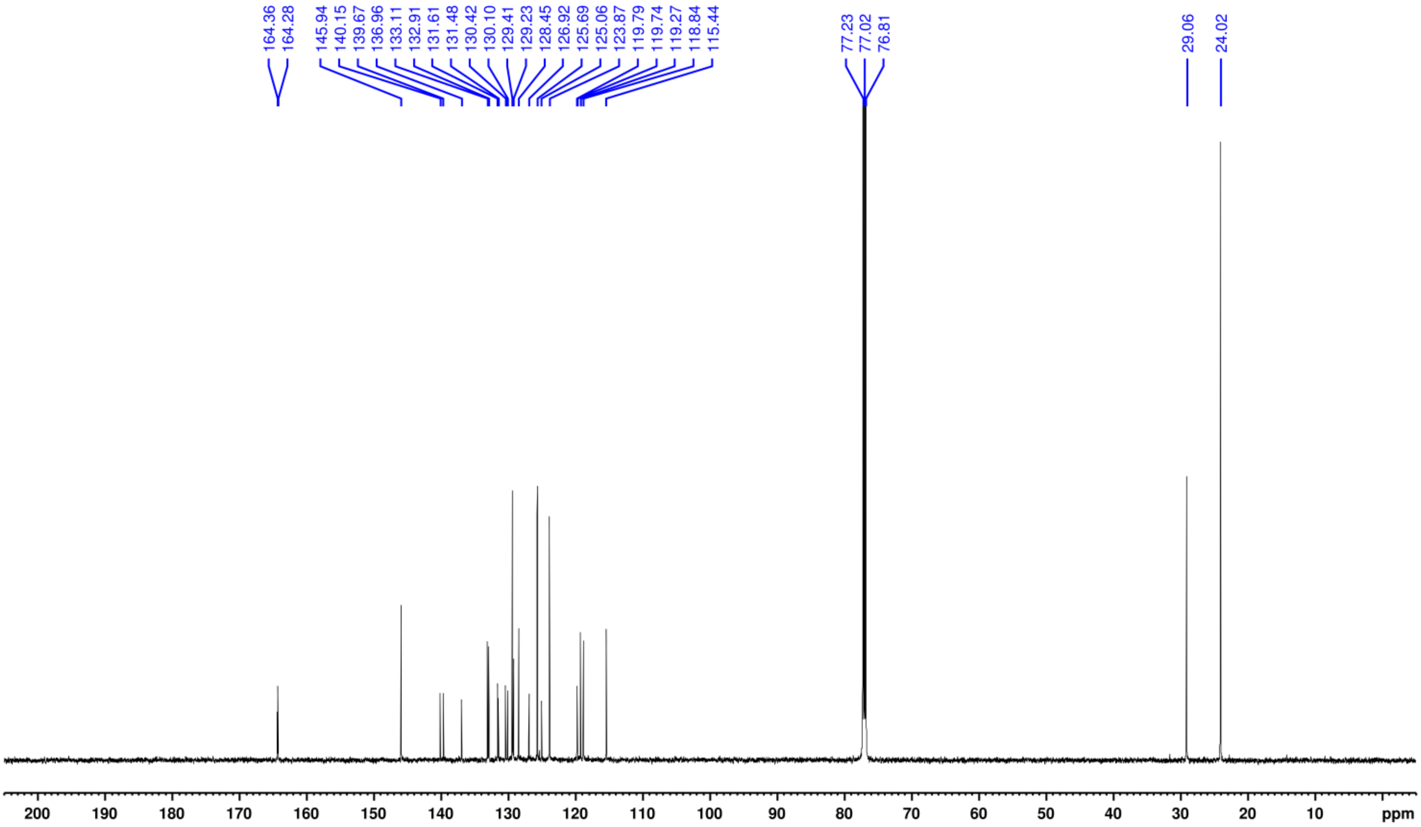

Figure S108. ^{13}C NMR spectrum of **6** (151 MHz, chloroform-*d*, 300 K).

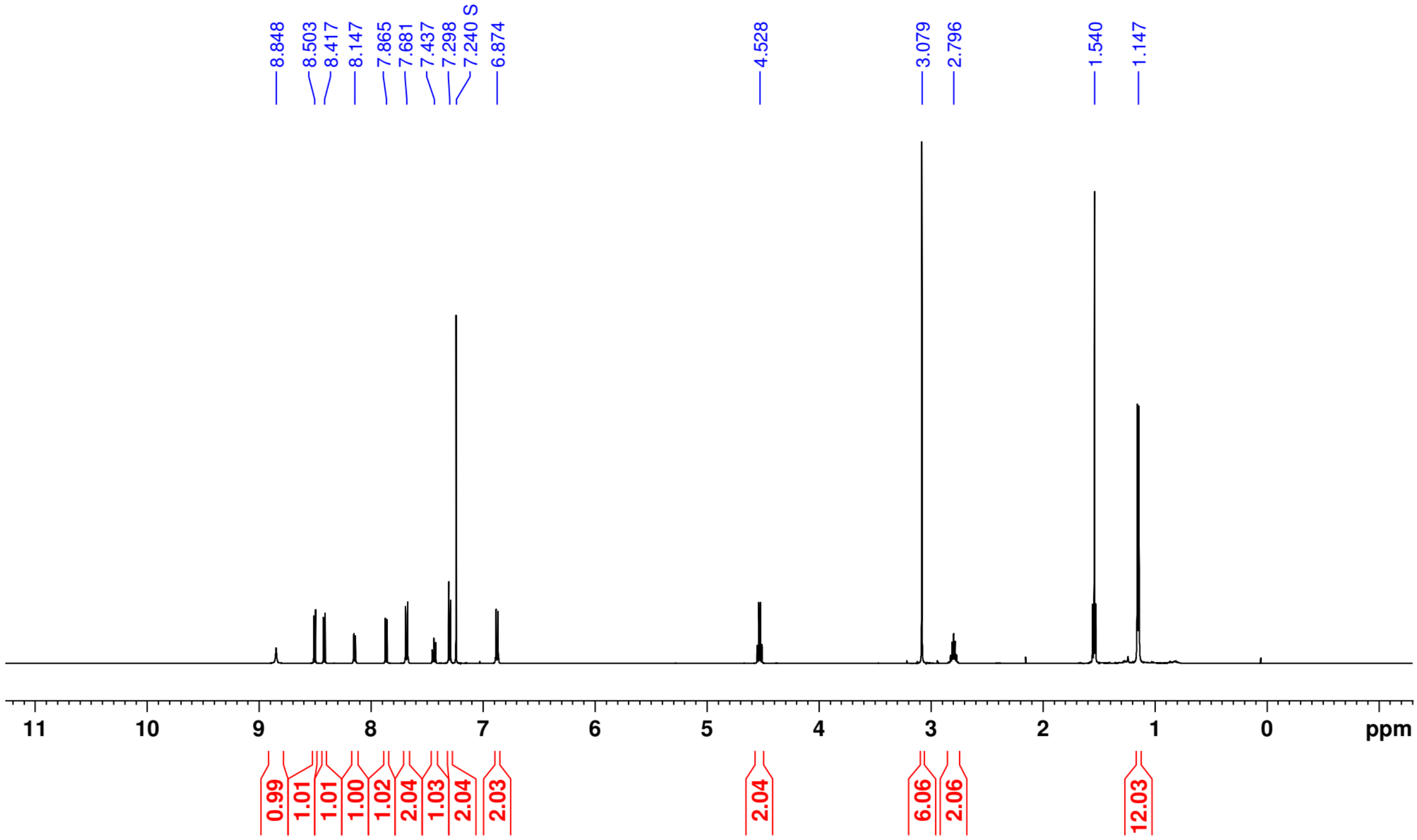

Figure S109. ^{1}H NMR spectrum of **5** (500 MHz, chloroform-*d*, 300 K).

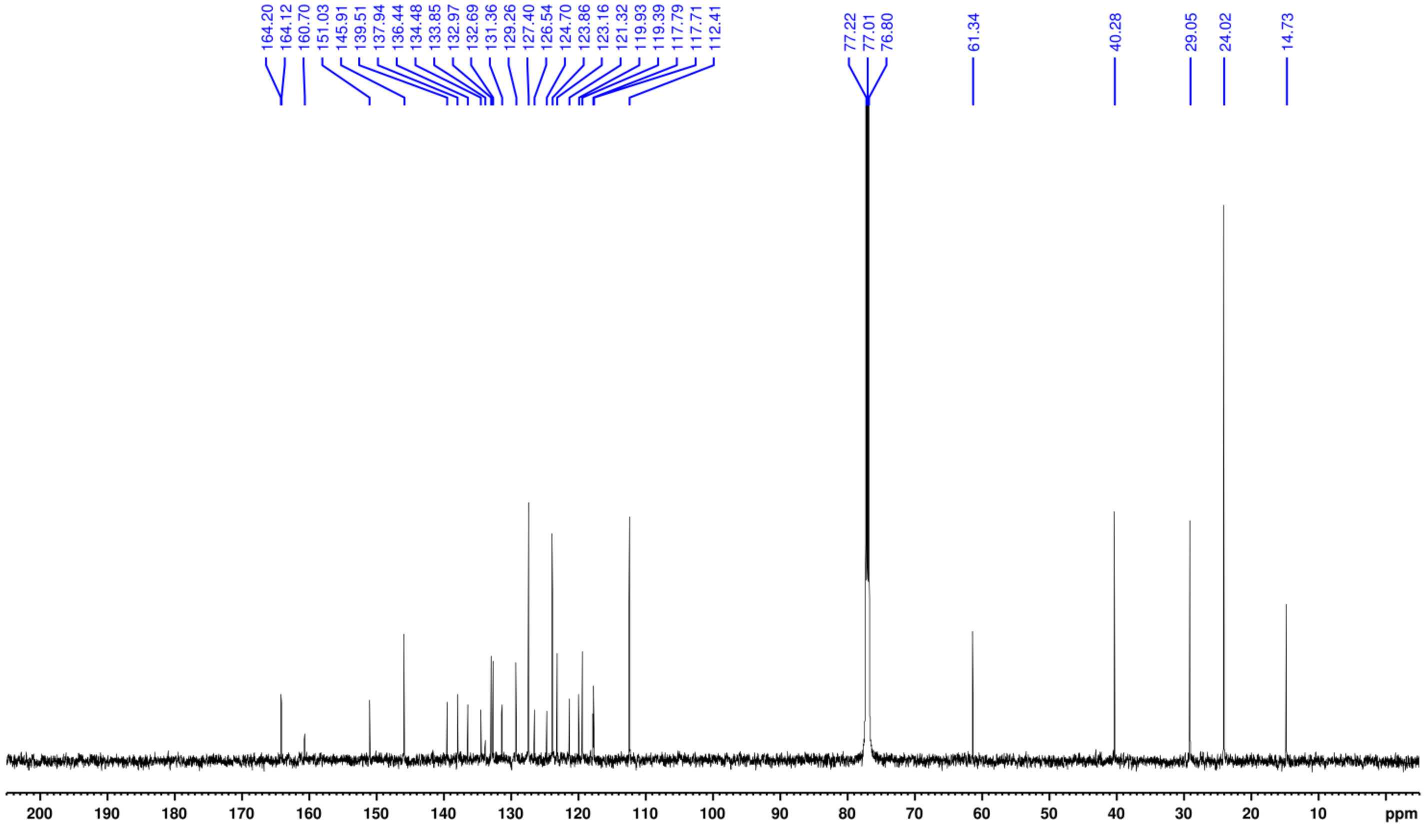

Figure S110. ^{13}C NMR spectrum of **5** (151 MHz, chloroform-*d*, 300 K).

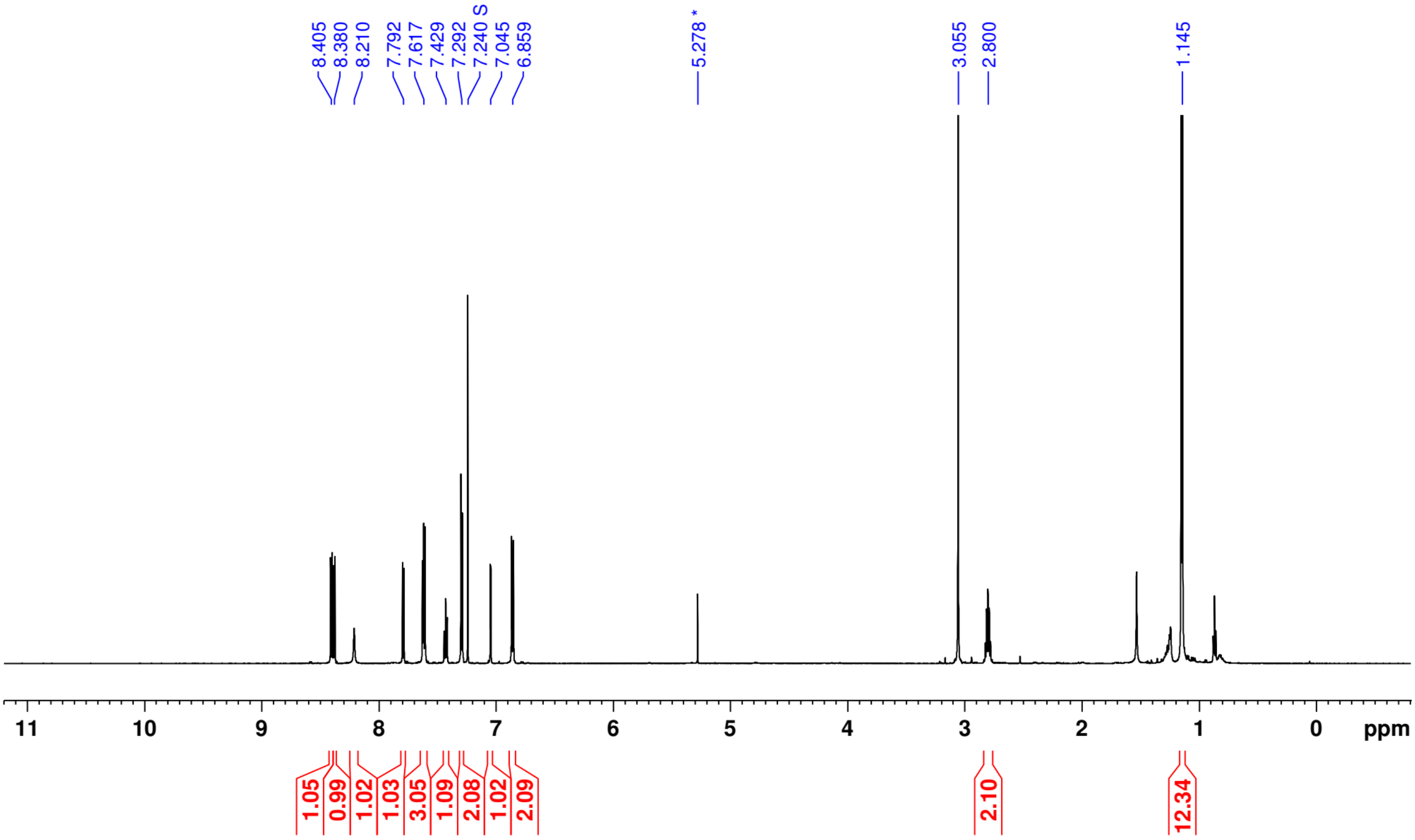

Figure S111. ^{1}H NMR spectrum of **7** (600 MHz, chloroform-*d*, 300 K).

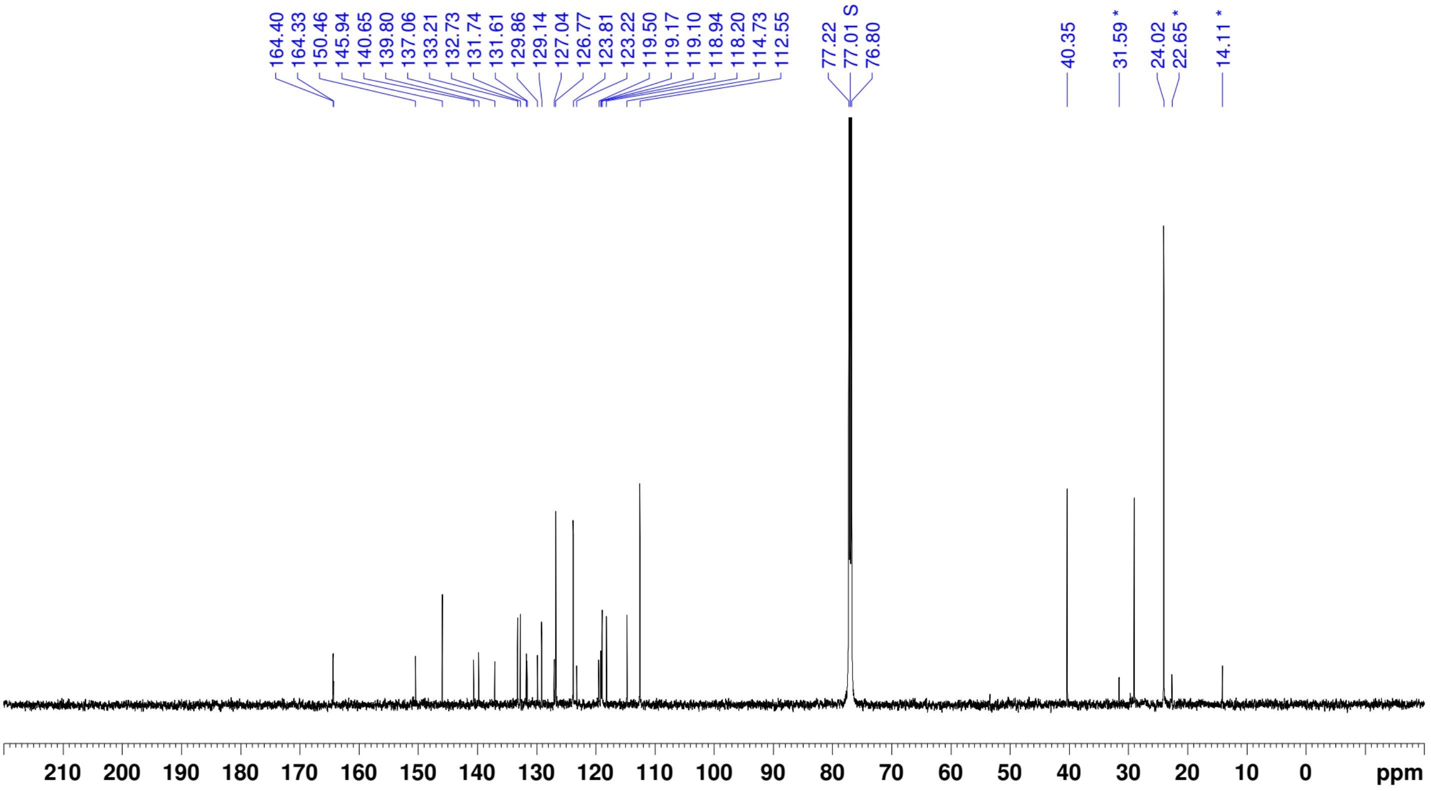

Figure S112. ^{13}C NMR spectrum of **7** (151 MHz, chloroform-*d*, 300 K).

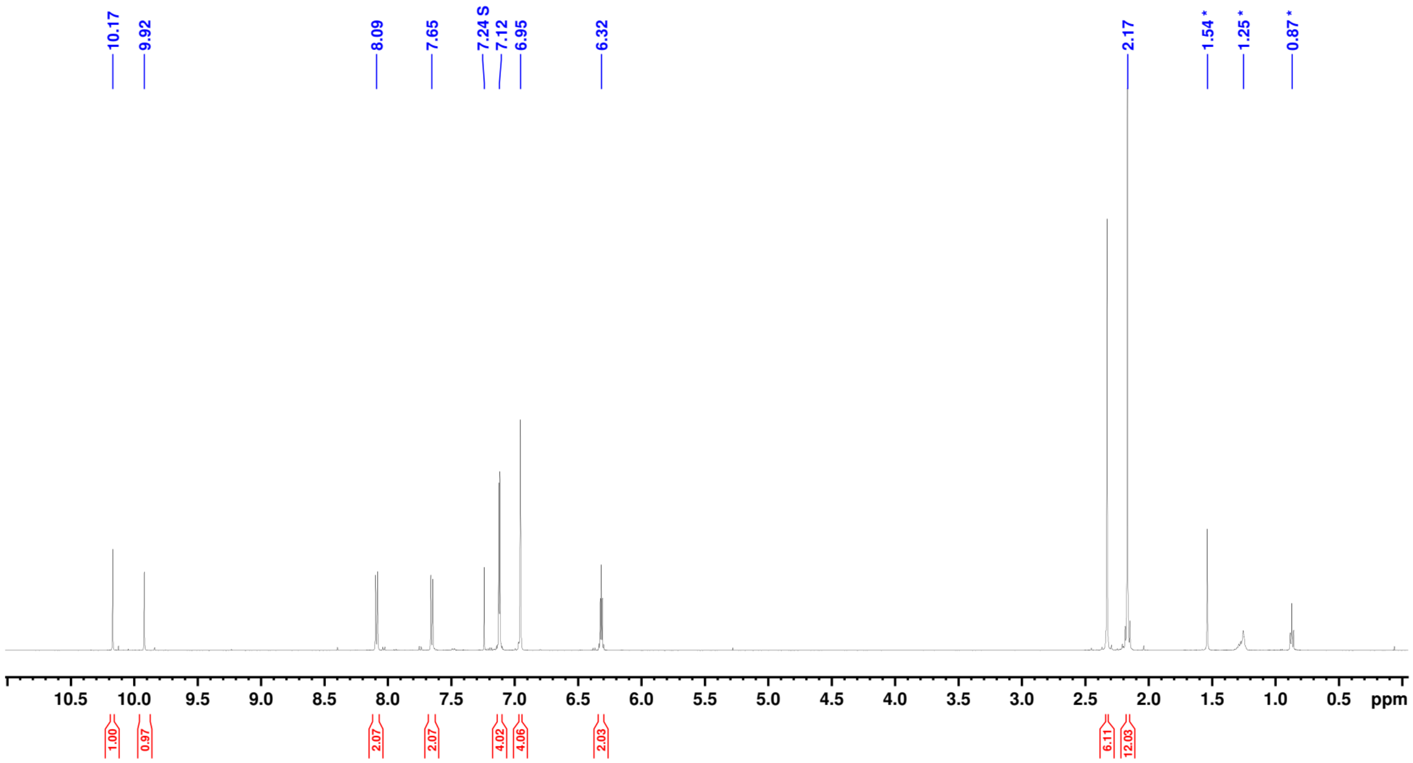

Figure S113. ^{1}H NMR spectrum of **S3** (600 MHz, chloroform-*d*, 300 K).

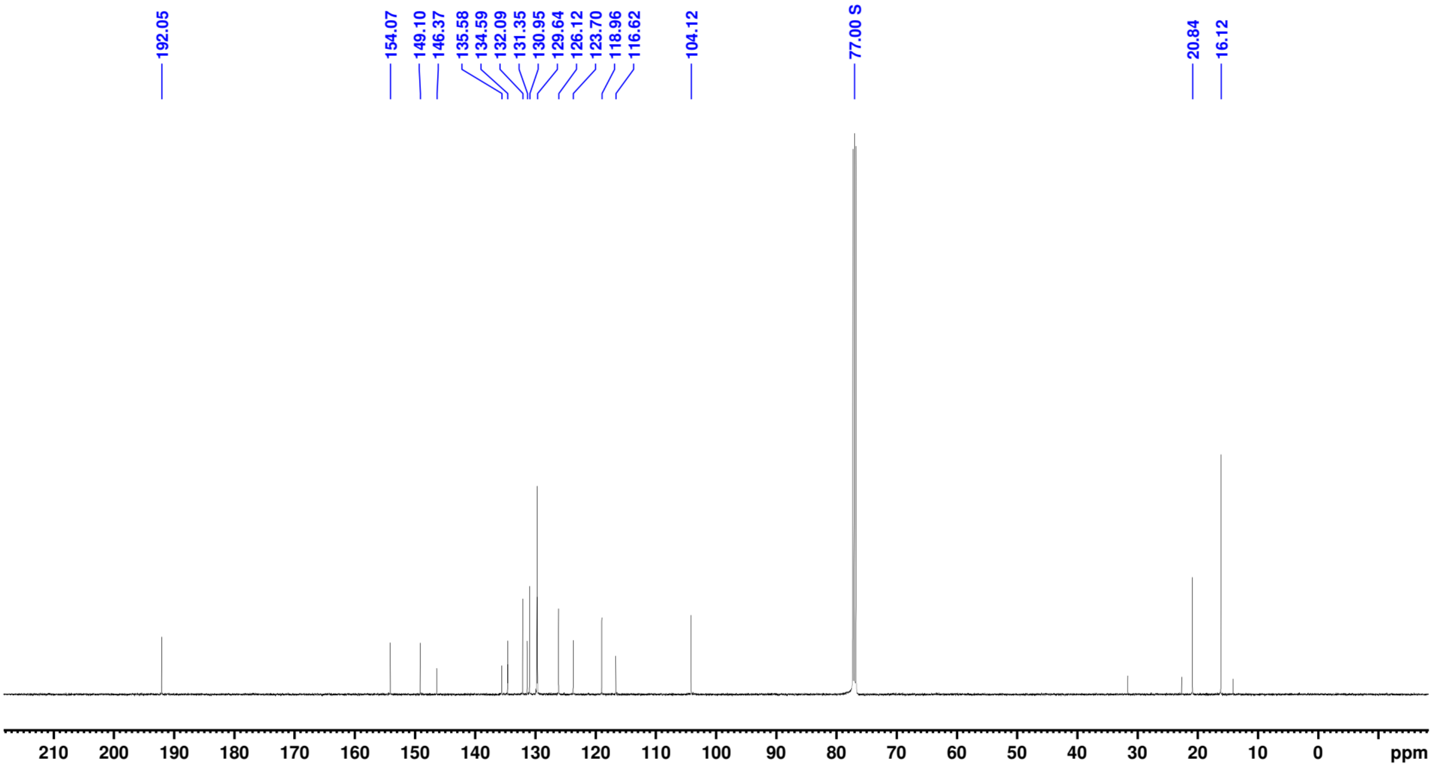

Figure S114. ^{13}C NMR spectrum of **S3** (151 MHz, chloroform-*d*, 300 K).

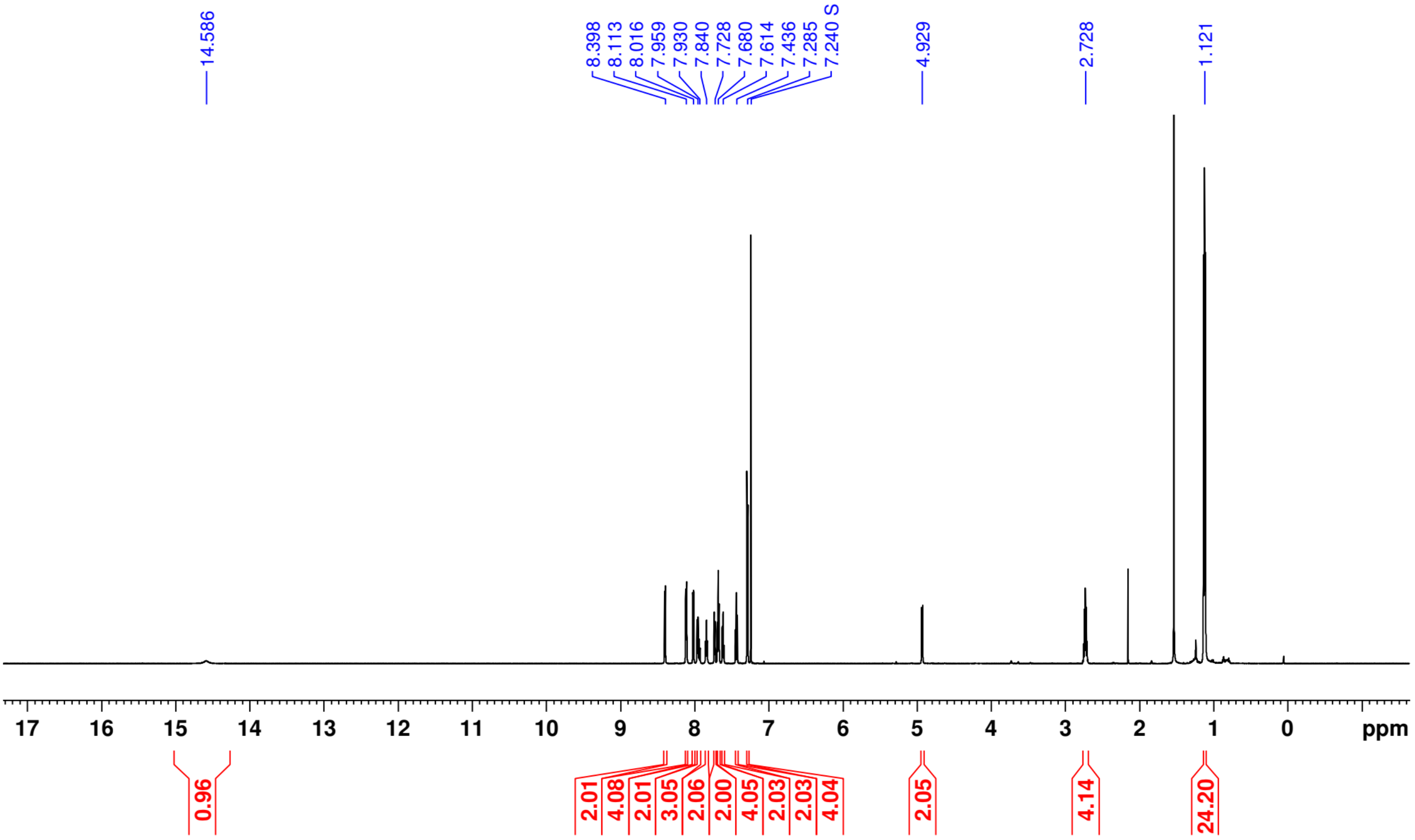

Figure S115. ^{1}H NMR spectrum of **1a** (600 MHz, chloroform-*d*, 300 K).

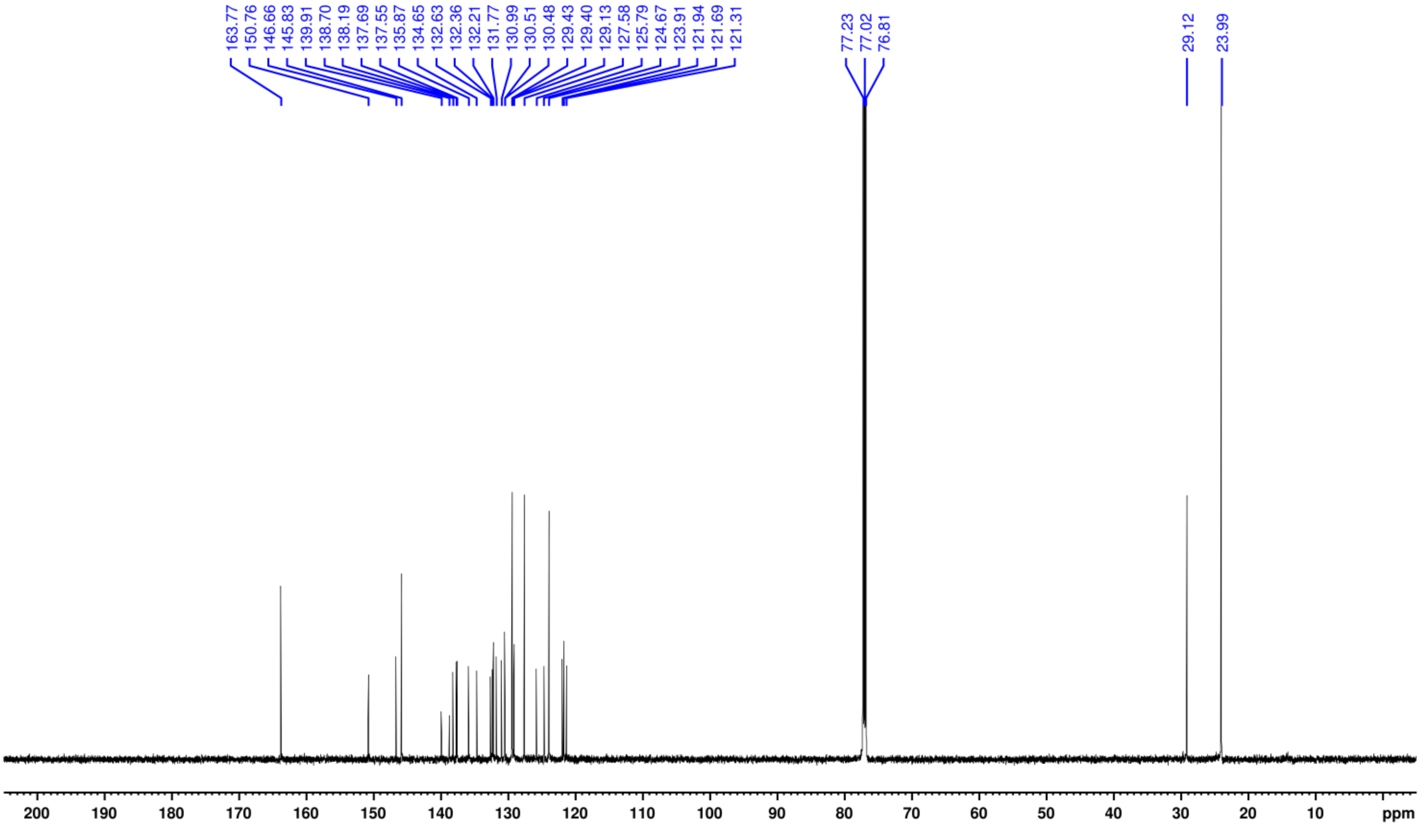

Figure S116. ^{13}C NMR spectrum of **1a** (151 MHz, chloroform-*d*, 300 K).

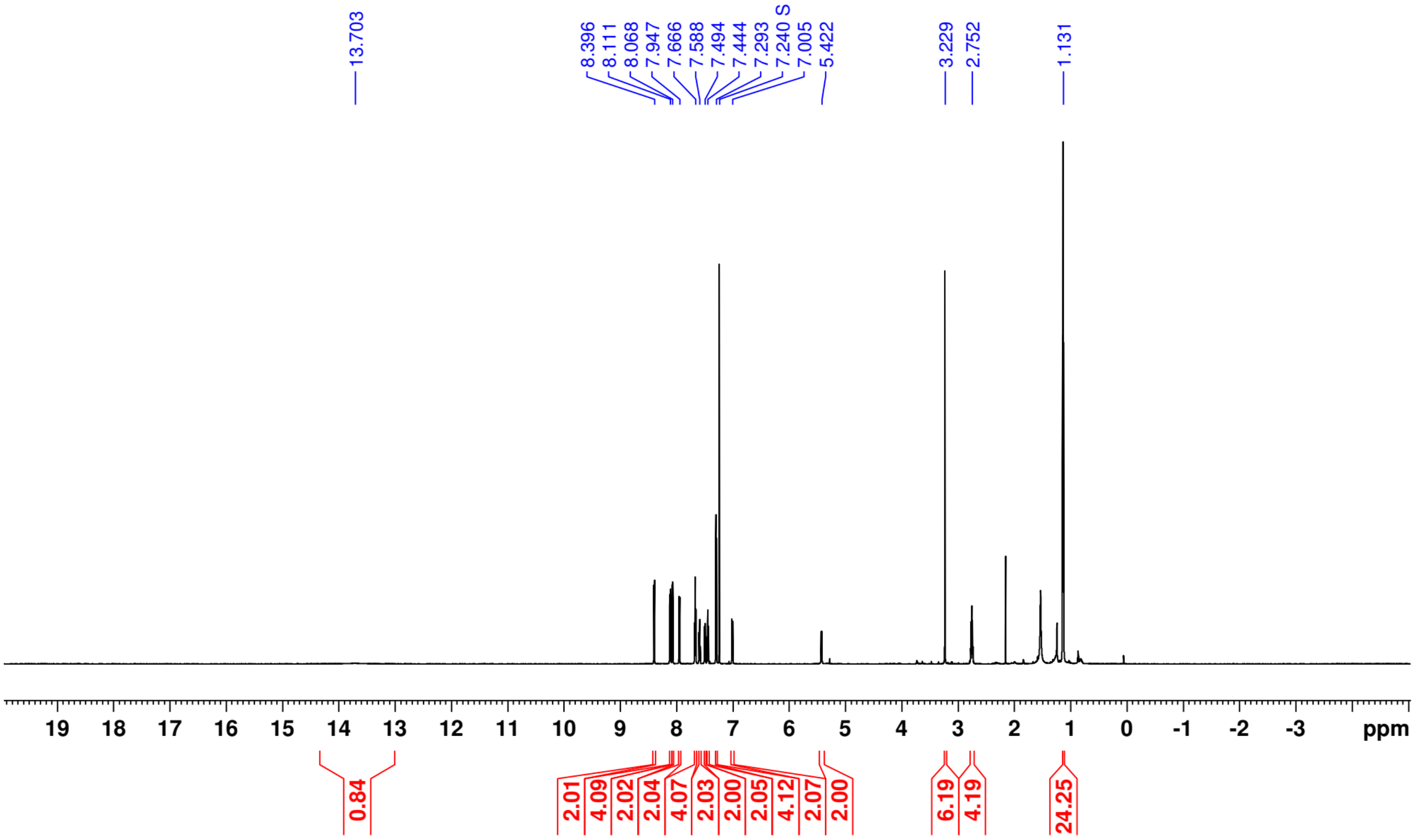

Figure S117. ^{1}H NMR spectrum of **1b** (600 MHz, chloroform-*d*, 300 K).

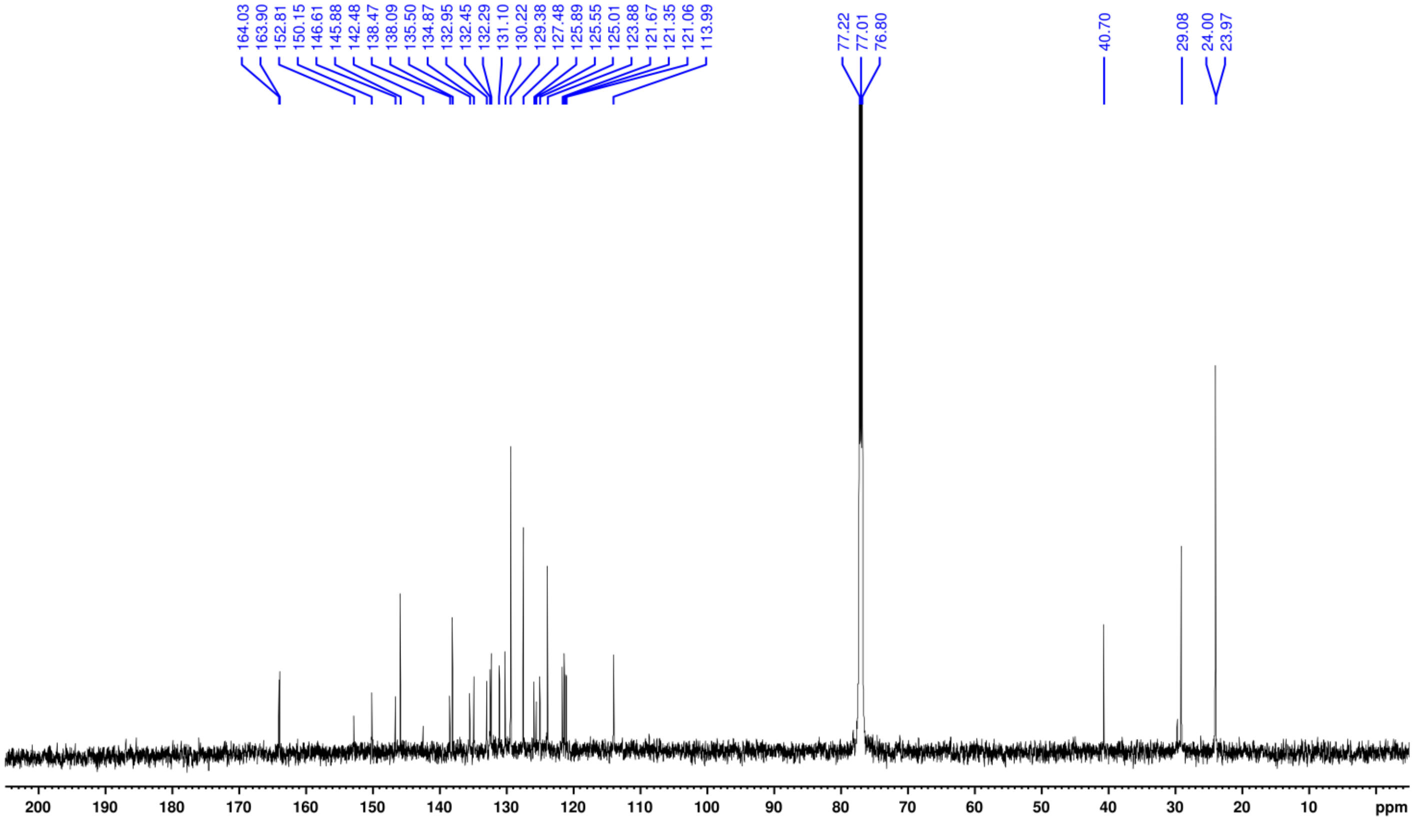

Figure S118. ^{13}C NMR spectrum of **1b** (151 MHz, chloroform-*d*, 300 K).

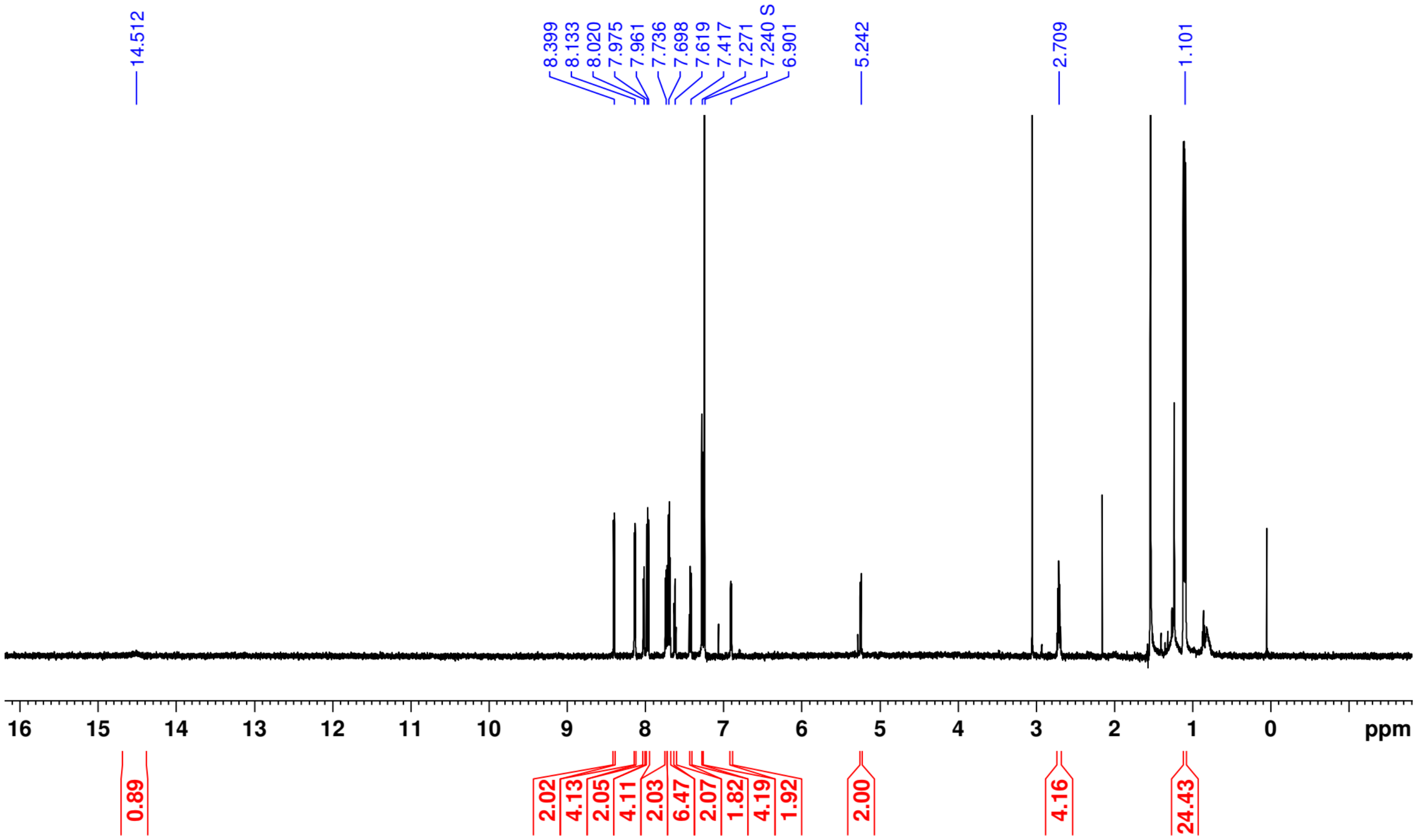

Figure S119. ^{1}H NMR spectrum of **1c** (600 MHz, chloroform-*d*, 300 K).

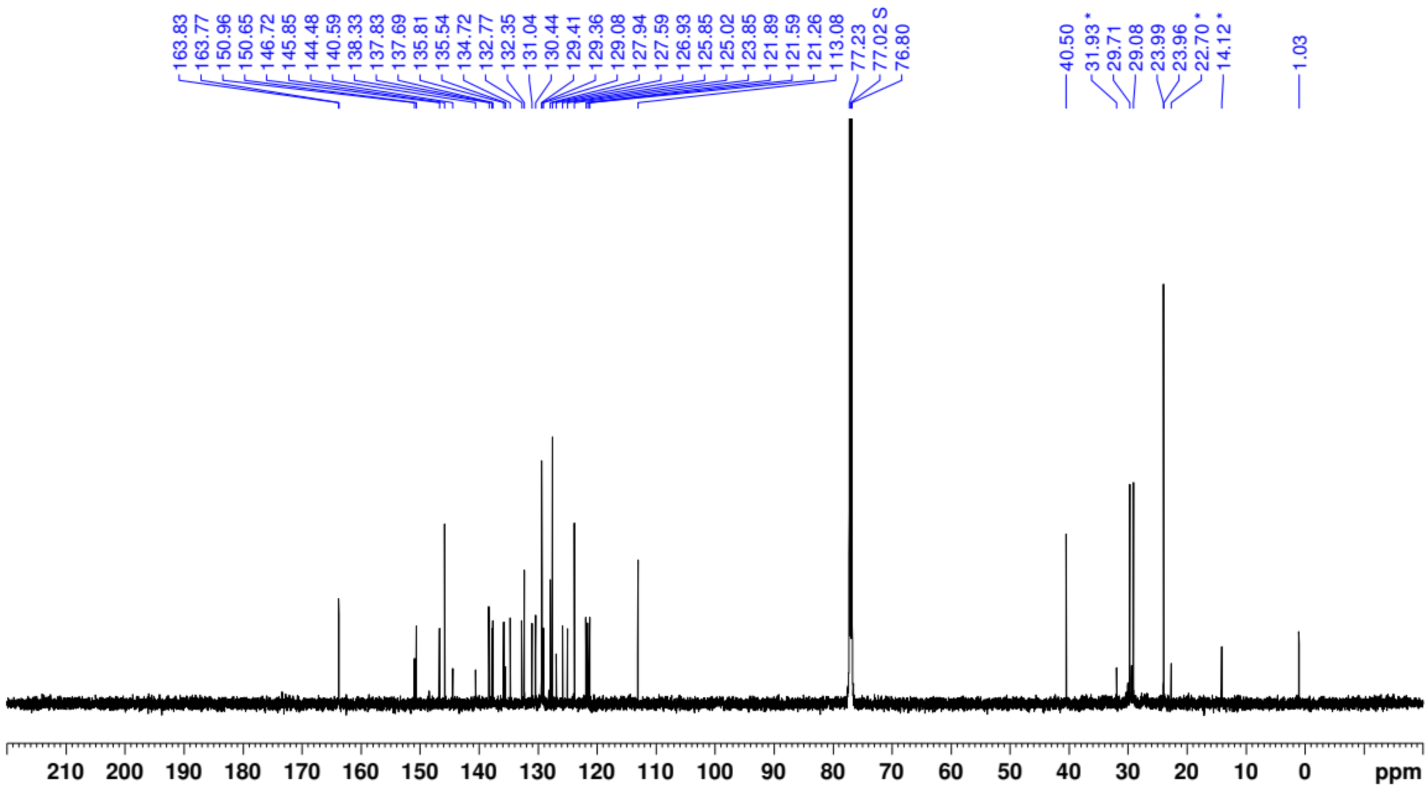

Figure S120. ^{13}C NMR spectrum of **1c** (151 MHz, chloroform-*d*, 300 K).

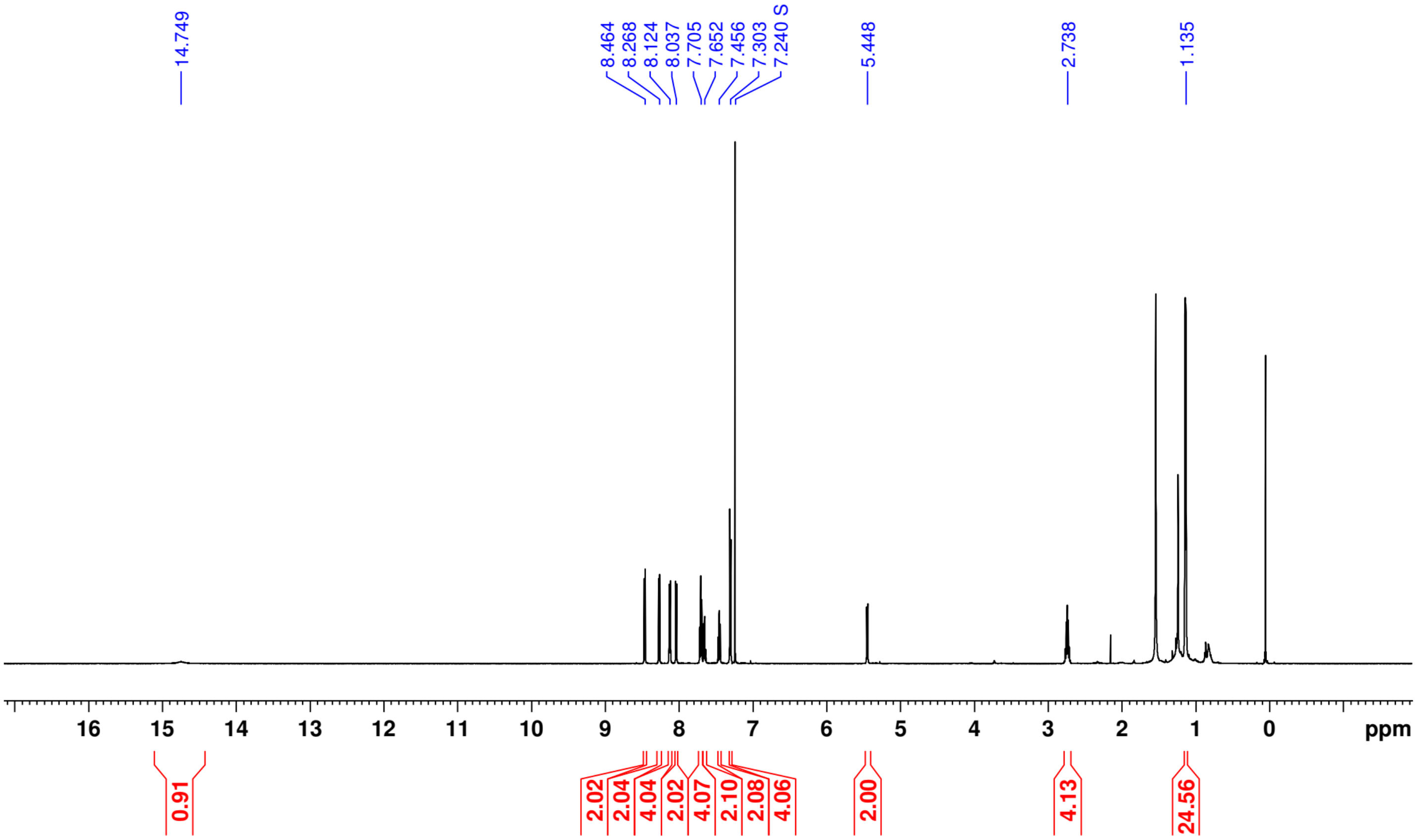

Figure S121. ^{1}H NMR spectrum of **1d** (500 MHz, chloroform-*d*, 300 K).

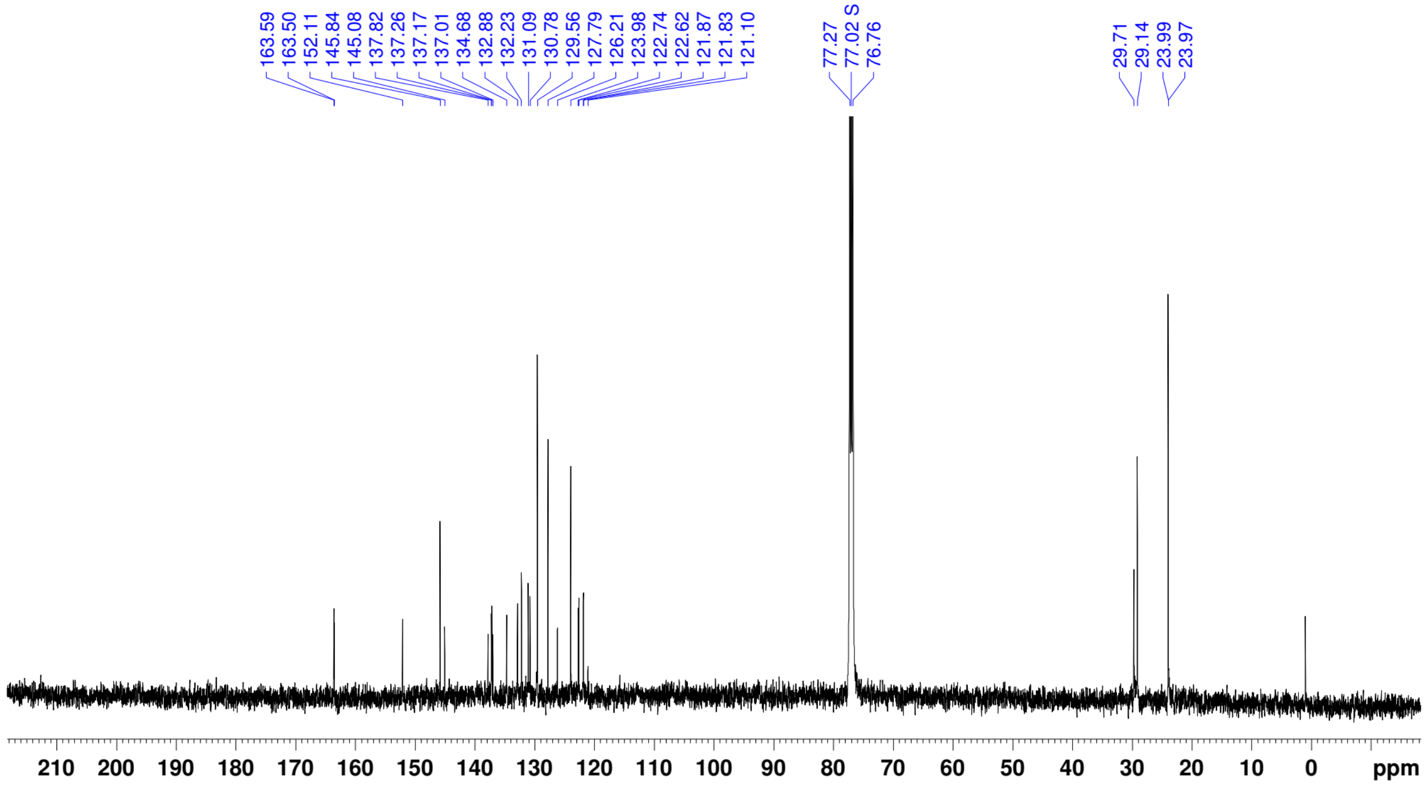

Figure S122. ^{13}C NMR spectrum of **1d** (125 MHz, chloroform-*d*, 300 K).

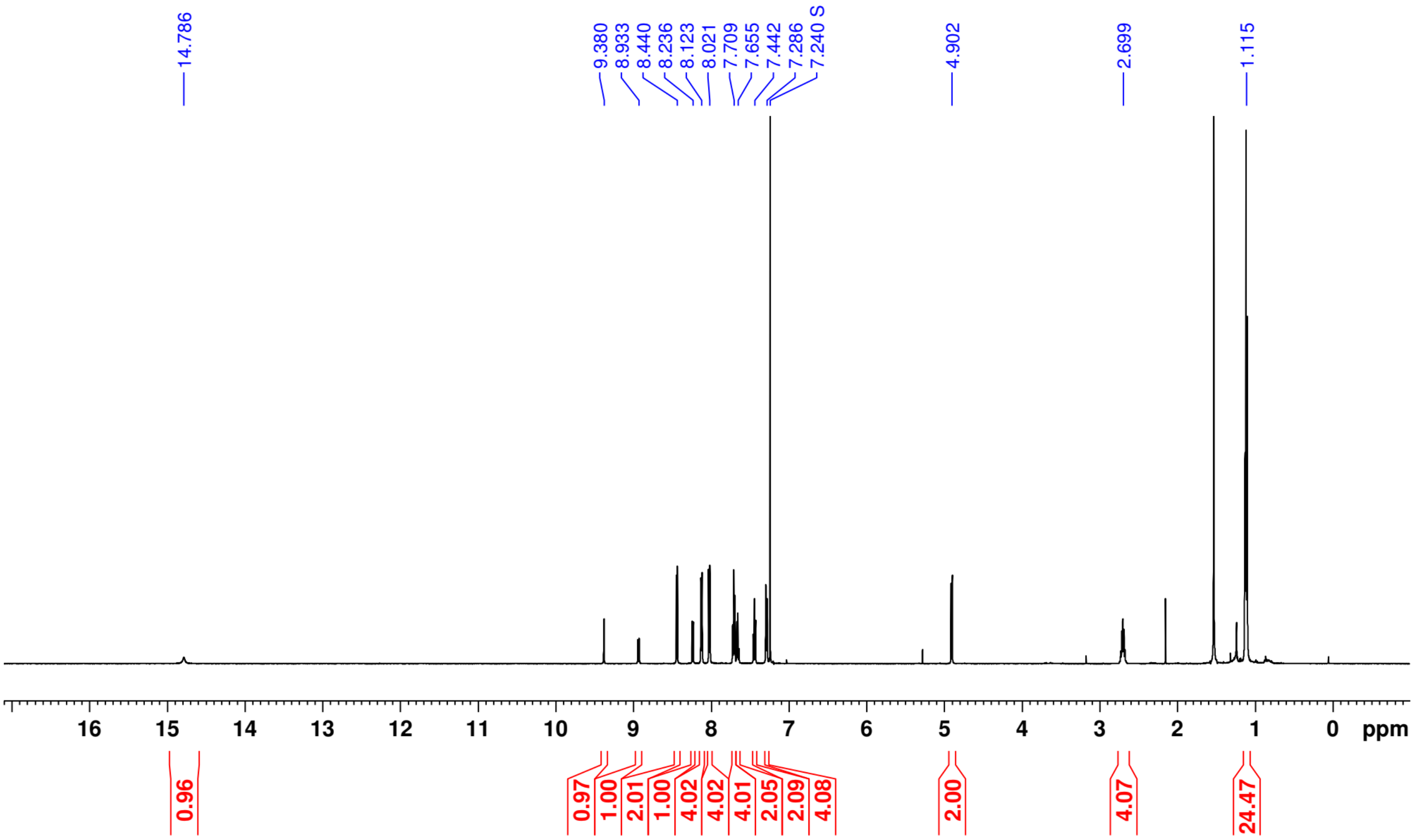

Figure S123. ^{1}H NMR spectrum of **1e** (500 MHz, chloroform-*d*, 300 K).

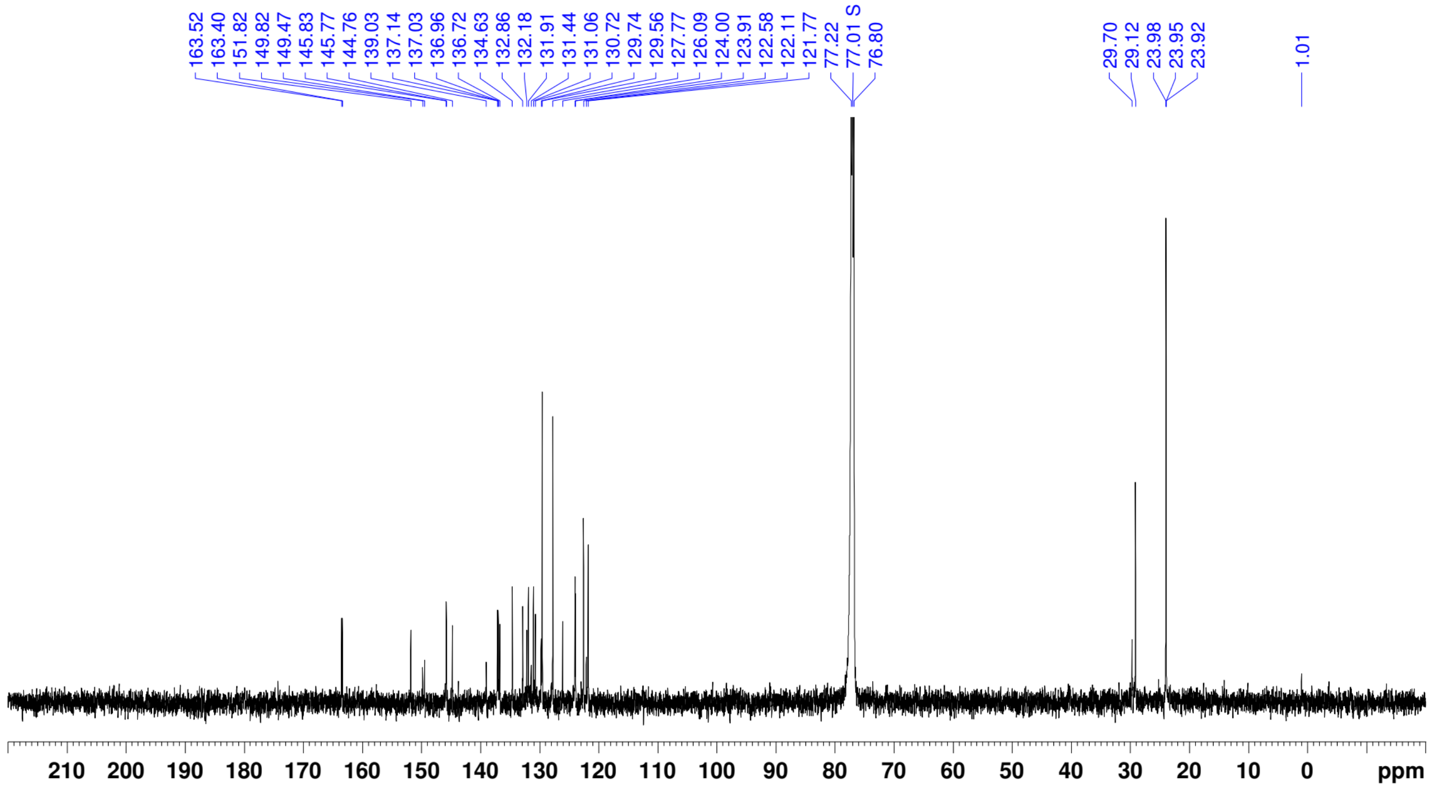

Figure S124. ^{13}C NMR spectrum of **1e** (151 MHz, chloroform-*d*, 300 K).

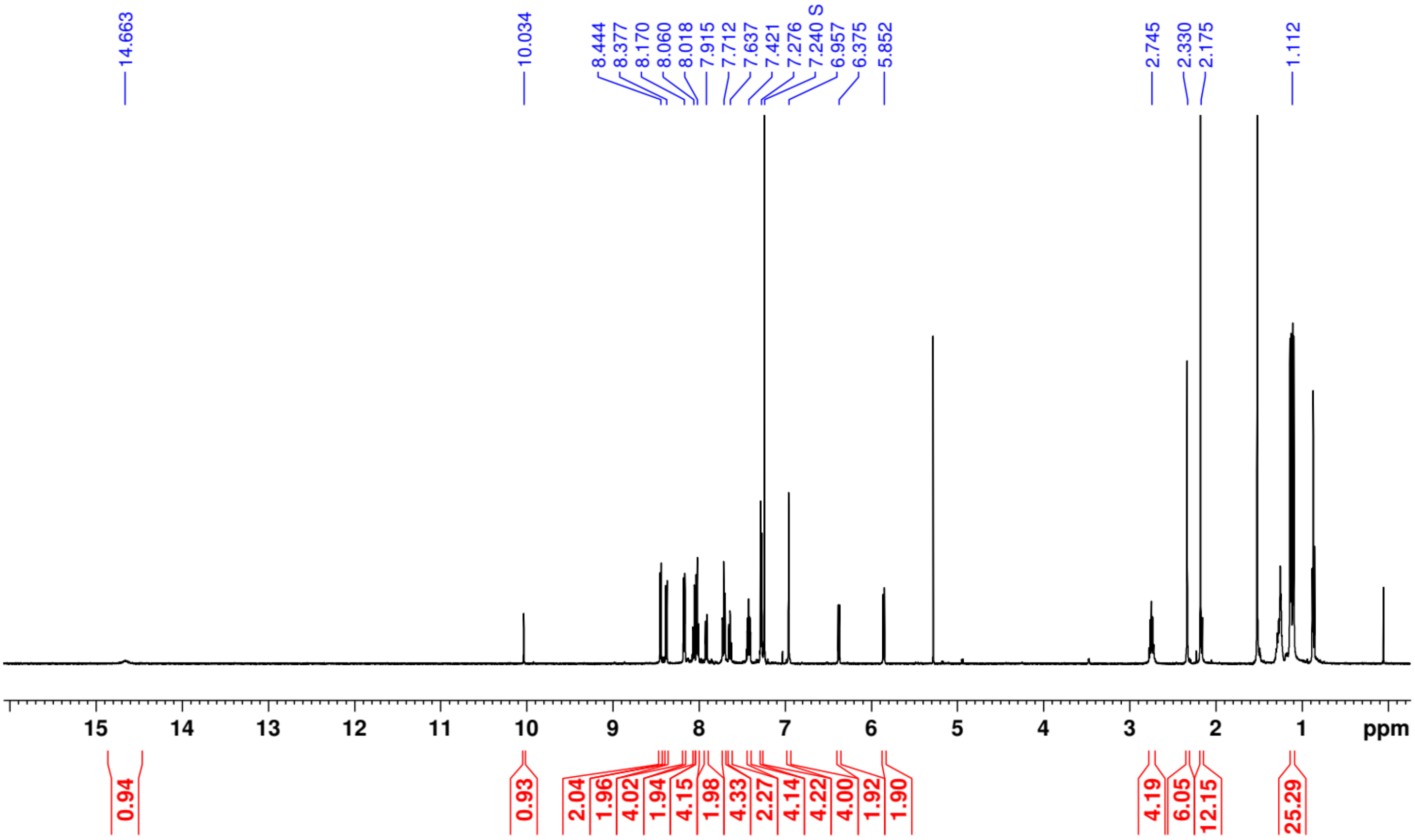

Figure S125. ^{1}H NMR spectrum of **1f** (500 MHz, chloroform-*d*, 300 K).

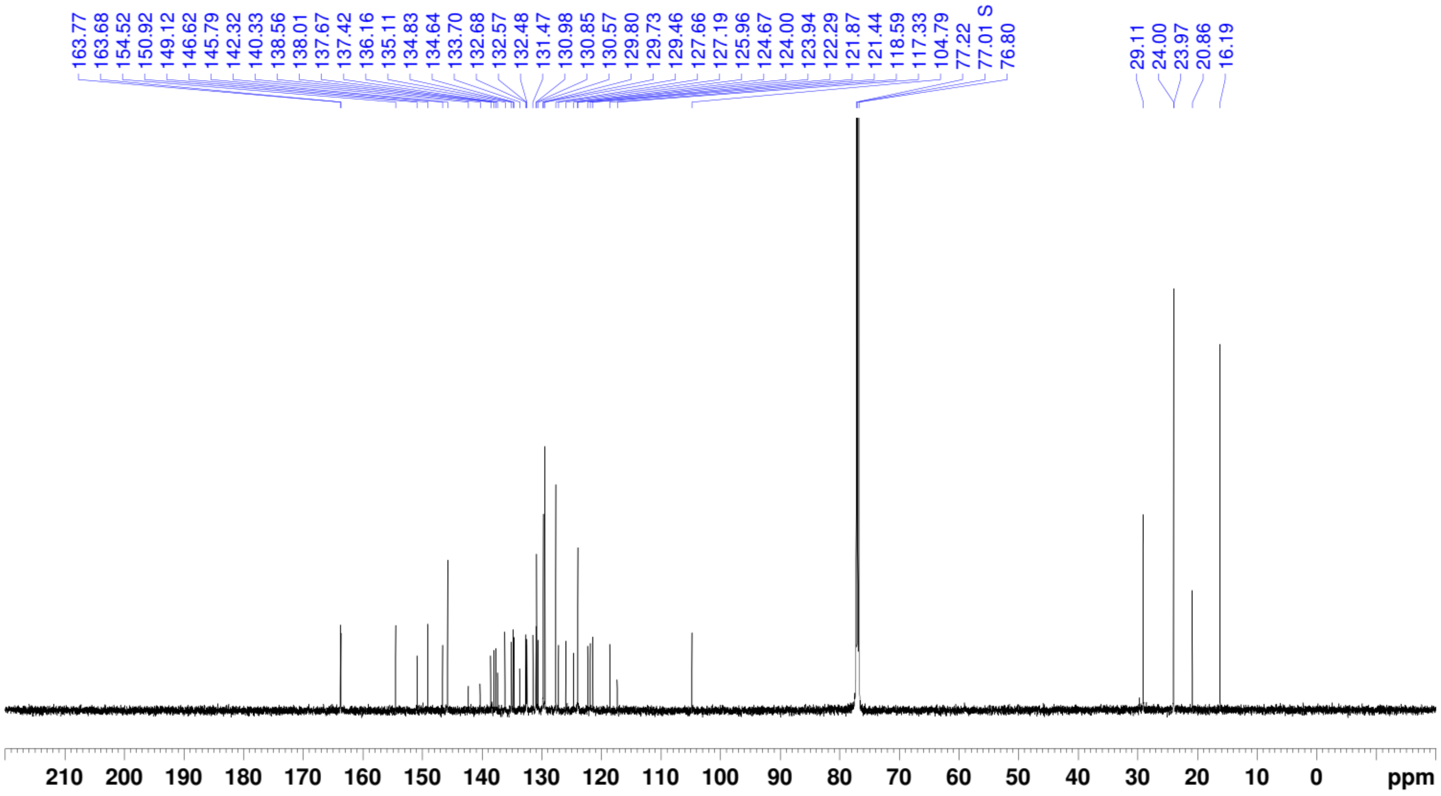

Figure S126. ^{13}C NMR spectrum of **1f** (151 MHz, chloroform-*d*, 300 K).

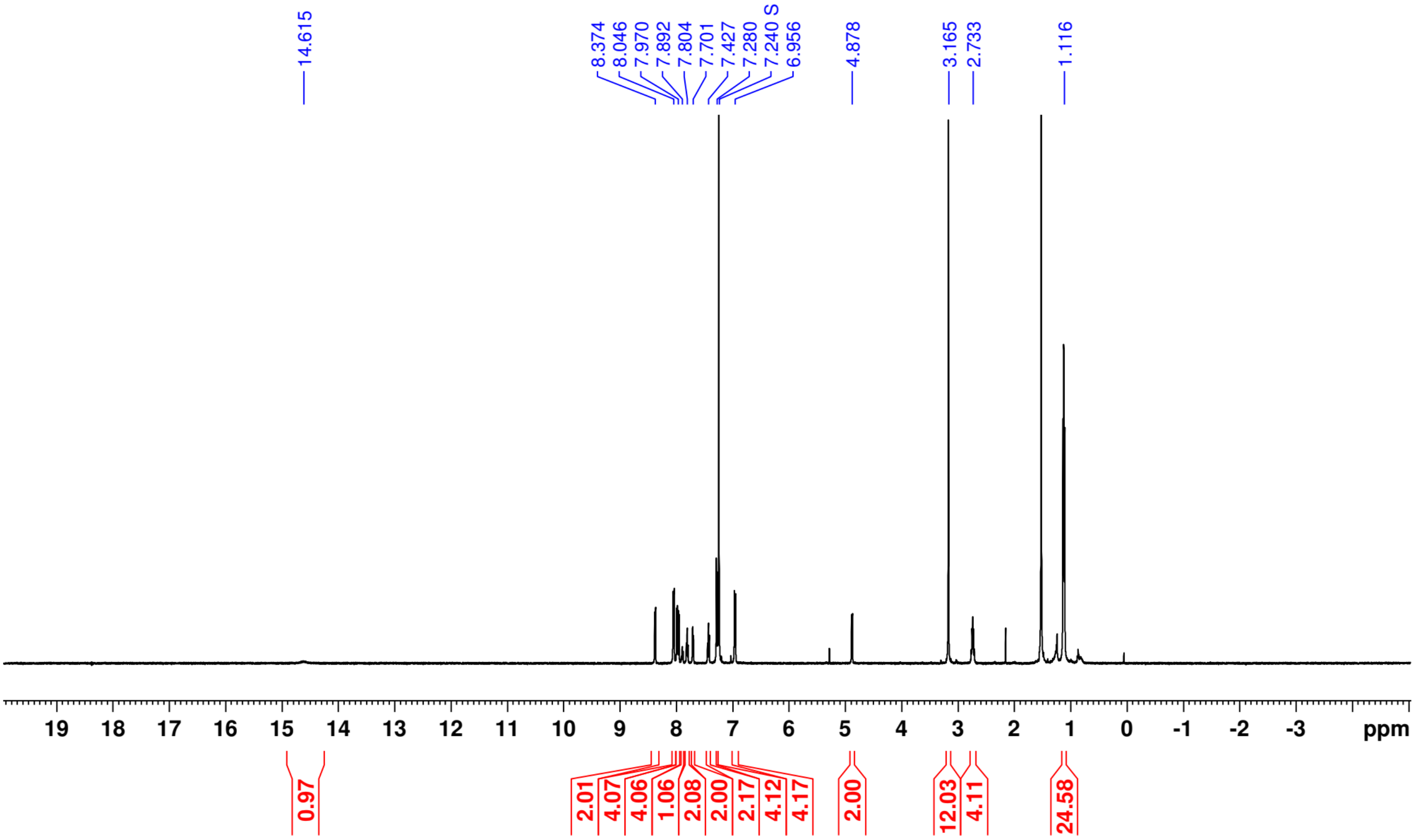

Figure S127. ^{1}H NMR spectrum of **2a** (500 MHz, chloroform-*d*, 300 K).

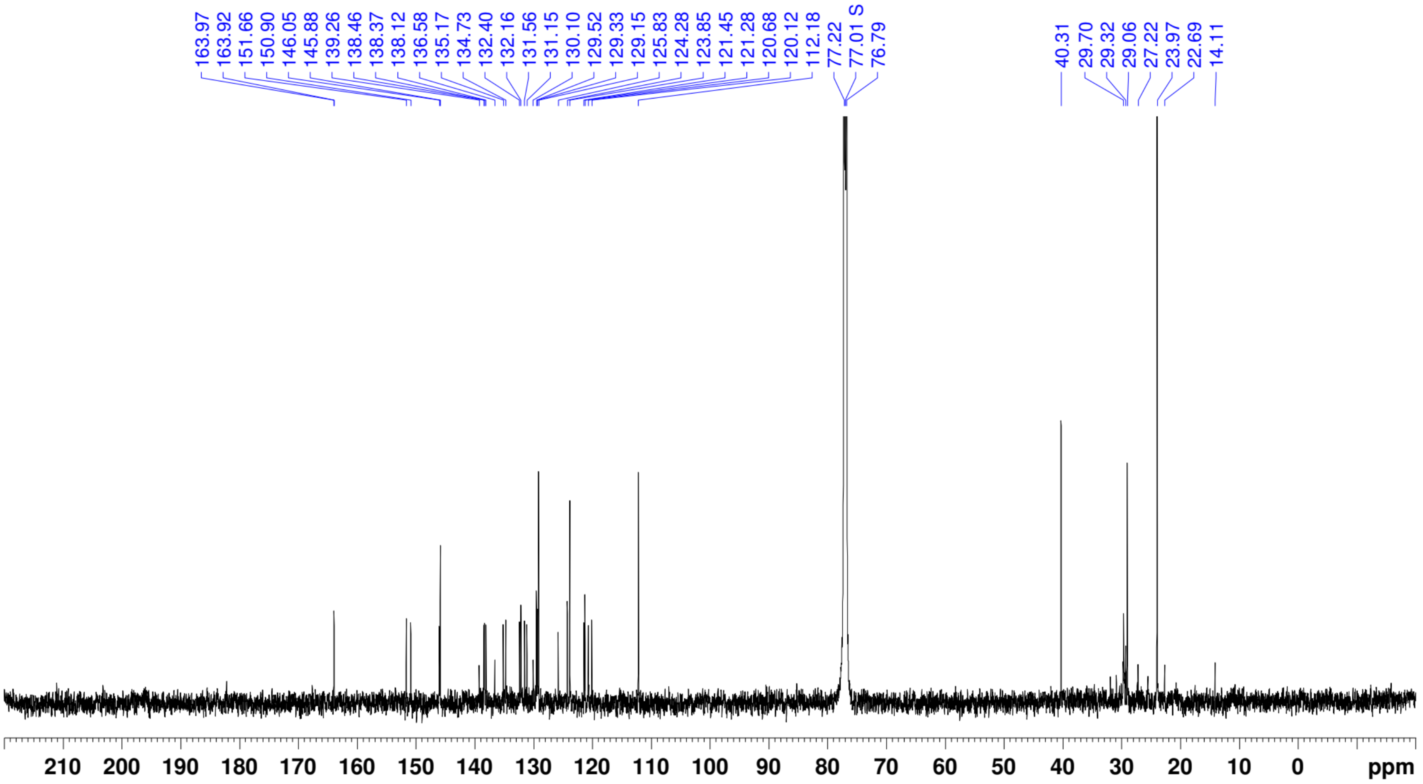

Figure S128. ^{13}C NMR spectrum of **2a** (151 MHz, chloroform-*d*, 300 K).

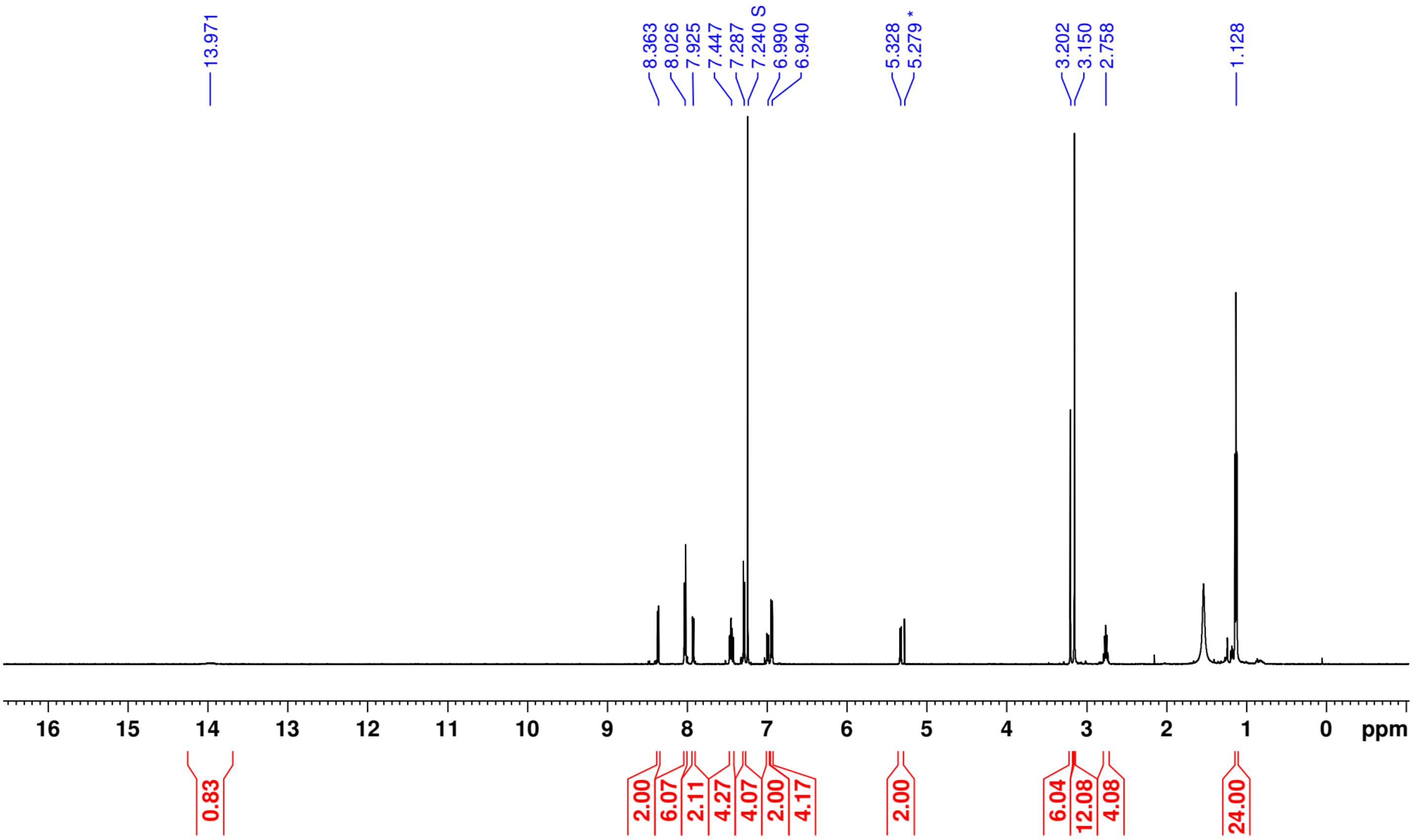

Figure S129. ^{1}H NMR spectrum of **2b** (500 MHz, chloroform-*d*, 300 K).

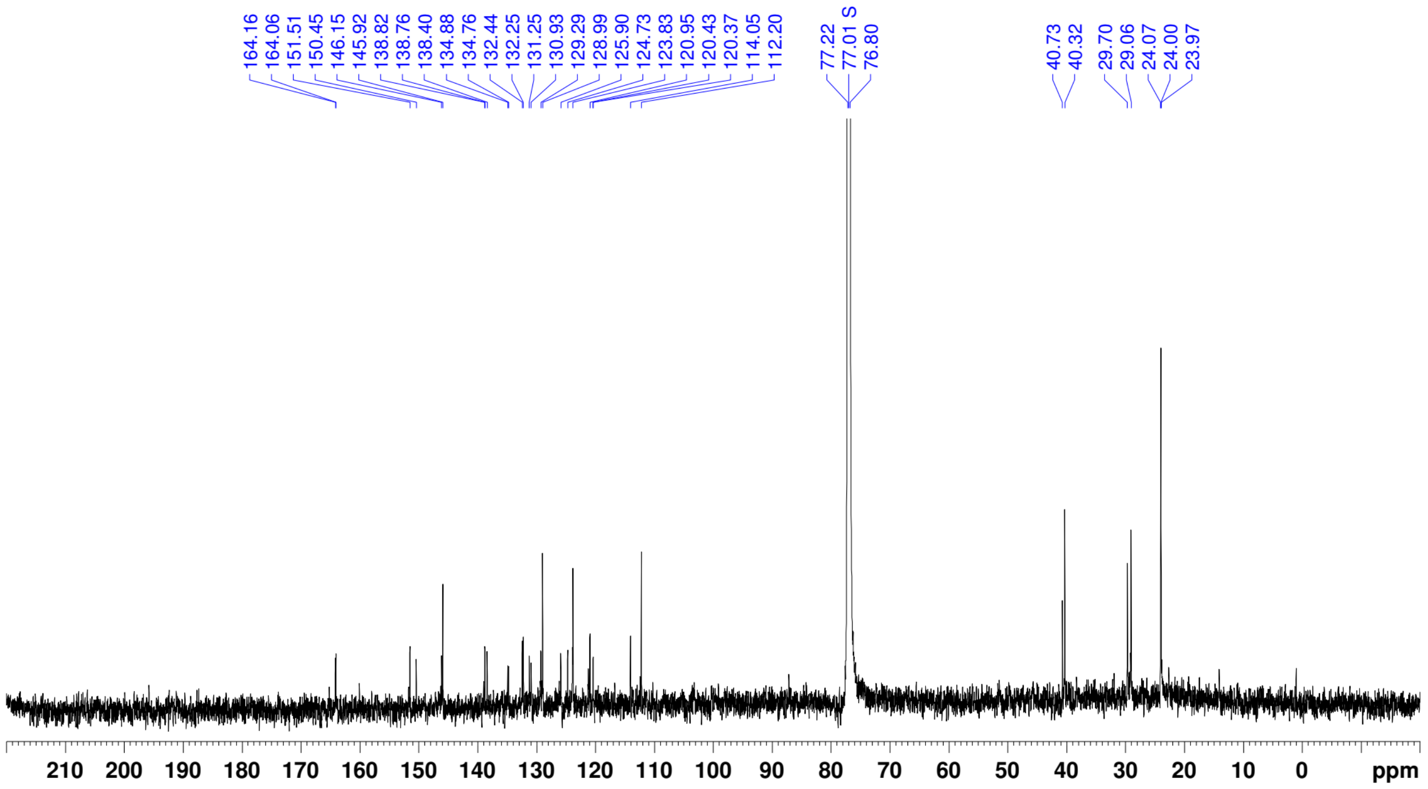

Figure S130. ^{13}C NMR spectrum of **2b** (151 MHz, chloroform-*d*, 300 K).

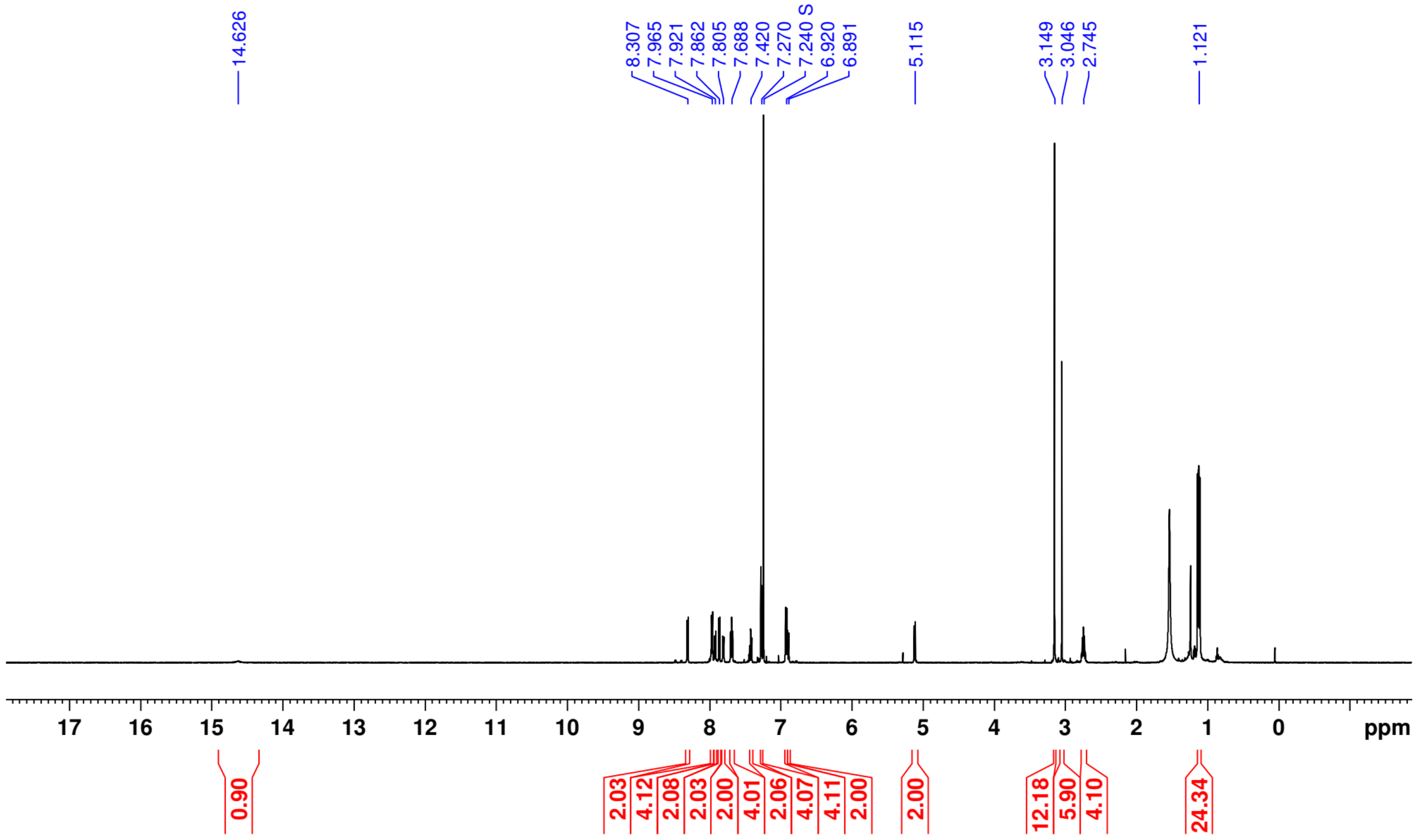

Figure S131. ^{1}H NMR spectrum of **2c** (500 MHz, chloroform-*d*, 300 K).

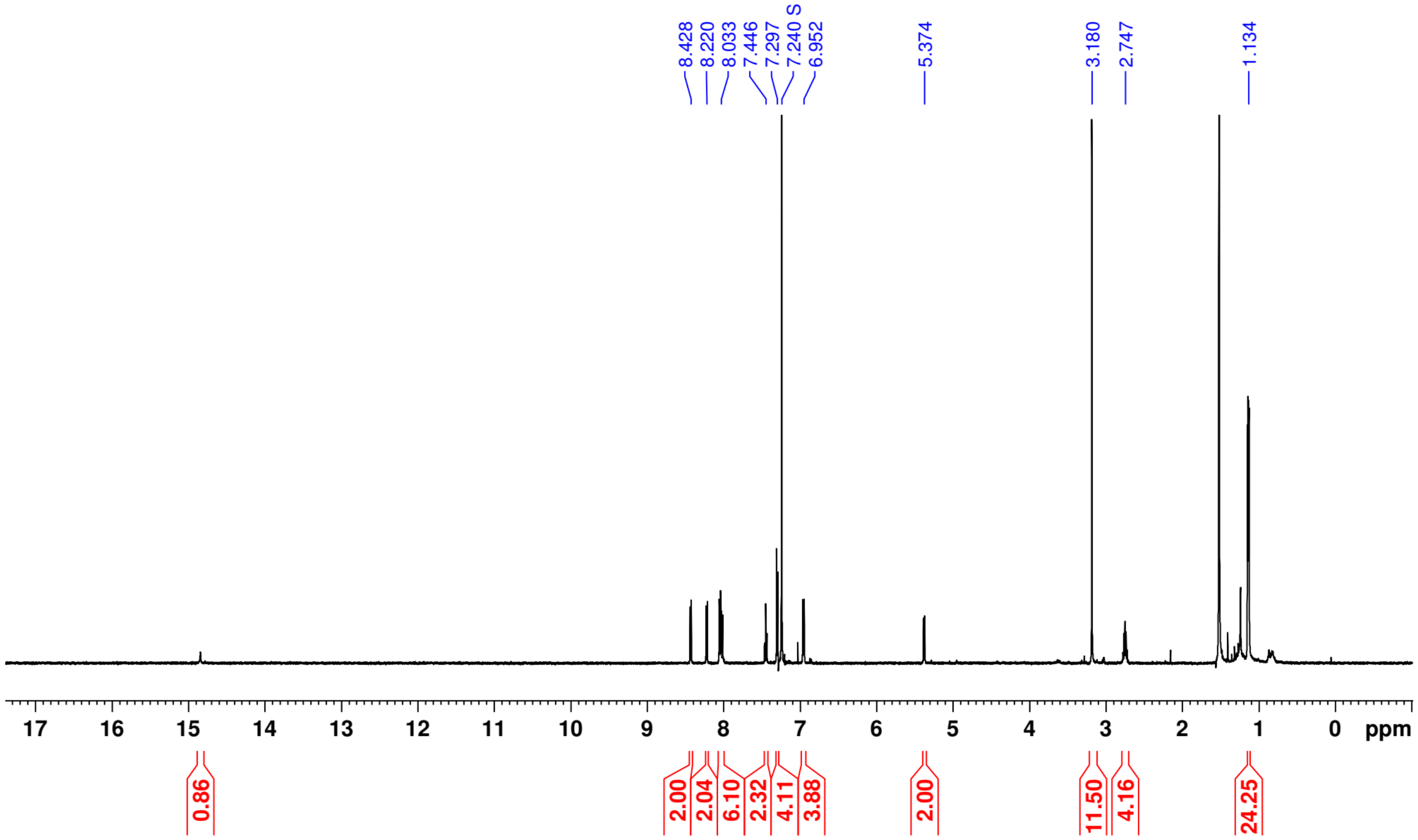

Figure S132. ^{1}H NMR spectrum of **2d** (500 MHz, chloroform-*d*, 300 K).

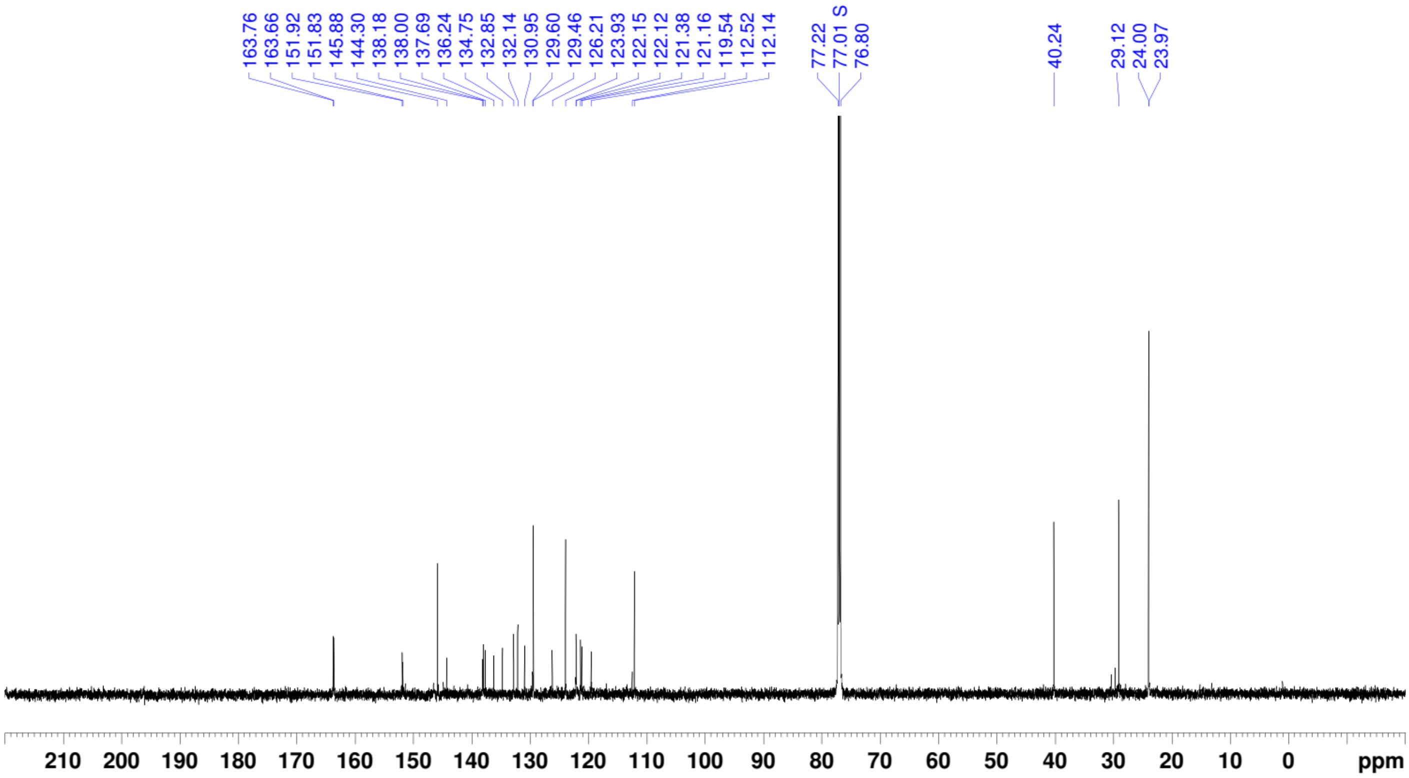

Figure S133. ^{13}C NMR spectrum of **2d** (151 MHz, chloroform-*d*, 300 K).

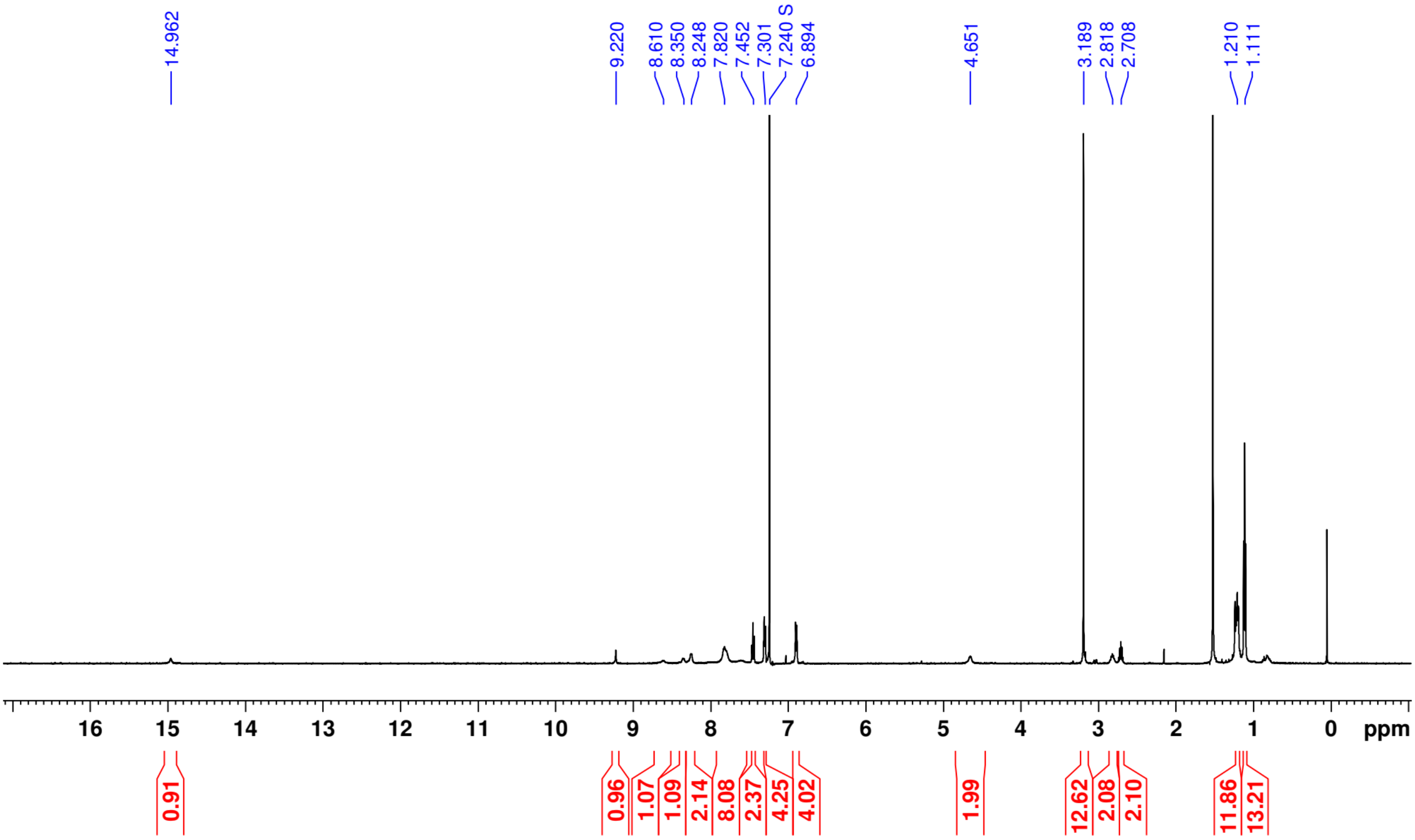

Figure S134. ^{1}H NMR spectrum of **2e** (500 MHz, chloroform-*d*, 300 K).

Mass Spectra

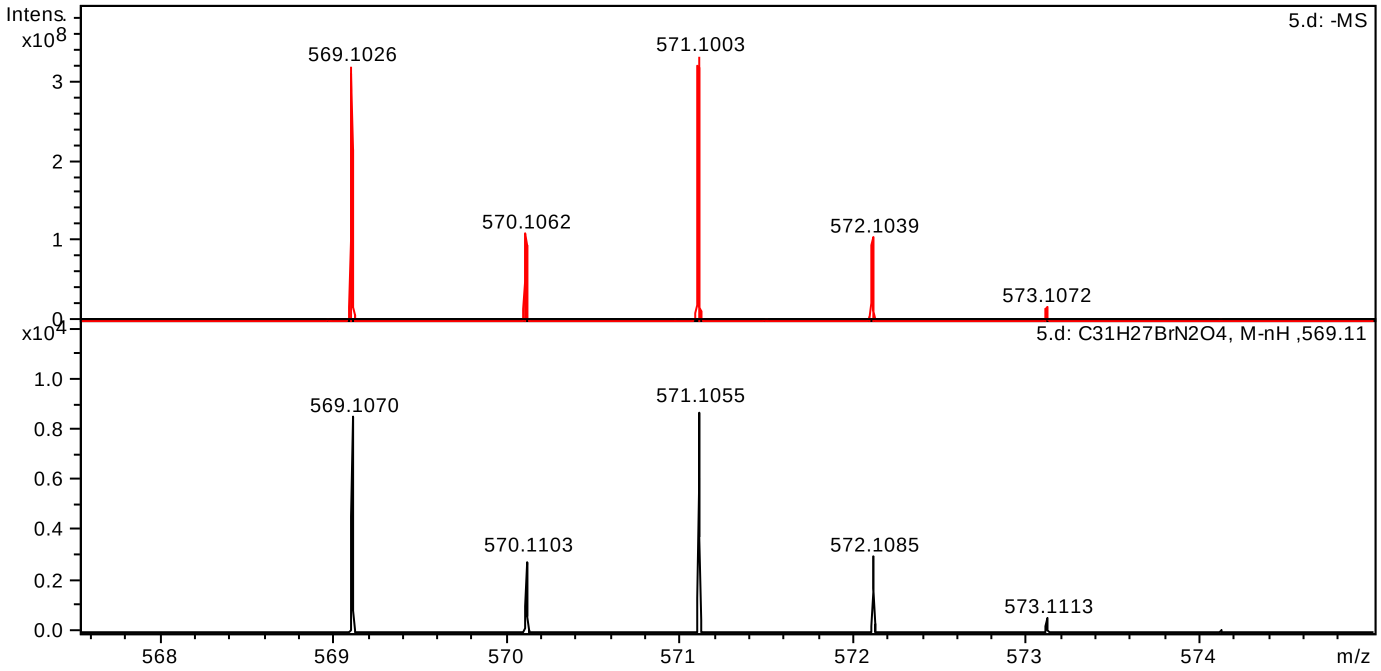

Figure S135. High resolution mass spectrum of **3** (ESI–TOF, top: experimental, bottom: simulated).

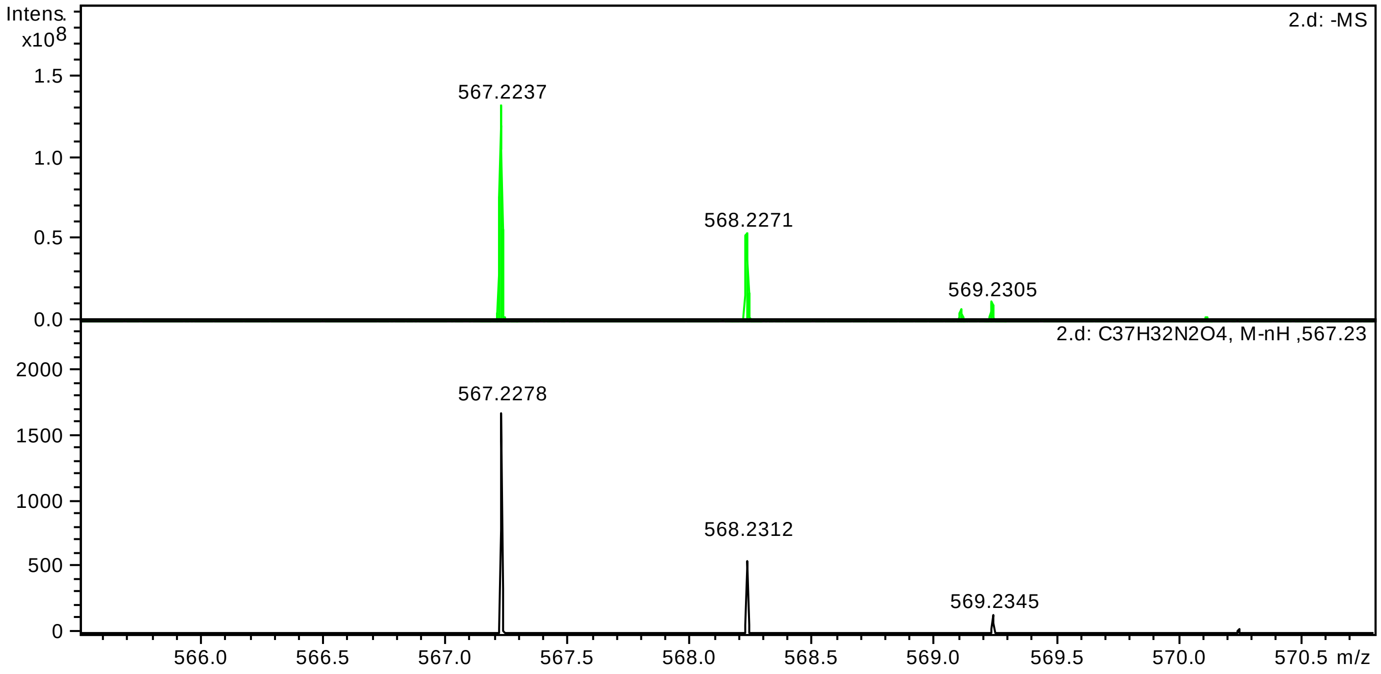

Figure S136. High resolution mass spectrum of **4** (ESI–TOF, top: experimental, bottom: simulated).

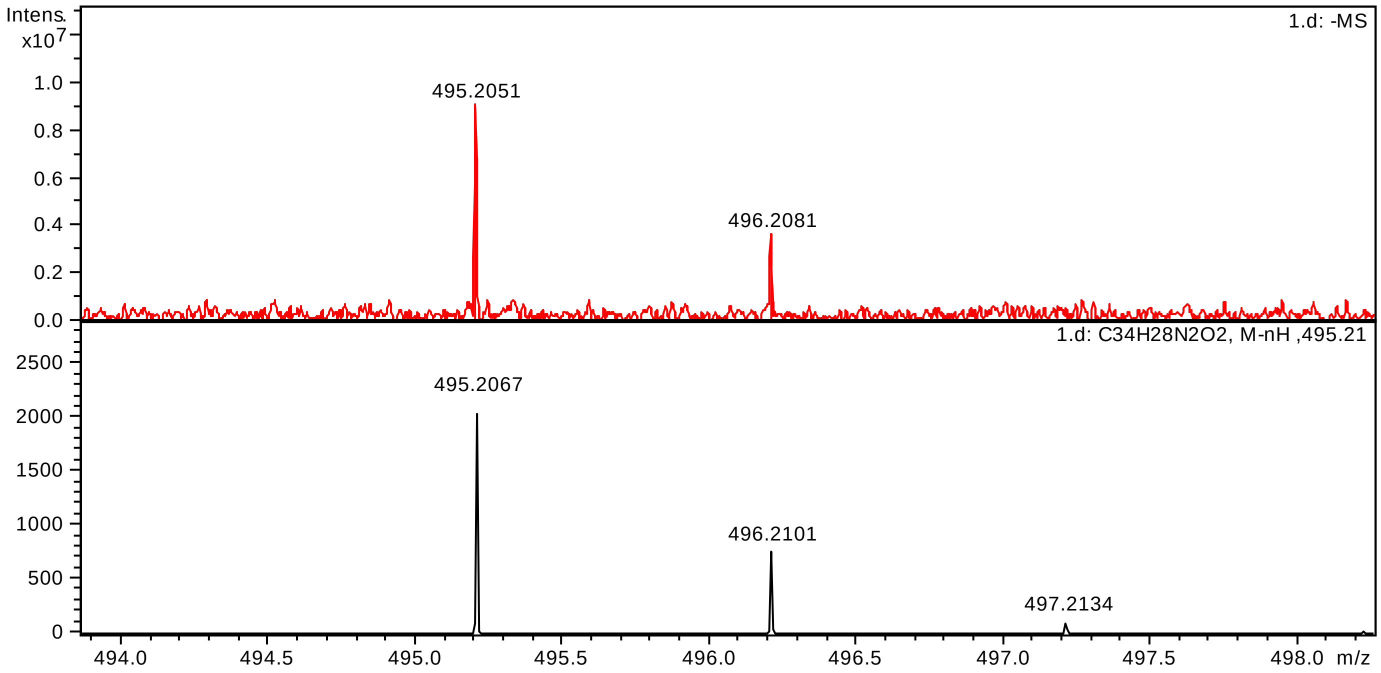

Figure S137. High resolution mass spectrum of **6** (ESI–TOF, top: experimental, bottom: simulated).

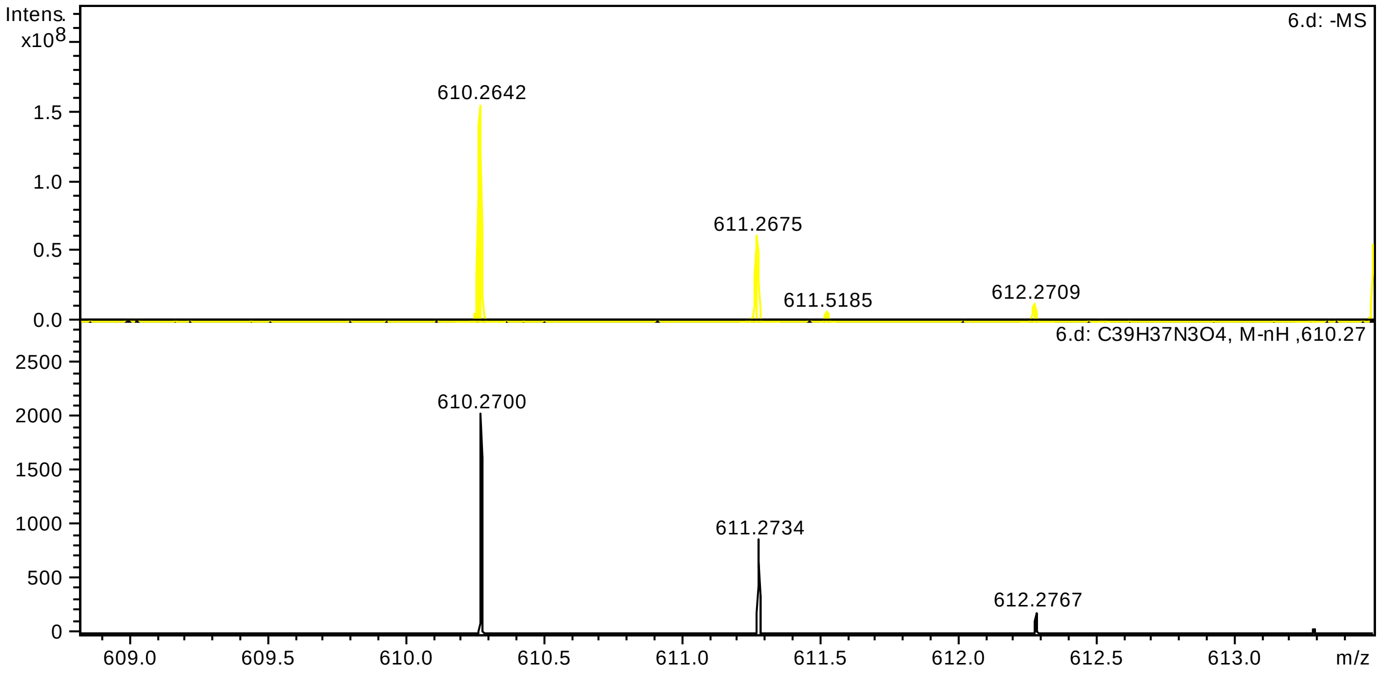

Figure S138. High resolution mass spectrum of **5** (ESI–TOF, top: experimental, bottom: simulated).

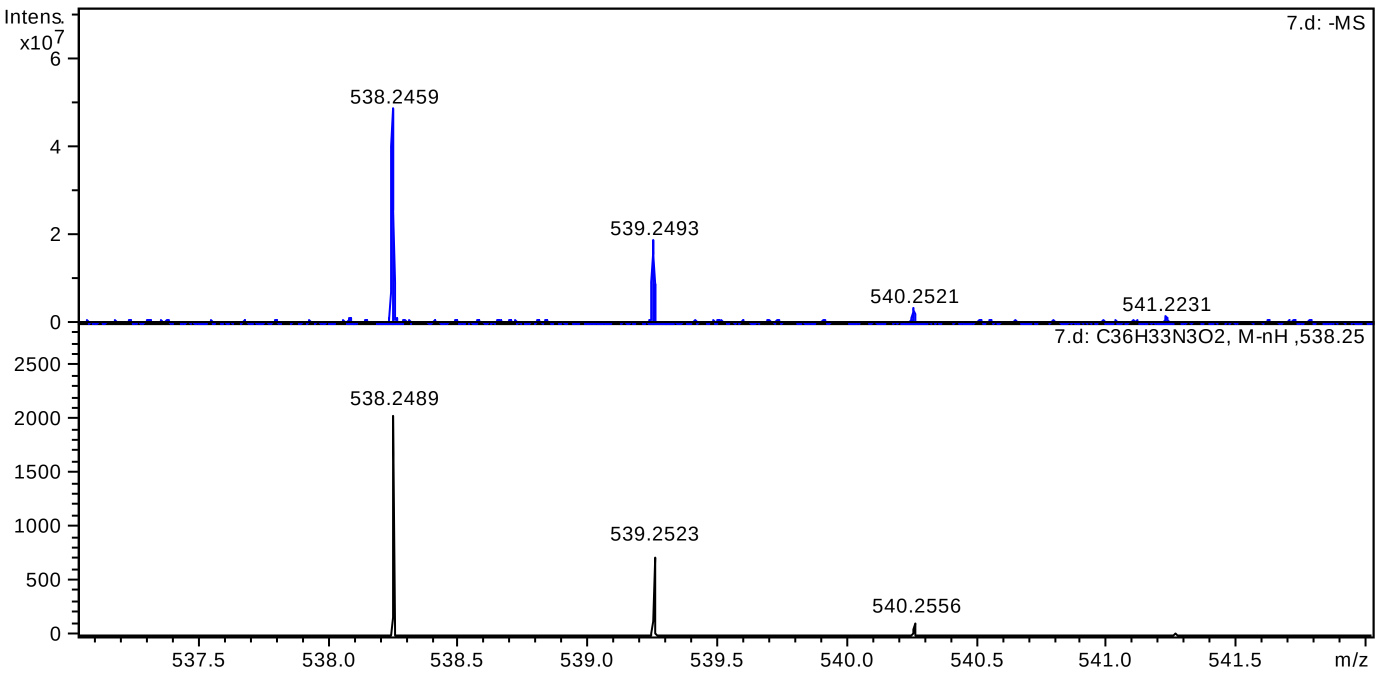

Figure S139. High resolution mass spectrum of **7** (ESI–TOF, top: experimental, bottom: simulated).

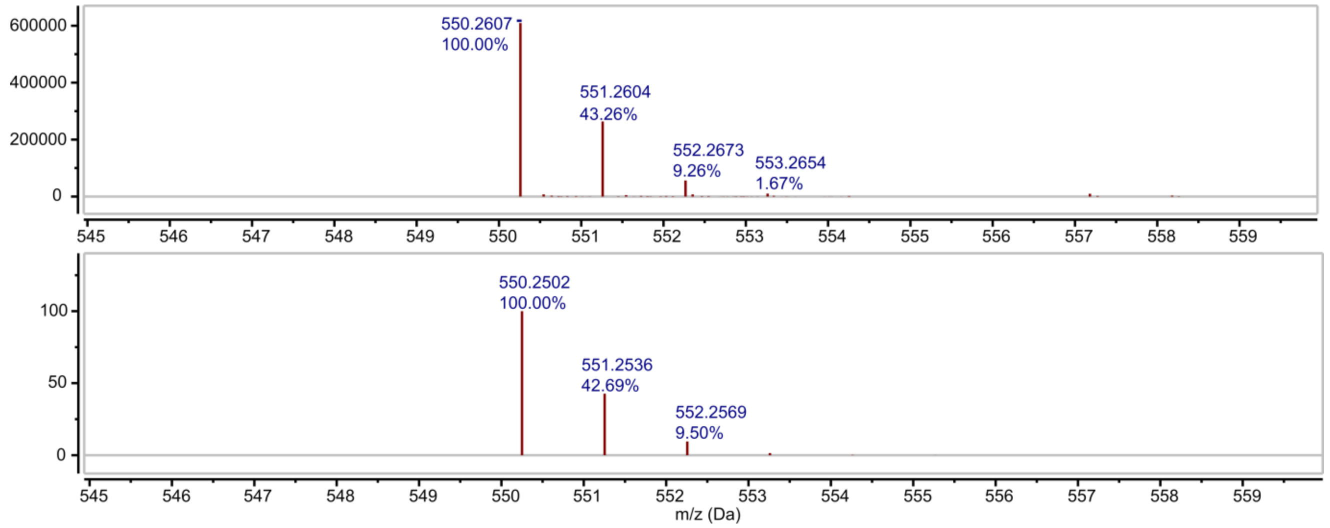

Figure S140. High resolution mass spectrum of **S3** (LC-MS, top: experimental, bottom: simulated).

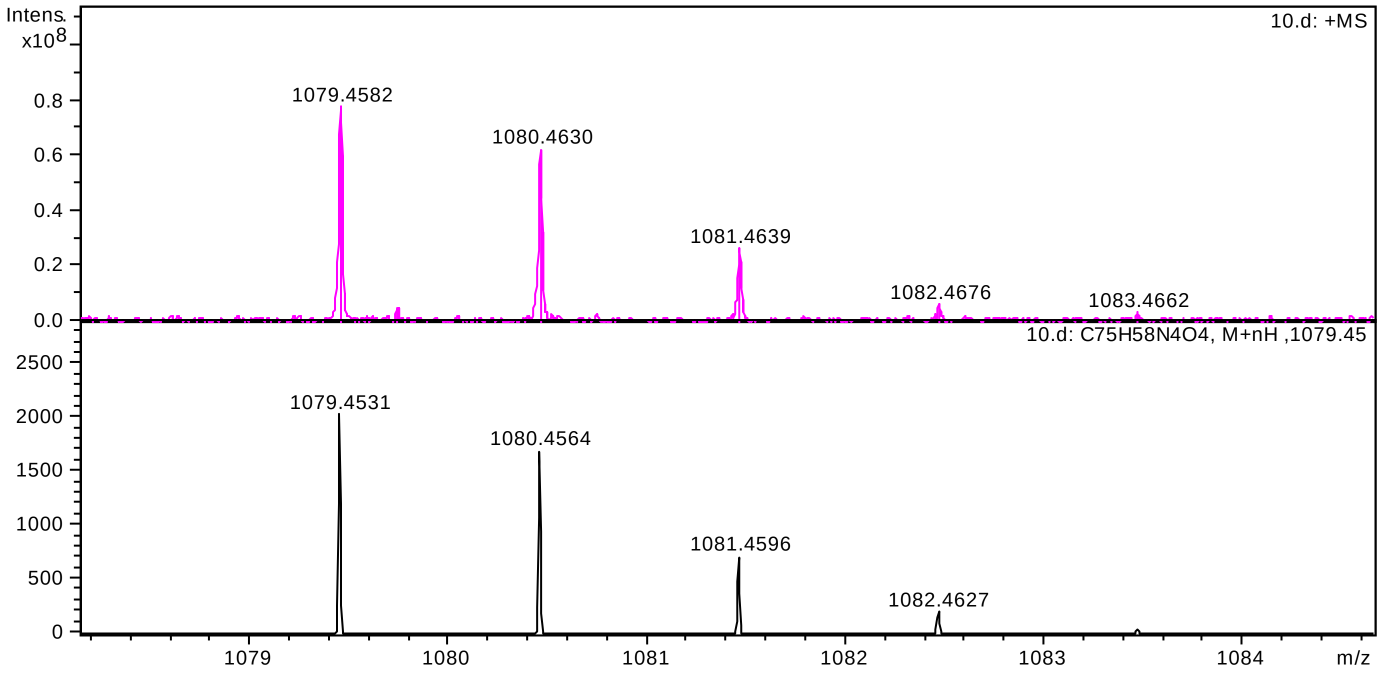

Figure S141. High resolution mass spectrum of **1a** (ESI–TOF, top: experimental, bottom: simulated).

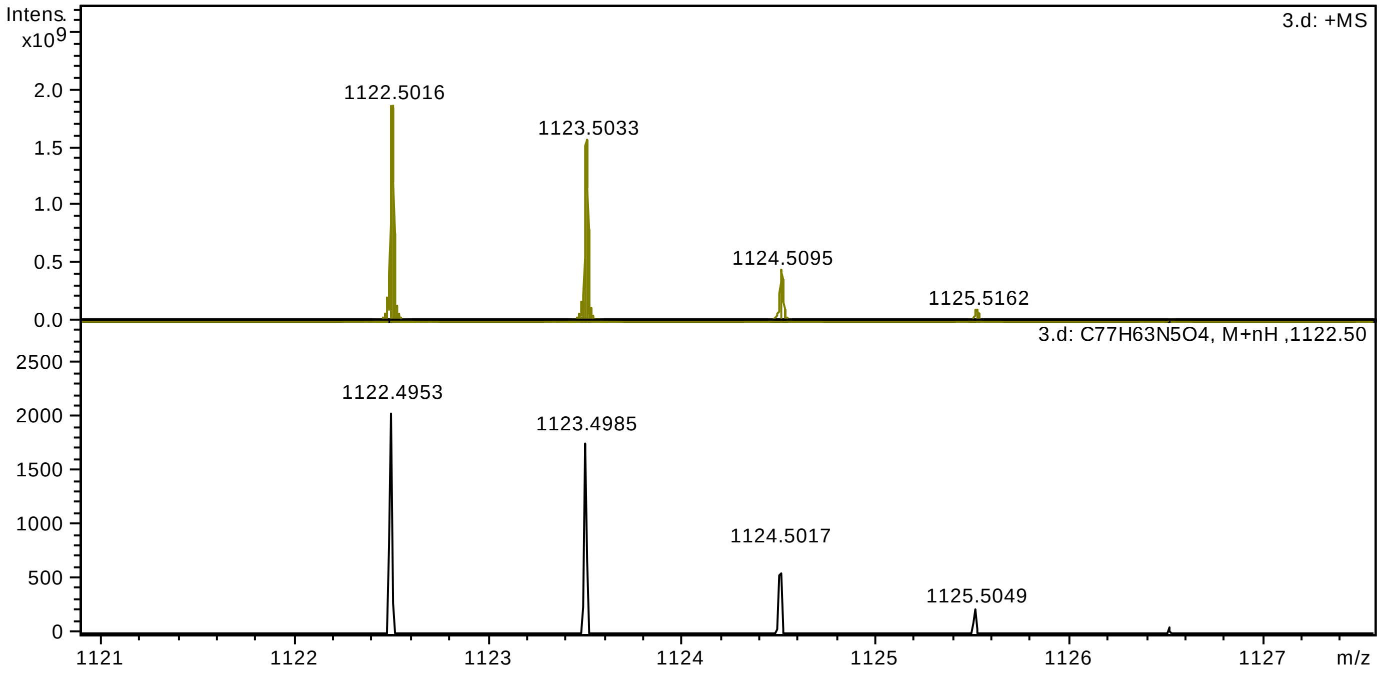

Figure S142. High resolution mass spectrum of **1b** (ESI–TOF, top: experimental, bottom: simulated).

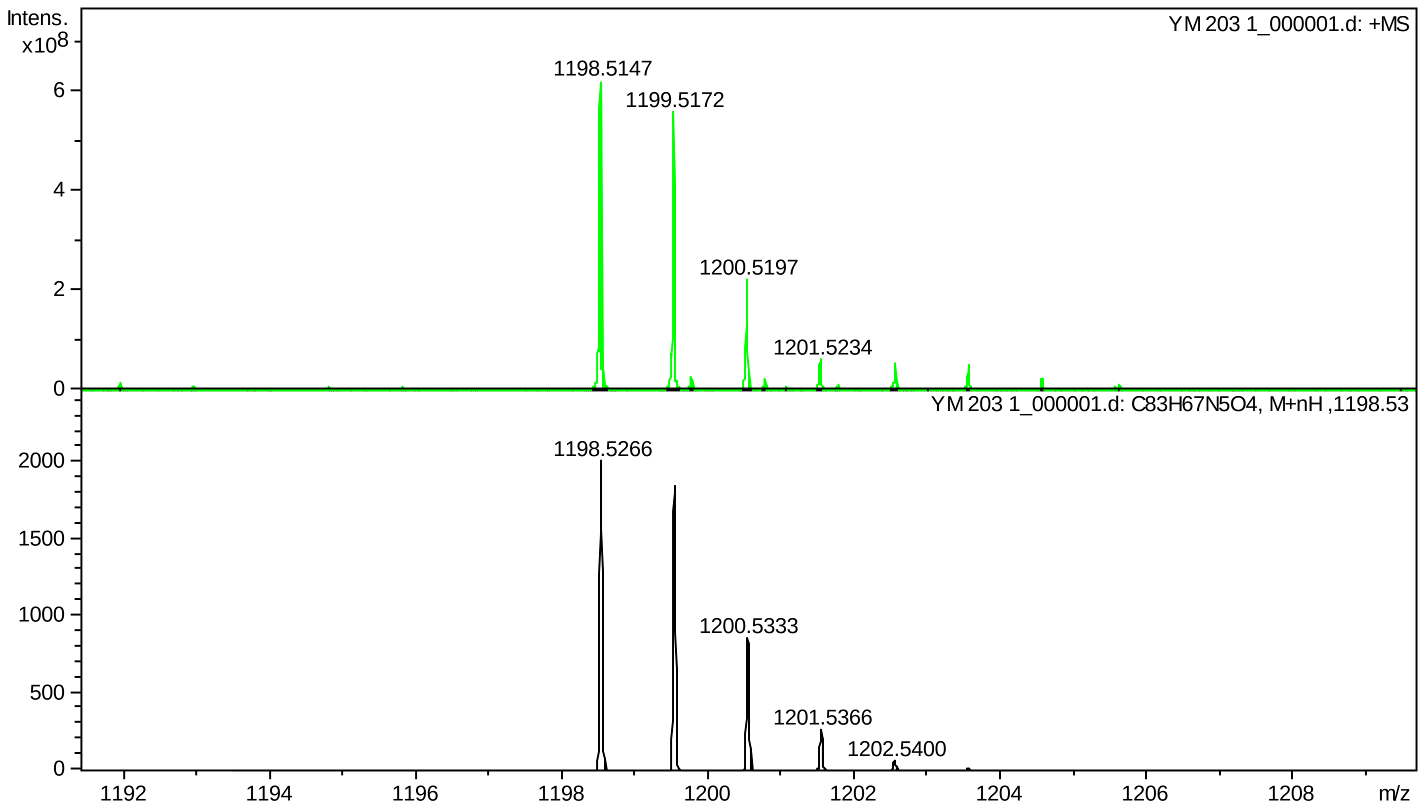

Figure S143. High resolution mass spectrum of **1c** (ESI–TOF, top: experimental, bottom: simulated).

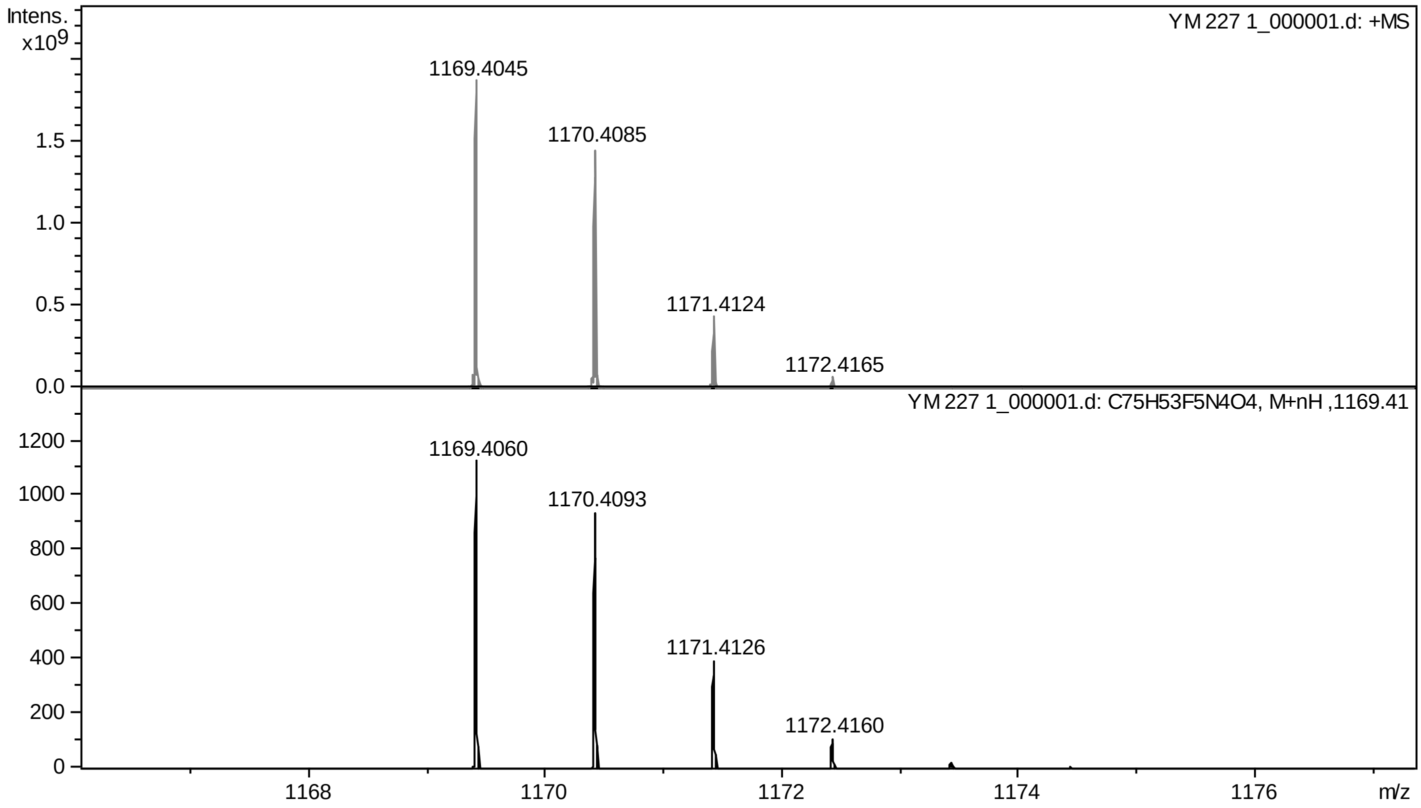

Figure S144. High resolution mass spectrum of **1d** (ESI–TOF, top: experimental, bottom: simulated).

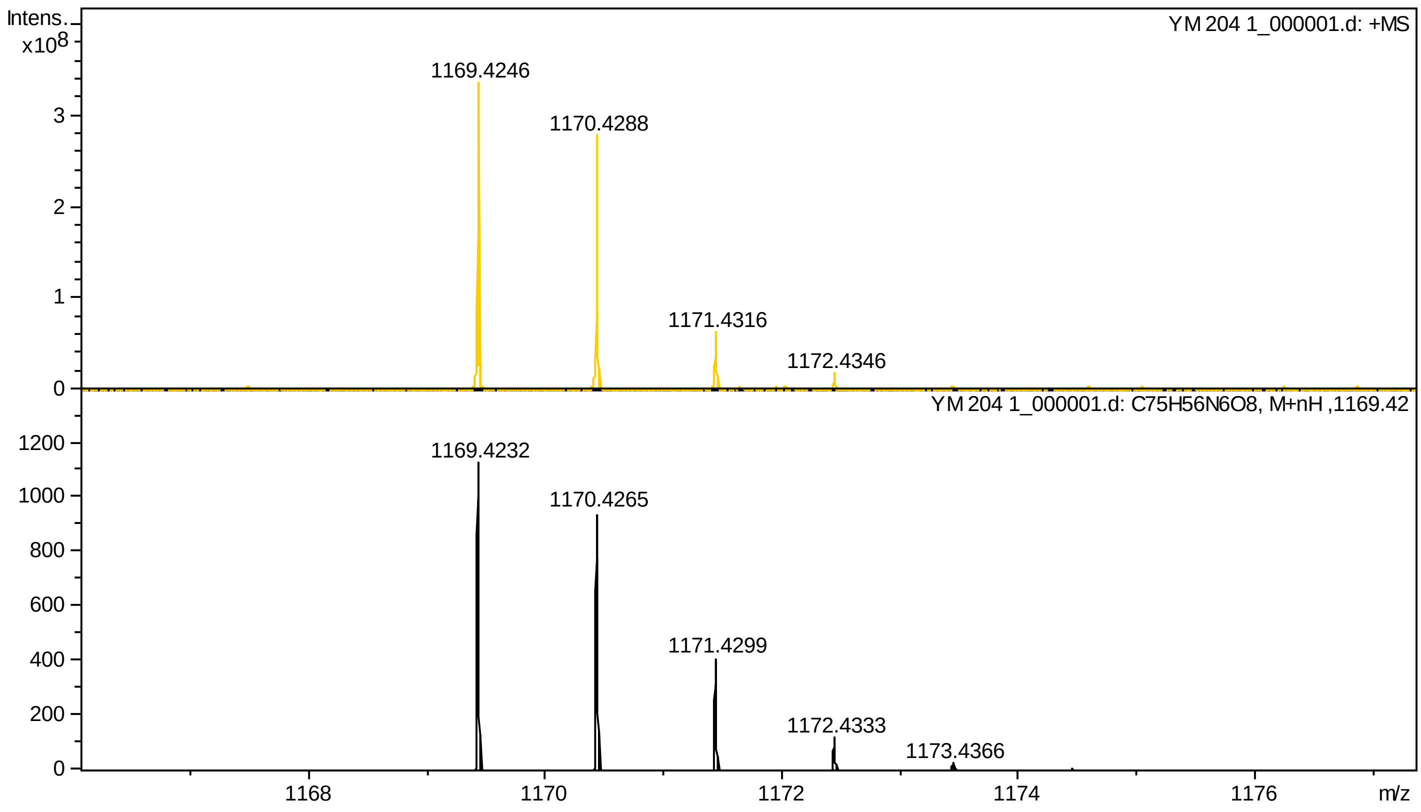

Figure S145. High resolution mass spectrum of **1e** (ESI–TOF, top: experimental, bottom: simulated).

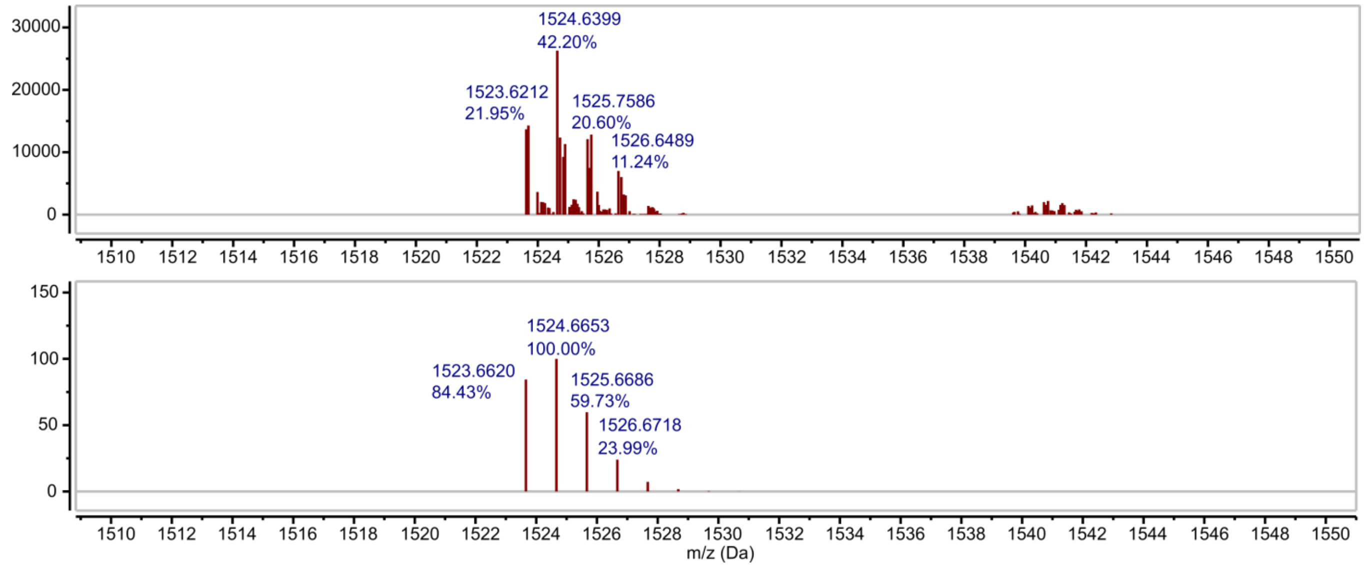

Figure S146. High resolution mass spectrum of **1f** (LC-MS, top: experimental, bottom: simulated).

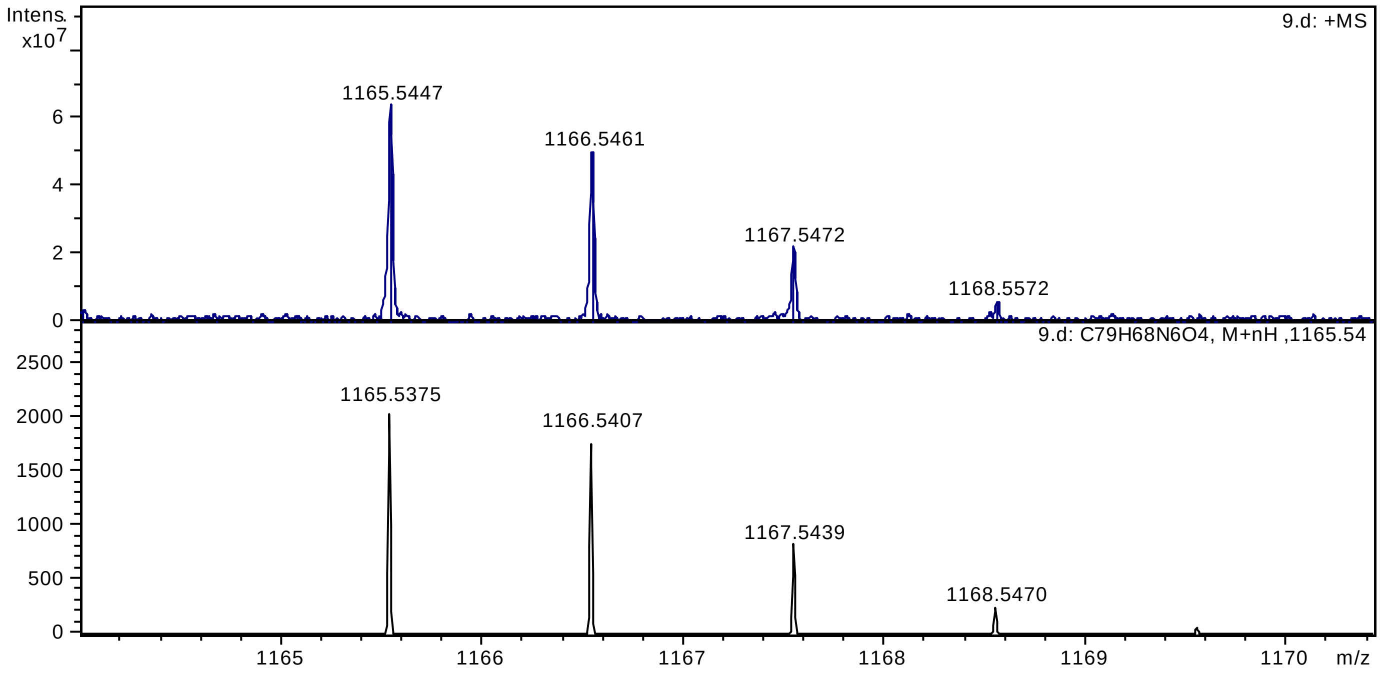

Figure S147. High resolution mass spectrum of **2a** (ESI–TOF, top: experimental, bottom: simulated).

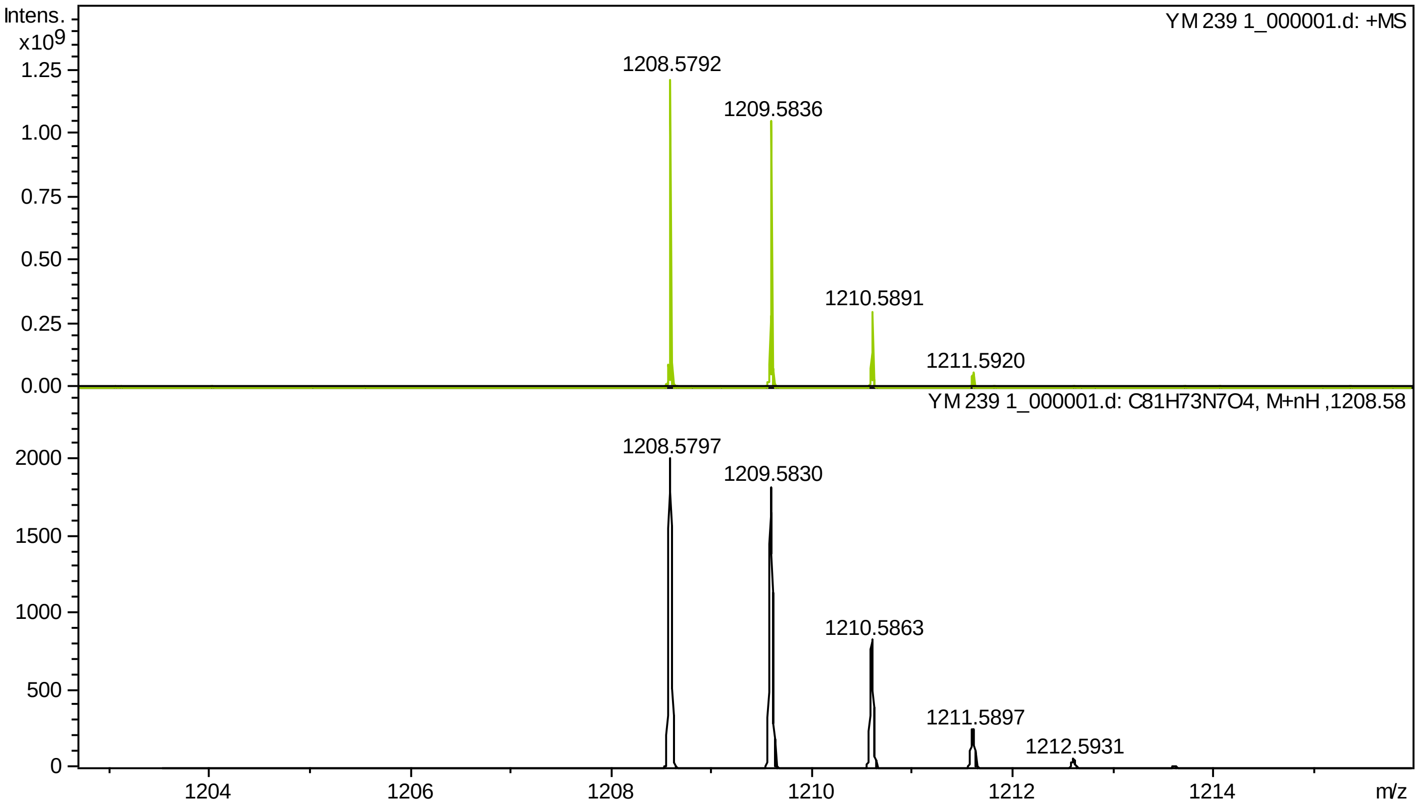

Figure S148. High resolution mass spectrum of **2b** (ESI–TOF, top: experimental, bottom: simulated).

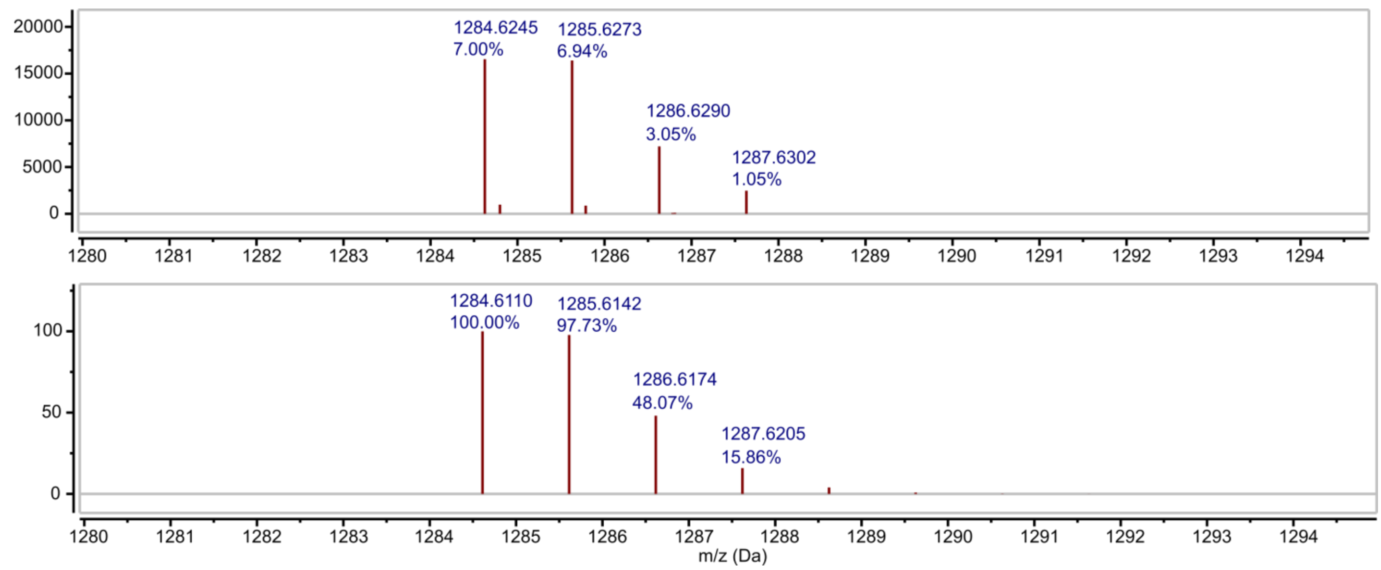

Figure S149. High resolution mass spectrum of **2c** (LC-MS, top: experimental, bottom: simulated).

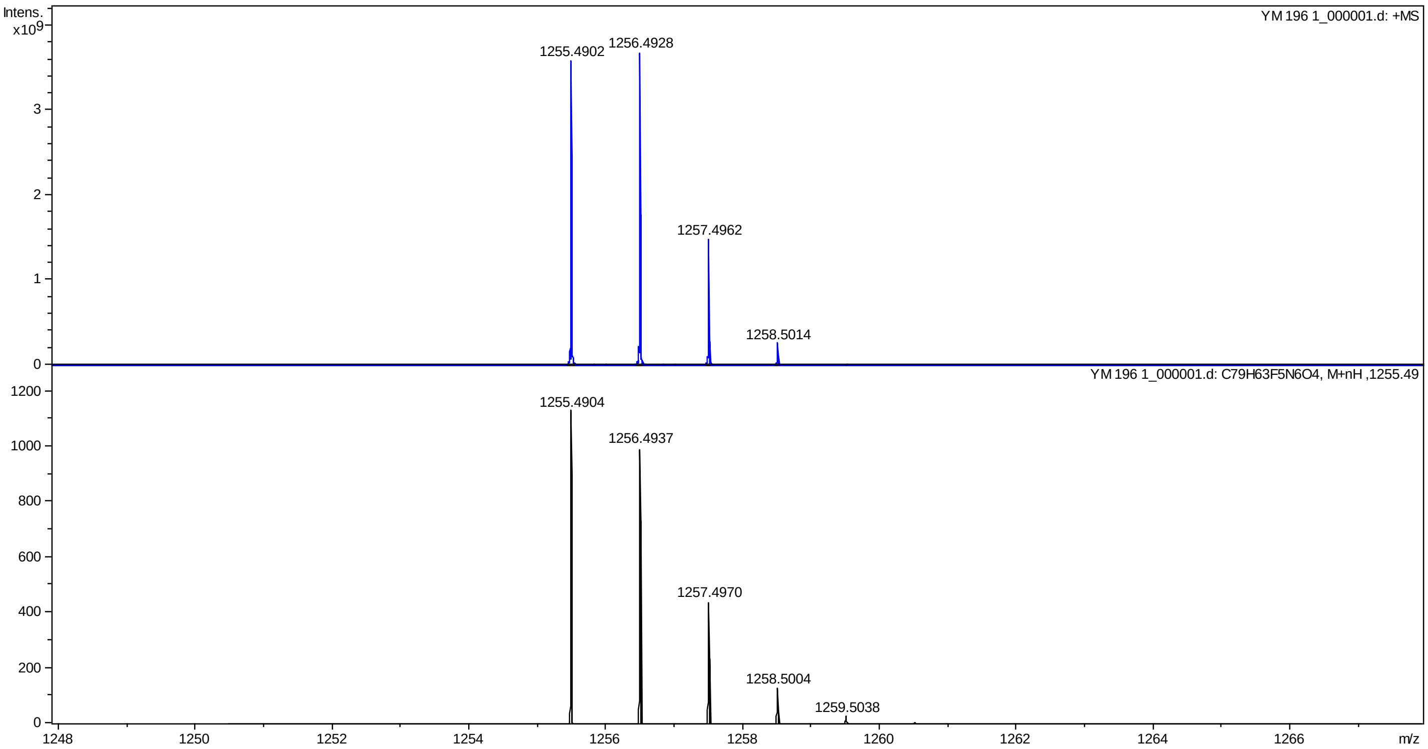

Figure S150. High resolution mass spectrum of **2d** (ESI–TOF, top: experimental, bottom: simulated).

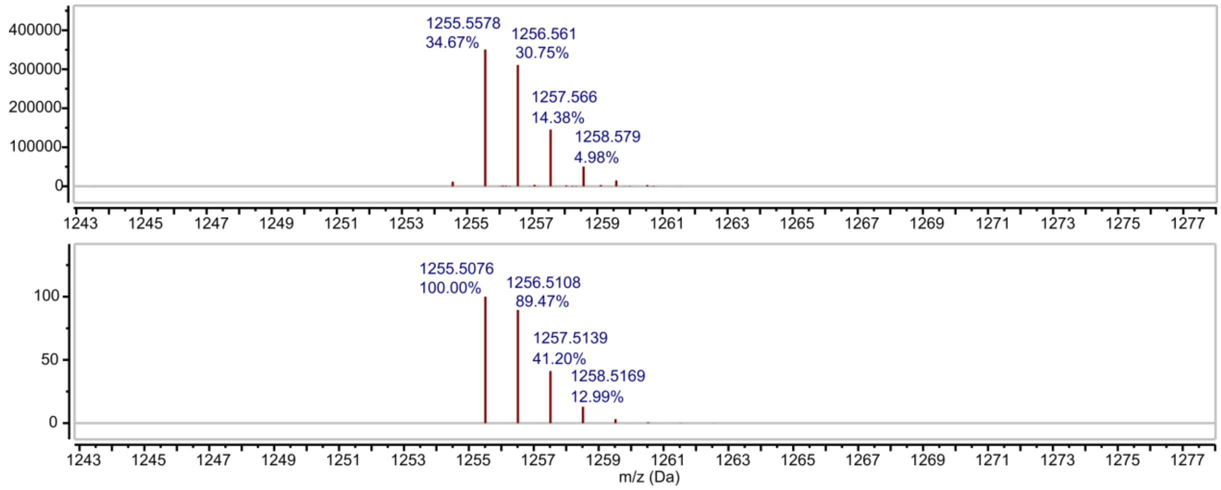

Figure S151. High resolution mass spectrum of **2e** (LC-MS, top: experimental, bottom: simulated).

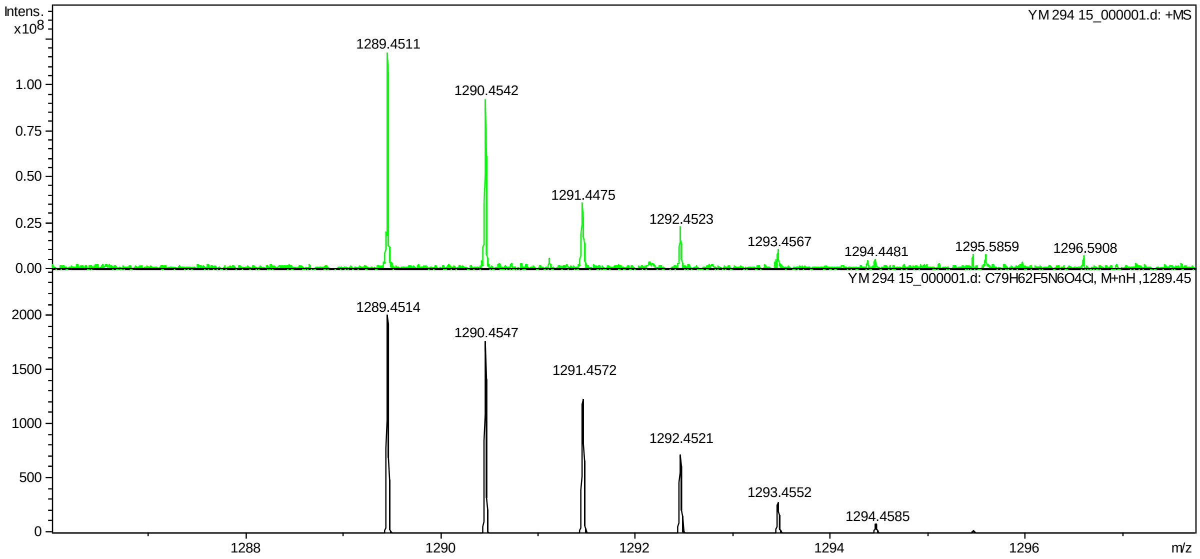

Figure S152. High resolution mass spectrum of **3d** (ESI–TOF, top: experimental, bottom: simulated).

References

[1] S. C. Virgil, in *Encycl. Reag. Org. Synth.*, American Cancer Society, **2001**.
[2] M. Żyła-Karwowska, L. Moshniaha, Y. Hong, H. Zhylitskaya, J. Cybińska, P. J. Chmielewski, T. Lis, D. Kim, M. Stępień, *Chem. – Eur. J.* **2018**, *24*, 7525–7530.
[3] Z. Jiang, C. Zhang, X. Wang, M. Yan, Z. Ling, Y. Chen, Z. Liu, *Angew. Chem. Int. Ed.* **2021**, *60*, 22376–22384.
[4] M. J. Frisch, G. W. Trucks, H. B. Schlegel, G. E. Scuseria, M. A. Robb, J. R. Cheeseman, G. Scalmani, V. Barone, G. A. Petersson, H. Nakatsuji, X. Li, M. Caricato, A. F. Izmaylov, G. Zheng, J. L. Sonnenberg, M. Hada, M. Ehara, K. Toyota, R. Fukuda, J. Hasegawa, M. Ishida, T. Nakajima, Y. Honda, O. Kitao, H. Nakai, T. Vreven, J. A. Montgomery, Jr., J. E. Peralta, F. Ogliaro, M. Bearpark, J. J. Heyd, E. Brothers, K. N. Kudin, V. N. Staroverov, R. Kobayashi, J. Normand, K. Raghavachari, A. Rendell, J. C. Burant, S. S. Iyengar, J. Tomasi, M. Cossi, J. M. Millam, M. Klene, C. Adamo, R. Gomperts, R. E. Stratmann, O. Yazyev, A. J. Austin, R. Cammi, C. Pomelli, J. W. Ochterski, R. L. Martin, K. Morokuma, V. G. Zakrzewski, G. A. Voth, P. Salvador, J. J. Dannenberg, S. Dapprich, A. D. Daniels, O. Farkas, J. B. Foresman, D. J. Fox, *Gaussian 16, Revision B.01*, Wallingford CT, **2016**.
[5] S. Grimme, C. Bannwarth, P. Shushkov, *J. Chem. Theory Comput.* **2017**, *13*, 1989–2009.
[6] C. Bannwarth, S. Ehlert, S. Grimme, *J. Chem. Theory Comput.* **2019**, *15*, 1652–1671.
[7] A. D. Becke, *Phys. Rev. A* **1988**, *38*, 3098–3100.
[8] A. D. Becke, *J. Chem. Phys.* **1993**, *98*, 5648–5652.
[9] C. Lee, W. Yang, R. G. Parr, *Phys. Rev. B* **1988**, *37*, 785–789.
[10] S. Grimme, S. Ehrlich, L. Goerigk, *J. Comput. Chem.* **2011**, *32*, 1456–1465.
[11] J. Tomasi, B. Mennucci, R. Cammi, *Chem. Rev.* **2005**, *105*, 2999–3094.
[12] N. K. S. Davis, A. L. Thompson, H. L. Anderson, *J. Am. Chem. Soc.* **2011**, *133*, 30–31.
